# Supplementary material for: Mammary-specific expression of Trim24 establishes a mouse model of human metaplastic breast cancer
Source: Nat Commun. 2021 Sep 10;12:5389. doi: 10.1038/s41467-021-25650-z (PMC8433435; doi:10.1038/s41467-021-25650-z)
Supplement: Supplementary file 1 — Supplementary Information [file 41467_2021_25650_MOESM1_ESM.pdf]

Supplementary Information

Mammary-specific expression of *Trim24* establishes a mouse model of human metaplastic breast cancer

Shah *et al.*

Supplementary Figures 1-5

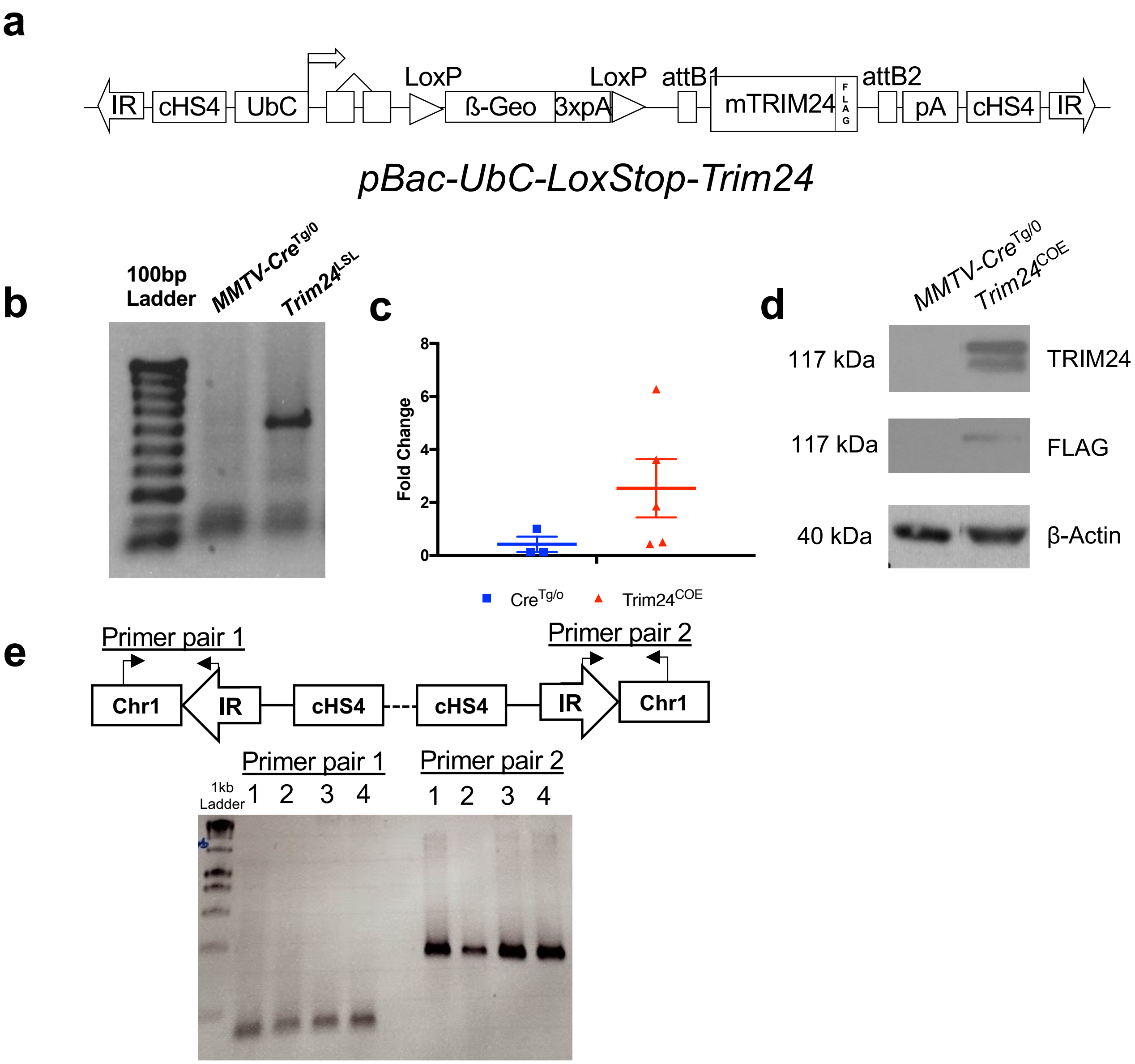
a
IR
cHS4
UbC
LoxP
ß-Geo
3xpA
LoxP
attB1
mTRIM24
FLAG
attB2
pA
cHS4
IR
pBac-UbC-LoxStop-Trim24
b
100bp Ladder
MMTV-Cre^Tg/0
Trim24^LSL
c
Fold Change
0
2
4
6
8
Cre^Tg/o
Trim24^COE
d
MMTV-Cre^Tg/0
Trim24^COE
117 kDa
TRIM24
117 kDa
FLAG
40 kDa
β-Actin
e
Primer pair 1
Primer pair 2
Chr1
IR
cHS4
cHS4
IR
Chr1
Primer pair 1
Primer pair 2
1kb Ladder
1 2 3 4
1 2 3 4

SUPPLEMENTARY FIGURE LEGENDS

Supplementary Figure 1 (Related to Figure 1): Generation of piggyBac-Ubc-TRIM24 conditional over expression mouse model. **a** Schematic representation of UBC-β-Geo-TRIM24-FLAG piggyBac construct. IR: Inverted Repeats, cHS4: beta-globin, UbC: human UbC promoter, β-Geo: Beta-galactosidase, 3XpA: polyadenylation sequence. **b** Genotyping analysis (PCR) of *MMTV-Cre*$^{Tg/0}$ and *Trim24*^{LSL} mice, showing positive band at 650 base pairs. This image is representative of >50 experiments. **c** qRT-PCR showing fold change of *Trim24* RNA expression in *Trim24*^{CO} mouse mammary glands compared to *MMTV-Cre*$^{Tg/0}$ (n= 5 biological replicates). Data represented in this graph as mean ± SEM. **d** Western blot analysis with TRIM24, Flag and β-Actin antibodies of *MMTV-Cre*$^{Tg/0}$ and *Trim24*^{COE} mouse mammary gland protein lysates. This image is representative of >10 experiments. **e** Schematic representation of primers used to validate the position of UBC-β-Geo-TRIM24-FLAG piggyBac transgene within chromosome 1q. Gel electrophoresis of PCR amplification of genomic DNA from 4 individual mice in a cohort shows derivation from a single founder. This image is representative of 3 experiments.

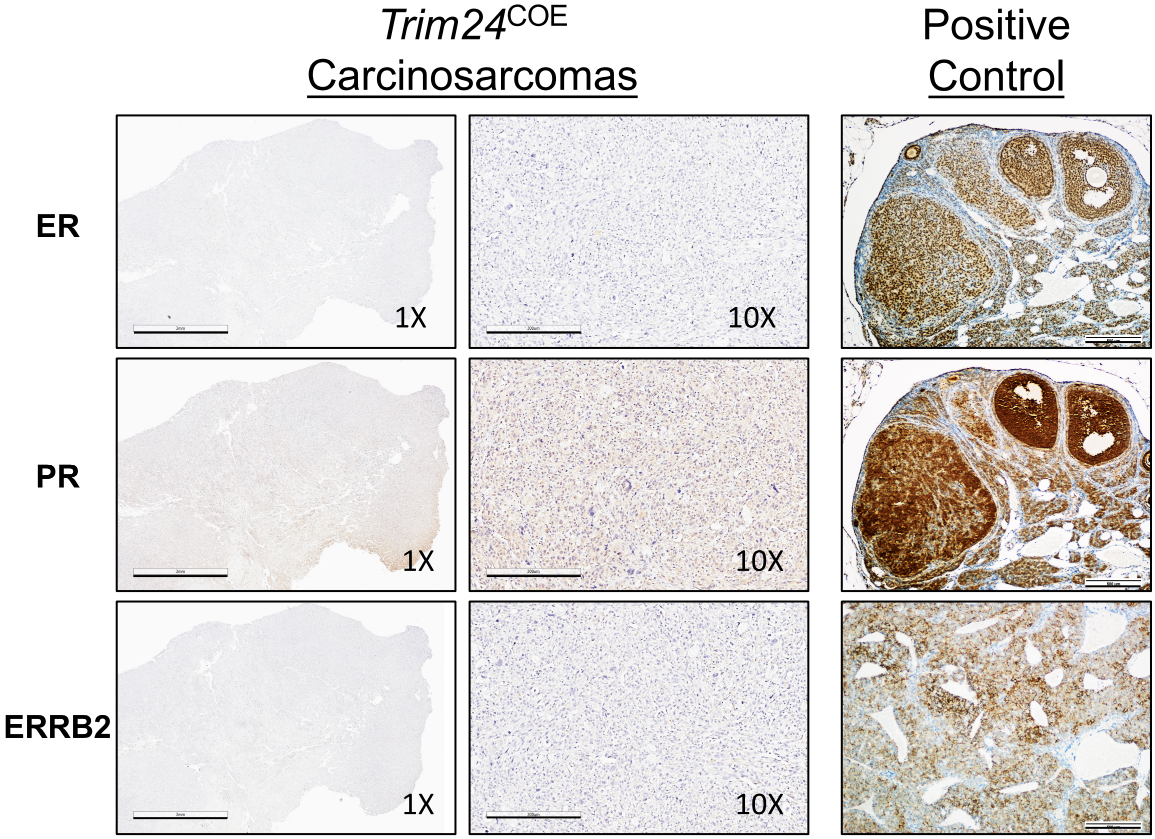

Trim24COE
Carcinosarcomas
Positive
Control
ER
PR
ERRB2
1X
10X
1X
10X
1X
10X

Supplementary Figure 2 (Related to Figure 2): IHC staining of *Trim24*^{COE} metaplastic carcinosarcomas for ER, PR and ERRB2 (HER2) at 1X and 10X magnifications (first two columns). The third column shows positive control IHC for each antibody: ER and PR IHC of a mouse uterus; ERRB2 IHC of a HER2-positive mouse mammary tumor (Scale bar= 500μM). Images are representative of 10 experiments. Scale bar = 3mM (1X) and 300μM (10X).

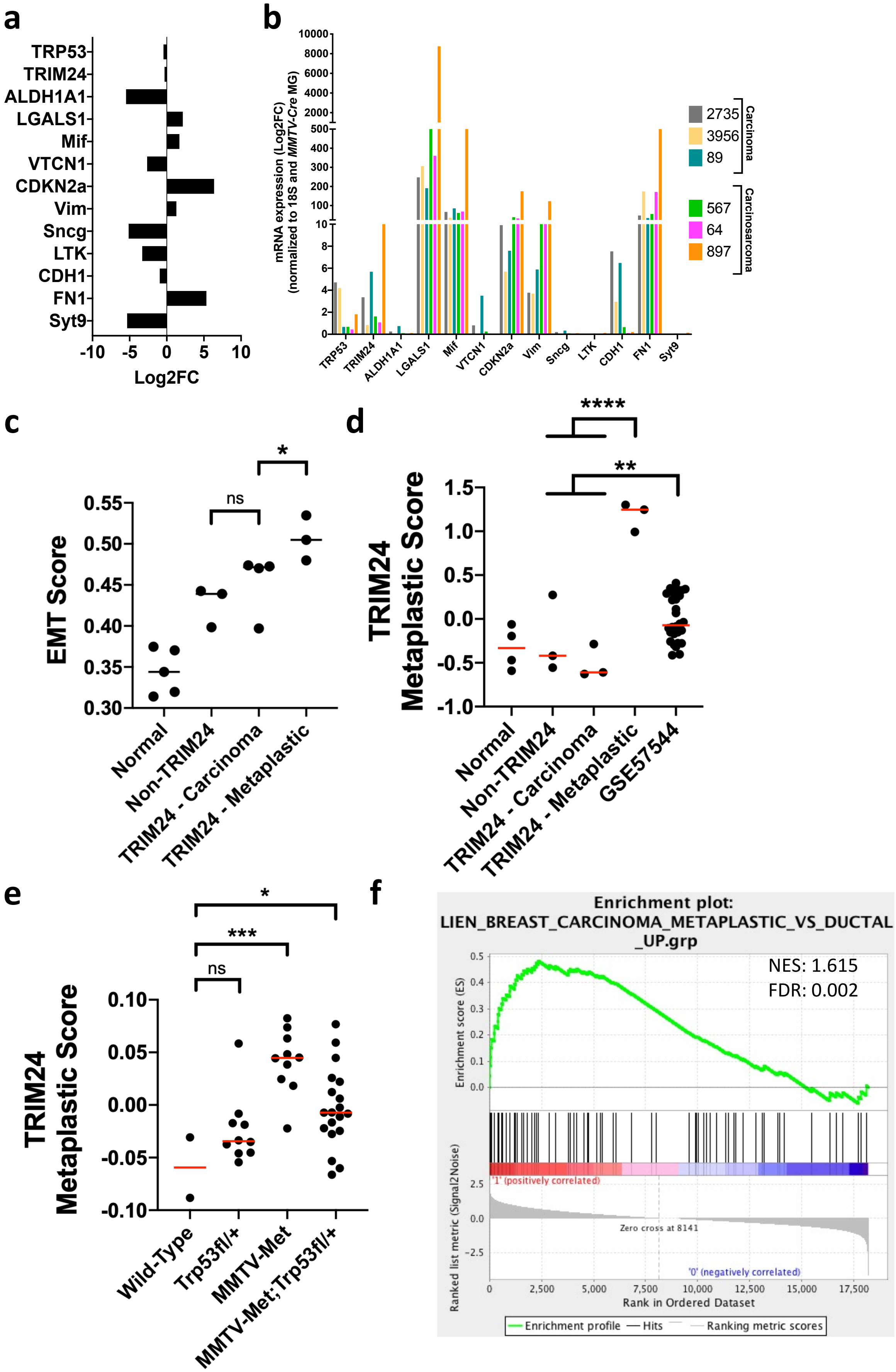

a
TRP53
TRIM24
ALDH1A1
LGALS1
Mif
VTCN1
CDKN2a
Vim
Sncg
LTK
CDH1
FN1
Syt9
-10
-5
0
5
10
Log2FC
b
mRNA expression (Log2FC)
(normalized to 18S and MMTV-Cre MG)
10000
8000
6000
4000
2000
500
400
300
200
100
10
8
6
4
2
0
2735
3956
89
Carcinoma
567
64
897
Carcinosarcoma
TRP53
TRIM24
ALDH1A1
LGALS1
Mif
VTCN1
CDKN2a
Vim
Sncg
LTK
CDH1
FN1
Syt9
c
EMT Score
0.55
0.50
0.45
0.40
0.35
0.30
ns
*
Normal
Non-TRIM24
TRIM24 - Carcinoma
TRIM24 - Metaplastic
d
TRIM24
Metaplastic Score
1.5
1.0
0.5
0.0
-0.5
-1.0
****
**
Normal
Non-TRIM24
TRIM24 - Carcinoma
TRIM24 - Metaplastic
GSE57544
e
TRIM24
Metaplastic Score
0.10
0.05
0.00
-0.05
-0.10
*
***
ns
Wild-Type
Trp53fl/+
MMTV-Met
MMTV-Met;Trp53fl/+
f
Enrichment plot:
LIEN_BREAST_CARCINOMA_METAPLASTIC_VS_DUCTAL_UP.grp
NES: 1.615
FDR: 0.002
Enrichment score (ES)
0.5
0.4
0.3
0.2
0.1
0.0
'1' (positively correlated)
Zero cross at 8141
'0' (negatively correlated)
Ranked list metric (Signal2Noise)
2.5
0.0
-2.5
0
2,500
5,000
7,500
10,000
12,500
15,000
17,500
Rank in Ordered Dataset
Enrichment profile
Hits
Ranking metric scores

Supplementary Figure 3 (Related to Figure 4): Assessment of genes differentially expressed in *Trim24*^{COE} tumors. **a** Log_2 fold change of expression of selected genes from RNA-Seq analysis of *Trim24*^{COE} metaplastic carcinosarcoma tumors. **b** qRT-PCR validation of individual tumor samples matched to selected genes from A. These values are normalized to 18S and delta-C_t values are normalized to *MMTV-Cre*$^{Tg/0}$ mammary gland analyses to calculate fold change. **c** Representative graph shows EMT pathway scores of Normal *MMTV-Cre*$^{Tg/0}$ mammary glands (n=5 independent samples), Non-TRIM24 driven mammary carcinomas (n= 3 independent tumors) , TRIM24-driven carcinomas (n= 4 independent tumors), and *Trim24*^{COE} metaplastic carcinosarcomas (n= 3 independent tumors). **d** Microarray expression of human breast metaplastic carcinoma (GSE57544) is compared to various genotypes of our cohort using TRIM24 metaplastic score. All samples are biologically independent replicates (normal: n= 4, Non-TRIM24, TRIM24-Carcinosarcoma and TRIM24- Metaplastic: n= 3 per group, and GSE57544: n=28). **e** The TRIM24 signature scores (Y-axis) are shown for biologically independent samples of wild type (WT) (n=2), Trp53$^{fl/+}$(n=10), MMTV-Met (n=10), and MMTV-Met;Trp53$^{fl/+}$ mammary cells (n=19). **f** GSEA enrichment plot of metaplastic breast cancer gene set aligned to RNA-Seq data of *Trim24*^{COE} tumors (1) compared to *MMTV-Cre*$^{Tg/0}$ mammary glands (0). Data represented in graph **c-e** as median values and p-values are calculated using one-way ANOVA for multiple comparison using Fisher's test. (ns- non-significant, * p<0.05, ** p<0.01, *** p<0.001, **** p<0.0001)

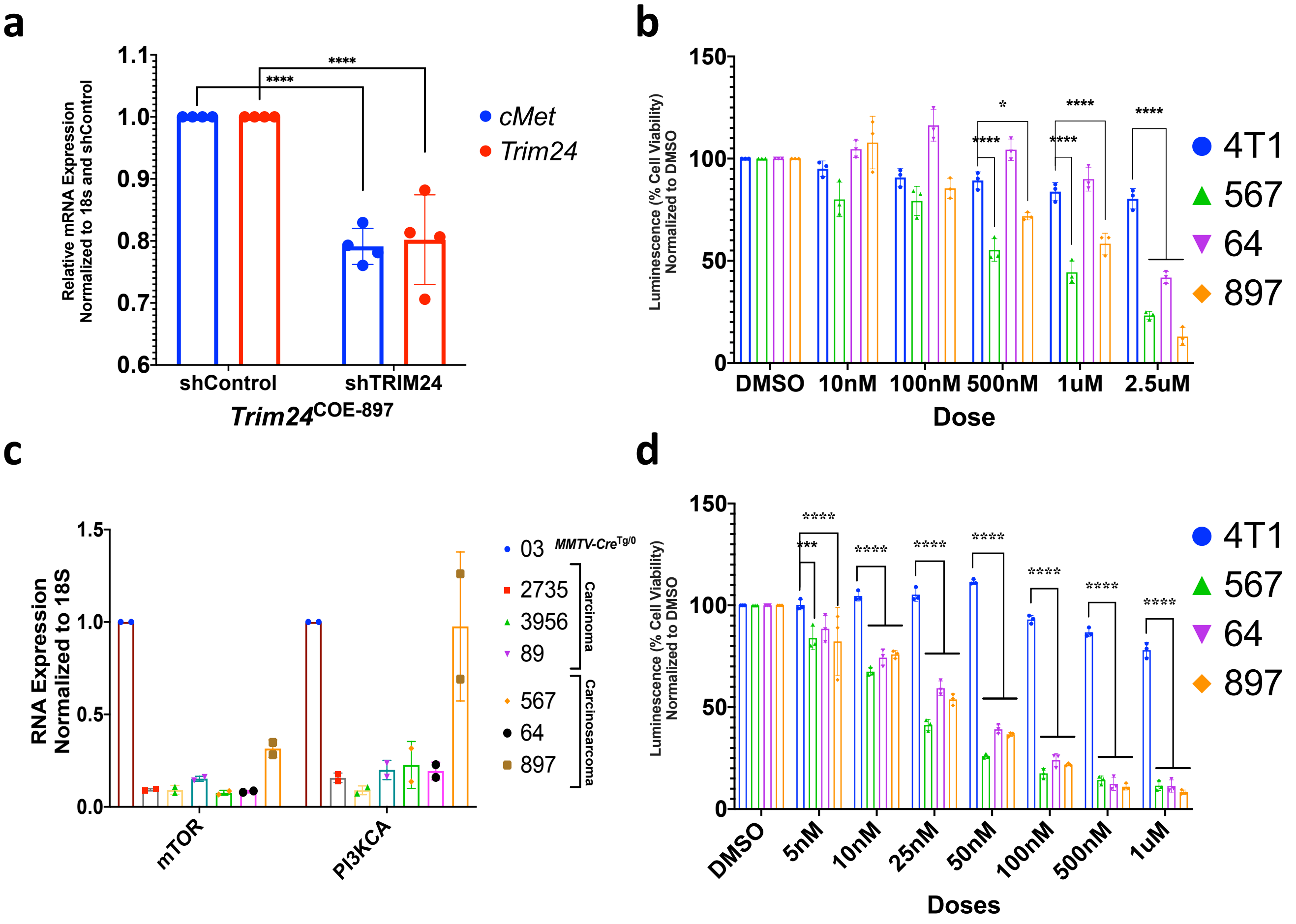

a
Relative mRNA Expression Normalized to 18s and shControl
1.1
1.0
0.9
0.8
0.7
0.6
****
****
cMet
Trim24
shControl
shTRIM24
Trim24COE-897
b
Luminescence (% Cell Viability) Normalized to DMSO
150
100
50
0
*
****
****
****
****
DMSO
10nM
100nM
500nM
1uM
2.5uM
Dose
4T1
567
64
897
c
RNA Expression Normalized to 18S
1.5
1.0
0.5
0.0
mTOR
PI3KCA
03
MMTV-CreTg/0
2735
3956
89
Carcinoma
567
64
897
Carcinosarcoma
d
Luminescence (% Cell Viability) Normalized to DMSO
150
100
50
0
****
***
****
****
****
****
****
****
DMSO
5nM
10nM
25nM
50nM
100nM
500nM
1uM
Doses
4T1
567
64
897

Supplementary Figure 4 (Related to Figure 5): TRIM24 activates c-MET and its downstream targets PI3K-mTOR to support metaplastic carcinosarcoma cell viability. **a** qRT-PCR analysis shows significant reduction of both *Trim24* and *cMet* at mRNA level in *Trim24* knockdown (shTRIM24) compared to control cell line (shControl) using *Trim24*^{COE} primary cell line 897 (n= 4 biological replicates). **b** Cell viability assay of 4T1 (mouse TNBC cell line, non-metaplastic) and *Trim24*^{COE} primary cell lines (567, 64 and 897) after treatment with PHA-665752, a selective small molecule inhibitor of c-Met. Percent cell viability is measured using a luminescence reader and values are normalized to DMSO, a vehicle control (n= 3 biological replicates). **c** qRT-PCR validation of mTOR and PI3KCA RNA expression in *Trim24*^{COE} tumors compared to *MMTV-Cre*$^{Tg/0}$ mammary gland (03) (n= 2 technical replicates). **d** Cell viability assay of 4T1 (mouse TNBC cell line, non-metaplastic) and *Trim24*^{COE} primary cell lines (567, 64 and 897) treated with Dactolisib, a dual ATP-competitive PI3K and mTOR inhibitor. Percent cell viability is measured using a luminescence reader and values are normalized to DMSO, a vehicle control (n= 3 biological replicates). Data represented as mean ± SEM in **a, c, d** and as median in **b**. p-values are calculated by two-way ANOVA for multiple comparison using Holm-Sidak method in **a, c, d**. (* p<0.05, ** p<0.01, *** p<0.001, **** p<0.0001)

a

PDX Model	BC Type	VB Subtype
PIM056	MpBC	BL2
PIM010	MpBC	M*
PIM077	MpBC	BL2
PIM228	MpBC	MSL
PIM091	TNBC	LAR
PIM172	TNBC	UNS
PIM262	TNBC	LAR
PIM190	TNBC	BL1

b

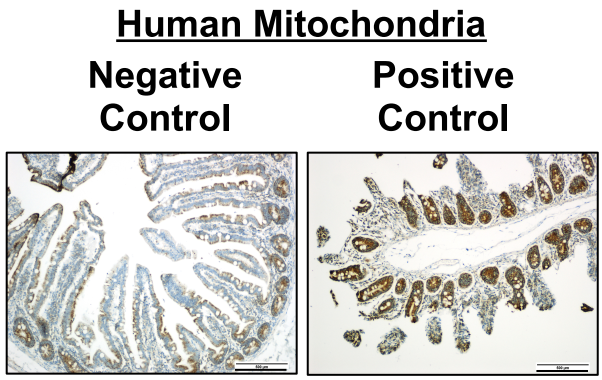

c

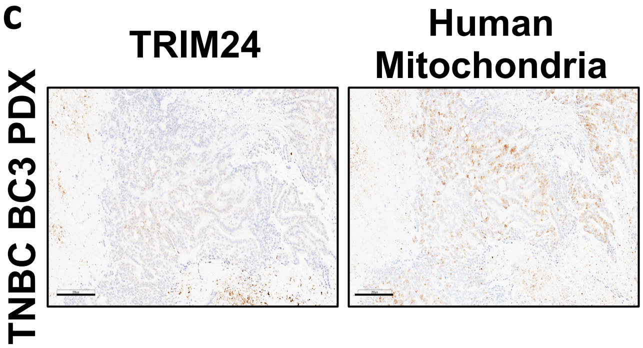

d

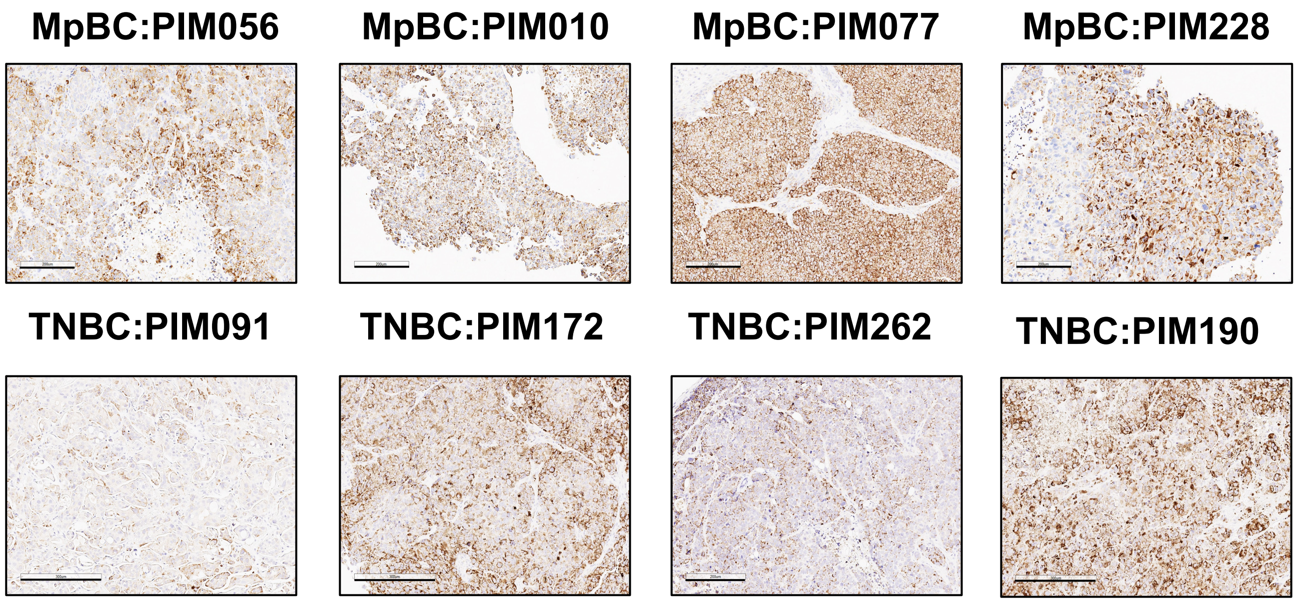

e

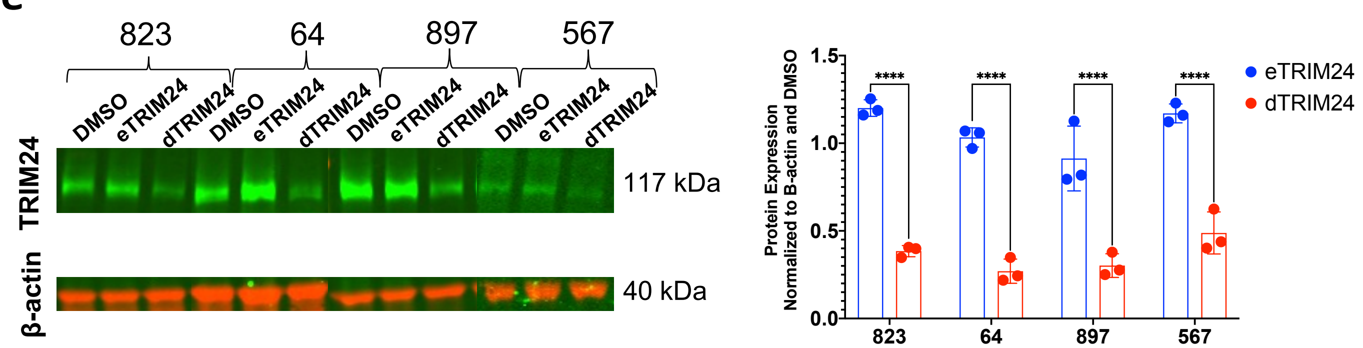

Supplementary Figure 5 (Related to Figure 7): Human mitochondria stain confirms expression of TRIM24 in MpBC and TNBC PDXs and degradation of TRIM24 via PROTAC reduces TRIM24 protein expressions in primary cell lines. **a** TNBC patient tumor samples were used to generate PDX mouse models. Breast cancer diagnosis (BC): MpBC and (non-MpBC) TNBC. Vanderbilt (VB) subtyping: BL- basal like, IM- immunomodulatory, LAR- luminal androgen receptor, M*- mesenchymal, MSL- mesenchymal stem-like and UNS- unstable tumor type **b** Representative images of positive and negative controls of human mitochondria stain using human intestine tissue. The image shown is representative of 3 experiments. Scale bar = 500μM. **c** Representative images of TNBC BC3 PDX for TRIM24 and human mitochondria stain at 10X magnification shows minimum expression of TRIM24. The image is representative of 3 experiments. Scale bar= 200μM **d** Representative images of human mitochondrial IHC staining of human MpBC PDXs compared to nonMpBC-TNBC PDXs. The image is representative of >5 experiments. Scale bars = 200μM for all MpBC set and PIM262, 300μM for PIM091, PIM172 and PIM190. **e** The PROTAC dTRIM24 reduced TRIM24 protein levels, as compared to eTRIM24 and DMSO treatments. A representative western blot analysis of control cell line, 823, and metaplastic carcinosarcoma derived cell lines, 64, 897 and 567, shows reduction of TRIM24 protein in dTRIM24 treatment compared to DMSO and eTRIM24 treatments. Green- TRIM24, Red- beta-actin (used as an endogenous control) (n= 3 biological replicates). The graph below shows Image J-analyzed/semi-quantification of three biological replicates of drug treatments. Data represented as mean $\pm$ SEM and p-values are calculated based on two-way ANOVA for multiple comparison using Holm-Sidak method (**** $p<0.0001$).

SUPPLEMENTARY DATA

Supplementary Data 1: List of antibodies used for western-blot analysis, immunohistochemistry and CyTOF.

Supplementary Data 2: List of primers used for Piggybac cloning, qRT-PCR and genotyping.

Supplementary Data 3: Table of discordant reads to identify Trim24-Flag insertion in mouse genome using 2 biological replicates.

Supplementary Data 4: List of TRIM24-driven mouse tumors and their pathological classification, necropsy details and samples selected for RNA-Seq.

Supplementary Data 5: TRIM24 IHC scoring on human MpBC patient samples and associated tumor subtypes.

Supplementary Data 6: List of differentially expressed genes of murine TRIM24-driven tumors and control mammary glands.

Supplementary Data 7: List of antibodies used in RPPA and normalized linear scores associated with Cre control and TRIM24-driven tumors.

Supplementary Data 8: Differentially expressed genes between TRIM24-driven carcinosarcoma and carcinoma tumors.

Supplementary Data 9: List of human TNBC and MpBC patient tumor details including response to chemotherapy, percentage of vimentin staining and tumor classification.
