# Supplementary material for: Mammary-specific expression of Trim24 establishes a mouse model of human metaplastic breast cancer
Source: Nat Commun. 2021 Sep 10;12:5389. doi: 10.1038/s41467-021-25650-z (PMC8433435; doi:10.1038/s41467-021-25650-z)
Supplement: Supplementary file 3 — Description of Additional Supplementary Files [file 41467_2021_25650_MOESM3_ESM.docx]

**Description of Additional Supplementary Files**

File Name: Supplementary Data 1

Description: List of antibodies used for western-blot analysis, immunohistochemistry and CyTOF.

File Name: Supplementary Data 2

Description: List of primers used for Piggybac cloning, qRT-PCR and genotyping.

File Name: Supplementary Data 3

Description: Table of discordant reads to identify Trim24-Flag insertion in mouse genome using 2 biological replicates.

File Name: Supplementary Data 4

Description: List of TRIM24-driven mouse tumors and their pathological classification, necropsy details and samples selected for RNA-Seq.

File Name: Supplementary Data 5

Description: TRIM24 IHC scoring on human MpBC patient samples and associated tumor subtypes.

File Name: Supplementary Data 6

Description: List of differentially expressed genes of murine TRIM24-driven tumors and control mammary glands.

File Name: Supplementary Data 7

Description: List of antibodies used in RPPA and normalized linear scores associated with Cre control and TRIM24-driven tumors.

File Name: Supplementary Data 8

Description: Differentially expressed genes between TRIM24-driven carcinosarcoma and carcinoma tumors.

File Name: Supplementary Data 9

Description: List of human TNBC and MpBC patient tumor details including response to chemotherapy, percentage of vimentin staining and tumor classification.
