# Supplementary material for: Mammary-specific expression of Trim24 establishes a mouse model of human metaplastic breast cancer
Source: Nat Commun. 2021 Sep 10;12:5389. doi: 10.1038/s41467-021-25650-z (PMC8433435; doi:10.1038/s41467-021-25650-z)
Supplement: Supplementary file 4 — Dataset 1 [file 41467_2021_25650_MOESM4_ESM.pdf]

Supplementary Table 1: List of antibodies used for western-blot analysis and immunohistochemistry.

Antibody	Target	Company	Application
TRIM24	TRIM24	Protein Technologies	WB, IHC
ER	Estrogen Receptor Alpha	Santa Cruz	IHC
PR	Progesterone Receptor	Abcam	IHC
ERBB2	ERBB2	Cell Signaling	IHC
VIMENTIN	VIMENTIN	Abcam	IHC
E-CADHERIN	E-CADHERIN	Protein Technologies	IHC
FLAG	M2(FLAG) epitope	Santa Cruz	IHC
FLAG-HRP	M2(FLAG) epitope	Sigma	IHC
K8	Keratin 8	Sigma	WB
K14	Keratin 14	Covance	IHC
TRIM24	TRIM24	Protein Technologies 14208-1-AP	CyTOF
p-PI3K(p85/p55)	Phospho-PI3K	Cell Signaling Tech 4228BF	CyTOF
pAKT	Phospho-AKT	BD Biosciences 560397	CyTOF
mTOR	mTOR	GenScript A01154	CyTOF
pan-CK	Pan-Cytokeratin	BioLegend 628602	CyTOF

**WB**	**Western blot**
**IHC**	**Immunohistochemistry**
