# Supplementary material for: Mammary-specific expression of Trim24 establishes a mouse model of human metaplastic breast cancer
Source: Nat Commun. 2021 Sep 10;12:5389. doi: 10.1038/s41467-021-25650-z (PMC8433435; doi:10.1038/s41467-021-25650-z)
Supplement: Supplementary file 5 — Dataset 2 [file 41467_2021_25650_MOESM5_ESM.pdf]

Supplementary Table 2: List of primers used for Piggybac cloning, qRT-PCR and genotyping.

piggyback Cloning

mTRIM24-FL F	CACCATGGAGGTGGCTGTGGAGAAG
mTRIM24-3XFlag-R	TTACTTGTCATCGTCATCCTTGTAATCGATATCATGATCTTTATAATCACCGTCATG GTCTTTGTAGTCCTTAAGCAGCTGGCGATCCTCGGTG

qRT-PCR

TRI24 F	CGGCCGGTGGTCCTT
TRI24 R	CAAACTGGCATCGAATGAC
VIMF1	CAGGCCAAGCAGGAGTCAAA
VIMR1	CTCCAGGGACTCGTTAGTGC
CDH1F1	AACCCAAGCACGTATCAGGG
CDH1R1	GAGTGTTGGGGGCATCATCA
TRP53F1	CATCCTGGCTGTAGGTAGCG
TRP53R1	TGGCAGTCATCCAGTCTTCG
MifF1	CGGACCGGGTCTACATCAAC
MifR1	GGACTCAAGCGAAGGTGGAA
Vtcn1F1	TGGGGCAGATCATCTTTTGGA
Vtcn1R1	CGTGATGAAGTGCTTGCCTGA
CDKN2aF1	CGCTCTGGCTTTCGTGAAC
CDKN2aR1	TTGCCCATCATCATCACCTGG
FN1F1	TACGCCATTGGAGAGGAGTG
FN1R1	GGCACCATTTAGATGAATCGCA
Aldh1A1F1	TCAGGAGTTTACATCAACTGGGA
Aldh1A1R1	ACCCCAAACTCCAACCAAGA
Lgals1F1	GCCAAGAGCTTTGTGCTGAA
Lgals1R1	TGGGCATTGAAGCGAGGATT
LTKF1	CTACAGCCTCTGGCAGTCAC
LTKR1	TGGCAGTCTTTGAAAGGGCA
SYT9F1	GACCCAGATATCTCTGTGAGCCTG
SYT9R1	AGACACCAAAAAGGGCCAGA
SNCGF1	GTATGTGGGCACCAAAACCA
SNCGR1	CAGCTTCACTCACGGCATTG
cMETF1	GTGCCCGAAGTGTAAGTCCA
cMETR1	TGCATGCTCCATTCTGAGAGG
cMETF2	TGATCATTGGTGCGGTCTCA
cMETR2	AGCGAACTAATTCACTGCCCA
aktF1	GCCGCCTGATCAAGTTCTCC
aktR1	TTCAGATGATCCATGCGGGG
mTORF1	CCGCTACTGTGTCTTGGCAT
mTORR1	CAGCTCGCGGATCTCAAAGA
pi3kcaF1	TGCAGAGGGTCAGAGCAATG
pi3kcaR1	CTAGGATTCGTGGGGGCATC
pi3kcaF2	TCATGGATGCTTTGCAGGGT
pi3kcaR2	AACCACAGTGGCCTTTTTGC

Genotyping Primers

Trim24 F (P23)	AATGAAGACTGGTGTGCTGTTTGT
Trim24 R (P24)	TCGTCTGAGTCGTCACTGAACTTAC
Cre F	GCGGTCTGGCAGTAAAAACTATC
Cre R	GTGAAACAGCATTGCTGTCACTT
P53 X6	AGCGTGGTGGTACCTTATGAGC
P53 X7	GGATGGTGGTATACTCAGAGCC
P53 Neo	GCTATCAGGACATAGCGTTGGC
