# Supplementary material for: Mammary-specific expression of Trim24 establishes a mouse model of human metaplastic breast cancer
Source: Nat Commun. 2021 Sep 10;12:5389. doi: 10.1038/s41467-021-25650-z (PMC8433435; doi:10.1038/s41467-021-25650-z)
Supplement: Supplementary file 6 — Dataset 3 [file 41467_2021_25650_MOESM6_ESM.pdf]

Supplementary Table 3: Table of discordant reads to identify Trim24-Flag insertion in mouse genome using 2 biological replicates.

ReadID	Flag	Vector	Start	MapQ	CIGAR	Chr	Start2	Seq
J00180:83:H22V2BBXY:8:2112:18923:24788	81	trim24_vector	1253	60	75M	19	4180516	AACTCTTTTTCCGAAGGTAACTGGCTTCAGCAGAGCGCAGATACCAAATACTGTTCTTCTAGTGTAGCCGTAGTA
J00180:83:H22V2BBXY:8:1209:20466:28797	81	trim24_vector	1884	60	75M	19	22915592	AGCCGAACGACCGAGCGCAGCGAGTCAGTGAGCGAGGAAGCGGAAGAGCGCCCAATACGCAAACCGCCTCTCCCC
J00180:83:H22V2BBXY:8:2204:24271:31769	129	trim24_vector	3148	0	40M35S	1	10940389	CGTCCCTGGAGGTGATGAAGAACGGTGGGGATGTGGCACTCAGGCACGTGGCAGGTGGGCACAGGGGGTGTGGTG
J00180:83:H22V2BBXY:8:2202:20405:16172	177	trim24_vector	3237	0	12S63M	12	38141962	GTCAAAACCTCAGATCTGGGAGGGCTTTTCCAGCCTTCATGATTTGACGATTGTATGAACATCTACATGGCAATT
J00180:83:H22V2BBXY:8:2202:22587:15451	1201	trim24_vector	3237	0	12S63M	12	38141962	GTCAAAACCTCAGATCTGGGAGGGCTTTTCCAGCCTTCATGATTTGACGATTGTATGAACATCTACATGGCAATT
J00180:83:H22V2BBXY:8:2106:26991:34231	161	trim24_vector	3342	0	71M2D4M	17	69719441	TCCAGGCAAGCTCTTCCACCCCTTCTGCTTGCATCCAGACACCATCAAACATGCAGGCTCAGACACGGGGAAGCA
J00180:83:H22V2BBXY:8:2203:3711:12919	161	trim24_vector	3346	0	42M33S	9	108241122	GGCAAGCTCTTCCACCCCTTCTGCTTGCATCCAGACACCATCCCACAGAGACTGACCACCCCACAGAGACTGACT
J00180:83:H22V2BBXY:8:2128:16985:46082	145	trim24_vector	3377	0	75M	8	92937552	CAGACACCATCAAACATGCAGGCTCAGACACAGGGACCAGCAGTGTCTGTGGCCTTTTTGTGCTCCTCTCCATGC
J00180:83:H22V2BBXY:8:2127:32319:6677	129	trim24_vector	3400	0	75M	9	104967896	TCAGACACAGGGACCAGCAGAGCCTGTGGCCAATTTGTGCGCCTCTCCATGCTGGGGTTTAACGTGCTCTTTGTC
J00180:83:H22V2BBXY:8:1104:2412:11688	145	trim24_vector	3469	0	75M	9	40477302	TTTGTCCTTCTATCCTATCTTCTTATCCTTAAGGCTGTTCTGAACGCTGTGACTTGGAGAGTGTCCCAGAGCCCT
J00180:83:H22V2BBXY:8:1108:29640:38609	177	trim24_vector	3499	0	75M	X	145552353	AAGGCTGTTCTGAACGCTGTGACTTGGAGAGTGTCCCAGAGCCCTCAACACCTGCATGTCCCACGTCCATGCTGT
J00180:83:H22V2BBXY:8:2109:16610:49019	177	trim24_vector	3579	0	75M	2	136711412	ACTTCCTTATCCCCAAGATCTGCCTCTCCGTGATGCACTGAATTGGCAAACATGTGTCACCCCAGACCAACAATN
J00180:83:H22V2BBXY:8:2214:19248:31646	145	trim24_vector	3775	0	25S50M	18	50260846	GGAGTGTGTGGGTAGGAGAGCAGGGTGCCCAAGGAGGCTGTGGATGCCCCATCCCTGCAGGCATTCAAGGCCAGG
J00180:83:H22V2BBXY:8:1125:8044:23768	113	trim24_vector	3791	0	75M	18	51408257	GGATGCCCCATCCCTGCAGGCATGCAAGGCCAGGCTGGATGTGGCTCTGGGCAGCCTGGGCTGCTGGTTGATGCC
J00180:83:H22V2BBXY:8:1109:6563:36534	129	trim24_vector	4481	60	28S47M	16	27215565	CCACACCCGGCGCGGCCTTATATAATCATCGGCGTTTCTGCGGAGGGATCTCCGTGGGGCGGTGAACGCCGATGA
J00180:83:H22V2BBXY:8:1109:6563:36534	401	trim24_vector	4517	60	39H36M	16	27215565	AACGCCGATGATTATATAAGGCCGCGCCGGGTGTGG
J00180:83:H22V2BBXY:8:2215:25134:15856	81	trim24_vector	4663	60	75M	2	135704973	CCGCCGGGCCGCTCGGTGGGACGGAAGCGTGTGGAGAGACCGCCAAGGGCTGTAGTCTGGGTCCGCGAGCAAGGT
J00180:83:H22V2BBXY:8:2212:3782:6501	1105	trim24_vector	5159	60	75M	5	101427655	TTTGGTCGGTTTTAAGTACCTATCTTCTTAAGTAGCTGAAGCTCCGGTTTTGAACTATGCGCTCGGGGTTGGCGA
J00180:83:H22V2BBXY:8:2212:3873:6378	81	trim24_vector	5159	60	75M	5	101427655	TTTGGTCGGTTTTATGTACCTATCTTCTTAAGTAGCTGAAGCTCCGGTTTTGAACTATGCGCTCGGGGTTGGCGA
J00180:83:H22V2BBXY:8:1224:23064:41352	129	trim24_vector	5204	60	53M22S	17	78104787	GGTTTTGAACTATGCGCTCGGGGTTGGCGAGTGTGTTTTGTGAAGTTATTTAGCACTAGAGGTGTTCTGTATTTG
J00180:83:H22V2BBXY:8:1224:23784:43058	1153	trim24_vector	5204	60	53M22S	17	78104787	GGTTTTGAACTATGCGCTCGGGGTTGGCGAGTGTGTTTTGTGAAGTTATTTAGCACTAGAGGTGTTCTGTATTTG
J00180:83:H22V2BBXY:8:2212:30472:10124	145	trim24_vector	5249	60	11S64M	11	107529015	GTATTCTTGAATTTTTTAGGCACCTTTTGAAATGTAATCATTTGGGTCAATATGTAATTTTCAGTGTTAGACTAG
J00180:83:H22V2BBXY:8:1209:32552:22766	97	trim24_vector	9331	60	57M18S	X	106203640	NCGATTCGCAGCGCATCGCCTTCTATCGCCTTCTTGACGAGTTCTTCTGAGGGGATCGATCCGCTGTAAGTCTGC
J00180:83:H22V2BBXY:8:1222:15615:10018	177	trim24_vector	10501	45	13S62M	1	154057290	ATCGAGGGACCTAATAACTTCGTATAGCATACATTATACGAAGTTATAGATCTAATTAACCCTCACTAAAGGGAA
J00180:83:H22V2BBXY:8:1222:15615:10053	1201	trim24_vector	10501	45	13S62M	1	154057290	ATCGAGGGACCTAATAACTTCGTATAGCATACATTATACGAAGTTATAGATCTAATTAACCCTCACTAAAGGGAA
J00180:83:H22V2BBXY:8:1222:16082:10194	1201	trim24_vector	10501	45	13S62M	1	154057290	ATCGAGGGACCTAATAACTTCGTATAGCATACATTATACGAAGTTATAGATCTAATTAACCCTCACTAAAGGGAA
J00180:83:H22V2BBXY:8:1111:25053:20287	177	trim24_vector	11171	0	18S57M	9	3019927	ACGAAATCACTGAAAAACGTGAAGACAATGCAGAAGCTAATGGGTTTTGTGTAGAGTGTGTTGAATGGCTCTGCA
J00180:83:H22V2BBXY:8:1103:19624:20621	417	trim24_vector	11256	0	38H37M	6	37915172	GAAGTTCACAAAAGACCACACAGTCAGGCAGAAAGAA
J00180:83:H22V2BBXY:8:2116:5467:41528	177	trim24_vector	11280	60	29S46M	2	26524787	AGAGGAGAGGTGTGTGCCCAGAAGACAGGCAGGCAGAAAGAAGAAGTATCTCCAGAGGCAGTTGGGGTGACCAGT
J00180:83:H22V2BBXY:8:1103:19624:20621	177	trim24_vector	11346	0	33S42M	6	37915172	TTCTTTCTGCCTGACTGTGTGGTCTTTTGTGAACTTCCATAAAAAGGAGCAGTTGAAACTTTACTGTGAAACATG
J00180:83:H22V2BBXY:8:2219:15483:30151	353	trim24_vector	11392	0	39H36M	6	37915309	AAACTGACCTGTCGAGACTGCCAGCTGCTAGAACAC
J00180:83:H22V2BBXY:8:2107:4046:19162	97	trim24_vector	11454	0	75M	12	118500706	AGAAGCTTTTCAGAATCAAAAAGTGATCATAGATACTCTAATCACCAAACTGATGGAAAAAACAAAATATATAAA
J00180:83:H22V2BBXY:8:2218:24292:24665	177	trim24_vector	11509	60	75M	14	16689068	GAAAAAACAAAATATATAAAGTATACAGGAAATCAGATCCAAAATAAGATAATTGAAATAAATCAAAACCAAAAG
J00180:83:H22V2BBXY:8:2103:3447:21078	1169	trim24_vector	11530	60	30S45M	15	52936244	TAAAAGGAACAAGCAAGAATCATATAATAATATACAGGGAATCAGATCCAAAATAGGATAATTGAAATAAATCAA
J00180:83:H22V2BBXY:8:2103:4553:14414	145	trim24_vector	11530	60	30S45M	15	52936244	TAAAAGGAACAAGCAAGAATCATATAATAATATACAGGGAATCAGATCCAAAATAGGATAATTGAAATAAATCAA
J00180:83:H22V2BBXY:8:2222:25814:29624	113	trim24_vector	11538	47	75M	6	121915259	AAATCAGATCCAAAATAGGATAATTGAAATAAATCAAAACCAAAAGCAGGTGGAACAGGATATTAAAGTTGCCAT
J00180:83:H22V2BBXY:8:1105:20811:38433	81	trim24_vector	11579	0	75M	12	61252405	AAAAGCAGGTGGAACAGGATATTAAAGTTGCCATCTTCACATTGATGGTGGAGATAAACAAAAAAGGGAAAGCTC
J00180:83:H22V2BBXY:8:2113:12804:2475	145	trim24_vector	11699	0	23S52M	1	109580470	AAAACAAAAGGAGTTGATTAAATTCATGCAACAACAGCAGGAAGTGGCTGGGCTTTCTAAGCAGTTAGAGCACGT
J00180:83:H22V2BBXY:8:1103:14194:48878	1105	trim24_vector	11761	52	75M	12	38622091	TCTAAATGGGCTGTTTCCAGTGGCAGCAGCACAGCCTTGCTGTACAGCAAGCGGCTGATTACATACAGGTTACGG
J00180:83:H22V2BBXY:8:1104:13119:1543	1105	trim24_vector	11761	52	75M	12	38622091	TCTAAATGGGCTGTTTCCAGTGGCAGCAGCACAGCCTTGCTGTACAGCAAGCGGCTGATTACATACAGGTTACGG
J00180:83:H22V2BBXY:8:2104:9841:3987	81	trim24_vector	11761	52	75M	12	38622091	TCTAAATGGGCTGTTTCCAGTGGCAGCAGCACAGCCTTGCTGTACAGCAAGCGGCTGATTACATACAGGTTACGG
J00180:83:H22V2BBXY:8:1124:25763:19408	65	trim24_vector	11783	60	75M	16	88039581	GCAGCAGCACAGCCTTGCTGTACAGCAAGCGGCTGATTACATACAGGTTACGGCACCTTCTTCGTGCAAGGTGTG
J00180:83:H22V2BBXY:8:1124:26200:19707	1089	trim24_vector	11783	60	75M	16	88039581	GCAGCAGCACAGCCTTGCTGTACAGCAAGCGGCTGATTACATACAGGTTACGGCACCTTCTTCGTGCAAGGTGTG
J00180:83:H22V2BBXY:8:1225:32522:17509	81	trim24_vector	11991	0	68M7S	6	37945170	TGTCGTGGAGCAGAGTTCACAGCCACCAGGTGGTTTACCTTCCAACCAGTTATCCAAGTTCCCAACACGCAACCN
J00180:83:H22V2BBXY:8:2213:2808:30450	81	trim24_vector	11991	0	75M	2	102117011	TGTCGTGGAGCAGAGTTCACAGCCACCAGGTGGTTTACCTTCCAACCAGTTATCCAAGTTCCCAACACAGATCAG
J00180:83:H22V2BBXY:8:2228:7608:37783	65	trim24_vector	12455	0	75M	6	37951525	TCACAAGTGCAGCTGGGTACGATGGAAAAGCTTTTAGTTCACCCATGATTGATCTGAGTGCACCGGTGGGAGGGT
J00180:83:H22V2BBXY:8:2218:21856:38521	81	trim24_vector	12548	23	75M	6	53731021	TTCCAGATATTGATTGTTCAAGTACTATAATGTTGGACAACATTGCAAGGAAAGACACAGGTGTAGATCACGCCC
J00180:83:H22V2BBXY:8:2218:24819:39154	177	trim24_vector	12846	0	24S51M	6	37957061	TTTGCTGGGGCCAATTCTATTCATACAGGAAAACAGTGGACCACCTGAAAATTATGATTTTCCTGTTGTTATAGT
J00180:83:H22V2BBXY:8:1104:14712:6730	161	trim24_vector	13049	0	75M	6	37957722	GACTCCATCAAGAAAATTCCTCAAATGGAAAGTCTGAGTGGTCGGATGCCTCCCAGAAGTCCCCTGTGCATGTCG
J00180:83:H22V2BBXY:8:2118:9820:16049	113	trim24_vector	13123	0	7S68M	5	117337551	AAGAGGAGGAGAGACGAGGAAGGAGGATGACCCCAATGAAGACTGGTGTGCTGTTTGTCAAAATGGTGGGGAACT
J00180:83:H22V2BBXY:8:1104:6370:16717	177	trim24_vector	13149	0	75M	6	95682727	CAATGAAGACTGGTGTGCTGTTTGTCAAAATGGTGGGGAACTCCTATGCTGTGAGAAATGTCCTAAAGTATTCCA
J00180:83:H22V2BBXY:8:2220:29406:27338	161	trim24_vector	13273	0	75M	15	16219793	ATCTGTACTTTCTGCCGAGACTTATCTAAGCCAGAGGTTGACTATGATTGTGATGTTCCCAGTCACCACTCAGAG

J00180:83:H22V2BBXY:8:1110:3386:18757	65	trim24_vector	13401	6	75M	5	142170463	TGAACGCCTACTTCTGTTTCTTTACTGCCATGAAATGAGCCTGGCTTTCCAAGACCCTGTTCCTCTAACTGTGCC
J00180:83:H22V2BBXY:8:2207:22638:14871	65	trim24_vector	13557	0	58M17S	8	29103654	AAAGCCTGAAGACTTTGTAGCTGATTTTAGATTGATCTTTCAAAACTGTGCTGAATTCTTGGTCATAGTATCATT
J00180:83:H22V2BBXY:8:1213:10500:19654	81	trim24_vector	13849	60	75M	6	37965626	GATTATAAAGATCATGATATCGATTACAAGGATGACGATGACAAGTAAAAGGGTGGGCGCGCCGACCCAGCTTTC
J00180:83:H22V2BBXY:8:2217:26078:28745	113	trim24_vector	13908	60	75M	19	18201969	CGCCGACCCAGCTTTCTTGTACAAAGTGGTTGATGGGGGATCCACTAGTTCTAGAGCGGCCGCTCTAGTGGATCC
J00180:83:H22V2BBXY:8:2224:11363:26529	113	trim24_vector	14040	60	75M	10	83043536	TCTCCTGGCCCTGGAAGTTGCCACTCCAGTGCCCACCAGCCTTGTCCTAATAAAATTAAGTTGCATCATTTTGTC
J00180:83:H22V2BBXY:8:1118:5731:31540	113	trim24_vector	14055	60	4S71M	11	115062867	TCTCAGTTGCCACTCCAGTGCCCACCAGCCTTGTCCTAATAAAATTAAGTTGCATCATTTTGTCTGACTAGGTGT
J00180:83:H22V2BBXY:8:2102:18345:26072	65	trim24_vector	14133	60	53M22S	1	172023718	TAATATTATGGGGTGGAGGGGGGTGGTATGGAGCAAGGGGCAAGTTGGGAAGAGTATGTTCCTCAGCATCTGACA
J00180:83:H22V2BBXY:8:2102:12936:11143	81	trim24_vector	14163	60	4S71M	X	23931629	ATATGAGCAAGGGGCAAGTTGGGAAGACAACCTGTAGGGCCTGCGGGGTCTATTGGGAACCAAGCTGGAGTGCAG
J00180:83:H22V2BBXY:8:2214:23043:24190	1153	trim24_vector	14171	60	75M	15	61540052	GGCAAGTTGGGAAGACAACCTGTAGGGCCTGCGGGGTCTATTGGGAACCAAGCTGGAGTGCAGTGGCACAATCTT
J00180:83:H22V2BBXY:8:2214:23094:24067	129	trim24_vector	14171	60	75M	15	61540052	GGCAAGTTGGGAAGACAACCTGTAGGGCCTGCGGGGTCTATTGGGAACCAAGCTGGAGTGCAGTGGCACAATCTT
J00180:83:H22V2BBXY:8:1228:5284:13886	81	trim24_vector	14179	60	40S35M	10	66798547	TGGAAGTCATTAAGAGCCTCTTAACATGAGGCCAGGAATCGGGAAGACAACCTGTAGGGCCTGCGGGGTCTATTG
J00180:83:H22V2BBXY:8:1111:9171:40561	97	trim24_vector	14247	60	75M	4	148883451	GCTCACTGCAATCTCCGCCTCCTGGGTTCAAGCGATTCTCCTGCCTCAGCCTCCCGAGTTGTTGGGATTCCAGGC
J00180:83:H22V2BBXY:8:1112:10226:11618	113	trim24_vector	14286	0	21S54M	13	28516230	GGCCTCAAACTCAGAAATCCGCCTGCCTCTGCCTCCCGAGTTGTTGGGATTCCAGGCATGCATGACCAGGCTCAA
J00180:83:H22V2BBXY:8:2123:25997:4163	65	trim24_vector	14543	60	75M	17	75824074	GCCATGCCCAACCGGTGGGACATTTGAGTTGCTTGCTTGGCACTGTCCTCTCATGCGTTGGGTCCACTCAGTAGA
J00180:83:H22V2BBXY:8:2209:17919:3459	145	trim24_vector	14547	60	75M	17	5140667	TGCCCAACCGGTGGGACATTTGAGTTGCTTGCTTGGCACTGTCCTCTCATGCGTTGAGTCCACTCAGTAGATGCC
J00180:83:H22V2BBXY:8:1223:22607:19988	145	trim24_vector	14601	60	3S33M39S	1	122275171	GTGTGGGTCCACTCAGTAGATGCCTGTTGAATTCGATATCAAGCTTATCGATAAGCTTATATTCCATGCTAGGGT
J00180:83:H22V2BBXY:8:1223:22719:19935	1169	trim24_vector	14601	60	3S33M39S	1	122275171	GTGTGGGTCCACTCAGTAGATGCCTGTTGAATTCGATATCAAGCTTATCGATAAGCTTATATTCCATGCTAGGGT
J00180:83:H22V2BBXY:8:1208:8501:23329	145	trim24_vector	14680	0	75M	12	3109884	AGAGATAATGGCCTTACGTTGTGCCAGGGGAGGGGCGGGCTGGACTCAGCAAGACTTACCTTCTCCAAAGAGCGG
J00180:83:H22V2BBXY:8:1121:11657:32314	161	trim24_vector	14811	0	53M22S	8	128312809	TGAAGAACTGTGGGGATGTGGCACTGAGGGACATGGCCAGTGGGCACGGTGGGAGGGAGGGAGGAAGGAAGGAAG
J00180:83:H22V2BBXY:8:2225:27499:12321	177	trim24_vector	14905	0	75M	8	126607545	GCCTTCATGATTTGACGATTGTATGAACATCTACATGGCAATTCTCCAGCTGCCTGTCCCAGTCCTACTGACCCA
J00180:83:H22V2BBXY:8:1203:19218:28745	65	trim24_vector	14936	0	56M19S	6	48350261	TACATGGCAATTCTCCAGCTGCCTGTCCCAGTCCTACTGACCCAGCTGTATCTCTCATATTTCAGTGACCATGGT
J00180:83:H22V2BBXY:8:2110:18791:39717	161	trim24_vector	15019	0	75M	7	139845108	TGCATCCAGACACCATCAAACATGCAGGCTCAGACACAGGGACCAGCAGTGTCTGTGGCCTTTTTGTGCTCCTCT
J00180:83:H22V2BBXY:8:1218:25408:40315	113	trim24_vector	15221	0	19S56M	6	28231845	TCTTTTAACCAACCCTGTTTCCTGCACTTCCTTATCCCCAAGATCTGCCTCTCCGTGATGCACTGAATTGGCAAA
J00180:83:H22V2BBXY:8:1106:7710:43515	177	trim24_vector	15369	0	75M	6	123563684	TTGGATATGAGGAGGCAGTTTTTCCCCCAGAGGGTGGTGACGCACTGAACAGGTTGCCCAAGGAGGCTGTGGATG
J00180:83:H22V2BBXY:8:2103:4087:25316	1105	trim24_vector	15884	60	75M	2	5954067	CCCGGGCTGCAGGAATTCGATAAAAGTTTTGTTACTTTATAGAAGAAATTTTGAGTTTTTGTTTTTTTTAATAAA
J00180:83:H22V2BBXY:8:2103:4198:23997	81	trim24_vector	15884	60	75M	2	5954067	CCCGGGCTGCAGGAATTCGATAAAAGTTTTGTTACTTTATAGAAGAAATTTTGAGTTTTTGTTTTTTTTAATAAA

ReadID	Flag	Vector	Start	MapQ	CIGAR	Chr	Start2	Seq
J00180:83:H22V2BBXY:8:2228:18933:28674	81	trim24_vector	2738	60	75M	1	182070041	ATCTCAGTCGCCGCTTGGAGCTCCCGTGAGGCGTGCTTGTCAATGCGGTAAGTGTCACTGATTTTGAACTATAAC
J00180:83:H22V2BBXY:8:2119:2483:25914	113	trim24_vector	4709	60	15S60M	1	85109564	GGTTTCTGGGGACTTGGGCTGTAGTCTGGGTCCGCGAGCAAGGTTGCCCTGAACTGGGGGTTGGGGGGAGCGCAG
J00180:83:H22V2BBXY:8:2122:26798:43040	113	trim24_vector	5183	60	75M	1	41153484	TTCTTAAGTAGCTGAAGCTCCGGTTTTGAACTATGCGCTCGGGGTTGGCGAGTGTGTTTTGTGAAGTTTTTTAGG
J00180:83:H22V2BBXY:8:1213:5954:36218	129	trim24_vector	11332	0	75M	1	173235504	CCAGTGTTTTGTCCCTTCCATAAAAAGGAGCAGTTGAAACTTTACTGTGAAACATGTGATAAACTGACCTGTCGA
J00180:83:H22V2BBXY:8:1118:29751:34055	145	trim24_vector	11634	27	33S42M	1	133019799	CAGGGCTACACAGAGAAACCCTGGCTCGGAAAAAAACAAAAAAGGGAAAGCTCTGCTGCACCAGCTTGAGAGTCT
J00180:83:H22V2BBXY:8:1127:3731:15557	1121	trim24_vector	13274	0	54M21S	1	137636167	TCTGTACTTTCTGCCGAGACTTATCTAAGCCAGAGGTTGACTATGATTGTGATGGGTAATCCTATTAGTTTGGAA
J00180:83:H22V2BBXY:8:1217:23378:1736	161	trim24_vector	13274	0	54M21S	1	137636167	TCTGTACTTTCTGCCGAGACTTATCTAAGCCAGAGGTTGACTATGATTGTGATGGGTAATCCTATTAGTTTGGAA
J00180:83:H22V2BBXY:8:1127:1235:27795	65	trim24_vector	13593	60	57M18S	1	169099437	CTTTCAAAACTGTGCTGAATTCAATGAGCCTGATTCTGAAGTAGCCAATGCTGGTATCTAACTCGCTAGGCTTCT
J00180:83:H22V2BBXY:8:2101:6685:17966	65	trim24_vector	13982	60	75M	1	34593441	CCCCGGGCTGCAGCCCGGGGGATCTGGGGTGGCATCCCTGTGACCCCTCCCCAGTGCCTCTCCTGGCCCTGGAAG
J00180:83:H22V2BBXY:8:1119:5700:29729	145	trim24_vector	14141	60	13S62M	1	46445954	CCTGGGCTTTTTTTGGGGTGGAGGGGGGGTGGTATGGAGCAAGGGGCAAGTTGGGAAGACAACCTGTAGGGCCTGC
J00180:83:H22V2BBXY:8:1218:7283:40139	161	trim24_vector	14155	60	75M	1	74201579	GTGGTATGGAGCAAGGGGCAAGTTGGGAAGACAACCTGTAGGGCCTGCGGGGTCTATTGGGAACCAAGCTGGAGT
J00180:83:H22V2BBXY:8:2218:10287:20586	161	trim24_vector	16070	60	75M	1	182070175	ATAAAACACATGCATCAATTTTACACATGATTATCTTTAACGTACGTCACAATATGATTATCTTTCTAGGGTTAA
J00180:83:H22V2BBXY:8:1226:6360:44095	145	trim24_vector	3579	0	16S59M	2	49418985	TTGAGTTCCAGTCCTGACTTCCTTATCCCCAAGATCTGCCTCTCCGTGATGCACTGAATTGGCAAACATGTGTCA
J00180:83:H22V2BBXY:8:1214:20009:18159	81	trim24_vector	13978	9	45S30M	2	155050907	ACCTAGTGGAAGCTAAAAAGCCGAATTCTAGAGGTACCGGTTGTTGATCCCCCGGGCTGCAGCCCGGGGGATCTG
J00180:83:H22V2BBXY:8:2116:10338:4180	161	trim24_vector	14164	60	47M28S	2	142999523	AGCAAGGGGCAAGTTGGGAAGACAACCTGTAGGGCCTGCGGGGTCTAAGGTTTTAATAAGTGTTAAACAAATATG
J00180:83:H22V2BBXY:8:2116:11688:3846	1185	trim24_vector	14164	60	47M28S	2	142999523	AGCAAGGGGCAAGTTGGGAAGACAACCTGTAGGGCCTGCGGGGTCTAAGGTTTTAATAAGTGTTAAACAAATATG
J00180:83:H22V2BBXY:8:2227:30919:44060	129	trim24_vector	14390	60	47M28S	2	128119787	TGGTCTCCAACTCCTAATCTCAGGTGATCTACCCACCTTGGCCTCCCCACAGCCCAGTACTGTTGTGCCCACTTG
J00180:83:H22V2BBXY:8:2106:21227:39471	65	trim24_vector	14425	60	68M7S	2	37319739	CCTTGGCCTCCCAAATTGCTGGGATTACAGGCGTGAACCACTGCTTCCTTCCCTGTCCTTCTGATTTTCTTGTAT
J00180:83:H22V2BBXY:8:1209:12662:36552	129	trim24_vector	14455	60	57M18S	2	70255817	GCGTGAACCACTGCTCCCTTCCCTGTCCTTCTGATTTTAAAATAACTATACCAGCAGATTTGGTCCAAACACATC
J00180:83:H22V2BBXY:8:2108:10318:14484	145	trim24_vector	3116	0	75M	3	132262919	GGCACAGCTGCCCACGGAGGTGGGGGGGTCACCGTCCCTGGAGGTGATGAAGAACTGTGGGGATGTGGCACTGAG
J00180:83:H22V2BBXY:8:2109:22465:47665	145	trim24_vector	3481	0	75M	3	60168935	TCCTATCTTCTTATCCTTAAGGCTGTTCTGAACGCTGTGACTTGGAGAGTGTCCCAGAGCCCTCAACACCTGCAT
J00180:83:H22V2BBXY:8:1123:19999:22502	129	trim24_vector	12328	58	75M	3	152755808	ATAAAGCCCAACTCCTTGCAAATGGCTTTTTTGGCTCAACAGGCCATAAAACAGTGGCAGATCAGCAGTGTACAG
J00180:83:H22V2BBXY:8:1227:14935:29694	129	trim24_vector	14150	60	75M	3	103404085	GGGGGGTGGTATGGAGCAAGGGGCAAGTTGGGAAGACAACCTGTAGGGCCTGCGGGGTCTATTGGGAACCAAGCT
J00180:83:H22V2BBXY:8:2204:1844:5464	145	trim24_vector	14391	60	75M	3	133449106	GGTCTCCAACTCCTAATCTCAGGTGATCTACCCACCTTGGCCTCCCAAATTGCTGGGATTACAGACGTGAACCAC
J00180:83:H22V2BBXY:8:2124:22435:39418	97	trim24_vector	14468	60	61M14S	3	148236135	CTCCCTTCCCTGTCCTTCTGATTTTAAAATAACTATACCAGCAGGAGGACGTCCAGACACAAGCACAGGTTTTAA
J00180:83:H22V2BBXY:8:2224:24637:2650	81	trim24_vector	15006	0	26S49M	3	19230214	GTGGAAGAATTCCTCCGTTACTTTTTCACCCCTTCTGCTTGCATCCAGACACCATCAAACATGCAGGCTCAGACA
J00180:83:H22V2BBXY:8:1205:14083:19249	161	trim24_vector	13502	0	75M	4	31524170	CAATGGACTTGTCAACCATCAAGAAAAGACTTCAGGAGGATTATTGCATGTATACAAAGCCTGAAGACTTTGTAG
J00180:83:H22V2BBXY:8:2105:1732:12093	129	trim24_vector	14264	60	75M	4	155776224	CCTCCTGGGTTCAAGCGATTCTCCTGCCTCAGCCTCCCGAGTTGTTGGGATTCCAGGCATGCATGACCAGGCTCA
J00180:83:H22V2BBXY:8:2106:13098:29747	129	trim24_vector	14278	60	75M	4	5316364	GCGATTCTCCTGCCTCAGCCTCCCGAGTTGTTGGGATTCCAGGCATGCATGACCAGGCTCAGCTAATTTTTGTTT
J00180:83:H22V2BBXY:8:1206:16244:17896	81	trim24_vector	14326	60	75M	4	137157517	ATGACCAGGCTCAGCTAATTTTTGTTTTTTTGGTAGAGACGGGGTTTCACCATATTGGCCAGGCTGGTCTCCAAC
J00180:83:H22V2BBXY:8:2109:28341:16946	161	trim24_vector	14387	60	75M	4	58884014	GGCTGGTCTCCAACTCCTAATCTCAGGTGATCTACCCACCTTGGCCTCCCAAATTGCTGGGATTACAGGCGTGAA
J00180:83:H22V2BBXY:8:1128:28442:27672	81	trim24_vector	14460	60	30S45M	4	40684322	GCCTGTGGAGTTAGTCCAGATTTGAATCATAACCACTGCTCCCTTCCCTGTCCTTCTGATTTTAAAATAACTATA
J00180:83:H22V2BBXY:8:2202:27854:24331	177	trim24_vector	14465	60	75M	4	150193160	CTGCTCCCTTCCCTGTCCTTCTGATTTTAAAATAACTATACCAGCAGGAGGACGTCCAGACACAGCATAGGCTAC
J00180:83:H22V2BBXY:8:2202:27864:24278	1201	trim24_vector	14465	60	75M	4	150193160	CTGCTCCCTTCCCTGTCCTTCTGATTTTAAAATAACTATACCAGCAGGAGGACGTCCAGACACAGCATAGGCTAC
J00180:83:H22V2BBXY:8:2126:8988:45906	145	trim24_vector	11520	60	75M	5	140701312	ATATATAAAGTATACAGGAAATCAGATCCAAAATAGGATAATTGAAATAAATCAAAACCAAAAGCAGGTGGAACA
J00180:83:H22V2BBXY:8:1128:5416:13482	1153	trim24_vector	14069	60	75M	5	44402139	TGCCCACCAGCCTTGTCCTAATAAAATTAAGTTGCATCATTTTTTCTGACTTGGTGTCCTTCTATAATATTATGG
J00180:83:H22V2BBXY:8:1128:6766:17192	129	trim24_vector	14069	60	75M	5	44402139	TGCCCACCAGCCTTGTCCTAATAAAATTAAGTTGCATCATTTTGTCTGACTAGGTGTCCTTCTATAATATTATGG
J00180:83:H22V2BBXY:8:1211:15930:36059	97	trim24_vector	14344	60	50M25S	5	149971426	TTTTTGTTTTTTTGGTAGAGACGGGGTTTCACCATATTGGCCAGGCTGGTTTTTGTGCACAGACATTTTTCATTT
J00180:83:H22V2BBXY:8:1107:6786:29008	113	trim24_vector	14532	60	75M	5	102609117	TAGGCTACCTGGCCATGCCCAACCGGTGGGACATTTGAGTTGCTTGCTTGGCACTGTCCTCTCATGCGTTGGGTC
J00180:83:H22V2BBXY:8:1119:19603:7468	161	trim24_vector	14599	60	35M40S	5	72639067	GTTGGGTCCACTCAGTAGATGCCTGTTGAATTCGATATCAAGCTTATATTCCATGCTAGGGTTCTGGATGATGGT
J00180:83:H22V2BBXY:8:2127:22150:19795	145	trim24_vector	15566	0	19S56M	5	87788798	GCCCAGATTCACATTGAGACCAGGCCGTTCTATGATTCTGTCATTCTAAATCTCTCTTTCAGCCTAAAGCTTTTT
J00180:83:H22V2BBXY:8:2113:29112:35339	81	trim24_vector	11526	33	52M23S	6	37915218	AAAGTATACAGGAAATCAGATCCAAAATAGGATAATTGAAAAGAATCAAAACTTGGATAGAGACAATCATTCCAT
J00180:83:H22V2BBXY:8:1106:17868:43462	145	trim24_vector	11530	60	47M28S	6	37919394	TATACAGGAAATCAGATCCAAAATAGGATAATTGAAATAAATCAAAAGAAGAGAAAGATGGAGAAACAGGAAAAG
J00180:83:H22V2BBXY:8:2107:19735:21553	81	trim24_vector	11781	60	75M	6	37931362	TGGCAGCAGCACAGCCTTGCTGTACAGCAAGCGGCTGATTACATACAGGTTAAGGCACCTTCTTCGTGCAAGGTG
J00180:83:H22V2BBXY:8:1212:30482:12568	177	trim24_vector	12110	0	37S38M	6	37945658	CCCCCCCCACGCTAGGCCCTTTCCTAGCCCTTCGTGCCTCAGATGCAACAGGTGCAACGGAGGCCAGCACCTGTG
J00180:83:H22V2BBXY:8:2128:26169:39277	161	trim24_vector	12315	23	75M	6	84113763	TCGGCAGACAACAATAAAGCCCAACCCCTTGCAAATGGCTTTTTTGGCTCAACAGGCCATAAAACAGTGGCAGAT
J00180:83:H22V2BBXY:8:2107:1773:43251	353	trim24_vector	13151	0	43H32M	6	37957726	ATGAAGACTGGTGTGCTGTTTGTCAAAATGGT
J00180:83:H22V2BBXY:8:2109:24312:33000	161	trim24_vector	13470	3	32S43M	6	37964754	CCTTGTTACTTAGCCTCTCTGGGTCTGTAGATTGTGCCTGATTATTATAAAATAATTAAAAACCCAATGGACTTG
J00180:83:H22V2BBXY:8:1228:20943:42987	97	trim24_vector	14020	60	68M7S	6	125068723	TGTGACCCCTCCCCAGTGCCTCTCCTGGCCCTGGAAGTTGCCACTCCAGTGCCCACCAGCCTTGTCCTGCTAGTT
J00180:83:H22V2BBXY:8:2117:14681:3899	145	trim24_vector	14218	60	75M	6	132222160	CCAAGCTGGAGTGCAGTGGCACAATCTTGGCTCACTGCAATCTCCGCCTCCTGGGTTCAAGCGATTCTCCTGCCT
J00180:83:H22V2BBXY:8:1208:1955:19689	145	trim24_vector	14334	60	75M	6	119637417	GCTCAGCTAATTTTTGTTTTTTTGGTAGAGACGGGGTTTCACCATATTGGCCAGGCTGGTCTCCAACTCCTAATC
J00180:83:H22V2BBXY:8:1208:2087:19918	1169	trim24_vector	14334	60	75M	6	119637417	GCTCAGCTAATTTTTGTTTTTTTGGTAGAGACGGGGTTTCACCATATTGGCCAGGCTGGTCTCCAACTCCTAATC
J00180:83:H22V2BBXY:8:2207:2960:15346	97	trim24_vector	14366	60	75M	6	82480666	GGGGTTTCACCATATTGGCCAGGCTGGTCTCCAACTCCTAATCTCAGGTGATCTACCCACCTTGGCCTTCCAAAT

J00180:83:H22V2BBXY:8:2214:3539:17403	113	trim24_vector	15389	0	75M	6	4426030	TTTCCCCCAGAGGGTGGTGACGCACTGAACAGGTTGCCCAAGGAGGCTGTGGATGCCCCATCCCTGCAGGCATTC
J00180:83:H22V2BBXY:8:2105:7070:17016	1137	trim24_vector	14139	60	75M	7	127519486	TATGGGGTGGAGGGGGGTGGTATGGAGCAAGGGGCAAGTTGGGAAGACAACCTGTAGGGCCTGCGGGGTCTATTG
J00180:83:H22V2BBXY:8:2105:7436:16665	113	trim24_vector	14139	60	75M	7	127519486	TATGGGGTGGAGGGGGGTGGTATGGAGCAAGGGGCAAGTTGGGAAGACAACCTGTAGGGCCTGCGGGGTCTATTG
J00180:83:H22V2BBXY:8:2105:7902:17016	1137	trim24_vector	14139	60	75M	7	127519486	TATGGGGTGGAGGGGGGTGGTATGGAGCAAGGGGCAAGTTGGGAAGACAACCTGTAGGGCCTGCGGGGTCTATTG
J00180:83:H22V2BBXY:8:1119:6076:28692	177	trim24_vector	14141	60	13S62M	7	103109060	CCTGGGCTTTTTTTGGGGTGGGGGGGGGTGGTATGGAGCAAGGGGCAAGTTGGGAAGACAACCTGTAGGGCCTGC
J00180:83:H22V2BBXY:8:2217:12428:36429	65	trim24_vector	14332	60	54M21S	7	45378472	AGGCTCAGCTAATTTTTGTTTTTTTGGTAGAGACGGGGTTTCACCATATTGGCCTAGCTCATTGTTAGACTGTGA
J00180:83:H22V2BBXY:8:2217:12652:35479	1089	trim24_vector	14332	60	54M21S	7	45378472	AGGCTCAGCTAATTTTTGTTTTTTTGGTAGAGACGGGGTTTCACCATATTGGCCTAGCTCATTGTTAGACTGTGA
J00180:83:H22V2BBXY:8:1111:12246:19724	129	trim24_vector	14550	60	68M7S	7	64886209	CCAACCGGTGGGACATTTGAGTTGCTTGCTTGGCACTGTCCTCTCATGCGTTGGGTCCACTCAGTAGACTGTTGC
J00180:83:H22V2BBXY:8:1126:11363:28956	161	trim24_vector	14804	0	49M26S	7	52306132	GAGGTGATGAAGAACTGTGGGGATGTGGCACTGAGGGACATGGCCAGTGTTCAGGTGGGGTTGGGGGGAGGGGGC
J00180:83:H22V2BBXY:8:2108:11018:20691	113	trim24_vector	3787	0	75M	8	105568136	CTGTGGATGCCCCATCCCTGCAGGCATTCAAGGCCAGGCTGGATGTGGCTCTGGGCAGCCTGGGCTGCTGGTTGA
J00180:83:H22V2BBXY:8:1118:14001:11864	97	trim24_vector	4239	60	75M	8	105794146	GGTTTTGGCGCCTCCCGCGGGCGCCCCCCTCCTCACGGCGAGCGCTGCCACGTCAGACGAAGGGCGCAGCGAGCG
J00180:83:H22V2BBXY:8:1118:16731:11319	1121	trim24_vector	4239	60	75M	8	105794146	GGTTTTGGCGCCTCCCGCGGGCGCCCCCCTCCTCACGGCGAGCGCTGCCACGTCAGACGAAGGGCGCAGCGAGCG
J00180:83:H22V2BBXY:8:1223:22861:7240	81	trim24_vector	11805	33	75M	8	113421416	CAGCAAGCGGCTGATTACATACAGATTACGGCACCTTCTTCGTGCAAGGTGTGATGCTTCTCCTGTGACCAACAC
J00180:83:H22V2BBXY:8:1203:14529:49036	97	trim24_vector	14231	60	60M15S	8	12838153	NAGTGGCACAATCTTGGCTCACTGCAATCTCCGCCTCCTGGGTTCAAGCGATTCTCCTGCTGAGGCAGCTTCCAT
J00180:83:H22V2BBXY:8:2211:14742:15574	97	trim24_vector	14603	60	31M44S	8	84701308	GGTCCACTCAGTAGATGCCTGTTGAATTCGATATCAAGCTTATCGATAAGCTTATATTCCATGCTAGGGTTCTGG
J00180:83:H22V2BBXY:8:2209:12875:20674	113	trim24_vector	4466	60	75M	9	3024100	AAAAGTAGTCCCTTCTCGGCGATTCTGCGGAGGGATCTCCGTGGGGCGGTGAACGCCGATGATTATATAAGGACG
J00180:83:H22V2BBXY:8:2108:12428:35514	81	trim24_vector	13167	0	33S42M	9	99983112	GGTAGTAATGACAGCTGTATCCTAAGGATGCTTTGTTTGTCAAAATGGTGGGGAACTCCTATGCTGTGAGAAATG
J00180:83:H22V2BBXY:8:1115:30969:43902	161	trim24_vector	14036	60	75M	9	90829644	TGCCTCTCCTGGCCCTGGAAGTTGCCACTCCAGTGCCCACCAGCCTTGTCCTAATAAAATTAAGTTGCATCATTT
J00180:83:H22V2BBXY:8:2121:25256:9491	161	trim24_vector	14099	60	50M25S	9	83755445	GTTGCATCATTTTGTCTGACTAGGTGTCCTTCTATAATATTATGGGGTGGGGGGTAGAATTAGAGAGAGGCAGAT
J00180:83:H22V2BBXY:8:2204:13301:22151	65	trim24_vector	14169	60	47M28S	9	58213813	GGGGCAAGTTGGGAAGACAACCTGTAGGGCCTGCGGGGTCTATTGGGGAAGGGACTAGGCTGAGGGACTCTCCTC
J00180:83:H22V2BBXY:8:1109:6664:20357	129	trim24_vector	14256	60	71M4S	9	93887241	AATCTCCGCCTCCTGGGTTCAAGCGATTCTCCTGCCTCAGCCTCCCGAGTTGTTGGGATACCAGGCATGCAATTG
J00180:83:H22V2BBXY:8:1218:7436:41000	97	trim24_vector	14392	60	41M34S	9	34753385	GTCTCCAACTCCTAATCTCAGGTGATCTACCCACCTTGGCCCTCCATGCTGCTGTTTTTATTTTAAAGTTTCGAA
J00180:83:H22V2BBXY:8:2114:5061:6325	177	trim24_vector	14405	60	75M	9	48106860	AATCTCAGGTGATCTACCCACCTTGGCCTCCCAAATTGCTGGGATTACAGGCGTGAACCACTGCTCCCTTCCCTG
J00180:83:H22V2BBXY:8:2216:25002:9684	97	trim24_vector	14467	60	75M	9	21402835	GCTCCCTTCCCTGTCCTTCTGATTTTAAAATAACTATACCAGCAGGAGGACGTCCAGACACAGCATAGGCTACCT
J00180:83:H22V2BBXY:8:2209:30279:47700	113	trim24_vector	14468	60	31S44M	9	49222118	CCCATTCTCCTTCCTAACTTCCTCCCCTACTCTCCCTTCCCTGTCCTTCTGATTTTAAAATAACTATACCAGCAG
J00180:83:H22V2BBXY:8:2209:30634:47436	1137	trim24_vector	14468	60	31S44M	9	49222118	CCCATTCTCCTTCCTAACTTCCTCCCCTACTCTCCCTTCCCTGTCCTTCTGATTTTAAAATAACTATACCAGCAG
J00180:83:H22V2BBXY:8:2213:16376:29237	1121	trim24_vector	14607	48	27M48S	9	8000039	CACTCAGTAGATGCCTGTTGAATTCGATATCAAGCTTATATTCCATGCTAGGGTTCTGGTATTGGTGCGTGGGGT
J00180:83:H22V2BBXY:8:2213:16386:27637	97	trim24_vector	14607	48	27M48S	9	8000039	CACTCAGTAGATGCCTGTTGAATTCGATATCAAGCTTATATTCCATGCTAGGGTTCTGGTGTTGGTGCGTGGGGT
J00180:83:H22V2BBXY:8:1221:2828:36394	161	trim24_vector	3149	0	75M	10	61613839	GTCCCTGGAGGTGATGAAGAACTGTGGGGATGTGGCACTGAGGGACATGGCCAGTGGGCACGGTGGGTGGGTTGG
J00180:83:H22V2BBXY:8:2205:19918:35163	177	trim24_vector	12267	0	75M	10	117663921	TGCTCCTTATCCTCAGCAGCTGAGATATTCACCAAGCCAGAATGTACCTCGGCAGACAACAATAAAGCCCAACCC
J00180:83:H22V2BBXY:8:1127:10399:18212	1153	trim24_vector	14020	60	75M	10	12767587	TCTGACCCCTCCCCAGTGCCTCTCCTGGCCCTGGAAGTTGCCACTCCAGTGCCCACCAGCCTTGTCCTAATAAAA
J00180:83:H22V2BBXY:8:1127:9546:15117	129	trim24_vector	14020	60	75M	10	12767587	TGTGACCCCTCCCCAGTGCCTCTCCTGGCCCTGGAAGTTGCCACTCCAGTGCCCACCAGCCTTGTCCTAATAAAA
J00180:83:H22V2BBXY:8:2215:17980:21219	129	trim24_vector	14066	60	43M32S	10	3828486	CAGTGCCCACCAGCCTTGTCCTAATAAAATTAAGTTGCATCATGATTTGTTTGATGATGTTTCCTTATTTTATGG
J00180:83:H22V2BBXY:8:1105:20192:31101	161	trim24_vector	14140	60	75M	10	79740271	ATGGGGTGGAGGGGGGTGGTATGGAGCAAGGGGCAAGTTGGGAAGACAACCTGTAGGGCCTGCGGGGTCTATTGG
J00180:83:H22V2BBXY:8:1119:6086:28674	177	trim24_vector	14141	60	13S62M	10	104231877	CCTGGGCTTTTTTTGGGGTGGAGGGGGGTGGTATGGAGCAAGGGGCAAGTTGGGAAGACAACCTGTAGGGCCTGC
J00180:83:H22V2BBXY:8:1216:9567:34565	113	trim24_vector	14168	60	75M	10	126238233	ATGGGCAAGTTGGGAAGACAACCTGTAGGGCCTGCGGGGTCTATTGGGAACCAAGCTGGAGTGCAGTGGCACAAT
J00180:83:H22V2BBXY:8:2221:29924:5974	177	trim24_vector	14262	60	75M	10	77367719	TGCCTCCTGGGTTCAAGCGATTCTCCTGCCTCAGCCTCCCGAGTTGTTGGGATTCCAGGCATGCATGACCAGGCT
J00180:83:H22V2BBXY:8:1104:29640:37800	65	trim24_vector	14308	60	75M	10	128694540	TTGGGATTCCAGGCATGCATGACCAGGCTCAGCTAATTTTTGTTTTTTTGGTAGAGACGGGGTTTCACCATATTG
J00180:83:H22V2BBXY:8:2112:4208:23276	177	trim24_vector	14361	60	14S61M	10	76101364	GTGCCAATCTGGTGGAGACGGGGTTTCACCATATTGGCCAGGCTGGTCTCCAACTCCTAATCTCAGGTGATCTAC
J00180:83:H22V2BBXY:8:1203:29295:18001	161	trim24_vector	14604	60	30M45S	10	127044882	GTCCACTCAGTAGATGCCTGTTGAATTCGATATCAAGCTTATCGATAAGCTTATATTCCATGCTAGGGTTCTGGT
J00180:83:H22V2BBXY:8:2127:2301:33404	113	trim24_vector	14144	60	8S67M	11	45504724	TCAGGCTTGGTGGAGGGGGGTGGTATGGAGCAAGGGGCAAGTTGGGAAGACAACCTGTAGGGCCTGCGGGGTCTA
J00180:83:H22V2BBXY:8:1111:1651:7486	161	trim24_vector	14196	60	75M	11	85009402	GGCCTTCGGGGTCTATTGGGAACCAAGCTGGAGTGCAGTGGCACAATCTTGGCTCACTGCAATCTCCGCCTCCTG
J00180:83:H22V2BBXY:8:1128:3356:47190	129	trim24_vector	14271	60	58M17S	11	96189951	GGTTCAAGCGATTCTCCTGCCTCAGCCTCCCGAGTTGTTGGGATTCCAGGCATGCATGCGCCCGGCTGAACATTG
J00180:83:H22V2BBXY:8:2209:12327:47155	145	trim24_vector	14348	60	75M	11	104683371	TGTTTTTTTGGTAGAGACGGGGTTTCACCATATTGGCCAGGCTGGTCTCCAACTCCTAATCTCAGGTGATCTACC
J00180:83:H22V2BBXY:8:2209:12479:46680	1169	trim24_vector	14348	60	75M	11	104683371	TGTTTTTTTGGTAGAGACGGGGTTTCACCATATTGGCCAGGCTGGTCTCCAACTCCTAATCTCAGGTGATCTACC
J00180:83:H22V2BBXY:8:2209:15869:44359	1169	trim24_vector	14348	60	75M	11	104683371	TGTTTTTTTGGTAGAGACGGGGTTTCACCATATTGGCCAGGCTGGTCTCCAACTCCTAATCTCAGGTGATCTACC
J00180:83:H22V2BBXY:8:1204:29082:14502	97	trim24_vector	14375	60	75M	11	109134366	CCATATTGGCCAGGCTGGTCTCCAACTCCTAATCTCAGGTGATCTACCCACCTTGGCCTCCCAAATTGCTGGGAT
J00180:83:H22V2BBXY:8:1116:11718:6818	161	trim24_vector	14511	60	75M	11	39311631	GGAGGACGTCCAGACACAGCATAGGCTACCTGGCCATGCCCAACCGGTGGGACATTTGAGTTGCTTGCTTGGCAC
J00180:83:H22V2BBXY:8:2219:21866:11425	129	trim24_vector	14515	60	75M	11	55921424	GACGTCCAGACACAGCATAGGCTACCTGGCCATGCCCAACCGGTGGGACATTTGAGTTGCTTGCTTGGCACTGTC
J00180:83:H22V2BBXY:8:1227:24931:2211	113	trim24_vector	14542	60	75M	11	8538434	GGCCATGCCCAACCGGTGGGACATTTGAGTTGCTTGCTTGGCACTGTCCTCTCATGCGTTGGGTCCACTCAGTAG
J00180:83:H22V2BBXY:8:1107:1692:21061	97	trim24_vector	14561	60	73M2S	11	32089265	GACATTTGAGTTGCTTGCTTGGTACTGTCCTCTCATGCGTTGGGTCCACTCAGTAGATGCCTGTTGAATTCGATA
J00180:83:H22V2BBXY:8:2128:13514:1525	97	trim24_vector	3214	0	41M34S	12	42596322	GGTGGGTTGGGGTTGGTCTTGGGGATCTTGGAGGGCTTTTCTCAAAGGACCAGCTCCTTGATTGTTAGATTCTTT
J00180:83:H22V2BBXY:8:1117:19989:12321	113	trim24_vector	14426	60	75M	12	22209179	CTTGGCCTCCCAAATTGCTGGGATTACAGGCGTGAACCACTGCTCCCTTCCCTGTCCTTCTGATTTTAAAATAAC
J00180:83:H22V2BBXY:8:1210:6827:17298	81	trim24_vector	14951	0	75M	12	119597719	CAGCTGCCTGTCCCAGTCCTACTGACCCAGCTGTATCTCTCCAGGCAAGCTCTTCCACCCCTTCTGCTTGCATCC

J00180:83:H22V2BBXY:8:1222:27072:23012	113	trim24_vector	13168	0	75M	13	106148682	GTTTGTCAAAATGGTGGGGAACTCCTATGCTGTGAGAAATGTCCTAAAGTATTCCATCTTACTTGTCATGTGCCC
J00180:83:H22V2BBXY:8:2214:2301:23839	145	trim24_vector	13425	0	28S47M	13	47799246	TTCTTTTCATTATGCTAACACTTCTTACCTGCCATGAAATGAGCCTGGCTTTCCAAGACCCTGTTCCTCTAACTG
J00180:83:H22V2BBXY:8:2209:7588:45344	129	trim24_vector	14112	60	41M34S	13	53883918	GTCTGACTAGGTGTCCTTCTATAATATTATGGGGTGGAGGGAGGAGTGGGGAAGGGAAGAGTGGGGAGGGGAGGA
J00180:83:H22V2BBXY:8:1113:24353:20058	1121	trim24_vector	14198	60	75M	13	20481200	CCTGCGGGGTCTATTGGGAACCAAGCTGGAGTGCAGTGGCACAATCTTGGCTCACTGCAATCTCCGCCTCCTGGG
J00180:83:H22V2BBXY:8:1113:24464:19478	97	trim24_vector	14198	60	75M	13	20481200	CCTGCGGGGTCTATTGGGAACCAAGCTGGAGTGCAGTGGCACAATCTTGGCTCACTGCAATCTCCGCCTCCTGGG
J00180:83:H22V2BBXY:8:2224:1986:21922	81	trim24_vector	14278	60	22S53M	13	73148351	ATGCTGAACAGTCTGCCTCTCTGCGATTCTCCTGCCTCAGCCTCCCGAGTTGTTGGGATTCCAGGCATGCATGAC
J00180:83:H22V2BBXY:8:1207:25357:46803	113	trim24_vector	14336	60	27S48M	13	28291057	GCATCCACCTAGGAAATACCAATATAATCAGCTAATTTTTGTTTTTTTGGTAGAGACGGGGTTTCACCATATTGG
J00180:83:H22V2BBXY:8:2204:1773:5728	145	trim24_vector	14391	60	75M	13	10925779	GGTCTCCAACTCCTAATCTCAGGTGATCTACCCACCTTGGCCTCCCAAATTGCTGGGATTACAGACGTGAACCAC
J00180:83:H22V2BBXY:8:1215:22171:9596	129	trim24_vector	14099	60	52M23S	14	30952065	GTTGCATCATTTTGTCTGACTAGGTGTCCTTCTATAATATTATGGGGTGGAGATGCCGGTTTGACCTAGTGACAG
J00180:83:H22V2BBXY:8:2220:7801:5376	145	trim24_vector	14210	60	75M	14	58237308	ATTGGGAACCAAGCTGGAGTGCAGTGGCACAATCTTGGCTCACTGCAATCTCCGCCTCCTGGGTTCAAGCGATTC
J00180:83:H22V2BBXY:8:1208:10561:35233	97	trim24_vector	14260	60	75M	14	54140366	TCCGCCTCCTGGGTTCAAGCGATTCTCCTGCCTCAGCCTCCCGAGTTGTTGGGATTCCAGGCATGCATGACCAGG
J00180:83:H22V2BBXY:8:1111:20669:39559	177	trim24_vector	14344	51	22S53M	14	38085291	GGTTTCTTTTTTTTTGTTTTTGTTTTTGTTTTTTTGGTAGAGACGGGGTTTCACCATATTGGCCAGACTGGTCTC
J00180:83:H22V2BBXY:8:1215:15463:37818	129	trim24_vector	2792	60	58M17S	15	17455155	TCACTGATTTTGAACTATAACGACCGCGTGAGTCAAAATGACGCATGATTATCTTTTAGGTGCTAGTTTGTATCC
J00180:83:H22V2BBXY:8:2222:11089:41774	145	trim24_vector	4650	60	75M	15	103513304	GGGGCTTTCGTGGCCGCCGGGCCGCTCGGTGGGACGGAAGCGTGTGGAGAGACCGCCAAGGGCTGTAGTCTGGGT
J00180:83:H22V2BBXY:8:1114:19136:23399	129	trim24_vector	13808	60	75M	15	4321927	AGGATCGCCAGCTGCTTAAGGACTACAAAGACCATGACGGTGATTATAAAGATCATGATATCGATTACAAGGATG
J00180:83:H22V2BBXY:8:2110:22597:36464	177	trim24_vector	14111	60	75M	15	62733788	TGTCTGACTAGGTGTCCTTCTATAATATTATGGGGTGGAGAGGGGTGGTATGGAGCAAGGGGCAAGTTGGGAAGA
J00180:83:H22V2BBXY:8:2205:11069:13183	161	trim24_vector	14133	60	48M27S	15	62277971	TAATATTATGGGGTGGAGGGGGGTGGTATGGAGCAAGGGGCAAGTTGGTACCAGGACTGTAGGGTTGTTGTTTTG
J00180:83:H22V2BBXY:8:1119:7172:27356	177	trim24_vector	14141	60	13S62M	15	7928590	CCTGGGCTTTTTTTGGGGTGGAGGGGGGTGGTATGGAGCAAGGGGCAAGTTGGGAAGACAACCTGTAGGGCCTGC
J00180:83:H22V2BBXY:8:2118:19928:7785	81	trim24_vector	14148	60	75M	15	97593531	GAGGGGGGTGGTATGGAGCAAGGGGCAAGTTGGGAAGACAACCTGTAGGGCCTGCGGGGTCTATTGGGAACCAAG
J00180:83:H22V2BBXY:8:1117:9414:29694	161	trim24_vector	14192	60	75M	15	62138554	GTAGGGCCTGCGGGGTCTATTGGGAACCAAGCTGGAGTGCAGTGGCACAATCTTGGCTCACTGCAATCTCCGCCT
J00180:83:H22V2BBXY:8:2222:9587:38539	65	trim24_vector	14241	60	61M14S	15	61913605	ATCTTGGCTCACTGCAATCTCCGCCTCCTGGGTTCAAGCGATTCTCCTGCCTCAGCCTCCCTACTGGCCTCCAGG
J00180:83:H22V2BBXY:8:1226:27732:12867	65	trim24_vector	14276	60	75M	15	101120443	AAGCGATTCTCCTGCCTCAGCCTCCCGAGTTGTTGGGATTCCAGGCATGCATGACCAGGCTCAGCTAATTTTTGT
J00180:83:H22V2BBXY:8:2108:28534:35075	81	trim24_vector	14344	60	17S58M	15	52034725	GCACCAATGTACCATTCTTTTTGTTTTTTTGGTAGAGACGGGGTTTCACCATATTGGCCAGGCTGGTCTCCAACT
J00180:83:H22V2BBXY:8:1108:1925:9051	177	trim24_vector	14353	60	75M	15	20658294	TTTTGGTAGAGACGGGGTTTCACCATATTGGCCAGGCTGGTCTCCAACTCCTAATCTCAGGTGATCTACCCACCT
J00180:83:H22V2BBXY:8:1211:21876:21465	113	trim24_vector	14613	12	21M54S	15	58870365	GTAGATGCCTGTTGAATTCGATATCAAGCTTATCGATAAGCTTATATTCCATGCTAGGGTTCTGGTGTTGGTGCG
J00180:83:H22V2BBXY:8:1123:7192:10440	129	trim24_vector	12790	0	75M	16	77207186	GGAGCTGATTCTACTCACAAGGTCCCAGTAGTCATGCTGGAGCCAATTCGAATAAAACAGGAAAACAGTGGACCA
J00180:83:H22V2BBXY:8:2116:11424:33387	177	trim24_vector	13978	60	23S52M	16	52366080	TGATTCTAGAGGTACCGGTTGTTGATCCCCCGGGCTGCAGCCCGGGGGATCTGGGGTGGCATCCCTGTGACCCCT
J00180:83:H22V2BBXY:8:1202:6329:45203	113	trim24_vector	14194	60	75M	16	91907465	AGGGCCTGCGGGGTCTATTGGGAACCAAGCTGGAGTGCAGTGGCACAATCTTGGCTCACTGCAATCTCCGCCTCC
J00180:83:H22V2BBXY:8:1211:7222:39787	97	trim24_vector	14214	60	75M	16	49309711	GGAACCAAGCTGGAGTGCAGTGGCACAATCTTGGCTCACTGCAATCTCCGCCTCCTGGGTTCAAGCGATTCTCCT
J00180:83:H22V2BBXY:8:1214:1499:33563	129	trim24_vector	14346	60	45M30S	16	33207375	TTTGTTTTTTTGGTAGAGACGGGGTTTCACCATATTGGCCAGGCTTGGTGGAGCATTCTATAACTTTAATGTTAG
J00180:83:H22V2BBXY:8:1119:26423:39506	113	trim24_vector	14461	60	35S40M	16	80539184	TTTGTCCACTTCTTTCTTTAAGCAGAGCCTTCTACACCACTGCTCCCTTCCCTGTCCTTCTGATTTTAAAATAAC
J00180:83:H22V2BBXY:8:1116:11799:7310	129	trim24_vector	14511	60	75M	16	5709215	GGAGGACGTCCAGACACAGCATAGGCTACCTGGCCATGCCCAACCGGTGGGACATTTGAGTTGCTTGCTTGGCAC
J00180:83:H22V2BBXY:8:2211:1773:4743	129	trim24_vector	14596	60	38M37S	16	22372547	TGCGTTGGGTCCACTCAGTAGATGCCTGTTGAATTCGATATCAAGCTTATAGTCCATGCTAGGGTTCTGGTGTTG
J00180:83:H22V2BBXY:8:1210:6664:17403	81	trim24_vector	3303	0	75M	17	68303734	CAGCTGCCTGTCCCAGTCCTACTGACCCAGCTGTATCTCTCCAGGCAAGCTCTTCCACCCCTTCTGCTTGCATCC
J00180:83:H22V2BBXY:8:1209:19451:12058	129	trim24_vector	14087	60	67M8S	17	46016794	TAATAAAATTAAGTTGCATCATTTTGTCTGACTAGGTGTCCTTCTATAATATTATGGGGCGGAGGGGAGATGGCT
J00180:83:H22V2BBXY:8:1216:21745:1332	81	trim24_vector	14128	60	13S62M	17	16703367	AACCAATCTTAAATTCTATAATATTATGGGGTGGAGGGGGGTGGTATGGAGCAAGGGGCAAGTTGGGAAGACAAC
J00180:83:H22V2BBXY:8:1222:17584:10581	97	trim24_vector	14206	60	75M	17	70912287	GTCTATTGGGAACCAAGCTGGAGTGCAGTGGCACAATCTTGGCTCACTGCAATCTCCGCCTCCTGGGTTCAAGCG
J00180:83:H22V2BBXY:8:1222:17624:10862	1121	trim24_vector	14206	60	75M	17	70912287	GTCTATTGGGAACCAAGCTGGAGTGCAGTGGCACAATCTTGGCTCACTGCAATCTCCGCCTCCTGGGTTCAAGCG
J00180:83:H22V2BBXY:8:1222:17858:10774	1121	trim24_vector	14206	60	75M	17	70912287	GTCTATTGGGAACCAAGCTGGAGTGCAGTGGCACAATCTTGGCGCACTGCAATCTCCGCCTCCTGGGTTCAAGCG
J00180:83:H22V2BBXY:8:1225:6786:33510	161	trim24_vector	14234	60	72M3S	17	45513383	TGGCACAATCTTGGCTCACTGCAATCTCCGCCTCCTGGGTTCAAGCGATTCTCCTGCCTCTGCCTCCCGAGTGCT
J00180:83:H22V2BBXY:8:2225:24424:20181	161	trim24_vector	14401	60	75M	17	32812476	TCCTAATCTCAGGTGATCTACCCACCTTGGCCTCCCAAATTGCTGGGATTACAGGCGTGAACCACTGCTCCCTTC
J00180:83:H22V2BBXY:8:1121:2290:9438	177	trim24_vector	14413	60	75M	17	11371872	GTGATCTACCCACCTTGGCCTCCCAAATTGCTGGGATTACAGGCGTGAACCACTGCTCCCTTCCCTGTCCTTCTG
J00180:83:H22V2BBXY:8:1217:10490:35954	113	trim24_vector	14442	60	75M	17	39771995	GCTGGGATTACAGGCGTGAACCACTGCTCCCTTCCCTGTCCTTCTGATTTTAAAATAACTATACCAGCAGGAGGA
J00180:83:H22V2BBXY:8:2105:8085:19795	65	trim24_vector	13444	60	59M16S	18	79278524	GCTTTCCAAGACCCTGTTCCTCTAACTGTGCCTGATTATTATAAAATAATTAAAAACCCCTGAGGGATGAATCTT
J00180:83:H22V2BBXY:8:2110:11373:10405	177	trim24_vector	13978	35	39S36M	18	80463692	TGGAAGCTAAAAAGCCGAATTCTAGAGGTACCGGTTGTTGATCCCCCGGGCTGCAGCCCGGGGAATCTGGGGTGG
J00180:83:H22V2BBXY:8:1217:22171:4180	1169	trim24_vector	14124	60	75M	18	81530281	GTCCTTCTATAATATTATGGGGTGGAGGGGGGTGGTATGGAGCAAGGGGCAAGTTGGGAAGACAACCTGTAGGGC
J00180:83:H22V2BBXY:8:2202:2493:39787	81	trim24_vector	14124	60	75M	18	81530281	GTCCTTCTATAATATTATGGGGTGGAGGGGGGTGGTATGGAGCAAGGGGCAAGTTGGGAAGACAACCTGTAGGGC
J00180:83:H22V2BBXY:8:2202:3102:39611	1105	trim24_vector	14124	60	75M	18	81530281	GTCCTTCTATAATATTATGGGGTGGAGGGGGGTGGTATGGAGCAAGGGGCAAGTTGGGAAGACAACCTGTAGGGC
J00180:83:H22V2BBXY:8:2220:18385:46153	97	trim24_vector	15033	0	75M	18	15504685	ATCAAACATGCAGGCTCAGACACAGGGACCAGCAGTGTCTGTGGCCTTTTTGTGCTCCTCTCCATGCTGGGTTTG
J00180:83:H22V2BBXY:8:1221:20770:25984	145	trim24_vector	11628	60	75M	19	18467181	GGAGATAAACAAAAAAGGGAAAGCTCTGCTGCACCAGCTTGAGAGTCTTGCAAAGGACCATCGAATGAAACTCAT
J00180:83:H22V2BBXY:8:2215:8684:2017	177	trim24_vector	12029	0	3S72M	19	37305743	GGGCTTCCAACCAGTTATCCAAGTTCCCAACACAGATCAGCCTAGCTCAGTTACAACTCCAGCATATTCAGCAAC
J00180:83:H22V2BBXY:8:1225:23155:8031	65	trim24_vector	14195	60	75M	19	44344006	GGGCCTGCGGGGTCTATTGGGAACCAAGCTGGAGTGCAGTGGCACAATCTTGGCTCACTGCAATCTCCGCCTCCT
J00180:83:H22V2BBXY:8:1225:23165:8013	1089	trim24_vector	14195	60	75M	19	44344006	GGGCCTGCGGGGTCTATTGGGAACCAAGCTGGAGTGCAGTGGCACAATCTTGGCTCACTGCAATCTCCGCCTCCT
J00180:83:H22V2BBXY:8:1102:13423:38328	97	trim24_vector	3319	0	75M	MT	16232	TCCTACTGACCCAGCTGTATCTCTCCAGGCAAGCTCTTCCACCCCTTCTGCTTGCATCCAGACACCATCAAACAC

J00180:83:H22V2BBXY:8:2215:25540:43638	177	trim24_vector	14212	60	75M	MT	11424	TGGGAACCAAGCTGGAGTGCAGTGGCACAATCTTGGCTCACTGCAATCTCCGCCTCCTGGGTTCAAGCGATTCTC
J00180:83:H22V2BBXY:8:2114:18791:27268	113	trim24_vector	57	60	75M	X	47052592	CATTCAAATATGTATCCGCTCATGAGACAATAACCCTGATAAATGCTTCAATAATATTGAAAAAGGAAGAGTATG
J00180:83:H22V2BBXY:8:2227:12905:40807	129	trim24_vector	14136	60	75M	X	93386038	TATTATGGGGTGGAGGGGGGTGGTATGGAGCAAGGGGCAAGTTGGGAAGACAACCTGTAGGGCCTGCGGGGTCTA
J00180:83:H22V2BBXY:8:2102:12997:11073	81	trim24_vector	14163	60	4S71M	X	23931629	ATATGAGCAAGGGGCAAGTTGGGAAGACAACCTGTAGGGCCTGCGGGGTCTATTGGGAACCAAGCTGGAGTGCAG
J00180:83:H22V2BBXY:8:1117:11160:13341	177	trim24_vector	14178	60	75M	X	122334535	TGGGAAGACAACCTGTAGGGCCTGCGGGGTCTATTGGGAACCAAGCTGGAGTGCAGTGGCACAATCTTGGCTCAC
J00180:83:H22V2BBXY:8:2105:2067:1736	161	trim24_vector	14187	60	75M	X	141829210	AACCTGTAGGGCCTGCGGGGTCTATTGGGAACCAAGCTGGAGTGCAGTGGCACAATCTTGGCTCACTGCAATCTC
J00180:83:H22V2BBXY:8:1203:4574:27496	129	trim24_vector	14226	60	75M	X	107381999	GAGTGCAGTGGCACAATCTTGGCTCACTGCAATCTCCGCCTCCTGGGTTCAAGCGATTCTCCTGCCTCAGCCTCC
J00180:83:H22V2BBXY:8:1205:9283:2985	97	trim24_vector	14391	60	45M30S	X	67300498	GGTCTCCAACTCCTAATCTCAGGTGATCTACCCACCTTGGCCTCCACATAGATGTCCCAATCTCCCACAGCACCT
J00180:83:H22V2BBXY:8:2102:31680:40877	97	trim24_vector	14604	60	30M45S	X	21738312	GTCCACTCAGTAGATGCCTGTTGAATTCGATATCAAGCTTATCGATAAGCTTATATTCCATGCTAGGGTGCTGGT
