# Supplementary material for: Mammary-specific expression of Trim24 establishes a mouse model of human metaplastic breast cancer
Source: Nat Commun. 2021 Sep 10;12:5389. doi: 10.1038/s41467-021-25650-z (PMC8433435; doi:10.1038/s41467-021-25650-z)
Supplement: Supplementary file 7 — Dataset 4 [file 41467_2021_25650_MOESM7_ESM.pdf]

Supplementary Table 4: List of TRIM24-driven mouse tumors and their pathological classification, necropsy details and samples selected for RNA-Seq.

Mouse Number	Age	Pathologist-1	Pathologist-2	Mets	Number of tumors	Tumor weight (g)	RNA-Seq	Call based on RNA-Seq
64	13m	Carcinosarcoma	Carcinosarcoma	Lung	3	2.13, 0.48, 0.19		Carcinosarcoma
89	13m	Carcinosarcoma	Carcinoma		1	1.2		Carcinoma
567	11m	Spindle Cell Carcinoma	Carcinosarcoma		1	1.09		Carcinosarcoma
629	8m	Carcinosarcoma	Carcinosarcoma	Liver	2	9.22,0.24		
630	10m	Carcinosarcoma	Carcinosarcoma		1			
779	11m	Adenocarcinoma;Carcinosarcoma	Carcinosarcoma		2			
801	10m	Carcinosarcoma	Carcinosarcoma		4	1, 0.77, 0.53, 0.21		
897	19m	Carcinosarcoma	Carcinosarcoma		2	2.09		Carcinosarcoma
1781	21m	Adenoma	Adenoma		1			
2642	9m	Carcinosarcoma	Carcinosarcoma		1			
3956	19m	Carcinosarcoma	Carcinoma(solid)		1			
273-5	9m	Carcinoma	Carcinoma(solid)		1			Carcinoma
