# Supplementary material for: Mammary-specific expression of Trim24 establishes a mouse model of human metaplastic breast cancer
Source: Nat Commun. 2021 Sep 10;12:5389. doi: 10.1038/s41467-021-25650-z (PMC8433435; doi:10.1038/s41467-021-25650-z)
Supplement: Supplementary file 8 — Dataset 5 [file 41467_2021_25650_MOESM8_ESM.pdf]

Supplementary Table 5: TRIM24 IHC scoring on human MpBC patient samples and associated tumor subtypes.

Patient No	TRIM24 nuc	TRIM24 cyt	TRIM cat Nuc	TRIM cat CYT	p53	date_diagnosis	date_OS	days	OS	MpBC Subtype
1	0	95 M	low	low	pos	5/4/07	10/14/08	529	1	spindle cell/matrix producing
2	0	100 M	low	low	pos	9/3/87	11/5/89	794	0	spindle cell
3	0	90 S	low	high	pos	NA	12/10/19	11381	1	spindle cell
4	15 W	5 M	low	low	neg	1/0/00	10/10/02	37539	1	spindle cell/matrix producing
5	10 W	20 W	low	low	neg	11/22/02	10/26/03	338	1	matrix producing
6	30 M	60 M	high	high	neg	3/14/01	2/25/02	348	1	squamous
7	0	10 W	low	low	neg	1/28/02	7/1/03	519	0	matrix producing
8	5 W	60 W	low	low	pos	12/10/02	4/12/04	489	0	matrix producing
9	10 M	60 M	low	low	neg	5/20/03	2/5/07	1357	0	matrix producing
10	30 M	5 W	high	high	neg	8/8/03	10/1/19	5898	1	matrix producing
11	60 M	50 W	high	high	neg	1/6/04	1/7/20	5845	0	matrix producing
12	70 M	90 W	high	high	neg	6/2/03	3/15/07	1382	1	matrix producing
13	1 M	40 M	low	low	pos	1/19/04	10/10/06	995	0	matrix producing
14	20 W	40 M	low	low	pos	9/24/04	7/20/07	1029	0	spindle cell
15	10 M	30 M	low	low	neg	10/25/04	1/4/07	801	1	spindle cell/matrix producing
16	10 S	60 S	high	high	neg	8/10/01	9/2/11	3675	1	spindle cell
17	5 W	80 S	low	high	neg	3/24/05	4/6/18	4761	1	matrix producing
18	10 M	70 W	low	low	pos	3/22/06	12/10/18	4646	0	spindle cell
19	0	40 M	low	low	pos	4/7/06	3/28/12	2182	0	matrix producing
20	0	80 W	low	low	neg	8/25/06	1/4/19	4515	0	spindle cell
21	50 M	5 W	high	high	pos	2/23/07	10/3/08	588	0	matrix producing
22	20 W	50 W	low	low	neg	3/13/07	1/29/20	4705	0	matrix producing
23	20 S	95W	high	high	pos	3/30/05	6/12/17	4457	1	NA
24	0	1W	low	low	pos	2/5/09	1/3/20	3984	1	spindle cell
25	3W	10 W	low	low	neg	6/5/07	7/15/19	4423	0	matrix producing
26	0	50 M	low	low	pos	3/17/08	3/5/20	4371	1	spindle cell/matrix producing
27	5M	40 W	low	low	pos	7/16/08	4/22/09	280	0	spindle cell/matrix producing
28	1W	95M	low	low	neg	6/25/08	2/10/20	4247	1	spindle cell
29	10 W	25 M	low	low	neg	11/16/08	5/6/19	3823	1	spindle cell
30	20 W	70 M	low	low	neg	5/5/09	9/1/16	2676	0	matrix producing
31	0	10 S	low	high	pos	8/27/10	2/13/11	170	1	spindle cell
32	20 M	10 W	high	high	neg	1/25/10	3/17/16	2243	0	matrix producing
33	15 M	60 M	high	high	pos	8/31/10	3/11/20	3480	1	matrix producing
34	10 W	10 W	low	low	pos	10/20/10	2/13/20	3403	1	spindle cell
35	0	50 M	low	low	neg	10/8/10	1/10/20	3381	1	osteochondroid
36	60 S	20 M	high	high	pos	12/15/10	10/23/11	312	1	spindle cell/matrix producing
37	20 M	15 W	high	high	pos	6/28/10	1/17/12	568	1	matrix producing
38	0	90 M	low	low	pos	3/24/11	7/30/19	3050	0	NA
39	70 S	30 W	high	high	pos	4/12/11	3/12/14	1065	1	matrix producing
40	5 W	20 W	low	low	neg	4/28/11	7/24/19	3009	0	spindle cell
41	30 M	90 M	high	high	pos	4/26/11	9/29/12	522	0	spindle cell
42	40 S	0	high	high	pos	8/30/11	6/19/13	659	1	matrix producing
43	70 W	70 M	high	high	pos	10/25/11	8/12/19	2848	0	matrix producing
44	20 M	50 M	high	high	neg	11/7/11	3/6/20	3042	0	chondrosarcomatoid
45	15 M	60 M	high	high	pos	2/6/12	4/4/18	2249	0	spindle cell
46	3W	60 M	low	low	neg	4/10/09	10/17/12	1286	1	spindle cell/matrix producing
47	10 M	90 M	low	low	neg	6/14/12	8/12/19	2615	1	spindle cell
