# Supplementary material for: Mammary-specific expression of Trim24 establishes a mouse model of human metaplastic breast cancer
Source: Nat Commun. 2021 Sep 10;12:5389. doi: 10.1038/s41467-021-25650-z (PMC8433435; doi:10.1038/s41467-021-25650-z)
Supplement: Supplementary file 9 — Dataset 6 [file 41467_2021_25650_MOESM9_ESM.pdf]

Supplementary Table 6: List of differentially expressed genes of murine TRIM24-driven tumors and control mammary glands.

												MMTV-Cre Mammary glands					TRIM24-driven mammary tumors				
**Gene ID**	**Gene Name**	**p.value**	**FDR**	**Num Samples Normal**	**Num Samples TRIM24**	**Mean Normal**	**Mean TRIM24**	**Delta**	**Log_2 Fold Change**	**Log_2 Fold Change (MLE)**	**Direction**	**133**	**392**	**417_3**	**517_3**	**273_5**	**3956**	**567**	**64**	**89**	**897**
ENSMUSG00000025804.5	Ccr1	2.92E-53	8.90E-49	4	6	143.25	3454.833333	3311.583333	4.440113706	4.506918366	Higher in TRIM24	137	133	138	165	2374	3179	3781	1935	2660	6800
ENSMUSG00000027875.12	Hmgcs2	1.05E-46	1.60E-42	4	6	3701	24.16666667	3676.833333	7.137844125	7.4684603	Higher in Normal	1242	2139	5064	6359	17	6	27	5	30	60
ENSMUSG00000025044.16	Msr1	1.02E-34	1.03E-30	4	6	234.5	7429.833333	7195.333333	4.740280899	4.870923304	Higher in TRIM24	213	205	253	267	3453	7175	6984	5365	3456	18146
ENSMUSG00000030669.13	Calca	1.66E-32	1.27E-28	4	6	6848	15	6833	7.952548375	8.682478214	Higher in Normal	2753	2850	8267	13522	7	1	11	38	15	18
ENSMUSG00000029304.14	Spp1	3.55E-31	2.16E-27	4	6	1984.5	118640.8333	116656.3333	5.577599525	5.82594988	Higher in TRIM24	4130	549	1134	2125	98837	77637	67916	104924	146527	216004
ENSMUSG00000029915.14	Clec5a	1.42E-28	7.22E-25	4	6	74	1455.166667	1381.166667	4.147579805	4.254781871	Higher in TRIM24	84	79	62	71	766	1451	2021	1015	1202	2276
ENSMUSG00000024386.8	Proc	2.52E-28	1.10E-24	4	6	153	9.5	143.5	4.001860471	4.098672659	Higher in Normal	97	153	158	204	7	8	9	5	13	15
ENSMUSG00000017309.11	Cd300lg	3.13E-28	1.19E-24	4	6	6303.25	400.1666667	5903.083333	4.02670184	4.124149453	Higher in Normal	5788	6042	5883	7500	168	219	551	170	687	606
ENSMUSG00000040136.10	Abcc8	4.25E-27	1.29E-23	4	6	635.25	15	620.25	5.357006666	5.605350996	Higher in Normal	671	359	800	711	4	5	15	3	37	26
ENSMUSG00000031410.14	Nxf7	1.50E-26	3.92E-23	4	6	932.25	11.83333333	920.4166667	6.001647914	6.369676288	Higher in Normal	413	412	1274	1630	5	6	17	1	25	17
ENSMUSG00000024679.10	Ms4a6d	1.55E-26	3.92E-23	4	6	230.25	4494	4263.75	3.964873421	4.065911469	Higher in TRIM24	187	338	182	214	2760	3416	4777	2555	2901	10555
ENSMUSG00000019768.16	Esr1	1.81E-24	4.24E-21	4	6	1885.25	287.1666667	1598.083333	2.80159839	2.839161207	Higher in Normal	1795	1620	2106	2020	149	115	336	248	374	501
ENSMUSG00000041324.14	Inhba	7.58E-24	1.65E-20	4	6	252.25	8512.666667	8260.416667	4.929886552	5.158499526	Higher in TRIM24	288	166	210	345	1954	9015	10649	8644	12528	8286
ENSMUSG00000037613.16	Tnfrsf23	1.52E-23	3.09E-20	4	6	138.5	5009.666667	4871.166667	5.041437199	5.291340913	Higher in TRIM24	164	109	127	154	1565	8734	4492	3961	4894	6412
ENSMUSG00000071547.3	Nt5dc2	1.91E-23	3.64E-20	4	6	178	3895.333333	3717.333333	4.054370849	4.179036994	Higher in TRIM24	130	100	218	264	1250	1091	3621	2664	3408	11338
ENSMUSG00000025927.13	Tfap2b	3.29E-23	5.89E-20	4	6	1983	45.16666667	1937.833333	5.503939734	5.827984245	Higher in Normal	692	1789	2652	2799	14	5	26	19	81	126
ENSMUSG00000033213.16	AA467197	4.49E-23	7.59E-20	4	6	26	908.1666667	882.1666667	4.789527414	5.007982938	Higher in TRIM24	22	44	20	18	682	808	1059	787	350	1763
ENSMUSG00000030792.8	Dkkl1	1.82E-22	2.92E-19	4	6	983	40.33333333	942.6666667	4.510128679	4.689632491	Higher in Normal	590	659	1085	1598	35	9	57	14	78	49
ENSMUSG00000047562.3	Mmp10	1.02E-21	1.55E-18	4	6	13.5	5922.666667	5909.166667	7.171348661	8.154707331	Higher in TRIM24	11	10	16	17	143	2724	2307	643	3392	26327
ENSMUSG00000042265.13	Trem1	1.13E-21	1.64E-18	4	6	23.75	865.6666667	841.9166667	4.966849541	5.230536701	Higher in TRIM24	24	11	27	33	311	997	1544	265	945	1132
ENSMUSG00000023913.17	Pla2g7	1.19E-21	1.64E-18	4	6	1362.25	13438.83333	12076.58333	2.993838829	3.046884569	Higher in TRIM24	838	999	1473	2139	9346	5691	10925	5622	11602	37447
ENSMUSG00000066000.12	Zfp979	1.87E-21	2.48E-18	4	6	710.75	89.33333333	621.4166667	3.130419765	3.191389685	Higher in Normal	1039	592	606	606	48	38	100	52	130	168
ENSMUSG00000022156.7	Gzme	3.26E-21	4.14E-18	4	6	4.25	965.8333333	961.5833333	6.890487172	7.780042331	Higher in TRIM24	10	2	4	1	363	763	1668	162	2375	464
ENSMUSG00000001403.13	Ube2c	6.66E-21	8.12E-18	4	6	446.25	3943.333333	3497.083333	3.006415208	3.062314488	Higher in TRIM24	327	261	477	720	2723	1391	4198	3795	3641	7912
ENSMUSG00000047631.3	Apof	9.78E-21	1.15E-17	4	6	360.75	4	356.75	6.06671852	6.591480758	Higher in Normal	58	130	527	728	3	1	2	2	7	9
ENSMUSG00000058914.12	C1qtnf3	1.09E-20	1.23E-17	4	6	30.75	2529.833333	2499.083333	5.827004607	6.308614431	Higher in TRIM24	50	18	25	30	414	777	6314	1671	3954	2049
ENSMUSG00000037280.12	Galnt6	2.48E-20	2.70E-17	4	6	92	616.3333333	524.3333333	2.571298827	2.606989894	Higher in TRIM24	52	91	111	114	356	397	678	385	576	1306
ENSMUSG00000111602.1	Gm32014	2.14E-19	2.23E-16	4	6	82.5	5.666666667	76.83333333	3.868260841	4.001822149	Higher in Normal	85	71	109	65	6	3	2	3	10	10
ENSMUSG00000001131.11	Timp1	2.20E-19	2.23E-16	4	6	382.75	18645.16667	18262.41667	5.235960479	5.596938273	Higher in TRIM24	146	331	388	666	9187	6484	15933	34687	5504	40076
ENSMUSG00000095497.2	Igkv1-122	4.14E-19	4.07E-16	4	6	260.25	1.5	258.75	6.821895327	7.741674372	Higher in Normal	48	399	238	356	0	1	3	0	0	5
ENSMUSG00000024039.14	Cbs	4.79E-19	4.56E-16	4	6	471.25	28.66666667	442.5833333	4.032106395	4.184961771	Higher in Normal	357	309	520	699	37	4	30	8	42	51
ENSMUSG00000030849.18	Fgfr2	1.28E-18	1.18E-15	4	6	4240.5	223.1666667	4017.333333	4.053231055	4.212961504	Higher in Normal	1566	2370	6540	6486	171	116	407	104	291	250
ENSMUSG00000031209.14	Heph	2.60E-18	2.33E-15	4	6	2306.25	136.6666667	2169.583333	4.105865193	4.275408671	Higher in Normal	2463	2357	2194	2211	40	54	183	52	307	184
ENSMUSG00000022211.8	Carmil3	4.14E-18	3.60E-15	4	6	108.25	12.33333333	95.91666667	3.21478479	3.295592675	Higher in Normal	116	121	113	83	12	6	7	9	18	22
ENSMUSG00000026193.15	Fn1	5.04E-18	4.27E-15	4	6	4037.25	165664.5	161627.25	5.043252865	5.392248262	Higher in TRIM24	2187	2623	5379	5960	34887	204775	175420	165761	39975	373169
ENSMUSG00000020672.14	Sntg2	7.02E-18	5.78E-15	4	6	216.5	13.66666667	202.8333333	3.981792694	4.140209919	Higher in Normal	140	186	247	293	4	3	18	7	29	21
ENSMUSG00000056071.12	S100a9	7.71E-18	6.03E-15	4	6	41.5	3028.333333	2986.833333	5.440349089	5.901603969	Higher in TRIM24	45	42	28	51	1697	226	1219	1403	9368	4257
ENSMUSG00000003477.5	Inmt	7.73E-18	6.03E-15	4	6	857	22	835	5.468218142	5.906327331	Higher in Normal	1325	1179	359	565	4	2	16	8	20	82
ENSMUSG00000049115.15	Agtr1a	1.24E-17	9.02E-15	4	6	1808.75	98	1710.75	4.120888697	4.300365178	Higher in Normal	1656	2086	1665	1828	41	30	123	89	224	81
ENSMUSG00000063430.9	Wscd2	1.24E-17	9.02E-15	4	6	382.25	25	357.25	4.010141136	4.175037875	Higher in Normal	562	372	286	309	10	8	10	25	46	51
ENSMUSG00000097383.1	1500026H17Rik	1.56E-17	1.11E-14	4	6	74.25	6.833333333	67.41666667	3.424608927	3.527950919	Higher in Normal	71	72	71	83	7	2	5	8	10	9
ENSMUSG00000029282.3	Amtn	1.77E-17	1.23E-14	4	6	462.25	2.166666667	460.0833333	6.864705261	7.91411774	Higher in Normal	29	272	634	914	4	1	0	0	1	7
ENSMUSG00000017724.14	Etv4	2.01E-17	1.36E-14	4	6	484	3672.833333	3188.833333	2.95191398	3.016912567	Higher in TRIM24	289	233	639	775	2784	3314	3767	3110	4150	4912
ENSMUSG00000052316.9	Lrrc15	2.56E-17	1.66E-14	4	6	142.75	7229.166667	7086.416667	5.269207678	5.697128865	Higher in TRIM24	114	56	173	228	831	6247	17824	2659	5769	10045
ENSMUSG00000058297.16	Spock2	2.92E-17	1.84E-14	4	6	2211	70.5	2140.5	4.677886409	4.953591688	Higher in Normal	705	903	3551	3685	20	23	114	59	122	85
ENSMUSG00000029335.5	Bmp3	2.96E-17	1.84E-14	4	6	2197.75	61	2136.75	4.836667083	5.144529312	Higher in Normal	1674	3437	1801	1879	21	96	46	39	123	41
ENSMUSG00000048776.8	Pthlh	3.07E-17	1.87E-14	4	6	104.25	1601.833333	1497.583333	3.797550747	3.942826567	Higher in TRIM24	44	49	152	172	1224	1430	809	722	2909	2517
ENSMUSG00000042684.8	Npl	3.14E-17	1.87E-14	4	6	182.5	3059.833333	2877.333333	3.770649242	3.912951293	Higher in TRIM24	349	133	109	139	3026	1666	3063	2498	1400	6706
ENSMUSG00000028996.9	Rbp7	4.33E-17	2.54E-14	4	6	1408	115.8333333	1292.166667	3.608943258	3.73183277	Higher in Normal	1837	1445	848	1502	84	32	80	156	145	198
ENSMUSG00000023349.14	Clec4n	5.04E-17	2.90E-14	4	6	278	7083.333333	6805.333333	4.404930548	4.643665878	Higher in TRIM24	230	168	311	403	1787	5654	15402	3696	3208	12753
ENSMUSG00000027931.12	Npr1	8.14E-17	4.59E-14	4	6	2458.25	448	2010.25	2.563043619	2.606522446	Higher in Normal	1886	2088	2977	2882	328	158	558	203	692	749
ENSMUSG00000050315.13	Synpo2	8.90E-17	4.93E-14	4	6	5524.75	350.3333333	5174.416667	3.958554452	4.126165537	Higher in Normal	3175	5799	7312	5813	175	109	702	118	504	494
ENSMUSG00000050578.10	Mmp13	1.06E-16	5.70E-14	4	6	285.75	51719.5	51433.75	6.412367934	7.35288121	Higher in TRIM24	148	100	374	521	1042	49493	82749	5454	27585	143994
ENSMUSG00000068246.5	Apol9b	1.07E-16	5.70E-14	4	6	108.75	2316.5	2207.75	4.004921099	4.184504733	Higher in TRIM24	64	187	72	112	1087	2318	1042	1173	3271	5008
ENSMUSG00000056054.9	S100a8	1.24E-16	6.51E-14	4	6	35.5	3310.333333	3274.833333	5.691260992	6.289769918	Higher in TRIM24	44	41	17	40	3462	257	1017	1083	10764	3279
ENSMUSG00000036362.2	P2ry13	1.27E-16	6.53E-14	4	6	59.75	729.6666667	669.9166667	3.481581747	3.597105467	Higher in TRIM24	66	66	39	68	829	868	597	420	454	1210
ENSMUSG00000033581.16	Igf2bp2	1.29E-16	6.53E-14	4	6	224.25	3457.666667	3233.416667	3.557403544	3.680859568	Higher in TRIM24	331	216	158	192	1284	2511	2396	2703	1921	9931
ENSMUSG00000020154.10	Ptprb	1.40E-16	6.98E-14	4	6	5987.75	953.1666667	5034.583333	2.665128864	2.714942071	Higher in Normal	5053	3227	9183	6488	526	635	1166	433	1421	1538
ENSMUSG00000025161.16	Slc16a3	1.49E-16	7.31E-14	4	6	368.25	8962.833333	8594.583333	4.1061313	4.302531043	Higher in TRIM24	148	262	444	619	1514	3857	7870	4803	7795	27938
ENSMUSG00000076580.3	Igkv8-27	2.47E-16	1.19E-13	4	6	277.5	4.833333333	272.6666667	5.479801343	5.984454426	Higher in Normal	26	183	378	523	5	2	2	1	8	11
ENSMUSG00000027848.12	Olfml3	2.49E-16	1.19E-13	4	6	560.25	6998.666667	6438.416667	3.527169474	3.649768732	Higher in TRIM24	510	398	553	780	1912	4780	10495	7974	6061	10770
ENSMUSG00000057346.11	Apol9a	2.83E-16	1.33E-13	4	6	122	2083.666667	1961.666667	3.691644134	3.834162591	Higher in TRIM24	60	176	94	158	1746	597	946	1392	3629	4192
ENSMUSG00000058441.7	Panx2	3.17E-16	1.46E-13	4	6	106	7.5	98.5	3.736653906	3.885837634	Higher in Normal	61	136	103	124	3	9	8	6	8	11
ENSMUSG00000078780.7	Gm5150	3.64E-16	1.65E-13	4	6	25.75	447.3333333	421.5833333	3.790989378	3.948316348	Higher in TRIM24	38	29	16	20	440	458	329	146	294	1017
ENSMUSG00000056515.8	Rab31	3.96E-16	1.77E-13	4	6	1198.75	10273	9074.25	2.952436408	3.023580321	Higher in TRIM24	934	1016	1308	1537	3781	10465	11382	4941	11700	19369
ENSMUSG00000038642.10	Ctss	4.37E-16	1.93E-13	4	6	3680	32999.83333	29319.83333	3.015175695	3.091373156	Higher in TRIM24	2077	4241	3443	4959	26017	25619	38924	25752	16979	64708
ENSMUSG00000043668.13	Tox3	4.81E-16	2.09E-13	4	6	229.75	7.833333333	221.9166667	4.731892574	5.04496506	Higher in Normal	78	84	372	385	3	2	6	2	16	18
ENSMUSG00000007888.15	Crlf1	6.82E-16	2.92E-13	4	6	91.25	3220.666667	3129.416667	4.939999967	5.323987053	Higher in TRIM24	73	64	83	145	8188	1706	2203	3654	762	2811
ENSMUSG00000022144.3	Gdnf	9.23E-16	3.88E-13	4	6	20.25	855.3333333	835.0833333	4.900999187	5.279551114	Higher in TRIM24	8	12	32	29	89	888	1044	290	619	2202
ENSMUSG00000029177.9	Cenpa	1.18E-15	4.84E-13	4	6	437.75	3145.833333	2708.083333	2.71008363	2.766521263	Higher in TRIM24	489	222	530	510	2970	910	3062	3077	2914	5942
ENSMUSG00000071347.3	C1qtnf9	1.19E-15	4.84E-13	4	6	1385	115.3333333	1269.666667	3.675714716	3.819912814	Higher in Normal	1666	1791	908	1175	64	26	156	66	211	169
ENSMUSG00000034488.14	Edil3	1.26E-15	5.03E-13	4	6	94.25	1114	1019.75	3.309959669	3.415682122	Higher in TRIM24	118	62	130	67	578	443	1747	878	632	2406
ENSMUSG00000040283.14	Btnl9	1.56E-15	6.16E-13	4	6	3019	277.8333333	2741.166667	3.509299161	3.635157881	Higher in Normal	3149	3945	2542	2440	87	92	353	268	409	458
ENSMUSG00000026773.19	Pfkfb3	1.67E-15	6.51E-13	4	6	12142.5	2419.166667	9723.333333	2.49721336	2.541268062	Higher in Normal	10783	9689	14907	13191	2276	867	1814	850	3728	4980
ENSMUSG00000047798.15	Cd300lf	2.47E-15	9.41E-13	4	6	102	1424.166667	1322.166667	3.599263632	3.740413751	Higher in TRIM24	145	62	87	114	744	1449	1219	415	2801	1917
ENSMUSG00000018830.8	Myh11	2.84E-15	1.07E-12	4	6	36665	270.1666667	36394.83333	6.224589997	7.072307577	Higher in Normal	3871	4181	71496	67112	106	57	312	65	935	146
ENSMUSG00000002944.15	Cd36	2.99E-15	1.11E-12	4	6	50713	2116.666667	48596.33333	4.510087183	4.795274206	Higher in Normal	65712	56574	37573	42993	1775	596	1909	383	6135	1902
ENSMUSG00000030560.17	Ctsc	3.59E-15	1.30E-12	4	6	3865	20779.16667	16914.16667	2.373703645	2.41258718	Higher in TRIM24	2991	3988	3857	4624	15290	19252	25066	15916	16521	32630
ENSMUSG00000038393.14	Txnip	4.37E-15	1.53E-12	4	6	68732.5	7775.666667	60956.83333	3.107165022	3.196323777	Higher in Normal	23631	75646	77747	97906	5017	4125	10740	5864	10214	10694

ENSMUSG00000028132.15	Tmem56	5.45E-15	1.89E-12	4	6	6054.25	159	5895.25	4.788295353	5.142685416	Higher in Normal	2374	4027	9902	7914	105	32	397	152	198	70
ENSMUSG00000044811.13	Cd300c2	8.67E-15	2.93E-12	4	6	229	1674.166667	1445.166667	2.70510641	2.7651202	Higher in TRIM24	154	258	214	290	953	1314	2161	1173	1054	3390
ENSMUSG00000048329.7	Mfsd6l	9.39E-15	3.14E-12	4	6	161.25	21	140.25	2.989234175	3.07019606	Higher in Normal	115	93	183	254	15	10	22	5	34	40
ENSMUSG00000079227.10	Ccr5	9.71E-15	3.21E-12	4	6	408.5	4105.166667	3696.666667	3.104756863	3.197392838	Higher in TRIM24	375	548	375	336	2243	3844	5480	2466	2432	8166
ENSMUSG00000044303.6	Cdkn2a	1.01E-14	3.29E-12	4	6	143.5	18831	18687.5	5.739321027	6.478346601	Higher in TRIM24	83	320	71	100	3687	1385	22212	16559	3397	65746
ENSMUSG00000038765.13	Lmx1b	1.06E-14	3.43E-12	4	6	1508.25	50.66666667	1457.583333	4.601299076	4.920238272	Higher in Normal	422	569	2490	2552	38	13	40	21	145	47
ENSMUSG00000028364.15	Tnc	1.09E-14	3.50E-12	4	6	1388.5	60393.5	59005	5.1970191	5.708320423	Higher in TRIM24	665	349	2216	2324	9496	46138	119603	104670	33322	49132
ENSMUSG00000082908.4	Gm13736	1.21E-14	3.84E-12	4	6	12.75	1311.5	1298.75	5.772752215	6.542242109	Higher in TRIM24	12	15	9	15	1590	10	903	1841	972	2553
ENSMUSG00000049036.7	Tmem121	1.27E-14	3.95E-12	4	6	15.25	483.3333333	468.0833333	4.555318861	4.882350802	Higher in TRIM24	13	15	15	18	209	134	488	825	118	1126
ENSMUSG00000074093.4	Svip	1.27E-14	3.95E-12	4	6	732.25	124.5	607.75	2.775469235	2.840635052	Higher in Normal	897	872	565	595	66	42	127	68	181	263
ENSMUSG00000030731.13	Syt3	1.38E-14	4.21E-12	4	6	168.5	32.83333333	135.6666667	2.545372934	2.595377018	Higher in Normal	211	171	132	160	20	12	32	18	45	70
ENSMUSG00000042845.7	Wfdc12	1.42E-14	4.29E-12	4	6	542.25	2	540.25	6.644191286	7.887537117	Higher in Normal	22	25	852	1270	1	1	3	2	4	1
ENSMUSG00000004609.11	Cd33	1.47E-14	4.40E-12	4	6	336.5	2114.333333	1777.833333	2.558425733	2.609827244	Higher in TRIM24	397	231	374	344	931	2149	2091	1592	1958	3965
ENSMUSG00000033777.3	Tlr13	1.53E-14	4.54E-12	4	6	101.75	1598	1496.25	3.501096854	3.639175861	Higher in TRIM24	102	181	64	60	1085	1196	1205	675	1215	4212
ENSMUSG00000032294.17	Pkm	1.75E-14	5.11E-12	4	6	14980.25	114329.3333	99349.08333	2.648555975	2.706103396	Higher in TRIM24	13186	9397	16505	20833	76210	37673	99353	87349	62707	322684
ENSMUSG00000068196.4	Col8a1	1.80E-14	5.22E-12	4	6	999.75	11785.83333	10786.08333	3.558829779	3.704811867	Higher in TRIM24	718	993	1153	1135	7544	9854	17478	17125	10811	7903
ENSMUSG00000024998.17	Plce1	2.28E-14	6.48E-12	4	6	2392	239.8333333	2152.166667	3.226966093	3.333183281	Higher in Normal	1367	1114	4027	3060	193	92	220	149	513	272
ENSMUSG00000024827.9	Gldc	2.30E-14	6.49E-12	4	6	420.25	45.16666667	375.0833333	3.327027976	3.443637661	Higher in Normal	414	236	530	501	13	10	67	25	47	109
ENSMUSG00000022840.8	Adcy5	2.38E-14	6.65E-12	4	6	2613.5	208.5	2405	3.618821502	3.771212192	Higher in Normal	2224	3647	2387	2196	140	113	284	80	465	169
ENSMUSG00000019916.14	P4ha1	2.48E-14	6.87E-12	4	6	1530.75	11793.33333	10262.58333	2.644868471	2.702880703	Higher in TRIM24	1101	1156	1784	2082	5785	4981	9140	9393	6819	34642
ENSMUSG00000058153.15	Sez6l	2.56E-14	7.02E-12	4	6	305	13	292	4.328992989	4.600132422	Higher in Normal	137	70	508	505	12	2	12	6	26	20
ENSMUSG00000066687.5	Zbtb16	2.84E-14	7.64E-12	4	6	1316	40.5	1275.5	4.876381612	5.276881969	Higher in Normal	881	849	2259	1275	4	13	33	6	117	70
ENSMUSG00000042961.13	Egflam	3.12E-14	8.32E-12	4	6	1128.25	186.3333333	941.9166667	2.585953914	2.64011479	Higher in Normal	1053	862	1197	1401	155	76	320	145	145	277
ENSMUSG00000036813.11	Entpd8	3.37E-14	8.93E-12	4	6	416	3.833333333	412.1666667	6.00159607	6.844302365	Higher in Normal	79	58	760	767	1	0	3	1	13	5
ENSMUSG00000056758.14	Hmga2	3.53E-14	9.27E-12	4	6	40	9396	9356	6.479305777	7.803875689	Higher in TRIM24	99	37	8	16	2712	19321	5586	5256	160	23341
ENSMUSG00000076564.2	Igkv12-46	3.59E-14	9.35E-12	4	6	133.5	12	121.5	3.664151251	3.823602909	Higher in Normal	205	95	98	136	6	2	12	3	14	35
ENSMUSG00000045318.6	Adra2c	3.80E-14	9.80E-12	4	6	224.5	3	221.5	5.519725511	6.161340521	Higher in Normal	56	104	319	419	0	2	5	1	9	1
ENSMUSG00000027221.5	Chst1	3.96E-14	1.01E-11	4	6	4831.5	838	3993.5	2.613526626	2.669938392	Higher in Normal	5761	4609	4021	4935	443	439	1248	668	566	1664
ENSMUSG00000051652.4	Lrrc3	4.34E-14	1.10E-11	4	6	429.5	38	391.5	3.331868749	3.452571463	Higher in Normal	339	404	456	519	61	26	43	17	56	25
ENSMUSG00000062937.7	Mtap	4.49E-14	1.12E-11	4	6	1078.25	6940.333333	5862.083333	2.552795758	2.605899613	Higher in TRIM24	1229	938	961	1185	2632	5899	6659	6513	6525	13414
ENSMUSG00000016942.5	Tmprss6	4.51E-14	1.12E-11	4	6	3502	46.5	3455.5	5.52785791	6.15403554	Higher in Normal	158	333	6134	7383	34	18	34	44	81	68
ENSMUSG00000039349.5	C130074G19Rik	4.58E-14	1.13E-11	4	6	11148.5	1274.833333	9873.666667	3.057866512	3.150005791	Higher in Normal	11890	9301	10103	13300	577	927	1837	751	2398	1159
ENSMUSG00000034450.7	Gulo	5.63E-14	1.38E-11	4	6	75	2.666666667	72.33333333	4.621423865	4.977339757	Higher in Normal	108	31	97	64	0	3	4	0	2	7
ENSMUSG00000027199.14	Gatm	6.13E-14	1.49E-11	4	6	614	8018	7404	3.629530346	3.792426522	Higher in TRIM24	585	699	577	595	7006	8064	5072	13141	3720	11105
ENSMUSG00000053411.16	Cbx7	6.94E-14	1.67E-11	4	6	3563.5	544.8333333	3018.666667	2.815571084	2.887993025	Higher in Normal	2227	3358	4461	4208	699	254	326	164	697	1129
ENSMUSG00000029163.9	Emilin1	6.98E-14	1.67E-11	4	6	415.25	5205	4789.75	3.551961619	3.704658739	Higher in TRIM24	312	333	498	518	3630	2145	5272	9539	2540	8104
ENSMUSG00000028713.17	Cyp4b1	8.01E-14	1.91E-11	4	6	2395.5	139.5	2256	4.040379034	4.266649341	Higher in Normal	1929	2783	2114	2756	64	34	249	42	317	131
ENSMUSG00000041828.15	Abca8a	9.33E-14	2.18E-11	4	6	1743.25	258.1666667	1485.083333	2.767747609	2.837232485	Higher in Normal	2058	1584	1735	1596	90	205	306	169	424	355
ENSMUSG00000045534.4	Kcna5	1.06E-13	2.46E-11	4	6	292.75	35.33333333	257.4166667	3.132471154	3.234924643	Higher in Normal	438	238	233	262	12	14	44	21	64	57
ENSMUSG00000030711.15	Sult1a1	1.15E-13	2.65E-11	4	6	3812.75	182.5	3630.25	4.303477672	4.584894733	Higher in Normal	6209	6125	1191	1726	67	122	213	122	361	210
ENSMUSG00000051048.17	P4ha3	1.52E-13	3.47E-11	4	6	42.5	926.8333333	884.3333333	4.138014097	4.398453777	Higher in TRIM24	88	31	24	27	257	726	1428	666	1667	817
ENSMUSG00000034810.7	Scn7a	1.75E-13	3.95E-11	4	6	5652.25	425	5227.25	3.714016952	3.892392283	Higher in Normal	5683	6364	5810	4752	161	156	487	207	1169	370
ENSMUSG00000076549.2	Igkv4-68	1.96E-13	4.38E-11	4	6	75.75	7.333333333	68.41666667	3.277709415	3.400485506	Higher in Normal	58	50	86	109	11	6	6	1	10	10
ENSMUSG00000074677.11	Sirpb1c	2.04E-13	4.54E-11	4	6	30.25	582.6666667	552.4166667	3.968518801	4.198348832	Higher in TRIM24	45	29	21	26	86	689	655	438	608	1020
ENSMUSG00000022696.17	Sidt1	2.38E-13	5.26E-11	4	6	2448	72.16666667	2375.833333	4.825754204	5.248197099	Higher in Normal	1141	728	3981	3942	62	7	32	14	206	112
ENSMUSG00000041794.13	Myrip	2.58E-13	5.65E-11	4	6	280.25	19.16666667	261.0833333	3.831917263	4.032237268	Higher in Normal	174	268	369	310	28	6	11	2	38	30
ENSMUSG00000029371.7	Cxcl5	2.90E-13	6.27E-11	4	6	32.25	4509.166667	4476.916667	5.825476916	6.757345351	Higher in TRIM24	19	22	21	67	3309	43	1289	528	11536	10350
ENSMUSG00000056602.11	Fry	2.90E-13	6.27E-11	4	6	3037.75	468.3333333	2569.416667	2.79388629	2.868528735	Higher in Normal	3792	3603	2741	2015	578	174	467	227	665	699
ENSMUSG00000017716.15	Birc5	3.04E-13	6.52E-11	4	6	369.5	3311.5	2942	2.970005317	3.061536497	Higher in TRIM24	399	188	371	520	2662	860	3048	3833	2072	7394
ENSMUSG00000032332.17	Col12a1	3.09E-13	6.57E-11	4	6	877.5	24232.66667	23355.16667	4.452584149	4.795528888	Higher in TRIM24	567	184	1548	1211	3821	16427	52259	8842	26546	37501
ENSMUSG00000046245.13	Pilra	3.21E-13	6.79E-11	4	6	131	1531.833333	1400.833333	3.428859275	3.573808723	Higher in TRIM24	107	135	107	175	661	1762	2678	1045	832	2213
ENSMUSG00000021255.17	Esrrb	3.41E-13	7.16E-11	4	6	1295.5	22.5	1273	5.300879626	5.893857699	Higher in Normal	158	90	2429	2505	33	5	13	8	27	49
ENSMUSG00000095407.1	Tmem200c	3.60E-13	7.50E-11	4	6	117	0.833333333	116.1666667	5.956334721	6.994413974	Higher in Normal	16	36	192	224	1	1	1	0	2	0
ENSMUSG00000037852.8	Cpe	3.72E-13	7.71E-11	4	6	2063.75	24350.16667	22286.41667	3.441097201	3.588469153	Higher in TRIM24	825	2366	2145	2919	8748	31655	20265	21897	24392	39144
ENSMUSG00000028965.13	Tnfrsf9	3.97E-13	8.17E-11	4	6	126	4195.5	4069.5	4.475022328	4.828060588	Higher in TRIM24	120	59	168	157	296	1605	6725	2878	1703	11966
ENSMUSG00000062209.15	Erbb4	5.23E-13	1.06E-10	4	6	661.25	18.16666667	643.0833333	5.01474868	5.511899003	Higher in Normal	331	182	1317	815	6	4	5	1	44	49
ENSMUSG00000079419.4	Ms4a6c	5.45E-13	1.10E-10	4	6	202.25	1643	1440.75	2.734641485	2.80704029	Higher in TRIM24	149	294	177	189	1375	1150	1235	1157	875	4066
ENSMUSG00000025902.13	Sox17	5.81E-13	1.16E-10	4	6	1545	242.5	1302.5	2.650599374	2.715798957	Higher in Normal	1098	1109	1822	2151	84	197	321	133	344	376
ENSMUSG00000076550.3	Igkv4-63	6.65E-13	1.32E-10	4	6	207.75	2.166666667	205.5833333	5.710375646	6.546007518	Higher in Normal	11	56	319	445	1	0	4	2	3	3
ENSMUSG00000021904.5	Sema3g	6.98E-13	1.38E-10	4	6	1646.25	198.5	1447.75	2.989150494	3.084500234	Higher in Normal	1433	1481	1828	1843	89	114	265	137	420	166
ENSMUSG00000017412.15	Cacnb4	7.17E-13	1.41E-10	4	6	224.75	26	198.75	3.100026552	3.206943921	Higher in Normal	153	123	372	251	11	16	16	13	58	42
ENSMUSG00000051159.16	Cited1	7.22E-13	1.41E-10	4	6	3885.5	116.8333333	3768.666667	4.525375443	4.884024038	Higher in Normal	1039	3165	4380	6958	123	20	181	208	102	67
ENSMUSG00000028108.15	Ecm1	7.37E-13	1.43E-10	4	6	2189.25	24204.5	22015.25	3.116361714	3.226811856	Higher in TRIM24	1401	2774	2009	2573	6561	12757	32602	17216	16114	59977
ENSMUSG00000024803.8	Ankrd1	7.70E-13	1.48E-10	4	6	132	10115.5	9983.5	5.571117006	6.38873178	Higher in TRIM24	148	88	121	171	341	13862	26196	7223	1266	11805
ENSMUSG00000078117.2	Gm16485	8.05E-13	1.54E-10	4	6	294.75	20	274.75	3.943818312	4.172945869	Higher in Normal	264	204	366	345	5	2	31	4	32	46
ENSMUSG00000035165.14	Kcne3	8.57E-13	1.63E-10	4	6	67.75	802.5	734.75	3.214013505	3.336752928	Higher in TRIM24	51	64	44	112	258	481	708	663	395	2310
ENSMUSG00000052951.5	C130021I20Rik	8.78E-13	1.66E-10	4	6	1198.5	58.66666667	1139.833333	4.21920486	4.506343261	Higher in Normal	780	901	1730	1383	19	12	45	23	201	52
ENSMUSG00000057135.12	Scimp	9.10E-13	1.71E-10	4	6	66	537	471	2.905919687	2.995526982	Higher in TRIM24	92	73	34	65	415	464	714	470	427	732
ENSMUSG00000020099.8	Unc5b	9.17E-13	1.71E-10	4	6	776	4207.166667	3431.166667	2.388946309	2.437449051	Higher in TRIM24	647	401	1071	985	2349	3486	4628	3760	2835	8185
ENSMUSG00000001985.9	Grik3	9.85E-13	1.81E-10	4	6	1069.5	31.66666667	1037.833333	4.731134511	5.15304138	Higher in Normal	165	545	2073	1495	18	18	33	2	74	45
ENSMUSG00000053550.13	Shisa7	9.88E-13	1.81E-10	4	6	187.25	4.166666667	183.0833333	5.058124351	5.593964486	Higher in Normal	54	53	311	331	0	1	5	1	11	7
ENSMUSG00000021708.17	Rasgrf2	1.06E-12	1.93E-10	4	6	1123.5	101	1022.5	3.375974117	3.518196444	Higher in Normal	1196	1465	1013	820	43	125	141	43	139	115
ENSMUSG00000022197.15	Pdzd2	1.32E-12	2.39E-10	4	6	3076	449.8333333	2626.166667	2.934394722	3.026719121	Higher in Normal	3262	3111	3612	2319	296	152	166	289	909	887
ENSMUSG00000061322.15	Dnaic1	1.55E-12	2.78E-10	4	6	107.75	13.5	94.25	3.044235029	3.14922717	Higher in Normal	99	127	78	127	8	4	20	14	9	26
ENSMUSG00000026656.15	Fcgr2b	1.59E-12	2.81E-10	4	6	582	12633.66667	12051.66667	3.910005464	4.148082944	Higher in TRIM24	614	1025	320	369	3986	10278	19425	5622	5726	30765
ENSMUSG00000020279.10	Il9r	1.59E-12	2.81E-10	4	6	101	10.66666667	90.33333333	3.345594725	3.485217814	Higher in Normal	50	108	128	118	6	2	11	3	19	23
ENSMUSG00000039154.6	Shd	1.64E-12	2.88E-10	4	6	56.5	6.333333333	50.16666667	3.161158427	3.279148804	Higher in Normal	52	31	74	69	5	4	4	1	12	12
ENSMUSG00000025473.16	Adam8	1.71E-12	2.99E-10	4	6	360.75	3737.5	3376.75	3.227855209	3.355839827	Higher in TRIM24	241	205	484	513	1013	4353	4062	1521	4622	6854
ENSMUSG00000047844.3	Bex4	2.01E-12	3.50E-10	4	6	107.5	1.5	106	5.411820246	6.153043435	Higher in Normal	68	15	155	192	1	0	5	0	1	2
ENSMUSG00000062070.12	Pgk1	2.33E-12	4.04E-10	4	6	3319.75	29574	26254.25	2.8038973	2.886938901	Higher in TRIM24	3217	2717	3054	4291	15218	8176	22187	28911	16353	86599
ENSMUSG00000057060.7	Slc35f3	2.42E-12	4.15E-10	4	6	820.25	13.5	806.75	5.15920096	5.755408739	Higher in Normal	145	262	1428	1446	4	1	27	22	19	8
ENSMUSG00000066438.6	Plekhd1	2.44E-12	4.15E-10	4	6	118	8.5	109.5	3.813577937	4.02985053	Higher in Normal	100	90	145	137	12	1	5	0	14	19

ENSMUSG00000021763.16	BC067074	2.48E-12	4.20E-10	4	6	297.75	36.33333333	261.4166667	3.01265394	3.115670336	Higher in Normal	237	231	462	261	14	15	48	23	76	42
ENSMUSG00000033174.17	Mgll	2.87E-12	4.83E-10	4	6	31817.75	2695.5	29122.25	3.617754725	3.802164606	Higher in Normal	46769	51955	11921	16626	1990	1036	2831	1743	5010	3563
ENSMUSG00000074813.12	Gm14005	3.07E-12	5.14E-10	4	6	124	1164.333333	1040.333333	2.994629534	3.098184218	Higher in TRIM24	136	145	89	126	426	594	1317	1478	832	2339
ENSMUSG00000004891.16	Nes	3.16E-12	5.26E-10	4	6	985.25	12688	11702.75	3.425838041	3.585057498	Higher in TRIM24	704	857	1117	1263	4724	6262	19141	13768	3616	28617
ENSMUSG00000085833.2	Gm13003	3.22E-12	5.33E-10	4	6	47.25	2.5	44.75	4.11634848	4.406566719	Higher in Normal	46	37	72	34	4	2	0	1	1	7
ENSMUSG00000022098.9	Bmp1	3.76E-12	6.19E-10	4	6	1882.75	17057.33333	15174.58333	3.054082063	3.165128495	Higher in TRIM24	1139	1721	2246	2425	4561	17846	19237	15787	12582	32331
ENSMUSG00000024215.13	Spdef	4.10E-12	6.72E-10	4	6	206.25	19.33333333	186.9166667	3.42814318	3.585624188	Higher in Normal	162	133	221	309	11	3	14	8	51	29
ENSMUSG00000015947.10	Fcgr1	4.35E-12	7.05E-10	4	6	150	2583.5	2433.5	3.763190994	3.983150301	Higher in TRIM24	141	313	74	72	1932	2195	2717	3057	1548	4052
ENSMUSG00000030340.16	Scnn1a	4.54E-12	7.31E-10	4	6	1154.25	86.66666667	1067.583333	3.573557527	3.754636521	Higher in Normal	232	740	1752	1893	81	37	86	38	176	102
ENSMUSG00000075707.5	Dio3	4.65E-12	7.46E-10	4	6	4	274.3333333	270.3333333	5.38993812	6.195972243	Higher in TRIM24	3	0	7	6	18	100	605	257	499	167
ENSMUSG00000037962.7	Rflna	5.51E-12	8.73E-10	4	6	41.25	349.1666667	307.9166667	3.025443541	3.135171496	Higher in TRIM24	61	18	32	54	330	282	582	236	233	432
ENSMUSG00000073680.2	Tmem88b	6.96E-12	1.09E-09	4	6	2347.5	80.5	2267	4.544640305	4.948575428	Higher in Normal	462	230	4185	4513	89	18	44	33	128	171
ENSMUSG00000086847.1	Tbx3os2	7.75E-12	1.20E-09	4	6	71.75	4.833333333	66.91666667	3.928624508	4.180695636	Higher in Normal	67	68	62	90	0	1	8	3	2	15
ENSMUSG00000024395.7	Lims2	8.35E-12	1.29E-09	4	6	1459	114.5	1344.5	3.729285237	3.942657215	Higher in Normal	1914	2378	676	868	57	59	114	37	274	146
ENSMUSG00000025479.9	Cyp2e1	8.69E-12	1.34E-09	4	6	4232.75	43.33333333	4189.416667	5.918004692	6.969703866	Higher in Normal	11080	5400	186	265	38	15	47	2	94	64
ENSMUSG00000074607.11	Tox2	9.30E-12	1.42E-09	4	6	699.5	40	659.5	3.858984879	4.098764477	Higher in Normal	379	493	896	1030	67	5	59	19	63	27
ENSMUSG00000095571.2	Ighv5-17	1.00E-11	1.52E-09	4	6	97.25	1.666666667	95.58333333	5.236420845	5.96074656	Higher in Normal	11	180	73	125	3	1	0	2	2	2
ENSMUSG00000059657.4	Stfa2l1	1.01E-11	1.52E-09	4	6	1.25	180.8333333	179.5833333	5.593638217	6.650882964	Higher in TRIM24	3	1	1	0	54	20	22	66	387	536
ENSMUSG00000040284.4	Gzmg	1.22E-11	1.84E-09	4	6	3	963.1666667	960.1666667	6.368280887	8.313067131	Higher in TRIM24	3	3	4	2	6	1207	1610	8	2408	540
ENSMUSG00000001739.14	Cldn15	1.25E-11	1.88E-09	4	6	773.5	90	683.5	3.310815242	3.459597222	Higher in Normal	759	1314	436	585	32	23	90	76	101	218
ENSMUSG00000101389.1	Ms4a4a	1.39E-11	2.06E-09	4	6	133.25	1923.5	1790.25	3.366944889	3.528252097	Higher in TRIM24	151	122	99	161	937	742	2625	943	462	5832
ENSMUSG00000036144.6	Meox2	1.63E-11	2.42E-09	4	6	1118.5	195	923.5	2.539778752	2.605559355	Higher in Normal	1158	1016	1013	1287	164	60	328	118	266	234
ENSMUSG00000029334.14	Prkg2	1.82E-11	2.68E-09	4	6	75.25	5574.833333	5499.583333	5.365212767	6.214162593	Higher in TRIM24	165	37	58	41	262	8610	2469	8008	634	13466
ENSMUSG00000037010.7	Apln	1.88E-11	2.75E-09	4	6	303.25	3065.666667	2762.416667	3.200660443	3.339581295	Higher in TRIM24	559	172	188	294	4027	2279	2891	2930	1261	5006
ENSMUSG00000030867.7	Plk1	2.01E-11	2.91E-09	4	6	181	1272.666667	1091.666667	2.784740489	2.874162042	Higher in TRIM24	157	58	234	275	1516	444	1389	1289	1344	1654
ENSMUSG00000056569.10	Mpz	2.02E-11	2.91E-09	4	6	1632.25	70.66666667	1561.583333	4.357178013	4.727361077	Higher in Normal	163	2344	1810	2212	44	21	75	18	184	82
ENSMUSG00000000223.13	Drp2	2.26E-11	3.24E-09	4	6	911.25	71.16666667	840.0833333	3.630147508	3.83514423	Higher in Normal	357	435	1521	1332	14	24	88	27	139	135
ENSMUSG00000015085.8	Entpd2	2.34E-11	3.35E-09	4	6	639.5	94	545.5	2.700800597	2.781797099	Higher in Normal	456	590	655	857	132	27	80	83	143	99
ENSMUSG00000052062.14	Pard3b	2.37E-11	3.37E-09	4	6	1013.5	154.6666667	858.8333333	2.683612468	2.762981867	Higher in Normal	578	645	1603	1228	53	80	188	113	271	223
ENSMUSG00000024140.9	Epas1	2.44E-11	3.45E-09	4	6	15459.5	2889	12570.5	2.39890511	2.455063767	Higher in Normal	11566	10708	19172	20392	1709	2120	2916	1298	5835	3456
ENSMUSG00000040147.14	Maob	2.44E-11	3.45E-09	4	6	1390.25	162.5	1227.75	3.119029591	3.246188356	Higher in Normal	1439	1378	1238	1506	114	24	153	133	354	197
ENSMUSG00000040170.13	Fmo2	2.47E-11	3.47E-09	4	6	3753.5	178.1666667	3575.333333	4.226631905	4.564546967	Higher in Normal	1775	1762	5946	5531	198	38	73	17	456	287
ENSMUSG00000033308.16	Dpyd	2.49E-11	3.48E-09	4	6	868.75	43	825.75	4.154991314	4.474623234	Higher in Normal	629	586	1155	1105	45	10	39	2	115	47
ENSMUSG00000022651.5	Retnlg	2.93E-11	4.08E-09	4	6	8	258.6666667	250.6666667	4.37035045	4.778101587	Higher in TRIM24	20	3	5	4	68	46	233	186	626	393
ENSMUSG00000109305.1	1810010D01Rik	3.04E-11	4.21E-09	4	6	318.25	14.66666667	303.5833333	4.350476929	4.727581843	Higher in Normal	33	389	360	491	4	7	23	4	8	42
ENSMUSG00000085183.1	Wincr1	3.22E-11	4.43E-09	4	6	5	883.3333333	878.3333333	5.816878895	7.111621844	Higher in TRIM24	7	10	0	3	3	658	692	685	1102	2160
ENSMUSG00000045392.8	Olfr1033	3.68E-11	5.03E-09	4	6	139	13.16666667	125.8333333	3.447629449	3.625823993	Higher in Normal	78	94	200	184	5	12	3	3	18	38
ENSMUSG00000005540.10	Fcer2a	3.71E-11	5.05E-09	4	6	62	5.833333333	56.16666667	3.289309994	3.445905701	Higher in Normal	25	48	90	85	4	7	3	3	8	10
ENSMUSG00000020407.13	Upp1	3.75E-11	5.07E-09	4	6	578.75	5042.833333	4464.083333	2.787611076	2.880029401	Higher in TRIM24	264	257	749	1045	3526	1952	3739	2052	3724	15264
ENSMUSG00000031883.13	Car7	3.95E-11	5.30E-09	4	6	205.5	28.33333333	177.1666667	2.934244399	3.04174432	Higher in Normal	358	163	125	176	13	11	31	19	51	45
ENSMUSG00000024366.7	Gfra3	4.07E-11	5.43E-09	4	6	67.75	4.166666667	63.58333333	3.840219679	4.098238464	Higher in Normal	53	53	84	81	1	2	3	2	14	3
ENSMUSG00000033948.3	Zswim5	4.30E-11	5.71E-09	4	6	285.5	20	265.5	3.677423479	3.898321371	Higher in Normal	136	56	522	428	10	6	14	13	43	34
ENSMUSG00000024989.14	Cep55	4.87E-11	6.44E-09	4	6	208.75	1207.166667	998.4166667	2.44310864	2.504931865	Higher in TRIM24	143	56	331	305	996	728	1145	688	1412	2274
ENSMUSG00000020193.3	Zpbp	5.11E-11	6.74E-09	4	6	53.25	11.16666667	42.08333333	2.327146955	2.380147198	Higher in Normal	59	32	65	57	6	8	9	6	15	23
ENSMUSG00000022853.6	Ehhadh	5.23E-11	6.86E-09	4	6	1311.5	223.8333333	1087.666667	2.454310622	2.516784268	Higher in Normal	1041	748	1600	1857	317	136	252	93	298	247
ENSMUSG00000078532.9	Nkain1	5.28E-11	6.91E-09	4	6	76.75	846.6666667	769.9166667	3.334276763	3.5013753	Higher in TRIM24	132	63	55	57	359	1007	1226	856	481	1151
ENSMUSG00000032322.14	Pstpip1	5.31E-11	6.91E-09	4	6	269.25	1868.833333	1599.583333	2.572327096	2.645389256	Higher in TRIM24	269	337	185	286	1003	965	1662	2312	1307	3964
ENSMUSG00000104213.5	Ighd	5.88E-11	7.61E-09	4	6	232.5	16	216.5	3.635333446	3.85390767	Higher in Normal	103	383	199	245	6	18	23	14	24	11
ENSMUSG00000059901.12	Adamts14	5.92E-11	7.63E-09	4	6	128	2371	2243	3.850132497	4.120517632	Higher in TRIM24	155	162	120	75	559	3999	2044	1149	1093	5382
ENSMUSG00000041757.16	Plekha6	6.01E-11	7.72E-09	4	6	4226.25	410.6666667	3815.583333	3.238182192	3.387706964	Higher in Normal	2419	2271	6588	5627	394	96	246	313	1008	407
ENSMUSG00000044092.8	C130050O18Rik	6.20E-11	7.90E-09	4	6	34	249.5	215.5	2.809502313	2.906925215	Higher in TRIM24	29	32	40	35	141	315	361	133	192	355
ENSMUSG00000055733.6	Nap1l3	6.24E-11	7.92E-09	4	6	179.5	12.33333333	167.1666667	3.665830802	3.889785508	Higher in Normal	148	105	226	239	6	8	21	1	28	10
ENSMUSG00000059256.6	Gzmd	6.30E-11	7.96E-09	4	6	9.25	1130.833333	1121.583333	5.691149854	6.903466484	Higher in TRIM24	25	0	5	7	178	1013	1880	66	3138	510
ENSMUSG00000020808.3	Pimreg	7.02E-11	8.80E-09	4	6	114.75	928.8333333	814.0833333	2.849253894	2.951333529	Higher in TRIM24	93	37	135	194	398	406	954	1032	647	2136
ENSMUSG00000056069.8	Otulinl	7.67E-11	9.56E-09	4	6	352	2140	1788	2.490019118	2.557193311	Higher in TRIM24	351	422	295	340	1298	1820	2905	2045	1282	3490
ENSMUSG00000094872.1	Igkv9-120	7.94E-11	9.82E-09	4	6	156.25	4.333333333	151.9166667	4.729905028	5.270236608	Higher in Normal	21	276	139	189	9	1	8	1	2	5
ENSMUSG00000020627.10	Klhl29	8.34E-11	1.03E-08	4	6	993.25	63.33333333	929.9166667	3.655095044	3.879352512	Higher in Normal	327	237	1859	1550	19	37	85	62	99	78
ENSMUSG00000074934.3	Grem1	8.43E-11	1.04E-08	4	6	41.75	4240.166667	4198.416667	5.544870155	6.636064342	Higher in TRIM24	49	13	65	40	34	8384	3300	599	1007	12117
ENSMUSG00000023826.15	Prkn	8.77E-11	1.07E-08	4	6	340.5	39.5	301	3.309141213	3.47177052	Higher in Normal	414	199	350	399	15	14	10	11	76	111
ENSMUSG00000079575.3	Rbpj-ps3	9.58E-11	1.17E-08	4	6	23.5	371.3333333	347.8333333	3.68314537	3.922456904	Higher in TRIM24	44	4	17	29	480	315	369	80	257	727
ENSMUSG00000037735.7	2810032G03Rik	1.14E-10	1.37E-08	4	6	88.75	16.5	72.25	2.600651633	2.678053615	Higher in Normal	106	96	71	82	7	6	12	10	29	35
ENSMUSG00000030317.8	Timp4	1.20E-10	1.44E-08	4	6	5810.25	93.66666667	5716.583333	5.532353058	6.48819073	Higher in Normal	6972	14037	1046	1186	38	14	14	8	370	118
ENSMUSG00000026480.12	Ncf2	1.21E-10	1.44E-08	4	6	480	3245.833333	2765.833333	2.619709846	2.700251175	Higher in TRIM24	533	563	348	476	1658	2559	5142	2961	2121	5034
ENSMUSG00000073802.4	Cdkn2b	1.43E-10	1.69E-08	4	6	204.75	3781.333333	3576.583333	3.772916433	4.037796646	Higher in TRIM24	345	272	98	104	1191	4377	3861	3144	1276	8839
ENSMUSG00000021604.15	Irx4	1.45E-10	1.71E-08	4	6	917	21	896	4.890663816	5.505074364	Higher in Normal	249	342	1401	1676	15	4	14	1	81	11
ENSMUSG00000060882.5	Kcnd2	1.48E-10	1.74E-08	4	6	520	7.833333333	512.1666667	5.530854737	6.499497002	Higher in Normal	116	180	958	826	0	0	6	0	14	27
ENSMUSG00000021469.9	Msx2	1.62E-10	1.90E-08	4	6	315.5	23.16666667	292.3333333	3.487871731	3.689102853	Higher in Normal	271	432	255	304	10	27	46	24	17	15
ENSMUSG00000040026.8	Saa3	1.71E-10	2.00E-08	4	6	61.5	2457.333333	2395.833333	4.605607634	5.146296706	Higher in TRIM24	83	39	60	64	266	520	4525	320	7275	1838
ENSMUSG00000033910.13	Gucy1a1	1.77E-10	2.06E-08	4	6	996	148	848	2.741226013	2.834749168	Higher in Normal	970	984	1050	980	160	72	148	50	326	132
ENSMUSG00000029553.10	Tfec	1.85E-10	2.15E-08	4	6	52.25	534	481.75	3.223719113	3.383594607	Higher in TRIM24	62	37	52	58	188	828	640	285	286	977
ENSMUSG00000042759.12	Apobr	1.87E-10	2.15E-08	4	6	534.75	2946.166667	2411.416667	2.342652533	2.400668406	Higher in TRIM24	531	369	569	670	1737	3390	2909	1226	2135	6280
ENSMUSG00000079186.2	Gzmc	1.99E-10	2.28E-08	4	6	25	982.1666667	957.1666667	4.64202685	5.205859011	Higher in TRIM24	51	16	22	11	46	490	2415	458	1692	792
ENSMUSG00000022382.14	Wnt7b	2.05E-10	2.34E-08	4	6	764.25	10485.5	9721.25	3.656191905	3.899214431	Higher in TRIM24	277	534	1038	1208	6852	18063	11528	4113	3440	18917
ENSMUSG00000038599.14	Capn8	2.06E-10	2.34E-08	4	6	178.75	16	162.75	3.607918858	3.831351911	Higher in Normal	195	64	307	149	7	2	10	3	27	47
ENSMUSG00000022419.15	Deptor	2.09E-10	2.38E-08	4	6	7407	1681.666667	5725.333333	2.343830004	2.401797281	Higher in Normal	6724	7827	7946	7131	1413	748	1185	423	2813	3508
ENSMUSG00000020798.14	Spns3	2.11E-10	2.39E-08	4	6	808.25	83.16666667	725.0833333	3.085416634	3.222455391	Higher in Normal	373	540	1033	1287	33	77	100	45	174	70
ENSMUSG00000028565.18	Nfia	2.19E-10	2.47E-08	4	6	5718.75	940.8333333	4777.916667	2.648594637	2.733498345	Higher in Normal	5341	6991	4622	5921	1228	267	843	534	1677	1096
ENSMUSG00000037016.11	Frem2	2.44E-10	2.72E-08	4	6	302.5	20.66666667	281.8333333	3.837493973	4.114626393	Higher in Normal	185	42	607	376	10	4	9	9	34	58
ENSMUSG00000074300.4	BC030870	2.53E-10	2.81E-08	4	6	98.25	3	95.25	4.688032422	5.242111699	Higher in Normal	44	16	155	178	0	0	4	2	2	10
ENSMUSG00000040046.14	Tph1	2.59E-10	2.86E-08	4	6	5701.25	79.16666667	5622.083333	5.331479293	6.205277377	Higher in Normal	396	168	10897	11344	44	11	45	24	274	77
ENSMUSG00000035283.4	Adrb1	2.66E-10	2.93E-08	4	6	539.5	49.33333333	490.1666667	3.27322723	3.440484207	Higher in Normal	412	306	707	733	78	15	44	17	108	34
ENSMUSG00000019942.13	Cdk1	2.79E-10	3.07E-08	4	6	557	4666.166667	4109.166667	2.849673322	2.959390616	Higher in TRIM24	347	127	726	1028	4578	1712	2932	3169	3942	11664

ENSMUSG00000062329.4	Cytl1	2.81E-10	3.07E-08	4	6	547.5	10	537.5	5.059693458	5.787247022	Higher in Normal	177	333	666	1014	12	1	12	0	33	2
ENSMUSG00000072553.10	Gm525	3.17E-10	3.42E-08	4	6	162	9	153	4.036285831	4.371447793	Higher in Normal	232	184	95	137	8	0	22	2	10	12
ENSMUSG00000045775.15	Slc16a5	3.30E-10	3.55E-08	4	6	372.25	19.33333333	352.9166667	4.065376188	4.408233078	Higher in Normal	203	39	598	649	9	16	18	2	15	56
ENSMUSG00000039476.13	Prrx2	3.35E-10	3.59E-08	4	6	116.75	1342	1225.25	3.075313388	3.216980251	Higher in TRIM24	172	117	76	102	1464	283	1188	895	521	3701
ENSMUSG00000059742.10	Kcnh7	3.52E-10	3.77E-08	4	6	159	27.33333333	131.6666667	2.71355999	2.807037873	Higher in Normal	140	149	152	195	9	12	27	6	50	60
ENSMUSG00000072966.11	Gprasp2	3.58E-10	3.79E-08	4	6	440.5	20	420.5	4.103770359	4.460201273	Higher in Normal	166	354	582	660	4	1	24	37	27	27
ENSMUSG00000064247.14	Plcxd1	3.58E-10	3.79E-08	4	6	125	19.33333333	105.6666667	2.699549111	2.791925037	Higher in Normal	91	51	157	201	10	11	9	12	36	38
ENSMUSG00000041423.16	Paqr6	3.60E-10	3.79E-08	4	6	554	41.66666667	512.3333333	3.719226394	3.975355301	Higher in Normal	268	591	588	769	22	9	38	5	118	58
ENSMUSG00000071005.7	Ccl19	3.62E-10	3.80E-08	4	6	101.75	2	99.75	4.967120386	5.686564956	Higher in Normal	6	46	119	236	0	1	3	1	4	3
ENSMUSG00000031870.16	Pgr	3.72E-10	3.90E-08	4	6	1283	90.66666667	1192.333333	3.747734832	4.010976301	Higher in Normal	670	569	2320	1573	29	7	166	38	112	192
ENSMUSG00000108624.1	Gm45091	3.83E-10	3.99E-08	4	6	104.5	15.5	89	2.741343699	2.839293863	Higher in Normal	95	121	85	117	10	7	21	10	31	14
ENSMUSG00000053964.17	Lgals4	3.85E-10	4.00E-08	4	6	898.25	102	796.25	3.240865035	3.406078367	Higher in Normal	680	1396	744	773	111	51	23	37	202	188
ENSMUSG00000052336.7	Cx3cr1	3.92E-10	4.06E-08	4	6	309.25	4141	3831.75	3.530637898	3.755155569	Higher in TRIM24	403	472	156	206	2846	3495	5246	6670	1569	5020
ENSMUSG00000026343.6	Gpr39	4.00E-10	4.13E-08	4	6	3.25	138.3333333	135.0833333	4.971749533	5.763731948	Higher in TRIM24	7	1	2	3	45	420	140	79	78	68
ENSMUSG00000026600.12	Soat1	4.03E-10	4.14E-08	4	6	1241.5	6691.333333	5449.833333	2.323060097	2.381868384	Higher in TRIM24	787	771	1718	1690	3675	4946	8020	5475	3327	14705
ENSMUSG00000044628.4	Rnf208	4.25E-10	4.36E-08	4	6	533.25	43.83333333	489.4166667	3.37181913	3.560692826	Higher in Normal	201	338	640	954	52	31	32	12	103	33
ENSMUSG00000030554.16	Synm	4.78E-10	4.86E-08	4	6	3861.75	346.3333333	3515.416667	3.306225432	3.48437138	Higher in Normal	1357	1434	6585	6071	191	153	642	109	535	448
ENSMUSG00000105300.1	Gm30613	4.88E-10	4.96E-08	4	6	80	1.166666667	78.83333333	5.436179636	6.459301996	Higher in Normal	18	23	145	134	0	0	1	0	0	6
ENSMUSG00000034245.10	Hdac11	4.92E-10	4.98E-08	4	6	1430	235.8333333	1194.166667	2.616165493	2.701411191	Higher in Normal	823	1050	1712	2135	359	66	160	100	331	399
ENSMUSG00000056457.6	Prl2c3	5.09E-10	5.11E-08	4	6	35.5	7437.166667	7401.666667	5.700524374	7.164568347	Higher in TRIM24	98	16	11	17	808	235	6422	43	18794	18321
ENSMUSG00000028194.15	Ddah1	5.13E-10	5.14E-08	4	6	171.5	2021.5	1850	3.315388923	3.500653829	Higher in TRIM24	225	219	106	136	662	1860	4304	963	2326	2014
ENSMUSG00000048905.4	4930539E08Rik	5.17E-10	5.16E-08	4	6	444.25	26	418.25	3.823687517	4.110806576	Higher in Normal	139	85	791	762	43	7	13	9	39	45
ENSMUSG00000029762.6	Akr1b8	5.18E-10	5.16E-08	4	6	702.5	5013.166667	4310.666667	2.534640662	2.612959682	Higher in TRIM24	555	757	626	872	1842	2362	7592	3249	2825	12209
ENSMUSG00000003545.3	Fosb	5.32E-10	5.28E-08	4	6	73760.75	463.1666667	73297.58333	5.944638296	7.393662422	Higher in Normal	711	85	144009	150238	89	437	345	95	525	1288
ENSMUSG00000057614.6	Gnai1	5.42E-10	5.36E-08	4	6	3663	242.6666667	3420.333333	3.790358096	4.069715805	Higher in Normal	6371	6069	1086	1126	175	190	320	121	438	212
ENSMUSG00000046782.14	Ttc6	5.90E-10	5.81E-08	4	6	607.25	17.83333333	589.4166667	4.690590641	5.270374384	Higher in Normal	318	526	991	594	47	1	3	1	17	38
ENSMUSG00000004043.14	Stat5a	5.98E-10	5.87E-08	4	6	5534.25	1069.333333	4464.916667	2.379065913	2.443183826	Higher in Normal	5068	2420	7318	7331	1243	369	1040	421	1762	1581
ENSMUSG00000071178.11	Serpina1b	6.03E-10	5.90E-08	4	6	747	76.83333333	670.1666667	3.232087047	3.399993304	Higher in Normal	1386	441	470	691	52	22	98	39	182	68
ENSMUSG00000046093.9	Hpcal4	6.10E-10	5.95E-08	4	6	342	5.5	336.5	5.138586131	5.956622395	Higher in Normal	43	23	658	644	2	3	5	0	17	6
ENSMUSG00000012705.16	Retn	6.52E-10	6.34E-08	4	6	10545	98.83333333	10446.16667	5.792723048	7.106322177	Higher in Normal	22417	19533	100	130	118	22	98	13	219	123
ENSMUSG00000036777.8	Anln	6.53E-10	6.34E-08	4	6	606	5005.166667	4399.166667	2.773161491	2.878548302	Higher in TRIM24	383	215	1037	789	2015	1781	7053	2779	3581	12822
ENSMUSG00000048644.8	Ctxn1	6.64E-10	6.42E-08	4	6	200.25	1827.666667	1627.416667	3.130203786	3.28587332	Higher in TRIM24	142	75	225	359	1252	922	3727	1837	794	2434
ENSMUSG00000024053.10	Emilin2	6.67E-10	6.42E-08	4	6	718.25	5906.166667	5187.916667	2.674940967	2.769070471	Higher in TRIM24	724	914	626	609	1634	3286	8229	3654	3921	14713
ENSMUSG00000083494.1	Gm12873	6.74E-10	6.47E-08	4	6	523.25	9.833333333	513.4166667	4.977943445	5.709183037	Higher in Normal	95	878	444	676	2	0	7	27	16	7
ENSMUSG00000040675.17	Mthfd1l	6.89E-10	6.60E-08	4	6	380.75	2413.666667	2032.916667	2.490690449	2.56594138	Higher in TRIM24	282	145	522	574	1092	1122	3347	1768	1576	5577
ENSMUSG00000045362.8	Tnfrsf26	6.98E-10	6.66E-08	4	6	85.5	594.6666667	509.1666667	2.661545065	2.754460435	Higher in TRIM24	77	65	97	103	138	525	820	358	840	887
ENSMUSG00000026424.8	Gpr37l1	7.36E-10	7.00E-08	4	6	46.25	4.166666667	42.08333333	3.350437258	3.54604324	Higher in Normal	78	27	49	31	2	3	3	4	8	5
ENSMUSG00000022803.12	Popdc2	7.71E-10	7.31E-08	4	6	402.75	38	364.75	3.349963911	3.54086965	Higher in Normal	316	327	418	550	8	14	72	10	77	47
ENSMUSG00000084772.1	Gm15473	8.16E-10	7.72E-08	4	6	5	384.1666667	379.1666667	5.05641107	5.946515941	Higher in TRIM24	6	6	4	4	463	10	529	228	12	1063
ENSMUSG00000050914.16	Ankrd37	8.45E-10	7.94E-08	4	6	37.25	312.5	275.25	2.731845245	2.834108301	Higher in TRIM24	33	43	34	39	153	65	201	289	562	605
ENSMUSG00000037813.13	D630003M21Rik	8.47E-10	7.94E-08	4	6	230.5	39.66666667	190.8333333	2.522180699	2.600458899	Higher in Normal	199	95	294	334	38	10	32	26	75	57
ENSMUSG00000100502.1	Gm28286	8.75E-10	8.18E-08	4	6	50.25	5.166666667	45.08333333	3.344154904	3.536572056	Higher in Normal	40	72	43	46	5	2	2	1	11	10
ENSMUSG00000012282.2	Wnt8a	8.80E-10	8.19E-08	4	6	162.25	1.833333333	160.4166667	5.30186909	6.290963871	Higher in Normal	45	33	282	289	5	1	0	0	5	0
ENSMUSG00000035914.11	Cd276	8.99E-10	8.34E-08	4	6	754.75	4565.166667	3810.416667	2.584625242	2.670446754	Higher in TRIM24	354	278	1090	1297	5761	2662	4406	3838	3303	7421
ENSMUSG00000022546.4	Gpt	9.05E-10	8.36E-08	4	6	3151.5	404.1666667	2747.333333	3.098281239	3.248412	Higher in Normal	4782	4849	1274	1701	251	162	270	284	777	681
ENSMUSG00000015476.6	Prrt1	9.06E-10	8.36E-08	4	6	262.5	36	226.5	2.939911191	3.06734299	Higher in Normal	181	486	187	196	32	19	32	18	70	45
ENSMUSG00000021190.14	Lgmn	9.87E-10	9.06E-08	4	6	4019	25394	21375	2.441896745	2.514057328	Higher in TRIM24	2199	3729	4338	5810	16409	10335	26013	25823	11645	62139
ENSMUSG00000091956.2	C2cd4b	9.87E-10	9.06E-08	4	6	1213.75	5	1208.75	6.009714912	7.659186881	Higher in Normal	8	12	1889	2946	15	1	2	2	9	1
ENSMUSG00000005952.15	Trpv1	9.98E-10	9.13E-08	4	6	129.5	3.333333333	126.1666667	4.824192271	5.503672504	Higher in Normal	306	133	48	31	3	1	0	2	11	3
ENSMUSG00000095788.6	Sirpb1a	1.08E-09	9.83E-08	4	6	48.75	314.1666667	265.4166667	2.488584245	2.565905847	Higher in TRIM24	38	73	29	55	184	296	368	202	251	584
ENSMUSG00000044156.14	Hepacam2	1.11E-09	1.01E-07	4	6	1240.5	57	1183.5	4.040196164	4.400719355	Higher in Normal	520	495	1822	2125	37	9	175	15	60	46
ENSMUSG00000032374.14	Plod2	1.23E-09	1.11E-07	4	6	821.75	7146.5	6324.75	2.721234377	2.824097033	Higher in TRIM24	640	650	1040	957	3512	2795	5588	5579	2035	23370
ENSMUSG00000016200.14	Syt14	1.27E-09	1.15E-07	4	6	127.75	9.666666667	118.0833333	3.829116687	4.130946434	Higher in Normal	191	82	134	104	1	0	12	3	13	29
ENSMUSG00000052353.13	Cemip	1.30E-09	1.17E-07	4	6	48.5	780	731.5	3.583741008	3.837483996	Higher in TRIM24	83	16	54	41	391	104	436	603	2086	1060
ENSMUSG00000041577.5	Prelp	1.33E-09	1.19E-07	4	6	10997	865.8333333	10131.16667	3.603197053	3.852736858	Higher in Normal	8939	16152	8844	10053	639	174	862	344	2685	491
ENSMUSG00000076569.3	Igkv5-39	1.34E-09	1.20E-07	4	6	310.75	4.333333333	306.4166667	5.313886796	6.312302466	Higher in Normal	9	788	193	253	3	2	13	3	0	5
ENSMUSG00000029120.10	Ppp2r2c	1.42E-09	1.27E-07	4	6	75.75	7	68.75	3.435382984	3.649904937	Higher in Normal	35	51	115	102	10	2	1	2	7	20
ENSMUSG00000004655.5	Aqp1	1.48E-09	1.31E-07	4	6	32607.75	5370.833333	27236.91667	2.614824107	2.705285754	Higher in Normal	18959	15683	43037	52752	2264	3111	8562	1730	5677	10881
ENSMUSG00000100627.6	A830008E24Rik	1.49E-09	1.32E-07	4	6	148.5	20.66666667	127.8333333	3.132115133	3.291006313	Higher in Normal	152	191	121	130	8	6	8	5	37	60
ENSMUSG00000021379.1	Id4	1.50E-09	1.32E-07	4	6	1376.75	50.66666667	1326.083333	4.23040307	4.659914832	Higher in Normal	406	422	1919	2760	90	5	94	13	71	31
ENSMUSG00000023387.7	Kcnk16	1.57E-09	1.37E-07	4	6	99.5	0.833333333	98.66666667	5.405652418	6.617067957	Higher in Normal	70	4	166	158	1	2	2	0	0	0
ENSMUSG00000062515.3	Fabp4	1.61E-09	1.41E-07	4	6	220556.5	8196.833333	212359.6667	4.527961212	5.073038706	Higher in Normal	342467	446845	37571	55343	11972	1123	2848	3710	20644	8884
ENSMUSG00000071068.6	Treml2	1.67E-09	1.45E-07	4	6	29	191.1666667	162.1666667	2.565613471	2.653060942	Higher in TRIM24	32	38	22	24	103	136	135	260	204	309
ENSMUSG00000035352.3	Ccl12	1.75E-09	1.51E-07	4	6	86.5	1337.166667	1250.666667	3.557705592	3.810187449	Higher in TRIM24	66	163	48	69	452	658	1005	2525	734	2649
ENSMUSG00000035493.9	Tgfbi	1.95E-09	1.68E-07	4	6	2374	15714.16667	13340.16667	2.605663446	2.697730989	Higher in TRIM24	1726	2642	2295	2833	5110	9832	23185	19510	12713	23935
ENSMUSG00000030703.8	Gdpd3	1.97E-09	1.69E-07	4	6	63.25	915.5	852.25	3.658273204	3.937958763	Higher in TRIM24	82	85	51	35	1645	549	920	1020	1148	211
ENSMUSG00000040412.16	5330417C22Rik	2.29E-09	1.95E-07	4	6	1853	92.5	1760.5	4.076644889	4.46536741	Higher in Normal	671	615	3105	3021	99	9	31	18	262	136
ENSMUSG00000026249.10	Serpine2	2.31E-09	1.96E-07	4	6	1670.75	18994.5	17323.75	3.400762281	3.621163163	Higher in TRIM24	1132	2129	1405	2017	40710	13794	28709	11437	9470	9847
ENSMUSG00000066319.6	Rtp3	2.36E-09	1.99E-07	4	6	111	16.83333333	94.16666667	2.829852105	2.949370122	Higher in Normal	150	138	74	82	4	8	15	15	27	32
ENSMUSG00000051879.3	Krt71	2.37E-09	1.99E-07	4	6	207.25	4.166666667	203.0833333	4.865075883	5.606334651	Higher in Normal	33	24	320	452	11	0	3	1	2	8
ENSMUSG00000040855.15	Reps2	2.45E-09	2.06E-07	4	6	872.75	158.1666667	714.5833333	2.413443604	2.485954723	Higher in Normal	491	494	1316	1190	131	59	204	83	293	179
ENSMUSG00000085747.1	Slc13a2os	2.65E-09	2.23E-07	4	6	31.5	1.333333333	30.16666667	4.275946548	4.760919079	Higher in Normal	27	31	44	24	1	0	0	0	5	2
ENSMUSG00000067261.4	Foxd3	2.71E-09	2.26E-07	4	6	28.25	1.333333333	26.91666667	4.178090422	4.620019837	Higher in Normal	14	22	44	33	0	0	2	0	2	4
ENSMUSG00000029484.12	Anxa3	2.77E-09	2.31E-07	4	6	1777	12700	10923	2.768458155	2.882452022	Higher in TRIM24	1427	1912	1610	2159	16954	14402	9410	10550	4254	20630
ENSMUSG00000063297.7	Luzp2	2.84E-09	2.36E-07	4	6	399	14	385	4.515545863	5.076663963	Higher in Normal	107	61	771	657	2	2	7	2	40	31
ENSMUSG00000030545.9	Pex11a	3.02E-09	2.51E-07	4	6	1914.25	351.5	1562.75	2.46251044	2.540668662	Higher in Normal	2006	1393	1905	2353	422	108	176	221	738	444
ENSMUSG00000015224.10	Cyp2j9	3.04E-09	2.52E-07	4	6	306.5	31.66666667	274.8333333	3.287480649	3.482401547	Higher in Normal	310	327	306	283	7	11	40	9	87	36
ENSMUSG00000032878.16	Ccdc85a	3.07E-09	2.53E-07	4	6	386.25	61.5	324.75	2.755643501	2.86682172	Higher in Normal	532	362	357	294	31	23	62	20	149	84
ENSMUSG00000022206.6	Npr3	3.16E-09	2.60E-07	4	6	9394.5	224	9170.5	5.054535998	5.910647965	Higher in Normal	18224	18328	518	508	242	42	87	27	459	487
ENSMUSG00000022949.9	Clic6	3.26E-09	2.66E-07	4	6	4272	179.1666667	4092.833333	4.256152073	4.71722403	Higher in Normal	1185	2227	6547	7129	30	47	125	29	686	158
ENSMUSG00000094840.5	Muc3a	3.26E-09	2.66E-07	4	6	48.25	1.666666667	46.58333333	4.482393645	5.064461294	Higher in Normal	56	76	24	37	0	1	2	0	6	1
ENSMUSG00000096965.7	3300005D01Rik	3.27E-09	2.66E-07	4	6	11.25	1057.166667	1045.916667	4.9293614	5.816022018	Higher in TRIM24	7	18	11	9	12	322	670	301	94	4944

ENSMUSG00000021250.13	Fos	3.27E-09	2.66E-07	4	6	111375	1960.333333	109414.6667	4.997312801	5.819013341	Higher in Normal	1785	1699	198923	243093	1605	657	1249	1555	2661	4035
ENSMUSG00000052485.7	Tmem171	3.47E-09	2.82E-07	4	6	9.5	280.3333333	270.8333333	4.386637285	4.945952257	Higher in TRIM24	11	6	15	6	14	593	203	146	122	604
ENSMUSG00000032572.9	Col6a4	3.62E-09	2.93E-07	4	6	550.5	37.66666667	512.8333333	3.781907585	4.093090466	Higher in Normal	329	155	984	734	4	6	60	11	57	88
ENSMUSG00000034675.17	Dbn1	3.65E-09	2.94E-07	4	6	827.25	6777	5949.75	2.865351791	2.994800545	Higher in TRIM24	420	704	1016	1169	3460	5077	11726	4919	1990	13490
ENSMUSG00000025330.6	Padi4	3.65E-09	2.94E-07	4	6	109.25	2355.666667	2246.416667	4.179193423	4.6442634	Higher in TRIM24	162	35	98	142	1235	5491	875	342	4530	1661
ENSMUSG00000113769.1	5033406O09Rik	3.96E-09	3.17E-07	4	6	30	209.1666667	179.1666667	2.649227772	2.750894857	Higher in TRIM24	19	36	36	29	80	165	215	264	133	398
ENSMUSG00000026271.15	Gpr35	4.09E-09	3.26E-07	4	6	209	1339.666667	1130.666667	2.592275657	2.686924057	Higher in TRIM24	307	61	250	218	920	1427	1595	520	1423	2153
ENSMUSG00000030865.4	Chp2	4.13E-09	3.28E-07	4	6	761	80.66666667	680.3333333	3.283796714	3.481640235	Higher in Normal	631	667	869	877	20	9	91	37	202	125
ENSMUSG00000009687.14	Fxyd5	4.27E-09	3.38E-07	4	6	1276.25	9550.166667	8273.916667	2.761115059	2.877175755	Higher in TRIM24	970	1827	955	1353	3239	10321	11744	10224	7056	14717
ENSMUSG00000105541.1	Gm43136	4.62E-09	3.63E-07	4	6	82.75	15.33333333	67.41666667	2.396995039	2.470964353	Higher in Normal	37	59	103	132	17	6	18	10	17	24
ENSMUSG00000046807.10	Lrrc75b	4.65E-09	3.64E-07	4	6	1869	143.3333333	1725.666667	3.658267453	3.941803074	Higher in Normal	1114	833	2785	2744	37	20	79	42	444	238
ENSMUSG00000012889.8	Podnl1	4.73E-09	3.69E-07	4	6	80.25	2930.166667	2849.916667	4.397064883	4.970603309	Higher in TRIM24	81	121	57	62	73	1365	2771	4941	676	7755
ENSMUSG00000030495.12	Slc7a10	4.98E-09	3.87E-07	4	6	1389.5	15.66666667	1373.833333	5.552156307	6.867590925	Higher in Normal	2429	3064	29	36	12	3	11	1	57	10
ENSMUSG00000026582.6	Sele	5.00E-09	3.87E-07	4	6	4361	35.83333333	4325.166667	5.581538572	6.925684714	Higher in Normal	68	34	9210	8132	4	11	68	3	92	37
ENSMUSG00000032315.6	Cyp1a1	5.08E-09	3.92E-07	4	6	32.75	2	30.75	3.900045889	4.259232218	Higher in Normal	41	14	43	33	0	1	0	1	4	6
ENSMUSG00000005611.15	Mrvi1	5.10E-09	3.93E-07	4	6	1120.25	115.3333333	1004.916667	3.106086391	3.27390296	Higher in Normal	396	387	1914	1784	60	39	181	60	222	130
ENSMUSG00000049410.8	Zfp683	5.25E-09	4.03E-07	4	6	57	3	54	4.053438503	4.459794866	Higher in Normal	58	13	92	65	3	0	0	1	6	8
ENSMUSG00000028528.16	Dnajc6	5.51E-09	4.20E-07	4	6	663.25	42.16666667	621.0833333	3.801223558	4.127095875	Higher in Normal	309	167	1210	967	9	28	21	5	106	84
ENSMUSG00000085651.1	Gm11695	5.70E-09	4.33E-07	4	6	43.5	0.166666667	43.33333333	5.681238368	7.250047389	Higher in Normal	10	6	84	74	0	0	0	0	1	0
ENSMUSG00000049107.13	Ntf3	5.94E-09	4.50E-07	4	6	90	9.666666667	80.33333333	3.227032424	3.419693279	Higher in Normal	80	141	77	62	7	3	19	2	15	12
ENSMUSG00000019856.14	Fam184a	5.96E-09	4.50E-07	4	6	141.25	19.83333333	121.4166667	3.084363907	3.248885155	Higher in Normal	208	113	144	100	4	3	20	7	25	60
ENSMUSG00000031636.7	Pdlim3	6.18E-09	4.66E-07	4	6	15892.75	541.3333333	15351.41667	4.291747898	4.788133696	Higher in Normal	4862	5301	23354	30054	580	155	1280	32	965	236
ENSMUSG00000021903.11	Galnt15	6.26E-09	4.71E-07	4	6	3612.75	418.5	3194.25	3.343764389	3.559291336	Higher in Normal	3001	4410	3608	3432	70	98	353	74	848	1068
ENSMUSG00000021573.15	Tppp	6.66E-09	4.98E-07	4	6	705.25	67.33333333	637.9166667	3.279102207	3.482305018	Higher in Normal	614	425	951	831	93	16	31	16	183	65
ENSMUSG00000032735.14	Ablim3	6.95E-09	5.19E-07	4	6	2420.25	272.1666667	2148.083333	3.240860104	3.437043026	Higher in Normal	2877	3000	1907	1897	43	147	314	65	607	457
ENSMUSG00000043518.7	Rai2	6.99E-09	5.20E-07	4	6	584	40.33333333	543.6666667	3.66838039	3.962751253	Higher in Normal	425	518	622	771	10	15	84	5	97	31
ENSMUSG00000022836.10	Mylk	7.27E-09	5.40E-07	4	6	22407.75	1424.5	20983.25	3.51951057	3.776838282	Higher in Normal	5123	5815	37527	41166	1033	471	2118	2107	1677	1141
ENSMUSG00000026691.10	Fmo3	7.36E-09	5.45E-07	4	6	64.25	2	62.25	4.559938162	5.198874528	Higher in Normal	20	10	102	125	0	0	1	2	2	7
ENSMUSG00000027073.5	Prg2	7.41E-09	5.48E-07	4	6	70.5	2.5	68	4.534931226	5.16696838	Higher in Normal	10	175	51	46	0	1	2	2	5	5
ENSMUSG00000026420.16	Il24	7.43E-09	5.48E-07	4	6	12	680.8333333	668.8333333	4.96716439	5.949656695	Higher in TRIM24	10	1	8	29	1166	796	57	13	1332	721
ENSMUSG00000044062.7	Plekhd1os	7.48E-09	5.50E-07	4	6	18.25	0.333333333	17.91666667	4.815360519	5.675707146	Higher in Normal	19	16	23	15	0	0	0	1	1	0
ENSMUSG00000059498.13	Fcgr3	7.59E-09	5.56E-07	4	6	591.5	6989	6397.5	3.16721209	3.355291164	Higher in TRIM24	740	1061	250	315	3356	6374	10891	3984	3832	13497
ENSMUSG00000096035.2	Odaph	7.64E-09	5.58E-07	4	6	12.75	483.8333333	471.0833333	4.739509113	5.548681721	Higher in TRIM24	31	11	6	3	166	1286	357	749	75	270
ENSMUSG00000049112.9	Oxtr	7.68E-09	5.60E-07	4	6	3322	112.8333333	3209.166667	4.306798905	4.817640582	Higher in Normal	2659	2701	3580	4348	259	13	224	13	145	23
ENSMUSG00000029797.13	Sspo	7.71E-09	5.60E-07	4	6	317.25	41	276.25	2.843950847	2.974131298	Higher in Normal	247	69	654	299	24	27	48	16	66	65
ENSMUSG00000015652.9	Steap1	8.02E-09	5.80E-07	4	6	49	1608.833333	1559.833333	4.167116331	4.655701259	Higher in TRIM24	78	27	51	40	41	639	1536	1316	348	5773
ENSMUSG00000020593.15	Lpin1	8.24E-09	5.93E-07	4	6	5235.75	873.3333333	4362.416667	2.568740392	2.663527278	Higher in Normal	7587	3417	5244	4695	954	464	739	279	1942	862
ENSMUSG00000059201.12	Lep	8.73E-09	6.25E-07	4	6	17564.25	159.6666667	17404.58333	5.681707889	7.228635447	Higher in Normal	32451	37552	131	123	62	35	95	15	627	124
ENSMUSG00000019987.9	Arg1	8.94E-09	6.39E-07	4	6	1208.75	11105.5	9896.75	2.84856668	2.983036508	Higher in TRIM24	749	1785	949	1352	13002	7981	6624	1774	15792	21460
ENSMUSG00000044788.10	Fads6	9.06E-09	6.46E-07	4	6	719.5	88.16666667	631.3333333	2.84266602	2.974053612	Higher in Normal	288	426	1146	1018	176	50	66	33	89	115
ENSMUSG00000028518.8	Prkaa2	9.20E-09	6.54E-07	4	6	1130.25	96.33333333	1033.916667	3.39286723	3.6246926	Higher in Normal	645	421	2097	1358	66	28	100	17	274	93
ENSMUSG00000025780.7	Itih5	9.23E-09	6.55E-07	4	6	7414.75	987.3333333	6427.416667	2.991443977	3.145994677	Higher in Normal	8353	10655	5778	4873	503	596	1385	164	1836	1440
ENSMUSG00000039092.9	Sptlc3	9.53E-09	6.75E-07	4	6	174	10.83333333	163.1666667	3.856777052	4.211314834	Higher in Normal	96	94	226	280	7	0	9	1	31	17
ENSMUSG00000071715.11	Ncf4	9.62E-09	6.79E-07	4	6	253.25	2352.833333	2099.583333	3.021939535	3.185776167	Higher in TRIM24	200	449	157	207	843	2262	3731	2520	2396	2365
ENSMUSG00000036395.15	Glb1l2	1.00E-08	7.04E-07	4	6	2647.75	77.16666667	2570.583333	4.7799982	5.539984436	Higher in Normal	4882	5334	213	162	51	15	54	15	228	100
ENSMUSG00000015733.13	Capza2	1.01E-08	7.05E-07	4	6	7240.5	381459.8333	374219.3333	4.731931129	5.548248159	Higher in TRIM24	5004	5938	7769	10251	878329	9163	331340	137212	9770	922945
ENSMUSG00000096844.1	Igkv6-14	1.08E-08	7.50E-07	4	6	52.5	1.5	51	4.371814581	4.975285982	Higher in Normal	63	33	57	57	5	2	2	0	0	0
ENSMUSG00000031785.16	Adgrg1	1.08E-08	7.51E-07	4	6	8650.5	799.6666667	7850.833333	3.222948113	3.421359831	Higher in Normal	2967	3282	13402	14951	613	279	826	326	2146	608
ENSMUSG00000054196.6	Cthrc1	1.26E-08	8.70E-07	4	6	244	4984.833333	4740.833333	3.769480514	4.121393267	Higher in TRIM24	136	97	290	453	626	586	5603	5589	1847	15658
ENSMUSG00000030187.15	Klra2	1.33E-08	9.16E-07	4	6	115.75	694.1666667	578.4166667	2.533974609	2.628970729	Higher in TRIM24	99	128	92	144	506	1073	381	469	708	1028
ENSMUSG00000030351.5	Tspan11	1.41E-08	9.63E-07	4	6	128.75	1520.5	1391.75	2.968609178	3.127115877	Higher in TRIM24	87	112	141	175	270	415	1497	757	706	5478
ENSMUSG00000044206.3	Vsig4	1.44E-08	9.84E-07	4	6	4.75	124.8333333	120.0833333	3.989548177	4.435287223	Higher in TRIM24	11	4	3	1	65	66	246	13	76	283
ENSMUSG00000027496.15	Aurka	1.46E-08	9.92E-07	4	6	318.75	1937	1618.25	2.50878861	2.60121427	Higher in TRIM24	207	123	403	542	1687	551	2045	2323	1309	3707
ENSMUSG00000031137.17	Fgf13	1.53E-08	1.03E-06	4	6	563.25	41.16666667	522.0833333	3.585533686	3.874318061	Higher in Normal	731	1043	256	223	16	32	92	26	55	26
ENSMUSG00000032940.16	Rbm11	1.57E-08	1.06E-06	4	6	74.25	10	64.25	2.886478876	3.029937576	Higher in Normal	90	48	73	86	1	5	20	6	8	20
ENSMUSG00000017692.8	Rhbdl3	1.63E-08	1.09E-06	4	6	958.5	115	843.5	2.924406966	3.073688379	Higher in Normal	852	384	1273	1325	22	50	139	101	252	126
ENSMUSG00000018451.6	6330403K07Rik	1.64E-08	1.10E-06	4	6	78.5	9.333333333	69.16666667	3.172710607	3.365391383	Higher in Normal	26	81	78	129	4	1	9	3	15	24
ENSMUSG00000038264.8	Sema7a	1.74E-08	1.16E-06	4	6	1399.5	14138.83333	12739.33333	3.388375883	3.637888176	Higher in TRIM24	708	660	2047	2183	9018	20097	24804	16513	4141	10260
ENSMUSG00000032218.7	Ccnb2	1.76E-08	1.17E-06	4	6	330	1942.333333	1612.333333	2.498047741	2.590333876	Higher in TRIM24	261	75	442	542	1479	770	2134	2202	1728	3341
ENSMUSG00000094930.2	Igkv6-25	1.76E-08	1.17E-06	4	6	119.5	8.5	111	3.949584697	4.351594522	Higher in Normal	20	256	79	123	4	1	8	3	5	30
ENSMUSG00000083443.1	Gm15519	1.78E-08	1.18E-06	4	6	217.5	9.833333333	207.6666667	4.394911786	4.982924476	Higher in Normal	404	411	30	25	8	4	2	3	14	28
ENSMUSG00000031098.18	Syt8	1.84E-08	1.22E-06	4	6	42	1051.166667	1009.166667	4.030748087	4.492600486	Higher in TRIM24	59	36	39	34	218	1145	199	120	3414	1211
ENSMUSG00000049130.6	C5ar1	1.92E-08	1.27E-06	4	6	335.5	2109.666667	1774.166667	2.518724091	2.614126627	Higher in TRIM24	280	424	298	340	734	2192	3056	1859	1360	3457
ENSMUSG00000056888.11	Glipr1	1.96E-08	1.29E-06	4	6	144.5	1524.333333	1379.833333	3.20240132	3.410751408	Higher in TRIM24	137	129	137	175	3947	653	1272	705	447	2122
ENSMUSG00000011034.6	Slc5a1	2.00E-08	1.31E-06	4	6	943	37.83333333	905.1666667	4.265174447	4.79580001	Higher in Normal	556	225	1458	1533	28	5	16	1	132	45
ENSMUSG00000001918.17	Slc1a5	2.00E-08	1.31E-06	4	6	19835	2391.5	17443.5	3.107937444	3.29191509	Higher in Normal	31367	33514	6866	7593	1603	1256	2068	1563	4762	3097
ENSMUSG00000087057.7	Gm11730	2.01E-08	1.31E-06	4	6	49.5	5.833333333	43.66666667	3.22670139	3.43442322	Higher in Normal	74	55	36	33	2	3	1	3	9	17
ENSMUSG00000054320.16	Lrrc36	2.03E-08	1.32E-06	4	6	199	19.5	179.5	3.587823202	3.880042779	Higher in Normal	224	173	185	214	11	2	3	2	32	67
ENSMUSG00000037161.14	Mgarp	2.07E-08	1.34E-06	4	6	18	257.5	239.5	3.296795954	3.528323917	Higher in TRIM24	38	10	14	10	125	58	155	302	111	794
ENSMUSG00000109244.1	Gm44751	2.09E-08	1.35E-06	4	6	119.5	954.5	835	2.507841517	2.602546025	Higher in TRIM24	186	154	61	77	452	494	763	456	762	2800
ENSMUSG00000020427.11	Igfbp3	2.11E-08	1.36E-06	4	6	2927.75	35207.83333	32280.08333	3.300019563	3.531417184	Higher in TRIM24	733	1279	4327	5372	8649	18831	63920	24315	14702	80830
ENSMUSG00000112532.1	Gm36283	2.12E-08	1.36E-06	4	6	58	6.666666667	51.33333333	3.016190827	3.186508208	Higher in Normal	62	38	81	51	1	9	9	1	9	11
ENSMUSG00000076594.2	Igkv6-13	2.14E-08	1.37E-06	4	6	27.5	2	25.5	3.60559639	3.919845253	Higher in Normal	35	32	16	27	3	1	2	0	4	2
ENSMUSG00000019368.13	Sec14l4	2.21E-08	1.41E-06	4	6	342	28.66666667	313.3333333	3.549310118	3.834681581	Higher in Normal	230	338	413	387	23	2	14	5	89	39
ENSMUSG00000033192.5	Lpcat2	2.24E-08	1.43E-06	4	6	410	3345	2935	2.598836464	2.705254932	Higher in TRIM24	201	255	456	728	1823	1223	2620	1603	1266	11535
ENSMUSG00000021614.16	Vcan	2.27E-08	1.44E-06	4	6	831.25	16616.5	15785.25	3.742572186	4.101322817	Higher in TRIM24	268	221	1673	1163	27052	3165	7361	4371	4695	53055
ENSMUSG00000112057.1	Gm21321	2.37E-08	1.50E-06	4	6	5.25	115.3333333	110.0833333	4.104748045	4.616493364	Higher in TRIM24	9	3	2	7	22	208	188	103	26	145
ENSMUSG00000113263.1	Gm4811	2.54E-08	1.60E-06	4	6	5.75	45.16666667	39.41666667	2.631753655	2.744551537	Higher in TRIM24	10	3	3	7	26	20	41	30	36	118
ENSMUSG00000029552.19	Tes	2.55E-08	1.61E-06	4	6	4047	201830.3333	197783.3333	4.623505159	5.431702308	Higher in TRIM24	1008	1166	6194	7820	296826	7001	189796	78535	5427	633397
ENSMUSG00000058435.5	Btnl4	2.60E-08	1.64E-06	4	6	32.75	1.833333333	30.91666667	4.017475079	4.463123869	Higher in Normal	49	16	35	31	0	1	0	0	4	6
ENSMUSG00000113581.1	Dio3os	2.62E-08	1.64E-06	4	6	2.75	70.66666667	67.91666667	4.04904957	4.551495661	Higher in TRIM24	4	4	2	1	6	34	116	54	145	69
ENSMUSG00000049723.14	Mmp12	2.66E-08	1.67E-06	4	6	648.25	9702.5	9054.25	3.664303691	4.001488168	Higher in TRIM24	229	461	881	1022	2360	8046	30743	2931	6038	8097

ENSMUSG00000036353.13	P2ry12	2.68E-08	1.68E-06	4	6	73.75	525.3333333	451.5833333	2.516546879	2.61405214	Higher in TRIM24	75	103	53	64	247	527	433	319	255	1371
ENSMUSG00000024578.2	Il17b	2.70E-08	1.68E-06	4	6	608	11.83333333	596.1666667	4.819811153	5.678139993	Higher in Normal	216	229	791	1196	13	0	19	0	36	3
ENSMUSG00000007034.15	Slc44a4	2.80E-08	1.74E-06	4	6	445.5	45.83333333	399.6666667	3.301172029	3.529670276	Higher in Normal	251	204	601	726	20	6	30	9	121	89
ENSMUSG00000086284.1	Frmpd1os	2.82E-08	1.75E-06	4	6	52	0.666666667	51.33333333	4.985665648	6.115693437	Higher in Normal	25	24	63	96	4	0	0	0	0	0
ENSMUSG00000041592.16	Sdk2	2.87E-08	1.78E-06	4	6	856.25	95.83333333	760.4166667	3.089283758	3.274283051	Higher in Normal	523	230	1635	1037	119	53	44	13	134	212
ENSMUSG00000053279.6	Aldh1a1	2.91E-08	1.80E-06	4	6	1739.75	90	1649.75	4.066245052	4.528696976	Higher in Normal	3172	2948	391	448	66	35	102	18	271	48
ENSMUSG00000086416.1	Gm14002	3.00E-08	1.84E-06	4	6	0.25	105	104.75	5.653629857	8.295179538	Higher in TRIM24	0	0	1	0	5	8	221	157	3	236
ENSMUSG00000063234.4	Gpr84	3.03E-08	1.86E-06	4	6	18.25	206	187.75	3.377750404	3.635240604	Higher in TRIM24	10	9	17	37	32	162	338	239	308	157
ENSMUSG00000015745.9	Plekho1	3.06E-08	1.87E-06	4	6	1041.75	6371.5	5329.75	2.346692236	2.4253288	Higher in TRIM24	1043	1417	740	967	3756	2905	9124	5886	3158	13400
ENSMUSG00000031980.9	Agt	3.06E-08	1.87E-06	4	6	902.5	48.5	854	3.916241242	4.324405809	Higher in Normal	916	954	711	1029	134	4	23	5	71	54
ENSMUSG00000020891.11	Alox8	3.09E-08	1.88E-06	4	6	69.5	9.666666667	59.83333333	2.990864019	3.156151207	Higher in Normal	57	29	108	84	4	2	5	2	15	30
ENSMUSG00000058396.7	Gpr182	3.20E-08	1.94E-06	4	6	435.5	55.66666667	379.8333333	2.973285022	3.138049842	Higher in Normal	403	426	376	537	23	20	86	10	134	61
ENSMUSG00000034911.8	Ushbp1	3.25E-08	1.97E-06	4	6	2980.25	577.3333333	2402.916667	2.566318139	2.669700537	Higher in Normal	4075	4214	1695	1937	170	307	496	358	947	1186
ENSMUSG00000048534.7	Jaml	3.36E-08	2.03E-06	4	6	93.25	715.1666667	621.9166667	2.739768717	2.869656627	Higher in TRIM24	120	88	77	88	239	596	1436	472	338	1210
ENSMUSG00000090665.2	Gad1-ps	3.41E-08	2.06E-06	4	6	7.25	96.5	89.25	3.365711952	3.62598592	Higher in TRIM24	12	7	8	2	44	98	63	30	234	110
ENSMUSG00000042821.7	Snai1	3.46E-08	2.08E-06	4	6	423.5	2791.833333	2368.333333	2.543546695	2.645931386	Higher in TRIM24	226	200	565	703	1348	1285	3379	2810	1133	6796
ENSMUSG00000026068.11	Il18rap	3.57E-08	2.14E-06	4	6	82.5	1097.333333	1014.833333	3.049290173	3.234271292	Higher in TRIM24	116	77	82	55	151	326	645	724	662	4076
ENSMUSG00000054675.5	Tmem119	3.59E-08	2.14E-06	4	6	459.75	3407.333333	2947.583333	2.724185197	2.852167312	Higher in TRIM24	496	633	293	417	1190	2451	5013	4696	2352	4742
ENSMUSG00000006675.10	P4htm	3.61E-08	2.15E-06	4	6	202	37.83333333	164.1666667	2.507530221	2.604129133	Higher in Normal	156	173	210	269	13	10	46	16	83	59
ENSMUSG00000076441.9	Ass1	3.61E-08	2.15E-06	4	6	426.25	2874.833333	2448.583333	2.813094494	2.955127518	Higher in TRIM24	221	263	533	688	5045	1456	2599	3864	2361	1924
ENSMUSG00000086040.8	Wipf3	3.72E-08	2.21E-06	4	6	811	45.5	765.5	3.801760878	4.175979236	Higher in Normal	161	166	1443	1474	25	23	30	7	140	48
ENSMUSG00000043157.7	Arl11	3.78E-08	2.25E-06	4	6	37	506.1666667	469.1666667	3.270426117	3.505978419	Higher in TRIM24	67	58	7	16	359	369	583	317	242	1167
ENSMUSG00000001095.5	Slc13a2	3.82E-08	2.26E-06	4	6	295	19.83333333	275.1666667	3.809836708	4.187411804	Higher in Normal	139	408	285	348	5	8	10	1	69	26
ENSMUSG00000028174.12	Rpe65	3.85E-08	2.27E-06	4	6	298.75	7	291.75	4.968414474	5.962702681	Higher in Normal	32	66	486	611	0	1	1	0	9	31
ENSMUSG00000041431.16	Ccnb1	3.85E-08	2.27E-06	4	6	301.5	2233.833333	1932.333333	2.697564795	2.822093225	Higher in TRIM24	164	65	468	509	2871	625	1658	1305	2081	4863
ENSMUSG00000030376.8	Slc8a2	3.91E-08	2.30E-06	4	6	288.5	13.5	275	3.884901712	4.291801197	Higher in Normal	134	33	514	473	28	6	19	1	14	13
ENSMUSG00000012428.9	Steap4	3.94E-08	2.31E-06	4	6	9690.75	1379	8311.75	2.89347716	3.046791622	Higher in Normal	14662	11179	6218	6704	1672	240	908	630	2961	1863
ENSMUSG00000040522.5	Tlr8	3.95E-08	2.31E-06	4	6	67.75	469.5	401.75	2.690560215	2.814288695	Higher in TRIM24	38	36	111	86	372	609	467	113	336	920
ENSMUSG00000030905.5	Crym	3.97E-08	2.32E-06	4	6	300.75	14.83333333	285.9166667	4.160749645	4.672958618	Higher in Normal	123	25	411	644	2	1	13	3	23	47
ENSMUSG00000036923.12	Stox1	4.03E-08	2.35E-06	4	6	157.75	33.5	124.25	2.354643372	2.434368912	Higher in Normal	115	77	215	224	11	8	41	19	41	81
ENSMUSG00000026204.15	Ptprn	4.08E-08	2.37E-06	4	6	104	1927.166667	1823.166667	3.892486745	4.325665959	Higher in TRIM24	201	31	96	88	134	1368	2824	3842	886	2509
ENSMUSG00000026840.14	Lamc3	4.15E-08	2.41E-06	4	6	867.5	86.16666667	781.3333333	3.107313359	3.30122538	Higher in Normal	853	388	1230	999	12	126	111	44	53	171
ENSMUSG00000093894.1	Ighv1-53	4.29E-08	2.49E-06	4	6	42.5	1.333333333	41.16666667	4.491142062	5.211103308	Higher in Normal	8	76	41	45	1	0	1	0	5	1
ENSMUSG00000015568.16	Lpl	4.41E-08	2.55E-06	4	6	93459.25	3086.833333	90372.41667	4.477728756	5.152801092	Higher in Normal	183386	180084	4666	5701	2638	960	4562	1965	6250	2146
ENSMUSG00000023992.14	Trem2	4.51E-08	2.60E-06	4	6	126.25	2257.5	2131.25	3.550614979	3.865878856	Higher in TRIM24	198	241	42	24	1441	1196	2371	2409	786	5342
ENSMUSG00000022227.4	Mcpt1	4.64E-08	2.67E-06	4	6	1.75	101	99.25	4.766656168	5.807155068	Higher in TRIM24	5	2	0	0	35	81	69	92	317	12
ENSMUSG00000036896.5	C1qc	4.73E-08	2.71E-06	4	6	2687.25	19624.16667	16936.91667	2.621839605	2.737194874	Higher in TRIM24	2035	4480	1672	2562	11715	12828	23001	23641	8724	37836
ENSMUSG00000021010.8	Npas3	4.92E-08	2.82E-06	4	6	405.25	57.66666667	347.5833333	2.845947221	2.993482169	Higher in Normal	357	133	578	553	44	6	26	41	105	124
ENSMUSG00000026580.16	Selp	4.97E-08	2.84E-06	4	6	6963.25	115.8333333	6847.416667	4.895231572	5.857791655	Higher in Normal	226	234	14342	13051	18	38	224	14	332	69
ENSMUSG00000032033.11	Barx2	5.07E-08	2.89E-06	4	6	3156.25	143.1666667	3013.083333	4.129440945	4.639524556	Higher in Normal	988	1770	4170	5697	29	20	75	19	603	113
ENSMUSG00000035451.7	Foxa1	5.13E-08	2.92E-06	4	6	2904.25	173.1666667	2731.083333	3.553095201	3.858583743	Higher in Normal	1208	1733	4183	4493	521	84	184	65	115	70
ENSMUSG00000055430.4	Nap1l5	5.18E-08	2.94E-06	4	6	320.25	16.5	303.75	4.099674061	4.599822296	Higher in Normal	480	680	58	63	9	6	16	5	50	13
ENSMUSG00000046711.15	Hmga1	5.55E-08	3.14E-06	4	6	854.25	9258.666667	8404.416667	3.165270525	3.381505844	Higher in TRIM24	330	354	1288	1445	1052	6026	15388	5999	6847	20240
ENSMUSG00000022132.15	Cldn10	5.57E-08	3.15E-06	4	6	789.25	57.16666667	732.0833333	3.58697173	3.904172008	Higher in Normal	317	641	902	1297	22	2	92	31	138	58
ENSMUSG00000079056.12	Kcnip3	5.61E-08	3.16E-06	4	6	342	61.83333333	280.1666667	2.328031045	2.407262223	Higher in Normal	121	199	460	588	41	34	76	65	75	80
ENSMUSG00000037872.5	Ackr1	5.62E-08	3.16E-06	4	6	1527	63.16666667	1463.833333	4.0655779	4.553045306	Higher in Normal	270	347	2279	3212	14	24	105	14	192	30
ENSMUSG00000036745.15	Ttll7	5.72E-08	3.21E-06	4	6	236.5	1823.166667	1586.666667	2.761816285	2.900170438	Higher in TRIM24	172	213	308	253	503	1024	2771	2366	681	3594
ENSMUSG00000001506.10	Col1a1	5.96E-08	3.33E-06	4	6	10844.5	167144.5	156300	3.519108361	3.831210419	Higher in TRIM24	11700	13641	8187	9850	12115	71689	450842	110415	164221	193585
ENSMUSG00000034310.8	Tmem132d	5.98E-08	3.33E-06	4	6	59.25	4	55.25	3.938615927	4.374338741	Higher in Normal	104	29	68	36	0	2	2	0	3	17
ENSMUSG00000019326.14	Aoc3	6.05E-08	3.36E-06	4	6	14841.5	740.8333333	14100.66667	4.07443819	4.568291723	Higher in Normal	28346	27032	2007	1981	639	373	768	233	1949	483
ENSMUSG00000023940.14	Sgo1	6.11E-08	3.38E-06	4	6	132.75	967.3333333	834.5833333	2.636884689	2.756471686	Higher in TRIM24	115	30	186	200	535	251	713	620	2058	1627
ENSMUSG00000062132.9	Arhgap33os	6.17E-08	3.41E-06	4	6	19.75	1	18.75	4.067567541	4.582406471	Higher in Normal	27	14	18	20	0	0	0	1	1	4
ENSMUSG00000096515.5	Igkv14-100	6.61E-08	3.64E-06	4	6	51.75	1.333333333	50.41666667	4.410874244	5.139669026	Higher in Normal	4	57	66	80	1	3	2	1	0	1
ENSMUSG00000031444.16	F10	6.66E-08	3.66E-06	4	6	66.5	1466.166667	1399.666667	3.971950941	4.459238633	Higher in TRIM24	58	105	49	54	151	2451	3468	232	727	1768
ENSMUSG00000102882.1	Gm2065	6.67E-08	3.66E-06	4	6	28.75	2.666666667	26.08333333	3.362838946	3.623044493	Higher in Normal	22	11	49	33	1	1	1	1	4	8
ENSMUSG00000026616.15	Cr2	6.70E-08	3.67E-06	4	6	114.5	14.83333333	99.66666667	2.932387326	3.098748616	Higher in Normal	46	77	196	139	1	6	16	12	21	33
ENSMUSG00000073821.11	8030451A03Rik	6.77E-08	3.70E-06	4	6	7.5	107.8333333	100.3333333	3.738731732	4.133142809	Higher in TRIM24	9	4	9	8	27	252	83	88	147	50
ENSMUSG00000002831.13	Plin4	7.17E-08	3.89E-06	4	6	18068.75	436.6666667	17632.08333	4.818275778	5.754489888	Higher in Normal	30720	39999	819	737	335	106	266	62	1582	269
ENSMUSG00000066361.3	Serpina3c	7.21E-08	3.90E-06	4	6	1138.75	20.33333333	1118.416667	5.063126982	6.218009604	Higher in Normal	1955	2543	26	31	11	3	21	2	71	14
ENSMUSG00000046841.5	Ckap4	7.26E-08	3.92E-06	4	6	1414.75	9247.666667	7832.916667	2.593254422	2.708130655	Higher in TRIM24	794	1036	1686	2143	2998	3854	14573	12404	5963	15694
ENSMUSG00000113599.1	9530014B07Rik	7.31E-08	3.94E-06	4	6	174.25	5	169.25	4.420543735	5.105480909	Higher in Normal	132	170	162	233	1	0	18	3	8	0
ENSMUSG00000000440.12	Pparg	7.54E-08	4.05E-06	4	6	3284.5	294.5	2990	3.441707074	3.724618957	Higher in Normal	5872	4558	1247	1461	274	106	151	97	898	241
ENSMUSG00000025128.7	Bhlhe22	7.80E-08	4.17E-06	4	6	10.75	169.8333333	159.0833333	3.782325124	4.197351924	Higher in TRIM24	9	8	12	14	20	107	246	386	194	66
ENSMUSG00000113101.1	Gm33424	7.89E-08	4.22E-06	4	6	59.5	5.5	54	3.378582823	3.646334932	Higher in Normal	13	62	67	96	2	0	6	4	9	12
ENSMUSG00000038463.8	Olfml2b	7.98E-08	4.25E-06	4	6	323.5	4707.833333	4384.333333	3.623042206	3.977156567	Higher in TRIM24	244	286	294	470	725	1264	6517	11965	1954	5822
ENSMUSG00000096422.2	Igkv12-44	8.18E-08	4.35E-06	4	6	272.5	18.16666667	254.3333333	3.605634778	3.940028701	Higher in Normal	317	361	187	225	28	7	48	2	16	8
ENSMUSG00000048779.5	P2ry6	8.25E-08	4.38E-06	4	6	212.5	2239.166667	2026.666667	3.112222423	3.323139291	Higher in TRIM24	246	401	99	104	874	2497	3384	2288	1391	3001
ENSMUSG00000078763.2	Slfn1	8.47E-08	4.48E-06	4	6	60.5	540.6666667	480.1666667	2.82699359	2.981276982	Higher in TRIM24	41	133	27	41	490	437	337	366	908	706
ENSMUSG00000079588.3	Tmem182	8.66E-08	4.55E-06	4	6	1638.25	96	1542.25	3.86615851	4.290260263	Higher in Normal	2936	2113	719	785	33	33	254	9	179	68
ENSMUSG00000026610.13	Esrrg	9.25E-08	4.83E-06	4	6	512.75	30.66666667	482.0833333	3.775296778	4.168154769	Higher in Normal	186	146	942	777	42	1	14	7	74	46
ENSMUSG00000043639.14	Rbm20	9.42E-08	4.91E-06	4	6	364.5	61.83333333	302.6666667	2.657343429	2.781886987	Higher in Normal	437	483	271	267	82	9	81	27	70	102
ENSMUSG00000024347.16	Psd2	9.44E-08	4.91E-06	4	6	281.25	17.66666667	263.5833333	3.670085154	4.028344475	Higher in Normal	95	58	514	458	7	12	3	6	54	24
ENSMUSG00000040649.15	Rimklb	9.65E-08	5.01E-06	4	6	199.25	17.83333333	181.4166667	3.664760209	4.018363164	Higher in Normal	246	131	234	186	4	0	11	2	29	61
ENSMUSG00000022687.12	Boc	9.84E-08	5.09E-06	4	6	7536	773	6763	2.992600471	3.175359766	Higher in Normal	3290	1441	13030	12383	832	579	479	350	1707	691
ENSMUSG00000031790.8	Mmp15	9.85E-08	5.09E-06	4	6	10291.75	1178.833333	9112.916667	2.866657199	3.025797953	Higher in Normal	3333	3505	16902	17427	1571	475	1271	639	2217	900
ENSMUSG00000046856.13	Gpr1	9.91E-08	5.11E-06	4	6	217.75	21.33333333	196.4166667	3.214203858	3.445291421	Higher in Normal	127	163	245	336	18	11	41	0	25	33
ENSMUSG00000032010.15	Usp2	1.05E-07	5.40E-06	4	6	1677.25	279.6666667	1397.583333	2.386448174	2.475869464	Higher in Normal	654	631	2456	2968	261	161	315	233	423	285
ENSMUSG00000036905.8	C1qb	1.06E-07	5.42E-06	4	6	3101.5	20390	17288.5	2.491516919	2.595422839	Higher in TRIM24	2139	5434	1900	2933	13495	13644	23308	24164	11333	36396
ENSMUSG00000043388.8	Tmem130	1.06E-07	5.46E-06	4	6	145.5	13.16666667	132.3333333	3.196351765	3.425318379	Higher in Normal	124	37	183	238	29	2	16	5	10	17
ENSMUSG00000028859.14	Csf3r	1.10E-07	5.65E-06	4	6	192	2208.333333	2016.333333	3.037058081	3.235886917	Higher in TRIM24	237	241	155	135	1126	919	800	596	6890	2919
ENSMUSG00000042379.8	Esm1	1.11E-07	5.66E-06	4	6	197.25	1111	913.75	2.521742211	2.630152296	Higher in TRIM24	104	162	243	280	614	1083	1072	1764	613	1520
ENSMUSG00000068079.5	Tcf15	1.12E-07	5.70E-06	4	6	440.75	33.83333333	406.9166667	3.646648612	4.001494351	Higher in Normal	715	622	168	258	5	9	26	16	115	32

ENSMUSG00000079685.10	Ulbp1	1.15E-07	5.84E-06	4	6	289	2741.5	2452.5	3.105253132	3.320025144	Higher in TRIM24	174	87	494	401	781	3191	4670	1618	874	5315
ENSMUSG00000091971.3	Hspa1a	1.24E-07	6.24E-06	4	6	17704.75	359.5	17345.25	4.830782129	5.832490167	Higher in Normal	175	181	31410	39053	47	84	167	331	306	1222
ENSMUSG00000038077.7	Kcna6	1.24E-07	6.25E-06	4	6	133.25	14.66666667	118.5833333	2.955476037	3.135573648	Higher in Normal	52	106	176	199	4	13	34	7	15	15
ENSMUSG00000029379.10	Cxcl3	1.28E-07	6.39E-06	4	6	50	1659.333333	1609.333333	4.217554511	4.869094479	Higher in TRIM24	63	11	58	68	99	225	1808	195	6283	1346
ENSMUSG00000015441.3	Gzmf	1.28E-07	6.41E-06	4	6	8.25	1122.333333	1114.083333	5.218520164	7.042493201	Higher in TRIM24	25	1	2	5	1	1140	2202	53	2790	548
ENSMUSG00000025185.14	Loxl4	1.29E-07	6.45E-06	4	6	71	831.5	760.5	3.496958515	3.822746243	Higher in TRIM24	29	37	133	85	318	1890	1031	437	235	1078
ENSMUSG00000086077.7	Gm14396	1.30E-07	6.48E-06	4	6	18.25	0.333333333	17.91666667	4.771313525	5.717270946	Higher in Normal	19	9	31	14	0	0	0	0	0	2
ENSMUSG00000037379.7	Spon2	1.32E-07	6.55E-06	4	6	2900	197.5	2702.5	3.641472847	3.999090685	Higher in Normal	998	1108	3887	5607	58	12	223	77	584	231
ENSMUSG00000044155.11	Lsm8	1.34E-07	6.65E-06	4	6	1110.25	63553	62442.75	4.560240932	5.466559853	Higher in TRIM24	817	887	1153	1584	99002	1058	5029	42775	1899	231555
ENSMUSG00000069792.5	Wfdc17	1.34E-07	6.65E-06	4	6	141.75	1468.166667	1326.416667	2.803009942	2.958091161	Higher in TRIM24	154	249	55	109	921	384	1904	401	1696	3503
ENSMUSG00000010830.7	Kdelr3	1.37E-07	6.75E-06	4	6	290.75	2988.166667	2697.416667	3.016187491	3.213805998	Higher in TRIM24	215	291	237	420	374	1130	3749	4100	1493	7083
ENSMUSG00000024810.16	Il33	1.39E-07	6.83E-06	4	6	257.25	4067.666667	3810.416667	3.290686098	3.556102876	Higher in TRIM24	134	194	340	361	4431	253	1353	813	4008	13548
ENSMUSG00000045287.6	Rtn4rl1	1.39E-07	6.83E-06	4	6	786	107.5	678.5	2.8783851	3.044067788	Higher in Normal	1003	1461	342	338	109	46	147	75	160	108
ENSMUSG00000105632.1	Gm43272	1.41E-07	6.91E-06	4	6	30.75	3.333333333	27.41666667	3.068243731	3.275694159	Higher in Normal	19	14	50	40	2	4	3	0	4	7
ENSMUSG00000029661.16	Col1a2	1.42E-07	6.97E-06	4	6	11818	244057.1667	232239.1667	3.972022861	4.493512083	Higher in TRIM24	11001	12753	11053	12465	13020	52596	383626	675241	133421	206439
ENSMUSG00000001025.8	S100a6	1.44E-07	7.03E-06	4	6	6330.25	75224	68893.75	3.082833893	3.296229134	Higher in TRIM24	3858	10968	3995	6500	10407	42176	73793	87276	36904	200788
ENSMUSG00000037086.3	Prr32	1.46E-07	7.10E-06	4	6	301.25	5.333333333	295.9166667	4.917897361	6.036243714	Higher in Normal	576	616	7	6	2	2	4	6	16	2
ENSMUSG00000051726.6	Kcnf1	1.50E-07	7.30E-06	4	6	7.5	295.8333333	288.3333333	4.581432038	5.527605647	Higher in TRIM24	4	5	14	7	5	424	631	383	13	319
ENSMUSG00000049538.14	Adamts16	1.54E-07	7.48E-06	4	6	24	528	504	3.876766016	4.358623041	Higher in TRIM24	57	16	19	4	20	461	786	483	580	838
ENSMUSG00000086256.1	Gm12052	1.57E-07	7.59E-06	4	6	27	2.833333333	24.16666667	3.132587892	3.357290351	Higher in Normal	10	20	38	40	3	2	1	2	2	7
ENSMUSG00000049649.8	Gpr3	1.57E-07	7.59E-06	4	6	654	15.5	638.5	4.444379956	5.187924402	Higher in Normal	16	12	1186	1402	11	7	23	18	19	15
ENSMUSG00000041272.11	Tox	1.58E-07	7.63E-06	4	6	345.5	43.83333333	301.6666667	3.074536108	3.280911164	Higher in Normal	195	125	467	595	10	5	28	18	85	117
ENSMUSG00000095007.1	Igkv12-41	1.67E-07	8.00E-06	4	6	41.25	4.333333333	36.91666667	3.040004952	3.245613475	Higher in Normal	18	45	58	44	5	3	5	2	9	2
ENSMUSG00000072663.12	Spef2	1.69E-07	8.11E-06	4	6	192.75	30	162.75	3.036718427	3.235513231	Higher in Normal	267	229	133	142	14	5	11	5	46	99
ENSMUSG00000027524.9	Edn3	1.76E-07	8.40E-06	4	6	19.75	1.666666667	18.08333333	3.465022184	3.780014639	Higher in Normal	17	13	22	27	0	1	1	0	3	5
ENSMUSG00000001197.7	Nalcn	1.82E-07	8.65E-06	4	6	305	16.33333333	288.6666667	4.05789552	4.592420677	Higher in Normal	92	69	597	462	6	0	3	4	35	50
ENSMUSG00000064293.14	Cntn4	1.83E-07	8.70E-06	4	6	316.5	19.33333333	297.1666667	3.884127864	4.343057425	Higher in Normal	329	13	503	421	3	2	9	9	39	54
ENSMUSG00000051111.16	Sv2c	1.87E-07	8.87E-06	4	6	448.5	34.83333333	413.6666667	3.483028216	3.800237941	Higher in Normal	102	309	668	715	5	3	71	27	41	62
ENSMUSG00000062345.10	Serpinb2	1.99E-07	9.38E-06	4	6	12.25	782.8333333	770.5833333	4.96346718	6.412686625	Higher in TRIM24	27	2	10	10	58	3056	137	53	1299	94
ENSMUSG00000036655.8	Colec11	2.01E-07	9.45E-06	4	6	79.25	12.66666667	66.58333333	2.815960132	2.973349875	Higher in Normal	43	71	90	113	4	1	6	7	23	35
ENSMUSG00000046182.8	Gsg1l	2.05E-07	9.58E-06	4	6	155.75	7.166666667	148.5833333	3.991646917	4.505794458	Higher in Normal	35	84	232	272	1	0	6	5	26	5
ENSMUSG00000104422.1	Ighv1-14	2.06E-07	9.61E-06	4	6	206.25	6.333333333	199.9166667	4.26792289	4.929032644	Higher in Normal	11	94	334	386	8	0	15	6	1	8
ENSMUSG00000038665.15	Dgki	2.08E-07	9.69E-06	4	6	421.25	45.66666667	375.5833333	3.299390896	3.566365514	Higher in Normal	179	863	352	291	79	9	28	7	48	103
ENSMUSG00000062542.11	Syt9	2.10E-07	9.79E-06	4	6	531.75	21.5	510.25	4.052544242	4.594674418	Higher in Normal	283	72	888	884	3	10	38	2	67	9
ENSMUSG00000074489.9	Bglap3	2.17E-07	1.01E-05	4	6	4032.75	128.1666667	3904.583333	4.388972354	5.116202775	Higher in Normal	1733	1799	6688	5911	30	27	29	5	633	45
ENSMUSG00000010751.15	Tnfrsf22	2.20E-07	1.02E-05	4	6	217.5	1677.833333	1460.333333	2.89812518	3.078062546	Higher in TRIM24	275	176	179	240	361	3060	1980	1165	1723	1778
ENSMUSG00000045328.11	Cenpe	2.20E-07	1.02E-05	4	6	422	2800	2378	2.513244127	2.626276754	Higher in TRIM24	309	95	688	596	1879	640	2199	2912	2337	6833
ENSMUSG00000035486.14	Plk5	2.21E-07	1.02E-05	4	6	46.5	4	42.5	3.283334786	3.553193076	Higher in Normal	24	24	60	78	1	5	8	0	3	7
ENSMUSG00000021779.16	Thrb	2.22E-07	1.02E-05	4	6	847.25	165.8333333	681.4166667	2.56828942	2.687521471	Higher in Normal	1021	966	781	621	164	27	176	34	228	366
ENSMUSG00000048981.1	Krt31	2.25E-07	1.03E-05	4	6	44.25	1	43.25	4.726326256	5.741785795	Higher in Normal	11	6	74	86	0	0	0	0	2	4
ENSMUSG00000040724.5	Kcna2	2.38E-07	1.08E-05	4	6	286.5	27.33333333	259.1666667	3.63138327	4.002370423	Higher in Normal	222	390	264	270	6	3	6	2	57	90
ENSMUSG00000028172.5	Tacr3	2.39E-07	1.09E-05	4	6	131.25	7.666666667	123.5833333	3.68707648	4.083833787	Higher in Normal	101	31	201	192	23	1	3	1	6	12
ENSMUSG00000055322.15	Tns1	2.42E-07	1.10E-05	4	6	17040.25	3207.666667	13832.58333	2.360340897	2.452345304	Higher in Normal	22672	18505	15067	11917	1321	2912	5165	1817	4939	3092
ENSMUSG00000020592.14	Sdc1	2.44E-07	1.11E-05	4	6	3896.5	30013	26116.5	2.876604745	3.053618369	Higher in TRIM24	1311	1337	5592	7346	10063	41679	16267	19142	43495	49432
ENSMUSG00000058568.6	Defb50	2.48E-07	1.12E-05	4	6	24.75	0.333333333	24.41666667	4.89187301	6.138571044	Higher in Normal	2	29	30	38	0	0	0	1	0	1
ENSMUSG00000046699.14	Slitrk4	2.55E-07	1.15E-05	4	6	198.75	14.33333333	184.4166667	3.788413526	4.221909811	Higher in Normal	184	291	166	154	10	1	1	2	44	28
ENSMUSG00000071230.6	Npw	2.56E-07	1.15E-05	4	6	311.25	14.66666667	296.5833333	3.820574702	4.270443005	Higher in Normal	58	58	417	712	36	1	13	11	8	19
ENSMUSG00000069516.8	Lyz2	2.56E-07	1.15E-05	4	6	15284.25	120975.6667	105691.4167	2.6442197	2.779086188	Higher in TRIM24	15594	19755	10571	15217	35428	111839	179018	59506	45227	294836
ENSMUSG00000036585.15	Fgf1	2.57E-07	1.15E-05	4	6	3031.25	166.6666667	2864.583333	3.696242091	4.095108302	Higher in Normal	780	1584	4699	5062	192	44	29	161	524	50
ENSMUSG00000084793.1	Gm2568	2.63E-07	1.18E-05	4	6	33.5	2.833333333	30.66666667	3.515974568	3.852244617	Higher in Normal	44	12	35	43	0	0	1	2	5	9
ENSMUSG00000050558.13	Prokr2	2.65E-07	1.19E-05	4	6	18	250	232	3.499514918	3.849263401	Higher in TRIM24	42	9	14	7	66	435	318	68	373	240
ENSMUSG00000098874.1	Gm27960	2.73E-07	1.22E-05	4	6	2.75	120.6666667	117.9166667	4.324158974	5.128491131	Higher in TRIM24	1	3	3	4	234	6	60	33	8	383
ENSMUSG00000038567.5	Cyp24a1	2.74E-07	1.22E-05	4	6	540.25	25.83333333	514.4166667	3.956072142	4.465484827	Higher in Normal	138	30	1007	986	49	5	10	3	29	59
ENSMUSG00000041134.15	Cyyr1	2.83E-07	1.26E-05	4	6	1598.75	190.3333333	1408.416667	2.886707745	3.063571753	Higher in Normal	874	413	3045	2063	71	158	247	41	368	257
ENSMUSG00000044349.15	Snhg11	2.86E-07	1.27E-05	4	6	1704.5	80.16666667	1624.333333	4.293410418	4.981711673	Higher in Normal	1178	3676	1266	698	25	9	5	5	284	153
ENSMUSG00000031196.13	F8	2.88E-07	1.28E-05	4	6	321.75	45.66666667	276.0833333	2.745191986	2.895663226	Higher in Normal	198	60	593	436	30	14	35	21	100	74
ENSMUSG00000022265.6	Ank	2.89E-07	1.28E-05	4	6	2249.25	13776.5	11527.25	2.328597898	2.419122365	Higher in TRIM24	2753	1552	2175	2517	4197	10411	14372	10056	4905	38718
ENSMUSG00000045136.6	Tubb2b	2.89E-07	1.28E-05	4	6	480	3227.666667	2747.666667	2.507345672	2.622022129	Higher in TRIM24	322	161	662	775	1230	1982	4649	1626	1329	8550
ENSMUSG00000049791.4	Fzd4	2.93E-07	1.29E-05	4	6	6322	890.1666667	5431.833333	2.847654151	3.017377511	Higher in Normal	10477	10111	2563	2137	454	798	935	487	1440	1227
ENSMUSG00000049336.16	Tenm2	2.96E-07	1.30E-05	4	6	1117.5	52.5	1065	4.252738314	4.918545532	Higher in Normal	716	264	2000	1490	12	6	11	0	103	183
ENSMUSG00000028716.15	Pdzk1ip1	3.02E-07	1.33E-05	4	6	517.5	52.66666667	464.8333333	3.122154928	3.351856817	Higher in Normal	336	477	467	790	95	15	38	7	123	38
ENSMUSG00000038496.16	Slc19a3	3.13E-07	1.37E-05	4	6	55.25	6.5	48.75	3.056690575	3.272296864	Higher in Normal	28	58	65	70	0	4	6	2	16	11
ENSMUSG00000095630.2	Igkv6-23	3.14E-07	1.37E-05	4	6	270	14.33333333	255.6666667	3.886647023	4.372615178	Higher in Normal	26	57	425	572	4	11	19	4	2	46
ENSMUSG00000037798.6	Mat1a	3.14E-07	1.37E-05	4	6	142	9.333333333	132.6666667	3.54577222	3.899997002	Higher in Normal	84	47	198	239	13	3	3	2	31	4
ENSMUSG00000029321.1	Slc10a6	3.15E-07	1.37E-05	4	6	769	78.66666667	690.3333333	3.101578892	3.326995548	Higher in Normal	235	160	1204	1477	16	38	110	35	143	130
ENSMUSG00000022435.5	Upk3a	3.18E-07	1.39E-05	4	6	610	13	597	4.680706083	5.660614595	Higher in Normal	178	287	789	1186	2	0	19	0	52	5
ENSMUSG00000022440.8	C1qtnf6	3.22E-07	1.40E-05	4	6	784	8685.333333	7901.333333	3.218846179	3.483452566	Higher in TRIM24	357	481	1006	1292	784	4468	11886	13575	3836	17563
ENSMUSG00000043903.3	Zfp469	3.33E-07	1.44E-05	4	6	79.25	638.1666667	558.9166667	2.897645745	3.083978827	Higher in TRIM24	103	52	82	80	163	251	806	1340	475	794
ENSMUSG00000045259.4	Klhdc9	3.48E-07	1.50E-05	4	6	19.75	2.333333333	17.41666667	3.007325082	3.216449641	Higher in Normal	18	12	22	27	1	2	2	0	4	5
ENSMUSG00000102329.1	Gm10851	3.60E-07	1.55E-05	4	6	162.25	23	139.25	2.978753803	3.177588889	Higher in Normal	194	130	157	168	18	2	8	4	53	53
ENSMUSG00000006567.11	Atp7b	3.62E-07	1.56E-05	4	6	365.75	33.33333333	332.4166667	3.176137875	3.423289122	Higher in Normal	325	37	655	446	43	18	8	19	70	42
ENSMUSG00000063506.13	Arhgap22	3.92E-07	1.67E-05	4	6	46.75	768.5	721.75	3.513112701	3.87943428	Higher in TRIM24	47	29	55	56	48	915	614	515	111	2408
ENSMUSG00000020695.14	Mrc2	3.93E-07	1.67E-05	4	6	776.5	9021.166667	8244.666667	3.012561769	3.227642014	Higher in TRIM24	660	1284	593	569	1442	2862	9359	11742	3992	24730
ENSMUSG00000017737.2	Mmp9	3.93E-07	1.67E-05	4	6	348.75	11837.16667	11488.41667	4.153282079	4.842882241	Higher in TRIM24	311	932	63	89	716	6694	5545	26039	6600	25429
ENSMUSG00000076523.2	Igkv15-103	3.95E-07	1.68E-05	4	6	120.75	9	111.75	3.481133922	3.822624418	Higher in Normal	22	236	98	127	8	6	11	4	22	3
ENSMUSG00000001435.15	Col18a1	3.97E-07	1.69E-05	4	6	4665.5	25286	20620.5	2.499126919	2.615611445	Higher in TRIM24	3783	3193	5663	6023	39510	34884	27542	9351	19100	21329
ENSMUSG00000026180.8	Cxcr2	4.05E-07	1.72E-05	4	6	36.75	804.1666667	767.4166667	3.677625896	4.111635123	Higher in TRIM24	99	6	30	12	652	103	264	223	1974	1609
ENSMUSG00000030048.5	Gkn3	4.21E-07	1.78E-05	4	6	31	2	29	3.674491094	4.104995626	Higher in Normal	33	57	11	23	2	1	5	1	1	2
ENSMUSG00000027379.13	Bub1	4.23E-07	1.79E-05	4	6	248.5	1458.333333	1209.833333	2.377816778	2.477562001	Higher in TRIM24	162	40	412	380	1093	498	1260	1073	1647	3179
ENSMUSG00000109875.1	Gm45456	4.32E-07	1.82E-05	4	6	0.5	102.6666667	102.1666667	5.210335375	7.824640575	Higher in TRIM24	0	1	1	0	2	2	265	225	112	10
ENSMUSG00000064156.8	Prol1	4.42E-07	1.86E-05	4	6	113	5.666666667	107.3333333	4.02489504	4.602639998	Higher in Normal	102	247	47	56	9	7	0	0	5	13
ENSMUSG00000036887.5	C1qa	4.51E-07	1.89E-05	4	6	4301.5	32886.16667	28584.66667	2.667552684	2.813027255	Higher in TRIM24	3290	7680	2376	3860	17649	20926	43187	43307	13009	59239

ENSMUSG00000055926.2	Gm14137	4.51E-07	1.89E-05	4	6	48.25	588.5	540.25	3.168796877	3.427828084	Higher in TRIM24	51	55	52	35	36	656	359	235	1130	1115
ENSMUSG00000028238.6	Atp6v0d2	4.53E-07	1.89E-05	4	6	62.5	981.3333333	918.8333333	3.413194665	3.748761787	Higher in TRIM24	111	92	18	29	331	384	663	1792	296	2422
ENSMUSG00000024056.9	Ndc80	4.63E-07	1.93E-05	4	6	222.75	1646.666667	1423.916667	2.553164722	2.679544846	Higher in TRIM24	191	81	269	350	760	331	864	831	3925	3169
ENSMUSG00000002578.17	Ikzf4	4.64E-07	1.93E-05	4	6	1841.5	171	1670.5	3.242971512	3.51301838	Higher in Normal	489	243	3151	3483	198	22	153	65	241	347
ENSMUSG00000032327.14	Stra6	4.72E-07	1.96E-05	4	6	263	3116	2853	3.467738271	3.823222161	Higher in TRIM24	110	53	374	515	7435	1995	3749	957	3736	824
ENSMUSG00000005800.3	Mmp8	4.77E-07	1.98E-05	4	6	16.5	297.5	281	3.679796795	4.123389936	Higher in TRIM24	49	3	8	6	49	430	272	120	469	445
ENSMUSG00000098836.1	Gm27572	4.79E-07	1.98E-05	4	6	51.75	6.166666667	45.58333333	3.11482423	3.349668964	Higher in Normal	52	20	58	77	7	0	2	1	9	18
ENSMUSG00000030873.9	Scnn1b	4.79E-07	1.98E-05	4	6	3375.75	85.66666667	3290.083333	4.504185581	5.380137663	Higher in Normal	560	1222	5284	6437	54	9	22	2	408	19
ENSMUSG00000067786.16	Nnat	4.97E-07	2.05E-05	4	6	19526	390.5	19135.5	4.787335093	5.922231066	Higher in Normal	26810	50619	387	288	148	179	1201	36	535	244
ENSMUSG00000082902.3	Ccl19-ps1	5.11E-07	2.10E-05	4	6	37.5	0.333333333	37.16666667	5.012284047	6.535528116	Higher in Normal	1	13	60	76	0	0	1	0	1	0
ENSMUSG00000084920.1	Gm15230	5.15E-07	2.12E-05	4	6	45.25	1.833333333	43.41666667	4.122638811	4.767744229	Higher in Normal	15	27	71	68	1	0	0	0	8	2
ENSMUSG00000044786.6	Zfp36	5.24E-07	2.15E-05	4	6	67536.5	2608	64928.5	3.967665787	4.514979017	Higher in Normal	2076	2320	118074	147676	2691	1666	2677	1762	4148	2704
ENSMUSG00000023828.2	Slc22a3	5.25E-07	2.15E-05	4	6	911.25	42.33333333	868.9166667	4.078912941	4.685029273	Higher in Normal	1551	1363	351	380	71	4	18	2	136	23
ENSMUSG00000100782.1	Gm28231	5.27E-07	2.15E-05	4	6	2	66.16666667	64.16666667	4.306905804	5.170069065	Higher in TRIM24	3	1	4	0	7	130	77	56	8	119
ENSMUSG00000097789.2	Gm2115	5.34E-07	2.18E-05	4	6	39.25	1430.166667	1390.916667	4.148962973	4.861465968	Higher in TRIM24	103	26	15	13	70	1448	1619	710	74	4660
ENSMUSG00000055937.1	Krt28	5.34E-07	2.18E-05	4	6	80.75	3.333333333	77.41666667	4.223128907	4.914726757	Higher in Normal	37	6	129	151	0	0	1	1	7	11
ENSMUSG00000020788.15	Atp2a3	5.49E-07	2.23E-05	4	6	2096	304.6666667	1791.333333	2.70594717	2.85775002	Higher in Normal	956	2644	2419	2365	105	119	735	166	404	299
ENSMUSG00000070524.1	Fcrlb	5.65E-07	2.28E-05	4	6	45.5	649.1666667	603.6666667	3.412352473	3.754713555	Higher in TRIM24	122	17	31	12	153	602	953	661	219	1307
ENSMUSG00000021892.14	Sh3bp5	5.65E-07	2.28E-05	4	6	6409.5	953.3333333	5456.166667	2.492322203	2.609358017	Higher in Normal	3138	3203	8913	10384	370	1365	1238	482	1090	1175
ENSMUSG00000035681.7	Kcnc2	5.79E-07	2.33E-05	4	6	285.75	53.66666667	232.0833333	2.670430231	2.816384325	Higher in Normal	372	285	263	223	7	28	17	30	74	166
ENSMUSG00000001930.17	Vwf	5.85E-07	2.35E-05	4	6	6364.75	782.5	5582.25	2.739814122	2.898667982	Higher in Normal	2134	2713	11245	9367	521	750	1505	232	948	739
ENSMUSG00000095682.1	Igkv3-1	5.90E-07	2.36E-05	4	6	174.5	5	169.5	4.61549303	5.619551032	Higher in Normal	4	496	85	113	9	0	4	0	4	13
ENSMUSG00000020303.2	Stc2	5.95E-07	2.37E-05	4	6	3402.75	465.5	2937.25	2.730864484	2.88829428	Higher in Normal	4868	3636	2291	2816	746	99	741	357	568	282
ENSMUSG00000069920.10	B3gnt9	6.10E-07	2.43E-05	4	6	112.75	971.3333333	858.5833333	2.697017851	2.851938637	Higher in TRIM24	92	124	110	125	117	369	1012	1081	582	2667
ENSMUSG00000035864.14	Syt1	6.27E-07	2.49E-05	4	6	383.75	26.5	357.25	3.776158317	4.242081149	Higher in Normal	183	38	703	611	15	3	5	2	46	88
ENSMUSG00000076538.1	Igkv13-84	6.28E-07	2.49E-05	4	6	50.75	1	49.75	4.518447935	5.53046838	Higher in Normal	123	6	27	47	3	1	2	0	0	0
ENSMUSG00000047730.17	Fcgbp	6.52E-07	2.59E-05	4	6	137670.25	2818	134852.25	4.670635824	5.726208492	Higher in Normal	21553	31583	267225	230320	357	166	631	152	15069	533
ENSMUSG00000028631.7	Kcnq4	6.54E-07	2.59E-05	4	6	396	52.5	343.5	2.854526882	3.038024873	Higher in Normal	620	585	203	176	40	45	78	15	93	44
ENSMUSG00000023064.4	Sncg	6.64E-07	2.62E-05	4	6	14297.75	614.1666667	13683.58333	4.152818449	4.816115513	Higher in Normal	25770	26505	1870	3046	867	53	599	94	1842	230
ENSMUSG00000020374.16	Rasgef1c	6.66E-07	2.63E-05	4	6	493.25	17.5	475.75	4.242024316	4.961938413	Higher in Normal	92	252	717	912	5	2	2	2	85	9
ENSMUSG00000096140.1	Ankrd66	6.77E-07	2.66E-05	4	6	3.5	63.5	60	3.514314981	3.914795792	Higher in TRIM24	6	6	2	0	8	29	68	68	72	136
ENSMUSG00000105746.1	Gm43595	7.03E-07	2.75E-05	4	6	26.5	1.5	25	3.805526508	4.315374957	Higher in Normal	48	16	34	8	3	0	0	1	2	3
ENSMUSG00000075588.6	Hoxb2	7.19E-07	2.81E-05	4	6	213.25	49.33333333	163.9166667	2.335610155	2.432797245	Higher in Normal	330	235	174	114	13	22	32	38	67	124
ENSMUSG00000015619.10	Gata3	7.37E-07	2.87E-05	4	6	7058	1087.666667	5970.333333	2.575445438	2.708322218	Higher in Normal	4169	4934	8338	10791	639	515	896	624	3240	612
ENSMUSG00000061780.6	Cfd	7.46E-07	2.90E-05	4	6	82505	1990.833333	80514.16667	4.690337365	5.784047066	Higher in Normal	105606	221479	1154	1781	1394	471	2316	145	6216	1403
ENSMUSG00000024672.11	Ms4a7	7.61E-07	2.95E-05	4	6	375.5	5126.5	4751	2.990474342	3.21325306	Higher in TRIM24	337	691	193	281	3523	797	2175	3647	1689	18928
ENSMUSG00000035849.14	Krt222	7.67E-07	2.97E-05	4	6	95.75	19.83333333	75.91666667	2.419061279	2.528370029	Higher in Normal	165	106	65	47	11	8	17	11	36	36
ENSMUSG00000043913.14	Ccdc60	7.77E-07	3.00E-05	4	6	127.75	7.333333333	120.4166667	3.981227712	4.557062248	Higher in Normal	151	31	207	122	0	3	0	0	15	25
ENSMUSG00000071561.2	BC100530	7.82E-07	3.01E-05	4	6	2.5	492.1666667	489.6666667	5.125309972	7.500851765	Higher in TRIM24	2	0	3	5	497	3	3	12	2200	238
ENSMUSG00000034551.12	Hdx	7.82E-07	3.01E-05	4	6	166.75	18.66666667	148.0833333	3.006685235	3.227146048	Higher in Normal	71	31	345	220	4	9	25	7	33	34
ENSMUSG00000059857.15	Ntng1	7.89E-07	3.04E-05	4	6	1524.5	101.8333333	1422.666667	3.538907581	3.920218787	Higher in Normal	563	889	2304	2342	258	5	20	50	130	148
ENSMUSG00000049892.7	Rasd1	7.91E-07	3.04E-05	4	6	2016	146.6666667	1869.333333	3.589825347	3.990416427	Higher in Normal	2130	3465	1098	1371	187	17	212	15	382	67
ENSMUSG00000029534.17	St7	7.97E-07	3.06E-05	4	6	578	16948.33333	16370.33333	4.043176954	4.713409054	Higher in TRIM24	357	269	742	944	38992	515	10298	5686	578	45621
ENSMUSG00000074207.10	Adh1	7.97E-07	3.06E-05	4	6	1154.25	73.33333333	1080.916667	3.812336514	4.307646859	Higher in Normal	1559	2679	135	244	37	29	59	25	242	48
ENSMUSG00000029082.17	Bst1	8.11E-07	3.10E-05	4	6	221.75	1581.166667	1359.416667	2.750281919	2.919596708	Higher in TRIM24	90	110	314	373	2910	322	1205	1184	2595	1271
ENSMUSG00000028641.16	P3h1	8.21E-07	3.14E-05	4	6	606.25	3617.333333	3011.083333	2.429830333	2.543037666	Higher in TRIM24	586	545	620	674	1129	1510	5134	5663	1913	6355
ENSMUSG00000014846.12	Tppp3	8.22E-07	3.14E-05	4	6	3010	479.6666667	2530.333333	2.468602055	2.58581239	Higher in Normal	1316	1917	3508	5299	197	263	1014	337	643	424
ENSMUSG00000036596.6	Cpz	8.53E-07	3.25E-05	4	6	31.75	415	383.25	3.375036759	3.717692276	Higher in TRIM24	23	21	44	39	62	110	1291	184	624	219
ENSMUSG00000009376.15	Met	8.63E-07	3.28E-05	4	6	6114.5	162810.6667	156696.1667	3.913271767	4.507021964	Higher in TRIM24	4138	3937	8468	7915	287283	4478	186147	45194	6439	447323
ENSMUSG00000028341.9	Nr4a3	8.64E-07	3.28E-05	4	6	6425	165.8333333	6259.166667	4.353323721	5.17244984	Higher in Normal	153	46	13645	11856	46	94	371	82	158	244
ENSMUSG00000030144.4	Clec4d	8.81E-07	3.35E-05	4	6	128	1285.666667	1157.666667	2.836067668	3.024970988	Higher in TRIM24	69	66	160	217	158	372	1878	394	1157	3755
ENSMUSG00000025175.12	Fn3k	8.97E-07	3.40E-05	4	6	194	22	172	3.110677234	3.360389628	Higher in Normal	383	140	112	141	16	4	11	8	72	21
ENSMUSG00000095335.2	Igkv3-5	9.29E-07	3.51E-05	4	6	162.5	6.5	156	4.343686732	5.162185165	Higher in Normal	11	73	239	327	4	2	0	0	2	31
ENSMUSG00000027333.18	Smox	9.52E-07	3.58E-05	4	6	1087.5	6491.333333	5403.833333	2.545854435	2.679223941	Higher in TRIM24	475	482	1503	1890	12139	4266	2972	2965	9799	6807
ENSMUSG00000065408.1	Mir31	9.68E-07	3.63E-05	4	6	5.75	131.1666667	125.4166667	3.930505656	4.549880225	Higher in TRIM24	4	4	4	11	2	201	38	77	299	170
ENSMUSG00000031383.8	Dusp9	1.04E-06	3.87E-05	4	6	180.75	1394.166667	1213.416667	2.573522867	2.712717328	Higher in TRIM24	60	73	250	340	1322	534	1077	420	609	4403
ENSMUSG00000000739.14	Sult5a1	1.09E-06	4.02E-05	4	6	129.5	26.5	103	2.451150824	2.568672734	Higher in Normal	156	207	82	73	12	10	42	13	33	49
ENSMUSG00000112003.1	Gm4864	1.09E-06	4.03E-05	4	6	8	101.3333333	93.33333333	3.112818089	3.381577768	Higher in TRIM24	16	11	4	1	33	70	96	93	47	269
ENSMUSG00000016529.5	Il10	1.09E-06	4.03E-05	4	6	385.25	43.66666667	341.5833333	3.099458404	3.350378243	Higher in Normal	79	71	621	770	41	10	21	12	53	125
ENSMUSG00000034161.7	Scx	1.09E-06	4.04E-05	4	6	187.75	1715.833333	1528.083333	3.011191938	3.247132983	Higher in TRIM24	85	105	204	357	1642	784	1250	2805	207	3607
ENSMUSG00000023013.4	Aqp2	1.10E-06	4.06E-05	4	6	33.5	536	502.5	3.513059585	3.920217707	Higher in TRIM24	58	41	19	16	70	168	1552	629	200	597
ENSMUSG00000024621.15	Csf1r	1.12E-06	4.13E-05	4	6	2468	14128.16667	11660.16667	2.366653683	2.473547664	Higher in TRIM24	1929	3464	2055	2424	4908	12182	21763	16029	7387	22500
ENSMUSG00000081189.1	Hspd1-ps4	1.17E-06	4.27E-05	4	6	425.75	8.833333333	416.9166667	4.755122821	6.000673601	Higher in Normal	780	913	2	8	3	2	11	1	28	8
ENSMUSG00000039954.8	Stk32a	1.18E-06	4.29E-05	4	6	237.5	25.16666667	212.3333333	3.044921679	3.283823908	Higher in Normal	87	24	450	389	26	7	10	22	28	58
ENSMUSG00000067879.3	Vxn	1.18E-06	4.30E-05	4	6	60	1.833333333	58.16666667	4.174085966	4.94905645	Higher in Normal	7	85	70	78	0	6	0	1	1	3
ENSMUSG00000024042.6	Sik1	1.18E-06	4.30E-05	4	6	39757.25	2670.5	37086.75	3.569236875	3.977744305	Higher in Normal	2276	3721	78805	74227	1510	829	1988	897	5867	4932
ENSMUSG00000020310.9	Madcam1	1.21E-06	4.38E-05	4	6	323.5	17.16666667	306.3333333	3.609448255	4.037519238	Higher in Normal	102	43	461	688	42	1	18	16	11	15
ENSMUSG00000087646.1	Gm1667	1.22E-06	4.41E-05	4	6	16.75	0.5	16.25	4.19529525	5.032631207	Higher in Normal	8	14	27	18	2	0	0	0	1	0
ENSMUSG00000032014.6	Oaf	1.26E-06	4.54E-05	4	6	1185.25	7717.166667	6531.916667	2.491525652	2.619067487	Higher in TRIM24	1062	1506	896	1277	1376	5461	10149	9977	5350	13990
ENSMUSG00000101089.1	2610016A17Rik	1.27E-06	4.55E-05	4	6	561	10.16666667	550.8333333	4.783603458	6.08004907	Higher in Normal	1073	1160	6	5	5	2	5	7	40	2
ENSMUSG00000049625.5	Tifab	1.27E-06	4.55E-05	4	6	200.5	2000.833333	1800.333333	3.056481639	3.308556833	Higher in TRIM24	337	314	84	67	858	3035	3044	1559	1185	2324
ENSMUSG00000113909.1	Gm36377	1.27E-06	4.57E-05	4	6	2	75.33333333	73.33333333	4.397146432	5.462961952	Higher in TRIM24	4	1	2	1	3	36	74	260	10	69
ENSMUSG00000096146.2	Kcnj11	1.29E-06	4.62E-05	4	6	259.25	35.83333333	223.4166667	2.783833579	2.963357964	Higher in Normal	290	178	270	299	8	18	74	6	69	40
ENSMUSG00000089941.1	Gm16168	1.30E-06	4.66E-05	4	6	51.25	1	50.25	4.757844178	6.113724109	Higher in Normal	77	119	7	2	0	1	0	0	3	2
ENSMUSG00000028051.10	Hcn3	1.31E-06	4.68E-05	4	6	128.5	14.33333333	114.1666667	3.060363404	3.306564345	Higher in Normal	276	15	154	69	8	5	8	16	18	31
ENSMUSG00000005124.9	Wisp1	1.32E-06	4.69E-05	4	6	337.75	4475	4137.25	3.315893651	3.651706453	Higher in TRIM24	159	95	544	553	301	1213	8948	4080	2086	10222
ENSMUSG00000021095.5	Gsc	1.35E-06	4.81E-05	4	6	78	3.833333333	74.16666667	3.995729267	4.631689583	Higher in Normal	79	210	8	15	1	1	7	4	6	4
ENSMUSG00000018796.13	Acsl1	1.38E-06	4.88E-05	4	6	13664	1664.833333	11999.16667	3.014493442	3.248801632	Higher in Normal	24771	18812	5519	5554	1535	414	1762	742	4365	1171
ENSMUSG00000097324.8	Carmn	1.45E-06	5.09E-05	4	6	57.75	10.83333333	46.91666667	2.433986378	2.553154672	Higher in Normal	76	74	44	37	11	10	3	7	17	17
ENSMUSG00000022902.7	Stfa2	1.45E-06	5.11E-05	4	6	1.25	79	77.75	4.410019606	5.535751281	Higher in TRIM24	2	1	2	0	51	5	4	8	228	178
ENSMUSG00000085095.2	Gm15635	1.48E-06	5.21E-05	4	6	56.75	8.666666667	48.08333333	2.662620844	2.822685244	Higher in Normal	66	68	56	37	5	12	1	7	10	17
ENSMUSG00000050063.18	Klk6	1.49E-06	5.22E-05	4	6	19	6105.166667	6086.166667	5.20657999	8.695639174	Higher in TRIM24	34	7	19	16	546	22050	7	13	13932	83

ENSMUSG00000034205.16	Loxl2	1.59E-06	5.53E-05	4	6	834.5	11057.5	10223	3.137738837	3.419825363	Higher in TRIM24	1321	1363	270	384	1866	4250	16483	12320	4724	26702
ENSMUSG00000086555.2	Gm13446	1.61E-06	5.60E-05	4	6	23.5	2.333333333	21.16666667	3.104956502	3.373953423	Higher in Normal	23	6	34	31	1	1	3	1	5	3
ENSMUSG00000029838.11	Ptn	1.63E-06	5.67E-05	4	6	13375.75	1717.166667	11658.58333	2.721107774	2.89151683	Higher in Normal	10106	13713	12514	17170	1152	295	2084	3649	1289	1834
ENSMUSG00000028427.13	Aqp7	1.71E-06	5.92E-05	4	6	1990.75	96.33333333	1894.416667	4.194670472	4.962818751	Higher in Normal	3124	3876	454	509	70	6	12	2	282	206
ENSMUSG00000028024.14	Enpep	1.74E-06	5.99E-05	4	6	1165.25	132.8333333	1032.416667	3.130346647	3.402094134	Higher in Normal	3427	601	332	301	78	68	102	51	267	231
ENSMUSG00000045573.9	Penk	1.74E-06	6.00E-05	4	6	1071.75	206.6666667	865.0833333	2.50181136	2.632627679	Higher in Normal	846	1778	690	973	47	61	326	172	254	380
ENSMUSG00000028399.18	Ptprd	1.78E-06	6.12E-05	4	6	1339.25	235	1104.25	2.698089842	2.865223753	Higher in Normal	2171	2045	607	534	172	53	261	81	425	418
ENSMUSG00000071036.2	Gm10309	1.82E-06	6.24E-05	4	6	42	3.166666667	38.83333333	3.454158643	3.841844646	Higher in Normal	52	7	61	48	0	3	3	0	7	6
ENSMUSG00000069421.4	Olfr810	1.89E-06	6.46E-05	4	6	6.5	243.8333333	237.3333333	4.25955039	5.213439367	Higher in TRIM24	11	14	0	1	8	348	352	370	117	268
ENSMUSG00000085558.7	4930412C18Rik	1.90E-06	6.47E-05	4	6	87.75	8	79.75	3.416811627	3.786816199	Higher in Normal	147	123	43	38	7	2	5	0	23	11
ENSMUSG00000046341.5	Gm11223	1.90E-06	6.47E-05	4	6	22.25	161	138.75	2.707916819	2.882684878	Higher in TRIM24	33	12	19	25	40	89	227	270	83	257
ENSMUSG00000031725.8	Ces1f	1.92E-06	6.50E-05	4	6	1983.25	54.83333333	1928.416667	4.605594203	5.756550502	Higher in Normal	3912	3938	39	44	32	4	12	3	181	97
ENSMUSG00000027469.16	Tpx2	1.94E-06	6.54E-05	4	6	646	3790.166667	3144.166667	2.381019102	2.49562059	Higher in TRIM24	401	127	979	1077	3734	970	3932	3106	2444	8555
ENSMUSG00000102697.1	Pcdhac2	1.96E-06	6.61E-05	4	6	56	4.5	51.5	3.393377599	3.757605022	Higher in Normal	71	24	86	43	0	1	7	1	14	4
ENSMUSG00000109145.1	Gm20744	1.97E-06	6.63E-05	4	6	1.25	31.33333333	30.08333333	3.725548613	4.294866675	Higher in TRIM24	1	1	3	0	21	17	12	3	41	94
ENSMUSG00000033849.3	B3galt2	1.99E-06	6.69E-05	4	6	2124.25	76.66666667	2047.583333	4.269654112	5.109760973	Higher in Normal	4585	3709	102	101	61	32	76	3	234	54
ENSMUSG00000034842.16	Art3	2.01E-06	6.75E-05	4	6	3437.25	272.3333333	3164.916667	3.527248547	3.941712728	Higher in Normal	7039	5866	383	461	325	83	194	125	608	299
ENSMUSG00000085890.7	Tnfsf13os	2.05E-06	6.87E-05	4	6	94.75	12.83333333	81.91666667	2.696657534	2.867024835	Higher in Normal	51	67	110	151	21	12	4	2	23	15
ENSMUSG00000040896.16	Kcnd3	2.09E-06	6.97E-05	4	6	69	9.833333333	59.16666667	2.952272163	3.178462288	Higher in Normal	102	31	93	50	4	5	4	0	15	31
ENSMUSG00000034936.2	Arl4d	2.10E-06	7.00E-05	4	6	2719	306	2413	3.217159541	3.520577281	Higher in Normal	487	483	4076	5830	68	52	235	103	262	1116
ENSMUSG00000096326.2	Ighv1-78	2.12E-06	7.08E-05	4	6	24	0.833333333	23.16666667	4.223625807	5.123905432	Higher in Normal	33	47	8	8	2	1	0	0	0	2
ENSMUSG00000086712.2	AI427809	2.13E-06	7.08E-05	4	6	42	270.8333333	228.8333333	2.345741841	2.456463889	Higher in TRIM24	62	65	22	19	249	148	386	102	265	475
ENSMUSG00000044103.4	Il1f9	2.13E-06	7.08E-05	4	6	8.25	162.5	154.25	3.615766357	4.105539144	Higher in TRIM24	21	1	4	7	35	39	282	28	418	173
ENSMUSG00000040133.2	Gpr176	2.14E-06	7.12E-05	4	6	130.5	1028.333333	897.8333333	2.729010199	2.910004041	Higher in TRIM24	156	73	127	166	121	386	1861	1228	657	1917
ENSMUSG00000040653.6	Ppp1r14c	2.17E-06	7.20E-05	4	6	17.5	534.3333333	516.8333333	3.925497213	4.601908608	Higher in TRIM24	9	35	10	16	6	142	1486	100	520	952
ENSMUSG00000082433.2	Gm9025	2.19E-06	7.24E-05	4	6	32.25	272.6666667	240.4166667	2.770659929	2.961925946	Higher in TRIM24	84	14	18	13	147	179	367	264	136	543
ENSMUSG00000105565.1	Gm43566	2.19E-06	7.25E-05	4	6	24	183.1666667	159.1666667	2.696473454	2.871323148	Higher in TRIM24	28	22	22	24	189	18	284	228	105	275
ENSMUSG00000037196.6	Pacrg	2.26E-06	7.45E-05	4	6	31.5	5.666666667	25.83333333	2.439842946	2.564588913	Higher in Normal	40	14	27	45	1	3	7	5	8	10
ENSMUSG00000109807.1	Gm45244	2.27E-06	7.48E-05	4	6	163	13.83333333	149.1666667	3.609322403	4.065743018	Higher in Normal	296	312	15	29	5	4	6	8	24	36
ENSMUSG00000091370.1	5730435O14Rik	2.31E-06	7.56E-05	4	6	19.25	2.666666667	16.58333333	2.844788204	3.050482129	Higher in Normal	11	20	17	29	3	1	1	1	3	7
ENSMUSG00000066058.11	Cldn19	2.33E-06	7.61E-05	4	6	99.75	11	88.75	3.413373043	3.785274138	Higher in Normal	87	96	100	116	2	2	0	2	15	45
ENSMUSG00000032796.7	Lama1	2.33E-06	7.61E-05	4	6	3145.25	296.3333333	2848.916667	3.002961412	3.24663496	Higher in Normal	859	476	6302	4944	512	152	413	95	352	254
ENSMUSG00000050368.4	Hoxd10	2.35E-06	7.66E-05	4	6	185.25	22.16666667	163.0833333	2.874047355	3.08535844	Higher in Normal	131	88	218	304	6	8	66	9	12	32
ENSMUSG00000021416.11	Eci3	2.35E-06	7.66E-05	4	6	297.75	27.83333333	269.9166667	3.258488244	3.580149891	Higher in Normal	508	470	89	124	41	3	14	30	56	23
ENSMUSG00000035431.4	Sstr1	2.38E-06	7.73E-05	4	6	32.5	2	30.5	3.752129174	4.284962169	Higher in Normal	53	11	31	35	1	0	0	0	7	4
ENSMUSG00000062380.4	Tubb3	2.43E-06	7.87E-05	4	6	39.75	388	348.25	2.829222661	3.036265332	Higher in TRIM24	74	19	37	29	35	151	453	192	739	758
ENSMUSG00000108236.1	0610033M10Rik	2.43E-06	7.87E-05	4	6	44	6.666666667	37.33333333	2.829508709	3.030587323	Higher in Normal	47	52	40	37	3	5	2	0	14	16
ENSMUSG00000085881.2	Gm15912	2.45E-06	7.93E-05	4	6	50.75	6.666666667	44.08333333	3.009470171	3.255830999	Higher in Normal	65	67	28	43	2	0	11	2	12	13
ENSMUSG00000027715.9	Ccna2	2.50E-06	8.05E-05	4	6	585	3292	2707	2.3957819	2.515570611	Higher in TRIM24	337	126	890	987	2595	909	3705	3941	2325	6277
ENSMUSG00000096719.1	Mrgpra2b	2.55E-06	8.18E-05	4	6	1	26.83333333	25.83333333	3.918284113	4.633559747	Higher in TRIM24	0	0	2	2	20	3	37	7	60	34
ENSMUSG00000054555.11	Adam12	2.56E-06	8.21E-05	4	6	492.75	3683	3190.25	2.863900878	3.080577358	Higher in TRIM24	664	420	512	375	612	6872	4574	3874	2836	3330
ENSMUSG00000042302.14	Ehbp1	2.60E-06	8.29E-05	4	6	4084.5	683.5	3401	2.500503794	2.636101378	Higher in Normal	928	941	7572	6897	355	374	742	392	665	1573
ENSMUSG00000026042.16	Col5a2	2.62E-06	8.36E-05	4	6	3521.75	27208	23686.25	2.730980628	2.916049516	Higher in TRIM24	4020	4389	2721	2957	5245	25966	66919	13297	24292	27529
ENSMUSG00000032131.16	Abcg4	2.63E-06	8.38E-05	4	6	50	10.5	39.5	2.388200245	2.504897645	Higher in Normal	84	24	54	38	3	4	13	5	10	28
ENSMUSG00000024883.6	Rin1	2.68E-06	8.52E-05	4	6	333	1863.666667	1530.666667	2.558611174	2.70807164	Higher in TRIM24	199	149	477	507	855	3606	1731	903	2099	1988
ENSMUSG00000014030.15	Pax5	2.76E-06	8.77E-05	4	6	285	44.66666667	240.3333333	2.93224651	3.160729043	Higher in Normal	193	435	276	236	4	7	53	12	65	127
ENSMUSG00000030050.4	Gkn1	2.79E-06	8.84E-05	4	6	1.25	24.33333333	23.08333333	3.654646755	4.209599969	Higher in TRIM24	2	1	0	2	5	9	37	35	16	44
ENSMUSG00000032291.8	Crabp1	2.84E-06	8.99E-05	4	6	226.75	4450.5	4223.75	3.665727585	4.195881716	Higher in TRIM24	142	258	187	320	64	1748	12838	3476	1186	7391
ENSMUSG00000030862.13	Cpxm2	2.93E-06	9.24E-05	4	6	505	4916.666667	4411.666667	3.092896797	3.378960883	Higher in TRIM24	233	236	648	903	580	3339	14080	2085	5625	3791
ENSMUSG00000021090.16	Lrrc9	2.93E-06	9.24E-05	4	6	70.75	7.666666667	63.08333333	3.259286563	3.584480745	Higher in Normal	71	28	112	72	2	1	3	0	18	22
ENSMUSG00000053746.8	Ptrh1	2.93E-06	9.24E-05	4	6	119.5	691.5	572	2.411232599	2.535593046	Higher in TRIM24	119	110	106	143	139	1047	711	456	519	1277
ENSMUSG00000079298.9	Klrb1b	2.96E-06	9.30E-05	4	6	46	400.1666667	354.1666667	2.887156548	3.113645374	Higher in TRIM24	44	53	30	57	46	422	912	250	264	507
ENSMUSG00000103254.1	Ighv1-15	3.03E-06	9.48E-05	4	6	20.75	1.333333333	19.41666667	3.691908168	4.233850164	Higher in Normal	6	48	11	18	1	1	1	1	1	3
ENSMUSG00000063018.6	2010204K13Rik	3.05E-06	9.53E-05	4	6	74.75	454.1666667	379.4166667	2.400536588	2.523404536	Higher in TRIM24	43	35	83	138	560	90	261	493	246	1075
ENSMUSG00000009356.12	Lpo	3.07E-06	9.57E-05	4	6	106.25	7.5	98.75	3.374111571	3.748929799	Higher in Normal	52	14	163	196	11	4	3	1	21	5
ENSMUSG00000048013.9	Krt35	3.07E-06	9.58E-05	4	6	126	1.166666667	124.8333333	5.002595893	7.031465415	Higher in Normal	0	3	209	292	0	1	0	0	1	5
ENSMUSG00000056895.4	Hist3h2ba	3.08E-06	9.58E-05	4	6	109.75	5.666666667	104.0833333	3.598723033	4.074905128	Higher in Normal	15	81	135	208	1	1	5	17	6	4
ENSMUSG00000004558.14	Ndrg2	3.14E-06	9.75E-05	4	6	6203.75	888.1666667	5315.583333	2.822315993	3.026852211	Higher in Normal	7059	11893	2513	3350	268	449	1703	413	1605	891
ENSMUSG00000032946.16	Rasgrp2	3.15E-06	9.80E-05	4	6	1439.25	231	1208.25	2.82297448	3.027775757	Higher in Normal	2434	2410	484	429	107	85	241	78	440	435
ENSMUSG00000006642.3	Tcf23	3.19E-06	9.89E-05	4	6	23.5	4.166666667	19.33333333	2.592108367	2.745768169	Higher in Normal	23	15	31	25	1	1	2	2	7	12
ENSMUSG00000029095.17	Ablim2	3.22E-06	9.96E-05	4	6	353	50.33333333	302.6666667	2.872805513	3.090179177	Higher in Normal	589	438	187	198	15	18	72	10	125	62
ENSMUSG00000050473.5	Slc35d3	3.25E-06	0.000100356	4	6	58.75	10.83333333	47.91666667	2.439676546	2.567263469	Higher in Normal	39	13	89	94	8	6	8	3	13	27
ENSMUSG00000000204.16	Slfn4	3.34E-06	0.000102779	4	6	74.5	1151	1076.5	3.341998724	3.7247706	Higher in TRIM24	101	84	62	51	1055	237	145	334	4435	700
ENSMUSG00000045284.9	Dcaf12l1	3.35E-06	0.000103123	4	6	190.5	26.33333333	164.1666667	2.75177696	2.941080913	Higher in Normal	105	63	270	324	7	7	50	7	48	39
ENSMUSG00000110071.1	Gm45512	3.37E-06	0.000103667	4	6	29.25	0.666666667	28.58333333	4.334196111	5.421027584	Higher in Normal	17	44	27	29	4	0	0	0	0	0
ENSMUSG00000006398.15	Cdc20	3.44E-06	0.000105471	4	6	465.25	2817	2351.75	2.456983967	2.591134034	Higher in TRIM24	223	105	629	904	2155	582	2728	3299	2511	5627
ENSMUSG00000051951.5	Xkr4	3.45E-06	0.000105751	4	6	47.75	5	42.75	2.964958371	3.212387793	Higher in Normal	32	20	102	37	1	6	8	1	9	5
ENSMUSG00000101925.1	Gm28156	3.46E-06	0.000105835	4	6	268.75	19.16666667	249.5833333	3.848683473	4.45264451	Higher in Normal	380	662	14	19	8	8	13	4	19	63
ENSMUSG00000038894.7	Irs2	3.48E-06	0.000106499	4	6	13775	1296.666667	12478.33333	3.105534069	3.389546762	Higher in Normal	1140	1388	28972	23600	1056	609	1061	899	1606	2549
ENSMUSG00000038179.13	Slamf7	3.53E-06	0.000107879	4	6	283.75	61.83333333	221.9166667	2.359968793	2.475734854	Higher in Normal	120	167	487	361	29	9	57	21	98	157
ENSMUSG00000026879.14	Gsn	3.55E-06	0.000108096	4	6	139368.75	15773	123595.75	3.133077807	3.426245975	Higher in Normal	197275	303327	24439	32434	4944	11003	21565	14182	12546	30398
ENSMUSG00000075589.3	Gm11536	3.57E-06	0.000108463	4	6	40	4.5	35.5	2.958089963	3.204347267	Higher in Normal	38	45	39	38	4	2	7	1	12	1
ENSMUSG00000027559.5	Car3	3.71E-06	0.000112207	4	6	174804.75	4586.166667	170218.5833	4.487589479	5.618595413	Higher in Normal	372616	320598	2639	3366	3890	746	3119	228	17463	2071
ENSMUSG00000112129.1	Pbld1	3.73E-06	0.000112551	4	6	261.5	24.66666667	236.8333333	3.496299731	3.925587775	Higher in Normal	439	279	176	152	9	6	2	1	66	64
ENSMUSG00000076555.3	Igkv4-57-1	3.74E-06	0.00011265	4	6	123.25	8.833333333	114.4166667	3.47314849	3.896198761	Higher in Normal	21	105	145	222	10	0	18	6	1	18
ENSMUSG00000071716.4	Apol7e	3.77E-06	0.000113443	4	6	41.25	5.833333333	35.41666667	2.900934704	3.126764089	Higher in Normal	27	25	75	38	6	1	2	0	8	18
ENSMUSG00000022491.4	Glycam1	3.78E-06	0.000113683	4	6	7219	81.83333333	7137.166667	4.645898982	5.982082749	Higher in Normal	615	17	10974	17270	8	257	36	5	164	21
ENSMUSG00000003123.15	Lipe	3.79E-06	0.000113828	4	6	12861.25	1152.166667	11709.08333	3.405755	3.798817375	Higher in Normal	20275	26483	2042	2645	1180	345	708	374	3323	983
ENSMUSG00000085295.7	4930430E12Rik	3.80E-06	0.00011383	4	6	17.25	166.8333333	149.5833333	2.796450229	3.005085563	Higher in TRIM24	37	3	8	21	161	60	47	119	119	495
ENSMUSG00000035615.12	Frmpd1	3.83E-06	0.000114718	4	6	279	25.5	253.5	3.411439167	3.806403534	Higher in Normal	175	158	409	374	25	0	13	1	46	68
ENSMUSG00000026399.12	Cd55	3.89E-06	0.000115961	4	6	1960.25	314.1666667	1646.083333	2.538936135	2.687139509	Higher in Normal	1314	1536	2442	2549	170	197	395	58	851	214
ENSMUSG00000079597.1	Gm5483	3.90E-06	0.000115998	4	6	3.5	332	328.5	4.429988357	5.734405008	Higher in TRIM24	13	0	0	1	37	24	42	47	326	1516

ENSMUSG00000045569.6	Mc2r	3.99E-06	0.00011833	4	6	460.5	16.33333333	444.1666667	4.373262962	5.396126244	Higher in Normal	827	998	9	8	7	1	6	4	48	32
ENSMUSG00000048087.6	Gm4737	4.00E-06	0.000118555	4	6	111	1039.5	928.5	2.692906725	2.877580123	Higher in TRIM24	261	111	28	44	376	496	1037	693	945	2690
ENSMUSG00000042256.4	Ptchd4	4.02E-06	0.00011888	4	6	113.25	15.5	97.75	3.147993461	3.44828639	Higher in Normal	152	116	110	75	3	1	2	12	11	64
ENSMUSG00000038216.7	Pnmt	4.10E-06	0.000120475	4	6	47.25	0.666666667	46.58333333	4.756411631	6.321128097	Higher in Normal	1	4	77	107	0	0	1	0	0	3
ENSMUSG00000022878.5	Adipoq	4.11E-06	0.000120847	4	6	28941.25	730.1666667	28211.08333	4.495079493	5.653138052	Higher in Normal	55562	59374	343	486	887	133	403	42	2618	298
ENSMUSG00000031775.5	Pllp	4.13E-06	0.000121342	4	6	24.75	3.666666667	21.08333333	2.76520741	2.965390319	Higher in Normal	20	39	19	21	1	2	2	3	8	6
ENSMUSG00000029375.6	Cxcl15	4.15E-06	0.000121684	4	6	1297.75	60.83333333	1236.916667	3.761980288	4.32939224	Higher in Normal	675	1272	1381	1863	218	10	56	1	54	26
ENSMUSG00000024973.16	Hrasls5	4.18E-06	0.000122458	4	6	43	2.666666667	40.33333333	3.487354032	3.935270823	Higher in Normal	34	29	61	48	3	3	0	1	9	0
ENSMUSG00000024892.15	Pcx	4.23E-06	0.000123791	4	6	12111.75	1252	10859.75	3.130467229	3.428397947	Higher in Normal	29540	8909	4740	5258	1327	195	769	847	3628	746
ENSMUSG00000103768.1	Gm37856	4.30E-06	0.000125532	4	6	20	0.5	19.5	4.430844707	5.570781232	Higher in Normal	35	35	2	8	0	0	1	0	1	1
ENSMUSG00000042401.6	Crtac1	4.31E-06	0.000125741	4	6	272.25	15.5	256.75	4.006116267	4.729920269	Higher in Normal	314	752	16	7	12	2	12	5	21	41
ENSMUSG00000005087.17	Cd44	4.35E-06	0.000126585	4	6	4551	25820.83333	21269.83333	2.479871806	2.621527583	Higher in TRIM24	1294	1973	7574	7363	9924	34916	31897	11971	27197	39020
ENSMUSG00000025746.11	Il6	4.45E-06	0.000128871	4	6	3301.75	97.33333333	3204.416667	4.165224442	5.011728694	Higher in Normal	47	9	6063	7088	37	62	61	102	125	197
ENSMUSG00000047298.3	Kcnv2	4.49E-06	0.000129819	4	6	24	1.666666667	22.33333333	3.590713773	4.087582433	Higher in Normal	40	9	39	8	2	1	0	0	2	5
ENSMUSG00000028539.14	Artn	4.53E-06	0.000131057	4	6	24	532.5	508.5	3.531187719	4.01863266	Higher in TRIM24	17	14	37	28	15	19	595	313	327	1926
ENSMUSG00000086604.2	Gm15510	4.55E-06	0.000131512	4	6	35.25	5.333333333	29.91666667	2.840719348	3.05900919	Higher in Normal	19	68	16	38	3	1	4	4	7	13
ENSMUSG00000041523.3	Upk2	4.59E-06	0.000132314	4	6	54.75	8.333333333	46.41666667	2.577053465	2.736591667	Higher in Normal	45	52	55	67	9	2	21	3	9	6
ENSMUSG00000079092.4	Prl2c2	4.63E-06	0.000133223	4	6	11.5	891.1666667	879.6666667	4.398037602	5.675231828	Higher in TRIM24	38	3	3	2	287	13	236	8	2418	2385
ENSMUSG00000035184.15	Fam124a	4.67E-06	0.000133879	4	6	309	39.5	269.5	2.922714892	3.16245283	Higher in Normal	277	659	122	178	18	8	64	42	72	33
ENSMUSG00000045281.5	Gpr20	4.68E-06	0.000134018	4	6	90	5.666666667	84.33333333	3.425028162	3.843065287	Higher in Normal	10	29	150	171	3	2	11	9	1	8
ENSMUSG00000047443.14	Erfe	4.77E-06	0.000136193	4	6	67.75	782.6666667	714.9166667	3.203621623	3.545743752	Higher in TRIM24	56	7	99	109	134	436	829	1214	144	1939
ENSMUSG00000028681.11	Ptch2	4.78E-06	0.000136193	4	6	485.25	59	426.25	3.039180926	3.312745946	Higher in Normal	805	924	121	91	58	30	56	22	117	71
ENSMUSG00000023945.6	Slc5a7	4.78E-06	0.000136193	4	6	627.75	28.33333333	599.4166667	4.092925946	4.88879238	Higher in Normal	102	529	1066	814	4	2	4	0	99	61
ENSMUSG00000041624.10	Gucy1a2	4.80E-06	0.000136615	4	6	186	32.5	153.5	2.495927926	2.638859553	Higher in Normal	120	52	359	213	15	8	26	14	80	52
ENSMUSG00000049988.4	Lrrc25	4.81E-06	0.00013682	4	6	179	1063.5	884.5	2.326515643	2.442926089	Higher in TRIM24	159	331	94	132	484	698	1614	1198	797	1590
ENSMUSG00000026818.5	Cel	4.83E-06	0.000137072	4	6	2090.75	69.16666667	2021.583333	4.114206747	4.928718982	Higher in Normal	1120	42	3109	4092	22	24	29	5	324	11
ENSMUSG00000032122.15	Slc37a2	4.83E-06	0.000137072	4	6	284	1918	1634	2.346484644	2.46614534	Higher in TRIM24	276	437	209	214	812	807	1373	2389	739	5388
ENSMUSG00000052749.9	Trim30b	4.90E-06	0.000138622	4	6	19.75	133.5	113.75	2.433237484	2.568936957	Higher in TRIM24	23	28	17	11	141	50	82	55	333	140
ENSMUSG00000064080.12	Fbln2	5.14E-06	0.000144463	4	6	5929.5	37497.83333	31568.33333	2.51530695	2.666321094	Higher in TRIM24	2504	3322	8555	9337	8868	40972	60614	19539	16503	78491
ENSMUSG00000026697.10	Myoc	5.17E-06	0.000145144	4	6	37.75	4.833333333	32.91666667	2.935576484	3.181454232	Higher in Normal	15	48	45	43	3	0	6	1	12	7
ENSMUSG00000031595.9	Pdgfrl	5.28E-06	0.00014804	4	6	304	1651.333333	1347.333333	2.346195956	2.466828344	Higher in TRIM24	249	183	339	445	486	421	2590	2396	2023	1992
ENSMUSG00000076646.3	Ighv2-6-8	5.30E-06	0.000148435	4	6	17.75	1	16.75	3.777948329	4.394656333	Higher in Normal	22	4	23	22	1	0	1	0	0	4
ENSMUSG00000038418.7	Egr1	5.34E-06	0.000149458	4	6	48145.5	3821	44324.5	3.236226538	3.578602936	Higher in Normal	2709	3678	89257	96938	3153	1835	3802	2726	5304	6106
ENSMUSG00000072849.10	Serpina1e	5.38E-06	0.000150253	4	6	21	0.833333333	20.16666667	3.984374344	4.772010118	Higher in Normal	37	12	15	20	0	0	1	0	4	0
ENSMUSG00000040794.5	C1qtnf4	5.38E-06	0.000150253	4	6	125.5	20	105.5	2.546393785	2.701656978	Higher in Normal	83	162	88	169	7	5	38	22	32	16
ENSMUSG00000070419.11	Cyp3a57	5.50E-06	0.000152958	4	6	277.75	18.83333333	258.9166667	3.646166374	4.170784913	Higher in Normal	267	651	115	78	2	2	51	8	37	13
ENSMUSG00000020787.14	P2rx1	5.51E-06	0.000153312	4	6	104.5	15.5	89	2.62185367	2.792460235	Higher in Normal	56	72	131	159	8	7	23	3	41	11
ENSMUSG00000025006.15	Sorbs1	5.60E-06	0.00015539	4	6	2176	305.5	1870.5	2.769094247	2.97315788	Higher in Normal	3666	1637	2222	1179	439	103	150	57	828	256
ENSMUSG00000047261.9	Gap43	5.62E-06	0.000155869	4	6	27.5	1978.333333	1950.833333	4.29083409	5.450726279	Higher in TRIM24	69	26	10	5	8	21	2014	970	614	8243
ENSMUSG00000112163.1	Gm8188	5.68E-06	0.00015739	4	6	2.5	23.83333333	21.33333333	2.844601257	3.081695522	Higher in TRIM24	4	2	3	1	14	24	22	6	21	56
ENSMUSG00000006269.7	Atp6v1b1	5.89E-06	0.000162463	4	6	12009.75	511.1666667	11498.58333	3.971965811	4.699787195	Higher in Normal	3875	6698	16599	20867	253	34	146	14	2481	139
ENSMUSG00000048376.6	F2r	5.90E-06	0.000162525	4	6	788.25	4959.5	4171.25	2.351018012	2.473847212	Higher in TRIM24	424	324	1141	1264	2331	2031	6838	2911	1297	14349
ENSMUSG00000022385.10	Gtse1	5.99E-06	0.000164639	4	6	189	1043	854	2.495196893	2.644611693	Higher in TRIM24	86	50	296	324	2184	724	865	636	666	1183
ENSMUSG00000056596.8	Trnp1	6.05E-06	0.000166242	4	6	408.75	42	366.75	2.994184721	3.261042827	Higher in Normal	87	206	547	795	45	11	34	14	128	20
ENSMUSG00000019899.16	Lama2	6.74E-06	0.000182648	4	6	2095	358.5	1736.5	2.340890573	2.461709469	Higher in Normal	1528	952	3527	2373	119	367	705	136	463	361
ENSMUSG00000092164.2	Rergl	6.82E-06	0.000184514	4	6	17.25	1	16.25	3.877001442	4.560703862	Higher in Normal	16	17	10	26	0	0	0	0	1	5
ENSMUSG00000026692.12	Fmo4	6.91E-06	0.00018656	4	6	43.5	5.166666667	38.33333333	3.126113049	3.440255401	Higher in Normal	71	46	26	31	0	4	4	0	9	14
ENSMUSG00000021238.11	Aldh6a1	7.15E-06	0.000192111	4	6	6957	1017.5	5939.5	2.851410461	3.082262065	Higher in Normal	11734	12379	1851	1864	1088	383	642	408	2174	1410
ENSMUSG00000030607.7	Acan	7.29E-06	0.000195191	4	6	14.25	636.6666667	622.4166667	4.32444979	5.596093215	Higher in TRIM24	27	13	13	4	0	375	256	2099	204	886
ENSMUSG00000039628.8	Hs3st6	7.43E-06	0.000198721	4	6	142.75	12.83333333	129.9166667	3.159761252	3.489166527	Higher in Normal	89	179	117	186	6	1	36	10	21	3
ENSMUSG00000094230.5	Gm21847	7.44E-06	0.00019878	4	6	13.5	0.333333333	13.16666667	4.329078126	5.362457642	Higher in Normal	24	9	15	6	0	0	0	0	0	2
ENSMUSG00000090582.1	Gm17024	7.65E-06	0.00020391	4	6	773	41.33333333	731.6666667	3.793640747	4.426197535	Higher in Normal	50	17	1958	1067	31	21	9	4	59	124
ENSMUSG00000020916.8	Krt36	7.71E-06	0.000205262	4	6	36	1.666666667	34.33333333	3.993761306	4.793755296	Higher in Normal	48	69	15	12	0	1	0	0	7	2
ENSMUSG00000038402.2	Foxf2	7.89E-06	0.000209512	4	6	13.5	434.3333333	420.8333333	3.920382114	4.727429807	Higher in TRIM24	27	11	13	3	15	13	511	754	66	1247
ENSMUSG00000053046.15	Brsk2	7.90E-06	0.00020955	4	6	37.25	5.5	31.75	2.889696965	3.133572967	Higher in Normal	73	35	21	20	3	1	1	4	8	16
ENSMUSG00000050625.4	Ccdc121	7.93E-06	0.000209981	4	6	36.25	3.166666667	33.08333333	3.397509417	3.821797291	Higher in Normal	6	31	54	54	0	0	3	1	6	9
ENSMUSG00000079293.11	Clec7a	7.94E-06	0.000209996	4	6	570	3958.833333	3388.833333	2.768721217	2.987008586	Higher in TRIM24	203	362	741	974	1630	7464	6300	1005	2450	4904
ENSMUSG00000064023.4	Klk8	7.95E-06	0.000209996	4	6	96	4523	4427	4.458155316	5.990094185	Higher in TRIM24	63	125	85	111	387	20192	100	128	6029	302
ENSMUSG00000025163.6	Cd7	7.95E-06	0.000209996	4	6	160.5	33.83333333	126.6666667	2.365504861	2.492381032	Higher in Normal	129	127	163	223	8	3	25	37	46	84
ENSMUSG00000040037.13	Negr1	8.17E-06	0.000215363	4	6	596.25	49	547.25	3.513626109	3.994614085	Higher in Normal	1317	952	70	46	55	11	29	12	107	80
ENSMUSG00000026494.12	Kif26b	8.25E-06	0.000217219	4	6	273.5	2344.833333	2071.333333	2.748386916	2.962167294	Higher in TRIM24	90	109	550	345	210	1387	2863	1728	1361	6520
ENSMUSG00000076583.1	Igkv8-24	8.45E-06	0.000221929	4	6	176.75	6	170.75	4.026271702	4.851688319	Higher in Normal	15	539	67	86	6	17	5	0	4	4
ENSMUSG00000076606.3	Igkj3	8.52E-06	0.000223303	4	6	12.75	0.833333333	11.91666667	3.545728533	4.081204661	Higher in Normal	12	11	16	12	1	0	0	0	3	1
ENSMUSG00000027674.16	Pex5l	8.74E-06	0.000228629	4	6	56.5	9.833333333	46.66666667	2.732097396	2.934714105	Higher in Normal	64	42	68	52	1	3	5	1	19	30
ENSMUSG00000031149.7	Praf2	8.84E-06	0.000230789	4	6	179.5	1219	1039.5	2.448060314	2.59456589	Higher in TRIM24	180	187	125	226	257	428	1418	1751	492	2968
ENSMUSG00000089652.1	Gm16025	8.96E-06	0.000233484	4	6	32.25	5.833333333	26.41666667	2.566685819	2.73209209	Higher in Normal	25	18	42	44	2	2	2	1	12	16
ENSMUSG00000081665.2	Gm15922	8.99E-06	0.000234071	4	6	12	77.5	65.5	2.525787903	2.688843064	Higher in TRIM24	10	7	27	4	33	80	98	28	128	98
ENSMUSG00000022780.5	Meltf	9.01E-06	0.000234463	4	6	1013.75	46.33333333	967.4166667	3.829176781	4.499189043	Higher in Normal	413	240	1622	1780	65	3	9	1	175	25
ENSMUSG00000038295.14	Atg9b	9.05E-06	0.000235322	4	6	97.75	2497.333333	2399.583333	4.08360365	5.071252856	Higher in TRIM24	170	30	104	87	742	9188	171	91	4284	508
ENSMUSG00000052684.4	Jun	9.16E-06	0.000237618	4	6	122807.5	6760	116047.5	3.627141255	4.174659289	Higher in Normal	2285	2990	222600	263355	3534	3676	7012	3908	8244	14186
ENSMUSG00000039620.17	Trmt9b	9.17E-06	0.000237618	4	6	311	43.33333333	267.6666667	2.796207655	3.01854603	Higher in Normal	334	280	323	307	46	4	29	10	137	34
ENSMUSG00000004110.15	Cacna1e	9.78E-06	0.000251671	4	6	337	46.16666667	290.8333333	3.176133656	3.520052017	Higher in Normal	387	693	197	71	21	8	20	4	82	142
ENSMUSG00000053199.13	Arhgap20	9.80E-06	0.000251671	4	6	256.5	42.33333333	214.1666667	2.856494016	3.096405958	Higher in Normal	466	195	200	165	21	10	17	2	79	125
ENSMUSG00000026628.13	Atf3	9.80E-06	0.000251671	4	6	45401.25	1741.5	43659.75	3.960375307	4.733274598	Higher in Normal	474	390	78284	102457	1262	1597	1329	322	1188	4751
ENSMUSG00000038963.15	Slco4a1	9.80E-06	0.000251671	4	6	45.5	313.5	268	2.696094833	2.900475234	Higher in TRIM24	50	11	55	66	111	183	167	690	163	567
ENSMUSG00000113682.1	Gm48630	9.82E-06	0.000251811	4	6	11.5	0.166666667	11.33333333	4.284867249	5.430937113	Higher in Normal	9	6	24	7	0	0	0	0	1	0
ENSMUSG00000108658.1		9.83E-06	0.000251884	4	6	348.75	65	283.75	2.34906598	2.476519204	Higher in Normal	337	601	192	265	77	70	41	34	117	51
ENSMUSG00000058715.11	Fcer1g	9.86E-06	0.000252542	4	6	785	5577	4792	2.438426316	2.584772905	Higher in TRIM24	636	1760	272	472	2752	4127	6702	4895	4181	10805
ENSMUSG00000105112.1	Gm42778	9.91E-06	0.000253129	4	6	57.75	2.833333333	54.91666667	3.691297489	4.296127351	Higher in Normal	12	3	171	45	1	2	2	1	8	3
ENSMUSG00000029377.5	Ereg	9.93E-06	0.000253496	4	6	76.75	2666.333333	2589.583333	3.939213057	4.789365139	Higher in TRIM24	57	17	119	114	11	91	972	3730	1011	10183
ENSMUSG00000015966.16	Il17rb	1.00E-05	0.000255742	4	6	137.75	25	112.75	2.490351402	2.64421057	Higher in Normal	147	140	151	113	32	6	4	13	60	35
ENSMUSG00000024353.4	Mzb1	1.04E-05	0.000264161	4	6	471.5	60.16666667	411.3333333	3.151103002	3.488772073	Higher in Normal	129	1120	269	368	46	8	96	11	19	181

ENSMUSG00000049241.5	Hcar1	1.04E-05	0.000264161	4	6	1350	60.83333333	1289.166667	3.992588122	4.798892922	Higher in Normal	2746	2445	105	104	73	14	22	3	226	27
ENSMUSG00000050671.12	Ism2	1.04E-05	0.000264161	4	6	42.75	7.166666667	35.58333333	2.450534596	2.598618507	Higher in Normal	43	13	55	60	11	3	5	7	2	15
ENSMUSG00000106619.1	Gm9353	1.07E-05	0.000270083	4	6	18	0.833333333	17.16666667	3.900188781	4.715478867	Higher in Normal	17	41	10	4	0	1	1	1	0	2
ENSMUSG00000050251.5	Olfr809	1.08E-05	0.000272425	4	6	2.75	101	98.25	4.156080802	5.303481076	Higher in TRIM24	6	4	1	0	1	51	109	323	43	79
ENSMUSG00000050587.14	Lrrc4c	1.09E-05	0.000274	4	6	268	64	204	2.376175913	2.509074277	Higher in Normal	338	202	246	286	13	11	68	14	84	194
ENSMUSG00000022416.15	Cacna1i	1.11E-05	0.000278479	4	6	54.75	9.5	45.25	2.728120841	2.936825349	Higher in Normal	96	53	54	16	2	5	7	2	12	29
ENSMUSG00000026090.16	2010300C02Rik	1.12E-05	0.000281661	4	6	1803	197.5	1605.5	2.907380651	3.16664648	Higher in Normal	702	580	2872	3058	169	57	125	74	684	76
ENSMUSG00000102778.1	Gm38165	1.13E-05	0.000283358	4	6	14.5	0.666666667	13.83333333	3.979169821	4.792677566	Higher in Normal	27	13	7	11	0	0	1	0	0	3
ENSMUSG00000030278.11	Cidec	1.14E-05	0.000285103	4	6	17737.25	597.5	17139.75	4.202307995	5.214503938	Higher in Normal	28867	41174	414	494	947	132	243	35	2010	218
ENSMUSG00000027408.7	Cpxm1	1.16E-05	0.00028897	4	6	1045.25	10330.16667	9284.916667	2.94819516	3.231587937	Higher in TRIM24	803	1177	904	1297	584	4631	30006	6267	8318	12175
ENSMUSG00000045441.5	Gprin3	1.16E-05	0.000289697	4	6	410	91.66666667	318.3333333	2.355296029	2.485583814	Higher in Normal	393	173	763	311	76	8	47	40	111	268
ENSMUSG00000094690.1	1600014C23Rik	1.18E-05	0.000293554	4	6	0	16.16666667	16.16666667	4.587816193	6.146380627	Higher in TRIM24	0	0	0	0	3	4	2	18	25	45
ENSMUSG00000039720.7	Got1l1	1.19E-05	0.000296842	4	6	38.5	4.166666667	34.33333333	3.158614798	3.506572603	Higher in Normal	72	36	26	20	4	1	1	0	12	7
ENSMUSG00000031283.16	Chrdl1	1.20E-05	0.000297518	4	6	1785.25	118.6666667	1666.583333	3.553457328	4.079921356	Higher in Normal	2255	4342	277	267	20	22	200	227	136	107
ENSMUSG00000063661.5	Krt73	1.21E-05	0.000300407	4	6	64	3	61	3.973050874	4.781869629	Higher in Normal	7	5	93	151	0	0	1	1	4	12
ENSMUSG00000032281.11	Acsbg1	1.21E-05	0.000300407	4	6	300.25	3136.5	2836.25	3.281440466	3.700789173	Higher in TRIM24	201	212	364	424	252	8719	4670	878	2092	2208
ENSMUSG00000026414.13	Tnnt2	1.21E-05	0.000300856	4	6	134.75	1348.166667	1213.416667	3.287660718	3.71037479	Higher in TRIM24	186	130	110	113	694	4640	1032	770	582	371
ENSMUSG00000084960.1	B430010I23Rik	1.22E-05	0.000301078	4	6	114.5	15.83333333	98.66666667	2.946522347	3.220669211	Higher in Normal	152	174	88	44	19	7	1	3	29	36
ENSMUSG00000036598.4	Ccdc113	1.22E-05	0.000301242	4	6	40.75	4	36.75	3.325049071	3.736224668	Higher in Normal	39	18	32	74	1	0	0	1	9	13
ENSMUSG00000024846.5	Cst6	1.23E-05	0.000304046	4	6	60.25	1325.166667	1264.916667	3.615635157	4.227839737	Higher in TRIM24	132	49	29	31	257	567	215	285	6305	322
ENSMUSG00000044678.11	Ly6k	1.24E-05	0.000306576	4	6	64.25	12	52.25	2.435951546	2.582783963	Higher in Normal	30	71	46	110	18	2	4	7	19	22
ENSMUSG00000046844.6	Vat1l	1.25E-05	0.000306897	4	6	211	23.16666667	187.8333333	2.998729762	3.290780915	Higher in Normal	318	207	155	164	27	12	9	4	80	7
ENSMUSG00000030000.10	Add2	1.25E-05	0.000306917	4	6	44	893.1666667	849.1666667	3.744678005	4.453762868	Higher in TRIM24	125	20	24	7	36	2502	472	297	631	1421
ENSMUSG00000097754.1	Ptgs2os2	1.28E-05	0.000313288	4	6	8	171.3333333	163.3333333	3.365667973	3.832103221	Higher in TRIM24	11	2	3	16	7	40	47	52	160	722
ENSMUSG00000031132.1	Cd40lg	1.32E-05	0.000322086	4	6	23	2.833333333	20.16666667	2.887647817	3.146825217	Higher in Normal	12	9	23	48	2	0	3	1	5	6
ENSMUSG00000082226.3	Gm715	1.33E-05	0.000324306	4	6	16.25	2.5	13.75	2.570084663	2.750812269	Higher in Normal	17	9	22	17	3	1	4	1	3	3
ENSMUSG00000042429.8	Adora1	1.33E-05	0.000324832	4	6	1234.5	106.1666667	1128.333333	3.281535691	3.682689311	Higher in Normal	1955	1634	660	689	257	11	16	38	247	68
ENSMUSG00000071104.10	Ccdc110	1.36E-05	0.000330187	4	6	45.75	5.833333333	39.91666667	2.986409954	3.27462673	Higher in Normal	44	13	71	55	2	3	1	0	11	18
ENSMUSG00000097440.1	Gm6277	1.37E-05	0.000333221	4	6	55	9.333333333	45.66666667	2.593093841	2.775098248	Higher in Normal	106	50	28	36	6	2	13	3	20	12
ENSMUSG00000097814.5	Panct2	1.39E-05	0.000337366	4	6	55.5	8.833333333	46.66666667	2.67182429	2.873389346	Higher in Normal	71	61	54	36	11	3	0	5	18	16
ENSMUSG00000045349.15	Sh2d5	1.41E-05	0.000341721	4	6	183.5	2142.666667	1959.166667	3.242972432	3.652599057	Higher in TRIM24	144	11	309	270	1116	2913	395	444	5893	2095
ENSMUSG00000100371.1		1.42E-05	0.000343382	4	6	54.25	1.5	52.75	4.263372439	5.441077489	Higher in Normal	116	95	4	2	2	1	0	0	5	1
ENSMUSG00000021414.7	Fam217a	1.44E-05	0.000348035	4	6	77.5	12.33333333	65.16666667	2.854108274	3.104362605	Higher in Normal	129	112	33	36	4	2	15	2	21	30
ENSMUSG00000063193.8	Cd300lb	1.47E-05	0.000354253	4	6	107	747.1666667	640.1666667	2.462604992	2.621067764	Higher in TRIM24	168	160	55	45	233	617	1350	474	480	1329
ENSMUSG00000094420.2	Igkv10-96	1.48E-05	0.000355134	4	6	294.25	37	257.25	2.923291516	3.196259366	Higher in Normal	77	375	294	431	75	3	32	5	34	73
ENSMUSG00000022044.14	Stmn4	1.48E-05	0.000355237	4	6	18	478.1666667	460.1666667	3.961061211	4.898337543	Higher in TRIM24	8	27	13	24	5	170	1515	1012	88	79
ENSMUSG00000024675.19	Ms4a4c	1.49E-05	0.000356546	4	6	88.25	647.3333333	559.0833333	2.616867819	2.811319724	Higher in TRIM24	69	185	44	55	789	355	229	1117	610	784
ENSMUSG00000026676.7	Ccdc3	1.50E-05	0.000358964	4	6	199.75	19.66666667	180.0833333	3.193891116	3.564773122	Higher in Normal	423	228	92	56	11	3	37	4	52	11
ENSMUSG00000001604.14	Tcea3	1.54E-05	0.000367407	4	6	289.75	57.33333333	232.4166667	2.350562997	2.484496058	Higher in Normal	176	355	269	359	14	11	79	51	121	68
ENSMUSG00000024041.8	Cryaa	1.56E-05	0.000370413	4	6	2.25	115.6666667	113.4166667	4.208272938	5.518742363	Higher in TRIM24	0	2	3	4	2	5	24	273	15	375
ENSMUSG00000074264.12	Amy1	1.56E-05	0.000370413	4	6	4665.75	201	4464.75	4.037370893	4.935613593	Higher in Normal	9922	8338	210	193	184	24	71	12	784	131
ENSMUSG00000020963.15	Tshr	1.57E-05	0.000373098	4	6	1796.25	54.83333333	1741.416667	4.287832192	5.462237972	Higher in Normal	4119	2999	54	13	47	4	13	3	219	43
ENSMUSG00000034570.12	Inpp5j	1.59E-05	0.000377933	4	6	215.75	37.83333333	177.9166667	2.470948444	2.628583006	Higher in Normal	114	77	309	363	19	4	51	15	80	58
ENSMUSG00000016498.9	Pdcd1lg2	1.63E-05	0.00038449	4	6	28.75	178.1666667	149.4166667	2.430017956	2.583531531	Higher in TRIM24	34	20	24	37	38	157	400	58	150	266
ENSMUSG00000024535.15	Snx24	1.63E-05	0.00038449	4	6	215.5	1376.166667	1160.666667	2.41267852	2.562407913	Higher in TRIM24	215	204	199	244	201	1281	2368	881	505	3021
ENSMUSG00000027762.6	Sucnr1	1.63E-05	0.00038449	4	6	474.75	8.166666667	466.5833333	4.610279248	6.355173653	Higher in Normal	583	1309	3	4	10	0	2	0	29	8
ENSMUSG00000042672.15	Dcst1	1.64E-05	0.000386629	4	6	185.25	29.5	155.75	2.838859877	3.089555	Higher in Normal	241	394	63	43	7	13	28	14	49	66
ENSMUSG00000032181.7	Scg3	1.66E-05	0.000390764	4	6	269.25	29.16666667	240.0833333	3.263365776	3.667596259	Higher in Normal	407	373	125	172	15	4	21	0	77	58
ENSMUSG00000078452.10	Raet1d	1.67E-05	0.000391122	4	6	8.25	63.16666667	54.91666667	2.723105891	2.949556793	Higher in TRIM24	12	3	11	7	15	35	79	107	24	119
ENSMUSG00000040552.8	C3ar1	1.71E-05	0.00039969	4	6	427.5	3706	3278.5	2.559785236	2.743094272	Higher in TRIM24	481	930	145	154	3602	1807	3522	2285	1507	9513
ENSMUSG00000086448.1	9330162012Rik	1.73E-05	0.000404878	4	6	110	15.5	94.5	2.814838667	3.060071723	Higher in Normal	190	76	102	72	20	6	1	3	36	27
ENSMUSG00000041660.8	Bbox1	1.74E-05	0.000405147	4	6	301	20.66666667	280.3333333	3.547272762	4.097163634	Higher in Normal	66	208	425	505	15	3	3	0	74	29
ENSMUSG00000035407.8	Kank4	1.83E-05	0.0004248	4	6	2716.5	192.8333333	2523.666667	3.299958918	3.726228403	Higher in Normal	1289	742	4396	4439	419	29	138	20	504	47
ENSMUSG00000021033.11	Gstz1	1.85E-05	0.000429783	4	6	10700.25	1487.333333	9212.916667	2.942881065	3.229838328	Higher in Normal	22418	17174	1373	1836	1523	554	818	764	1982	3283
ENSMUSG00000102923.1	Gm31728	1.85E-05	0.000430365	4	6	16.25	1.166666667	15.08333333	3.412905024	3.933186411	Higher in Normal	21	25	11	8	0	2	2	0	2	1
ENSMUSG00000059146.12	Ntrk3	1.86E-05	0.000430365	4	6	2387.25	225.6666667	2161.583333	3.110495369	3.457619521	Higher in Normal	2395	723	3546	2885	75	32	20	276	727	224
ENSMUSG00000036356.15	Csgalnact1	1.87E-05	0.000432593	4	6	404.25	2125.5	1721.25	2.361684045	2.503427542	Higher in TRIM24	382	285	484	466	463	3106	3034	2011	867	3272
ENSMUSG00000049555.10	Tmie	1.87E-05	0.000432665	4	6	239.5	36.83333333	202.6666667	2.827907803	3.078495726	Higher in Normal	360	190	218	190	10	6	19	4	115	67
ENSMUSG00000086584.8	Gm12002	1.88E-05	0.000434166	4	6	218.25	40.5	177.75	2.650986596	2.853611056	Higher in Normal	498	218	71	86	8	16	34	20	55	110
ENSMUSG00000027460.7	Angpt4	1.90E-05	0.00043834	4	6	22.75	204.5	181.75	2.80762165	3.062648476	Higher in TRIM24	37	13	21	20	10	104	399	191	148	375
ENSMUSG00000096966.2	Gm18336	1.93E-05	0.000444202	4	6	24	1.5	22.5	3.678624385	4.337687155	Higher in Normal	59	11	16	10	0	1	0	0	4	4
ENSMUSG00000004328.15	Hif3a	1.94E-05	0.000444202	4	6	106.75	17.83333333	88.91666667	2.788644784	3.028547161	Higher in Normal	196	91	88	52	22	3	4	2	21	55
ENSMUSG00000079112.1	Fam90a1a	1.95E-05	0.000445816	4	6	60.25	4	56.25	3.514767774	4.068921202	Higher in Normal	35	174	26	6	6	1	3	6	6	2
ENSMUSG00000073902.5	Gm1966	1.97E-05	0.000449418	4	6	99.5	494.3333333	394.8333333	2.345733304	2.485259035	Higher in TRIM24	63	58	199	78	542	862	410	151	387	614
ENSMUSG00000090081.1	Gm16587	1.98E-05	0.000450709	4	6	71.75	2.5	69.25	4.166031707	5.243625221	Higher in Normal	14	5	159	109	0	0	0	0	6	9
ENSMUSG00000073420.10	Btnl5-ps	1.99E-05	0.000451239	4	6	40.75	1.833333333	38.91666667	4.107668973	5.110560293	Higher in Normal	66	3	61	33	0	0	0	0	1	10
ENSMUSG00000024538.7	Ppic	2.00E-05	0.000453575	4	6	1225.5	9287.666667	8062.166667	2.749322801	2.987462068	Higher in TRIM24	1273	1270	947	1412	984	5422	17592	16945	4214	10569
ENSMUSG00000025529.14	Zfp711	2.00E-05	0.000453575	4	6	191.5	42.16666667	149.3333333	2.353747912	2.491679549	Higher in Normal	108	69	313	276	15	8	21	13	79	117
ENSMUSG00000054619.6	Mettl7a1	2.02E-05	0.000455904	4	6	3436.75	513	2923.75	2.734964797	2.961708551	Higher in Normal	6301	5644	780	1022	435	185	733	273	985	467
ENSMUSG00000047143.3	Dmrta2	2.06E-05	0.000464638	4	6	95	747.6666667	652.6666667	2.708346944	2.935429734	Higher in TRIM24	161	27	92	100	879	573	44	679	821	1490
ENSMUSG00000087166.9	L1td1	2.10E-05	0.000472049	4	6	25	2	23	3.45271277	3.970094226	Higher in Normal	49	8	35	8	0	1	1	0	4	6
ENSMUSG00000017943.15	Gdap1l1	2.10E-05	0.000472244	4	6	64.75	8.166666667	56.58333333	3.024523445	3.343927246	Higher in Normal	123	21	47	68	1	0	3	8	12	25
ENSMUSG00000022577.16	Ly6h	2.11E-05	0.000472814	4	6	94	8.166666667	85.83333333	3.587359765	4.177831503	Higher in Normal	134	197	25	20	2	4	0	1	14	28
ENSMUSG00000028871.7	Rspo1	2.14E-05	0.000479565	4	6	3263.25	121.1666667	3142.083333	3.842671965	4.614827628	Higher in Normal	2282	507	4200	6064	8	8	586	31	73	21
ENSMUSG00000043857.16	Mgat5b	2.15E-05	0.000479987	4	6	45.25	2.666666667	42.58333333	3.693919738	4.361908415	Higher in Normal	95	4	57	25	0	0	1	1	9	5
ENSMUSG00000097120.1	Gm26887	2.18E-05	0.000486739	4	6	90	7.333333333	82.66666667	3.525349467	4.07714473	Higher in Normal	32	8	145	175	1	3	2	0	5	33
ENSMUSG00000040181.14	Fmo1	2.22E-05	0.00049628	4	6	3285.5	341.8333333	2943.666667	3.190096089	3.578256374	Higher in Normal	5857	3818	1577	1890	183	51	223	26	1335	233
ENSMUSG00000028186.14	Uox	2.23E-05	0.000496962	4	6	28.5	769	740.5	3.69195493	4.426105725	Higher in TRIM24	101	6	4	3	75	179	1077	889	178	2216
ENSMUSG00000064202.15	4430402I18Rik	2.28E-05	0.000507608	4	6	189.25	24.16666667	165.0833333	2.97224545	3.277505138	Higher in Normal	376	281	57	43	21	10	11	6	68	29
ENSMUSG00000111598.1	Gm47095	2.33E-05	0.000516796	4	6	28.75	2	26.75	3.556226049	4.136356716	Higher in Normal	21	5	61	28	0	0	1	0	5	6
ENSMUSG00000065608.1	Mirlet7c-2	2.36E-05	0.000522923	4	6	20.75	1.166666667	19.58333333	3.812034937	4.607867027	Higher in Normal	12	55	9	7	2	0	0	0	2	3
ENSMUSG00000086154.1	Gm16196	2.40E-05	0.000530422	4	6	25.75	3	22.75	3.218324825	3.616468845	Higher in Normal	35	17	21	30	1	1	0	0	2	14

ENSMUSG00000023885.8	Thbs2	2.41E-05	0.0005311	4	6	2280.25	23098.16667	20817.91667	2.845390284	3.12021201	Higher in TRIM24	1917	5176	1008	1020	7614	18711	58397	5288	17567	31012
ENSMUSG00000092618.2	Btnl6	2.41E-05	0.0005311	4	6	14.5	0.333333333	14.16666667	4.196811205	5.337807492	Higher in Normal	27	1	17	13	0	0	0	0	1	1
ENSMUSG00000100209.1	Gm28793	2.43E-05	0.000535599	4	6	10	0.166666667	9.833333333	4.187428799	5.302593699	Higher in Normal	18	5	12	5	0	0	0	0	0	1
ENSMUSG00000082491.1	Gm5909	2.43E-05	0.000535599	4	6	17.5	116.3333333	98.83333333	2.398280605	2.552809349	Higher in TRIM24	46	5	9	10	104	45	110	100	78	261
ENSMUSG00000034917.8	Tjp3	2.46E-05	0.000539997	4	6	4791.25	432.8333333	4358.416667	3.132291699	3.501236054	Higher in Normal	2539	1757	6722	8147	570	63	124	69	1590	181
ENSMUSG00000085382.1	Gm13861	2.48E-05	0.000544984	4	6	11.75	1.333333333	10.41666667	2.966026758	3.287462305	Higher in Normal	11	14	9	13	1	1	2	0	2	2
ENSMUSG00000022484.7	Hoxc10	2.49E-05	0.000546456	4	6	201.25	12.83333333	188.4166667	3.777697755	4.518554658	Higher in Normal	168	503	52	82	26	0	7	0	6	38
ENSMUSG00000043463.6	Rab9b	2.51E-05	0.000549287	4	6	27	4	23	2.931434787	3.220870103	Higher in Normal	35	21	21	31	0	0	2	2	4	16
ENSMUSG00000042988.10	Notum	2.53E-05	0.000553586	4	6	11	476	465	3.771335966	4.596459015	Higher in TRIM24	19	3	13	9	26	10	22	172	117	2509
ENSMUSG00000028111.4	Ctsk	2.55E-05	0.000557878	4	6	1017.75	12524.33333	11506.58333	3.031715721	3.37880258	Higher in TRIM24	797	1978	545	751	679	5359	10976	22399	4426	31307
ENSMUSG00000025509.15	Pnpla2	2.60E-05	0.000567245	4	6	25501.25	3815.333333	21685.91667	2.726518591	2.958081661	Higher in Normal	43147	39060	8530	11268	3059	1900	2724	1174	11642	2393
ENSMUSG00000087030.1	Gm16143	2.65E-05	0.000577758	4	6	28.75	2.333333333	26.41666667	3.490156644	4.046161963	Higher in Normal	64	21	14	16	4	0	0	0	2	8
ENSMUSG00000000126.11	Wnt9a	2.66E-05	0.00057962	4	6	120	788.5	668.5	2.388229676	2.541905082	Higher in TRIM24	81	184	112	103	94	784	1199	402	650	1602
ENSMUSG00000076695.7	Ighv1-18	2.66E-05	0.00057962	4	6	116.75	2.333333333	114.4166667	4.376106808	5.859391638	Higher in Normal	0	15	214	238	3	0	3	0	0	8
ENSMUSG00000047861.2	Foxi1	2.75E-05	0.000594815	4	6	1986.75	137.8333333	1848.916667	3.456792089	3.990688865	Higher in Normal	958	1459	2437	3093	101	16	56	4	611	39
ENSMUSG00000049556.5	Lingo1	2.80E-05	0.00060468	4	6	498.25	64.16666667	434.0833333	2.594290703	2.792719787	Higher in Normal	92	179	775	947	50	28	110	55	111	31
ENSMUSG00000031538.6	Plat	2.86E-05	0.000616669	4	6	1347.25	7173	5825.75	2.349220063	2.495860264	Higher in TRIM24	527	665	1974	2223	1837	4362	15712	7015	4183	9929
ENSMUSG00000013584.5	Aldh1a2	2.87E-05	0.000617541	4	6	65.75	871.8333333	806.0833333	3.146513307	3.551765681	Higher in TRIM24	67	156	14	26	174	578	2383	334	1285	477
ENSMUSG00000030806.6	Stx1b	2.87E-05	0.000618594	4	6	307	32.83333333	274.1666667	3.210195231	3.621530986	Higher in Normal	704	395	80	49	21	18	12	2	83	61
ENSMUSG00000059632.3	Krtap8-1	2.88E-05	0.000618833	4	6	50.25	0	50.25	4.868248895	7.907446356	Higher in Normal	2	0	82	117	0	0	0	0	0	0
ENSMUSG00000024972.16	Lgals12	2.90E-05	0.000624501	4	6	3279.25	142.3333333	3136.916667	3.919670739	4.800909508	Higher in Normal	6927	5626	270	294	318	12	30	5	415	74
ENSMUSG00000039168.15	Dap	3.00E-05	0.000643113	4	6	3446	27234	23788	2.327740136	2.470893029	Higher in TRIM24	2598	4088	2962	4136	8043	6001	18830	17591	6558	106381
ENSMUSG00000060594.6	Layn	3.05E-05	0.000652678	4	6	160.25	1009	848.75	2.338773544	2.484475992	Higher in TRIM24	102	100	228	211	106	511	1293	921	467	2756
ENSMUSG00000032718.4	Mansc1	3.06E-05	0.000653042	4	6	1915	307.8333333	1607.166667	2.413104949	2.571211997	Higher in Normal	732	553	2920	3455	380	130	304	94	716	223
ENSMUSG00000021822.2	Plau	3.07E-05	0.000653982	4	6	2370.25	22396	20025.75	3.126357325	3.525083102	Higher in TRIM24	1886	1967	2499	3129	2152	69563	16996	9510	7095	29060
ENSMUSG00000052920.14	Prkg1	3.08E-05	0.000656098	4	6	693.25	76.16666667	617.0833333	2.837387648	3.108233474	Higher in Normal	224	55	1610	884	46	74	100	12	114	111
ENSMUSG00000022490.6	Ppp1r1a	3.11E-05	0.000661542	4	6	983.75	70.83333333	912.9166667	3.457185483	4.001557597	Higher in Normal	1812	1828	127	168	10	9	209	67	71	59
ENSMUSG00000076541.3	Igkv4-79	3.12E-05	0.000663049	4	6	38.25	3.166666667	35.08333333	3.580656704	4.20439668	Higher in Normal	9	101	21	22	1	0	5	0	2	11
ENSMUSG00000092920.1	Mirt2	3.14E-05	0.000666309	4	6	2.5	44.66666667	42.16666667	3.291534198	3.795604847	Higher in TRIM24	6	3	0	1	29	4	28	14	120	73
ENSMUSG00000038738.15	Shank1	3.16E-05	0.000670451	4	6	237.25	48	189.25	2.409629744	2.567400966	Higher in Normal	389	108	290	162	11	10	23	31	122	91
ENSMUSG00000019124.10	Scrn1	3.17E-05	0.000671817	4	6	99	549.5	450.5	2.382261629	2.538046574	Higher in TRIM24	140	108	71	77	497	299	649	1056	152	644
ENSMUSG00000058354.6	Krt6a	3.18E-05	0.000672632	4	6	34.5	6090.666667	6056.166667	4.544820999	7.375960333	Higher in TRIM24	22	7	45	64	5303	15	65	7	31111	43
ENSMUSG00000049362.6	Olfr173	3.23E-05	0.000679721	4	6	16.5	2.5	14	2.684126848	2.913976273	Higher in Normal	13	16	21	16	0	1	1	4	2	7
ENSMUSG00000060675.13	Pla2g16	3.24E-05	0.0006818	4	6	12145.25	2335.833333	9809.416667	2.430695613	2.593739942	Higher in Normal	18279	23152	2905	4245	2021	1262	2408	1817	3494	3013
ENSMUSG00000083674.3	Zfp133-ps	3.28E-05	0.000689802	4	6	40.25	8.666666667	31.58333333	2.3647329	2.514791958	Higher in Normal	58	65	16	22	7	3	8	5	11	18
ENSMUSG00000032271.13	Nnmt	3.30E-05	0.000693059	4	6	2175.75	153.5	2022.25	3.544517568	4.147547352	Higher in Normal	2639	5883	93	88	47	65	276	96	279	158
ENSMUSG00000006204.6	5430419D17Rik	3.38E-05	0.000708735	4	6	209.25	26.83333333	182.4166667	2.991628037	3.319486872	Higher in Normal	184	210	253	190	3	3	42	1	59	53
ENSMUSG00000066682.11	Pilrb2	3.44E-05	0.00071722	4	6	46.25	302.5	256.25	2.45017712	2.623512382	Higher in TRIM24	46	93	19	27	174	356	545	181	171	388
ENSMUSG00000109998.1	Gm45437	3.44E-05	0.00071722	4	6	29	2.5	26.5	3.168037651	3.577330717	Higher in Normal	18	8	57	33	5	0	1	0	6	3
ENSMUSG00000030108.14	Slc6a13	3.44E-05	0.00071722	4	6	311.25	21	290.25	3.80743812	4.611279586	Higher in Normal	557	629	31	28	7	0	17	1	36	65
ENSMUSG00000043613.8	Mmp3	3.45E-05	0.000718674	4	6	1800	26365.33333	24565.33333	2.913369743	3.227114394	Higher in TRIM24	2025	2286	1179	1710	838	4300	20676	12217	12055	108106
ENSMUSG00000112327.1	Gm36827	3.48E-05	0.000723641	4	6	122.75	4	118.75	4.335685047	5.815756077	Higher in Normal	69	417	4	1	1	0	0	1	7	15
ENSMUSG00000073460.4	Pnldc1	3.48E-05	0.000723824	4	6	206	38.5	167.5	2.639944619	2.855764847	Higher in Normal	374	324	74	52	23	8	21	19	74	86
ENSMUSG00000040289.8	Hey1	3.51E-05	0.000728649	4	6	5516.5	726.1666667	4790.333333	2.676209989	2.902384772	Higher in Normal	722	2405	8868	10071	238	241	507	695	1989	687
ENSMUSG00000006369.14	Fbln1	3.53E-05	0.000731669	4	6	609.75	4342.333333	3732.583333	2.556580926	2.756487692	Higher in TRIM24	906	581	409	543	654	1924	12341	2584	4333	4218
ENSMUSG00000019278.5	Dpep1	3.59E-05	0.000742937	4	6	2071.5	415.5	1656	2.379946003	2.534164146	Higher in Normal	3070	2905	1032	1279	97	203	822	170	674	527
ENSMUSG00000042763.9	Maneal	3.64E-05	0.000751405	4	6	24.75	4.5	20.25	2.453057168	2.626112832	Higher in Normal	21	37	18	23	1	1	6	5	8	6
ENSMUSG00000067878.13	Map7d3	3.69E-05	0.000758246	4	6	118.75	24.66666667	94.08333333	2.515064241	2.70023071	Higher in Normal	204	177	56	38	10	12	15	6	44	61
ENSMUSG00000076598.3	Igkv3-7	3.74E-05	0.000766614	4	6	72	7	65	2.922080451	3.235321104	Higher in Normal	35	118	51	84	17	10	7	2	4	2
ENSMUSG00000076608.2	Igkj5	3.75E-05	0.000766873	4	6	18.25	0.833333333	17.41666667	3.879180945	4.800806153	Higher in Normal	2	29	23	19	0	0	2	0	0	3
ENSMUSG00000020423.6	Btg2	3.75E-05	0.000767059	4	6	19169.75	2340.5	16829.25	2.629562829	2.844744326	Higher in Normal	2209	2911	33037	38522	2987	1921	2448	1488	2382	2817
ENSMUSG00000033022.7	Cdo1	3.77E-05	0.000770544	4	6	17730.75	1201	16529.75	3.5678643	4.19899255	Higher in Normal	39385	24878	2738	3922	1291	104	438	121	4812	440
ENSMUSG00000048480.5	Cxcr1	3.77E-05	0.000770544	4	6	2.75	36.66666667	33.91666667	3.102614874	3.504844117	Higher in TRIM24	2	0	8	1	23	5	17	15	81	79
ENSMUSG00000041731.13	Pgm5	3.81E-05	0.000777671	4	6	1297.5	162.5	1135	2.998357777	3.334661482	Higher in Normal	351	488	2194	2157	20	14	266	21	170	484
ENSMUSG00000073145.6	Gm12000	3.85E-05	0.000781865	4	6	15.75	1	14.75	3.436035994	4.019012836	Higher in Normal	6	8	26	23	2	0	0	0	3	1
ENSMUSG00000018008.7	Cyth4	3.88E-05	0.000786645	4	6	905.5	5519.5	4614	2.358219549	2.512611811	Higher in TRIM24	883	1837	406	496	2228	5127	8623	6129	3909	7101
ENSMUSG00000029828.4	4921507P07Rik	3.97E-05	0.000804059	4	6	129.5	21	108.5	2.37276116	2.528225571	Higher in Normal	118	69	146	185	35	23	16	2	34	16
ENSMUSG00000025813.14	Homer2	4.02E-05	0.00081135	4	6	1218.5	123.6666667	1094.833333	2.818120333	3.092365097	Higher in Normal	655	469	2149	1601	137	9	74	303	99	120
ENSMUSG00000063011.6	Msln	4.03E-05	0.000812904	4	6	278.25	5471	5192.75	3.636705252	4.39782466	Higher in TRIM24	115	85	402	511	56	13556	1130	1071	1417	15596
ENSMUSG00000104616.1	Gm6069	4.06E-05	0.000817706	4	6	123	12.5	110.5	2.993432164	3.331884135	Higher in Normal	110	83	133	166	24	1	6	2	38	4
ENSMUSG00000017344.4	Vtn	4.06E-05	0.000819106	4	6	506.75	63.33333333	443.4166667	2.857819844	3.145876183	Higher in Normal	423	739	360	505	2	19	131	48	125	55
ENSMUSG00000087333.1	Gm13652	4.08E-05	0.000821442	4	6	28.25	3.5	24.75	2.875876295	3.171801252	Higher in Normal	32	16	39	26	0	1	7	0	8	5
ENSMUSG00000046169.9	Adamts6	4.11E-05	0.000827083	4	6	68.5	465	396.5	2.500630824	2.689952202	Higher in TRIM24	90	22	103	59	125	720	346	103	268	1228
ENSMUSG00000024743.14	Syt7	4.22E-05	0.000847217	4	6	896.5	191	705.5	2.560312622	2.760092912	Higher in Normal	615	471	1301	1199	63	14	60	112	83	814
ENSMUSG00000109293.1	Dcst2	4.24E-05	0.000850171	4	6	165.25	28.66666667	136.5833333	2.76676494	3.025839866	Higher in Normal	290	265	44	62	17	11	11	4	55	74
ENSMUSG00000036770.11	Stpg3	4.24E-05	0.000851138	4	6	44.5	3.5	41	3.57378181	4.218277445	Higher in Normal	114	7	46	11	2	0	1	0	4	14
ENSMUSG00000090843.2	Heatr4	4.32E-05	0.000865441	4	6	72	11.83333333	60.16666667	2.754227857	3.008697151	Higher in Normal	159	16	75	38	4	1	7	5	18	36
ENSMUSG00000022040.7	Ephx2	4.33E-05	0.000867124	4	6	3858.25	533.8333333	3324.416667	2.908887789	3.217435151	Higher in Normal	5904	8337	524	668	440	469	197	187	748	1162
ENSMUSG00000021732.14	Fgf10	4.34E-05	0.000867967	4	6	1184	77.16666667	1106.833333	3.658743686	4.371634431	Higher in Normal	1821	2792	59	64	6	14	139	24	186	94
ENSMUSG00000032942.14	Ucp3	4.34E-05	0.000867967	4	6	880.75	79.16666667	801.5833333	3.253929742	3.711876338	Higher in Normal	1604	908	470	541	80	12	52	2	294	35
ENSMUSG00000071984.10	Fndc1	4.37E-05	0.000873173	4	6	355	2763.166667	2408.166667	2.51546213	2.710241813	Higher in TRIM24	440	722	135	123	1048	1203	5647	1627	3506	3548
ENSMUSG00000025856.15	Pdgfa	4.38E-05	0.000874234	4	6	1630.5	9542.166667	7911.666667	2.551798132	2.756252585	Higher in TRIM24	1154	1619	1411	2338	3122	6602	13629	22963	3807	7130
ENSMUSG00000113334.1	D030007L05Rik	4.41E-05	0.000880055	4	6	3	30.83333333	27.83333333	2.951186793	3.302707776	Higher in TRIM24	5	4	2	1	4	21	67	29	29	35
ENSMUSG00000029121.15	Crmp1	4.44E-05	0.000884367	4	6	27.5	327.3333333	299.8333333	3.25064625	3.74145231	Higher in TRIM24	31	39	19	21	33	146	536	1000	63	186
ENSMUSG00000020155.2	Kcnmb1	4.52E-05	0.000897106	4	6	797.25	69	728.25	2.961123639	3.291587057	Higher in Normal	89	103	1390	1607	111	71	57	17	114	44
ENSMUSG00000030268.17	Bcat1	4.58E-05	0.000907473	4	6	225.75	2050	1824.25	2.588796884	2.804934404	Higher in TRIM24	293	239	192	179	163	512	2116	2142	704	6663
ENSMUSG00000029499.11	Pxmp2	4.61E-05	0.000912008	4	6	459.25	67.33333333	391.9166667	2.764523186	3.026373079	Higher in Normal	1003	670	69	95	35	40	99	32	98	100
ENSMUSG00000044576.6	Garem2	4.61E-05	0.000912588	4	6	9.25	58	48.75	2.469882446	2.656439338	Higher in TRIM24	15	10	3	9	19	33	83	96	53	64
ENSMUSG00000032454.9	Rbp2	4.66E-05	0.000920509	4	6	15.25	458.6666667	443.4166667	3.732399541	4.616348187	Higher in TRIM24	38	9	12	2	48	1	492	761	158	1292
ENSMUSG00000035964.8	Tmem59l	4.66E-05	0.000920509	4	6	7.25	97.16666667	89.91666667	3.315796259	3.855704239	Higher in TRIM24	5	12	8	4	390	36	47	55	16	39
ENSMUSG00000035020.11	Epgn	4.69E-05	0.000925048	4	6	27.75	929.8333333	902.0833333	3.79647865	4.751517626	Higher in TRIM24	19	2	47	43	44	33	23	198	3541	1740
ENSMUSG00000030781.13	Slc5a2	4.72E-05	0.000928556	4	6	18.5	1.5	17	3.316217452	3.827196286	Higher in Normal	19	5	36	14	0	2	0	0	2	5

ENSMUSG00000047104.5	Pbp2	4.72E-05	0.000929246	4	6	1.5	27.16666667	25.66666667	3.496058893	4.189127695	Higher in TRIM24	5	0	0	1	6	15	68	20	39	15
ENSMUSG00000075224.8	Lrrc55	4.74E-05	0.000931354	4	6	8.75	85.33333333	76.58333333	3.043252685	3.431630548	Higher in TRIM24	18	4	8	5	29	164	100	17	174	28
ENSMUSG00000087054.1	Gm12405	4.82E-05	0.000944692	4	6	8	0.166666667	7.833333333	3.935431164	4.991234252	Higher in Normal	6	10	8	8	0	0	1	0	0	0
ENSMUSG00000029925.13	Tbxas1	4.84E-05	0.000947618	4	6	192.5	1203.833333	1011.333333	2.355169436	2.513567839	Higher in TRIM24	317	308	74	71	781	1401	1601	737	651	2052
ENSMUSG00000055026.13	Gabrg3	4.87E-05	0.000952191	4	6	235.5	19.5	216	3.593483043	4.267447211	Higher in Normal	165	203	318	256	2	0	3	0	38	74
ENSMUSG00000086332.1	4930480G23Rik	4.89E-05	0.000954745	4	6	20	0.5	19.5	4.175547573	5.520490906	Higher in Normal	26	42	7	5	0	0	0	0	3	0
ENSMUSG00000048186.14	Bend7	4.92E-05	0.00095907	4	6	541	94	447	2.324229207	2.472920959	Higher in Normal	333	185	833	813	22	33	101	107	222	79
ENSMUSG00000007107.6	Atp1a4	5.01E-05	0.000973876	4	6	144.25	17.5	126.75	3.039731631	3.407374702	Higher in Normal	275	250	34	18	21	2	19	5	29	29
ENSMUSG00000022055.7	Nefl	5.03E-05	0.000974559	4	6	4	90.16666667	86.16666667	3.479264764	4.153770489	Higher in TRIM24	4	10	2	0	7	56	123	100	9	246
ENSMUSG00000076609.2	Igkc	5.03E-05	0.000974559	4	6	27146	4617.833333	22528.16667	2.98230498	3.325745117	Higher in Normal	8056	69048	12143	19337	2367	806	3585	559	1227	19163
ENSMUSG00000047507.12	Baiap3	5.03E-05	0.000974559	4	6	108.75	22.83333333	85.91666667	2.446192412	2.622104777	Higher in Normal	171	127	75	62	4	3	13	20	43	54
ENSMUSG00000006345.10	Ggt1	5.03E-05	0.000974607	4	6	1067.5	105	962.5	2.888236521	3.19587743	Higher in Normal	394	315	1600	1961	326	12	68	39	101	84
ENSMUSG00000097730.3	Gm26588	5.11E-05	0.000987493	4	6	74	11	63	2.997119289	3.345720678	Higher in Normal	128	112	39	17	5	1	2	3	20	35
ENSMUSG00000020893.17	Per1	5.13E-05	0.000990496	4	6	8499.5	1310.166667	7189.333333	2.528896003	2.725975629	Higher in Normal	1141	1275	16355	15227	851	1084	1005	569	1051	3301
ENSMUSG00000031853.5	Map3k21	5.14E-05	0.000991241	4	6	1054	83	971	3.281177256	3.765392036	Higher in Normal	650	380	1694	1492	67	9	28	4	361	29
ENSMUSG00000026837.15	Col5a1	5.17E-05	0.000996453	4	6	2792.25	24071.83333	21279.58333	2.729212416	2.99305075	Higher in TRIM24	3268	4017	1973	1911	1601	8007	52227	32363	16941	33292
ENSMUSG00000068747.14	Sort1	5.21E-05	0.00100314	4	6	6145.25	1024.666667	5120.583333	2.383411855	2.545964705	Higher in Normal	8026	6138	5565	4852	2110	404	874	633	1846	281
ENSMUSG00000031443.7	F7	5.26E-05	0.001011119	4	6	17.25	303.3333333	286.0833333	3.434845916	4.066061098	Higher in TRIM24	11	45	3	10	145	261	1182	63	71	98
ENSMUSG00000018211.13	Wfdc15b	5.28E-05	0.001014108	4	6	16	0.5	15.5	3.962882219	4.993652412	Higher in Normal	13	1	22	28	0	0	0	0	2	1
ENSMUSG00000025329.3	Padi1	5.29E-05	0.001014512	4	6	23	347.5	324.5	3.530314794	4.236932493	Higher in TRIM24	24	27	23	18	56	1285	49	33	551	111
ENSMUSG00000097252.1	Gm6634	5.29E-05	0.001014512	4	6	26.75	392.1666667	365.4166667	3.302835159	3.840531427	Higher in TRIM24	65	27	10	5	20	517	267	676	86	787
ENSMUSG00000022226.5	Mcpt2	5.33E-05	0.001019023	4	6	1.5	26	24.5	3.251749525	3.776457155	Higher in TRIM24	2	1	3	0	24	2	30	12	8	80
ENSMUSG00000091212.1	Krtap11-1	5.33E-05	0.001019023	4	6	29.5	0.166666667	29.33333333	4.562021154	6.677200742	Higher in Normal	3	0	51	64	0	0	0	0	0	1
ENSMUSG00000000216.8	Scnn1g	5.38E-05	0.001026411	4	6	824.25	48.5	775.75	3.493486419	4.110525016	Higher in Normal	120	122	1499	1556	32	11	11	3	218	16
ENSMUSG00000033491.13	Prss35	5.46E-05	0.001039152	4	6	31.75	690	658.25	3.657148531	4.48558565	Higher in TRIM24	59	9	32	27	16	50	3202	394	176	302
ENSMUSG00000048138.9	Dmrt2	5.48E-05	0.001041172	4	6	1078.75	46.5	1032.25	3.874125587	4.817152862	Higher in Normal	2726	1561	12	16	18	9	92	9	131	20
ENSMUSG00000054409.4	Tmem74	5.48E-05	0.001041292	4	6	1.25	77.66666667	76.41666667	4.04045561	5.462197444	Higher in TRIM24	1	0	1	3	0	7	121	16	8	314
ENSMUSG00000028359.4	Orm3	5.50E-05	0.001043936	4	6	284.5	12.66666667	271.8333333	3.861418477	4.795159354	Higher in Normal	932	188	12	6	18	5	5	0	29	19
ENSMUSG00000028031.6	Dkk2	5.51E-05	0.001045395	4	6	165.5	3330	3164.5	3.408283181	4.02082553	Higher in TRIM24	114	219	151	178	149	393	441	7032	369	11596
ENSMUSG00000032334.10	Loxl1	5.54E-05	0.001049378	4	6	1352.75	11719.66667	10366.91667	2.774535492	3.056903816	Higher in TRIM24	1513	1868	869	1161	718	5182	22298	19719	5304	17097
ENSMUSG00000074771.10	Ankef1	5.58E-05	0.001055262	4	6	2248.25	81.5	2166.75	4.045229143	5.196632717	Higher in Normal	5255	3690	29	19	48	10	23	5	363	40
ENSMUSG00000091568.8	Gm8206	5.60E-05	0.001058472	4	6	16.5	1.5	15	3.094644554	3.511171184	Higher in Normal	8	8	25	25	0	1	1	3	0	4
ENSMUSG00000029603.15	Dtx1	5.63E-05	0.001062662	4	6	476	66.5	409.5	2.662755208	2.89965819	Higher in Normal	213	257	671	763	64	6	89	12	180	48
ENSMUSG00000026834.13	Acvr1c	5.68E-05	0.001069735	4	6	1196	68	1128	3.768215551	4.611181288	Higher in Normal	2034	2660	52	38	58	14	22	2	230	82
ENSMUSG00000031780.2	Ccl17	5.84E-05	0.001096135	4	6	22.25	342.5	320.25	3.429397714	4.065483755	Higher in TRIM24	8	19	26	36	34	57	1511	290	52	111
ENSMUSG00000093955.2	Ighv1-34	5.87E-05	0.001099743	4	6	15.25	0.5	14.75	4.108390157	5.326971694	Higher in Normal	3	42	12	4	0	0	0	0	1	2
ENSMUSG00000110250.1	Gm9077	5.87E-05	0.001100626	4	6	32.5	2.666666667	29.83333333	3.492171931	4.112625091	Higher in Normal	67	7	39	17	0	0	1	0	5	10
ENSMUSG00000095519.2	Ighv1-66	5.90E-05	0.001103295	4	6	36.5	3.666666667	32.83333333	2.873487339	3.188960536	Higher in Normal	6	8	65	67	5	2	6	2	3	4
ENSMUSG00000094088.2	Ighv1-64	5.93E-05	0.00110717	4	6	38.75	3.5	35.25	3.391834788	3.948422925	Higher in Normal	2	40	58	55	2	0	2	0	3	14
ENSMUSG00000066975.12	Cryba4	5.99E-05	0.00111604	4	6	14.5	143.3333333	128.8333333	2.661360016	2.909149256	Higher in TRIM24	9	17	14	18	23	31	23	83	341	359
ENSMUSG00000027792.11	Bche	5.99E-05	0.001116187	4	6	1680	94.83333333	1585.166667	3.6135745	4.330935189	Higher in Normal	3715	2001	539	465	228	6	30	2	272	31
ENSMUSG00000041347.5	Bdkrb1	6.04E-05	0.00112219	4	6	33.25	455.5	422.25	2.967959936	3.332563505	Higher in TRIM24	35	10	43	45	11	136	394	252	100	1840
ENSMUSG00000035112.17	Wnk4	6.04E-05	0.00112219	4	6	2431.75	157	2274.75	3.311725688	3.825846527	Higher in Normal	292	169	4503	4763	477	19	40	74	89	243
ENSMUSG00000001943.8	Vsig2	6.07E-05	0.001126242	4	6	287.25	45	242.25	2.622417858	2.850336906	Higher in Normal	403	419	164	163	6	44	46	12	119	43
ENSMUSG00000044749.13	Abca6	6.11E-05	0.001132148	4	6	134	22	112	2.804169939	3.08836992	Higher in Normal	177	98	163	98	2	4	14	1	48	63
ENSMUSG00000078451.5	Ppil6	6.13E-05	0.001134404	4	6	79.5	11.83333333	67.66666667	2.599960955	2.821504316	Higher in Normal	43	36	120	119	9	7	2	2	37	14
ENSMUSG00000032500.10	Dclk3	6.13E-05	0.00113472	4	6	85	14.16666667	70.83333333	2.503062675	2.69938105	Higher in Normal	87	115	64	74	9	14	26	0	19	17
ENSMUSG00000045875.12	Adra1a	6.17E-05	0.001139286	4	6	741	60.66666667	680.3333333	3.380342463	3.936830598	Higher in Normal	753	410	1140	661	31	5	4	1	233	90
ENSMUSG00000029718.14	Pcolce	6.21E-05	0.001143239	4	6	1785.75	14586.83333	12801.08333	2.547758484	2.761184644	Higher in TRIM24	2047	2352	1115	1629	2651	2524	11417	24725	5841	40363
ENSMUSG00000060579.12	Fhit	6.30E-05	0.001159746	4	6	182.75	23.66666667	159.0833333	2.646057839	2.883114088	Higher in Normal	196	228	129	178	61	29	7	4	22	19
ENSMUSG00000058163.14	Gm5431	6.42E-05	0.001175928	4	6	32	223.1666667	191.1666667	2.484028685	2.681704118	Higher in TRIM24	43	57	18	10	432	130	141	84	370	182
ENSMUSG00000038523.10	1700003F12Rik	6.43E-05	0.001175928	4	6	15	165	150	2.919852511	3.26881724	Higher in TRIM24	23	19	8	10	20	24	348	224	50	324
ENSMUSG00000041620.8	Mmp1b	6.49E-05	0.001184435	4	6	0.25	61.66666667	61.41666667	4.335652275	6.95839627	Higher in TRIM24	1	0	0	0	0	15	1	8	85	261
ENSMUSG00000027525.17	Phactr3	6.53E-05	0.001190211	4	6	57.75	9	48.75	2.700241494	2.952828057	Higher in Normal	95	12	63	61	4	2	1	4	22	21
ENSMUSG00000005268.20	Prlr	6.56E-05	0.001194507	4	6	13911.5	1079.166667	12832.33333	3.178910829	3.627117467	Higher in Normal	5237	12522	21117	16770	3360	33	654	237	1804	387
ENSMUSG00000030087.11	Klf15	6.61E-05	0.001202181	4	6	742.5	88.16666667	654.3333333	2.937677396	3.277068295	Higher in Normal	1689	1090	88	103	111	32	91	46	174	75
ENSMUSG00000024124.9	Prss30	6.62E-05	0.001204894	4	6	7.25	47.33333333	40.08333333	2.761186775	3.048047262	Higher in TRIM24	7	3	12	7	39	121	38	26	33	27
ENSMUSG00000046275.2	Tusc5	6.64E-05	0.001206573	4	6	3631	258.5	3372.5	3.516772443	4.172632935	Higher in Normal	6583	7556	157	228	255	66	127	37	899	167
ENSMUSG00000093979.7	Gm2237	6.70E-05	0.001214963	4	6	111.25	14.16666667	97.08333333	2.597476468	2.822432669	Higher in Normal	31	66	153	195	4	5	14	27	27	8
ENSMUSG00000034226.7	Rhov	6.71E-05	0.001216875	4	6	3623.25	287.1666667	3336.083333	3.315650894	3.840565475	Higher in Normal	256	961	5554	7722	41	23	90	81	1101	387
ENSMUSG00000058921.3	Slc10a5	6.75E-05	0.001222593	4	6	30.25	4.833333333	25.41666667	2.774778616	3.051294029	Higher in Normal	35	20	46	20	1	2	0	1	8	17
ENSMUSG00000022766.12	Serpind1	6.82E-05	0.001233034	4	6	15	191.8333333	176.8333333	3.385038835	4.006463857	Higher in TRIM24	20	12	20	8	16	149	54	772	46	114
ENSMUSG00000001555.10	Fkbp10	6.82E-05	0.001233034	4	6	1200	7866.833333	6666.833333	2.434455288	2.619750796	Higher in TRIM24	480	815	1504	2001	1821	1623	11645	10827	2607	18678
ENSMUSG00000096670.1	Ighv2-6	7.00E-05	0.001259945	4	6	41.75	5.666666667	36.08333333	2.832287622	3.131528954	Higher in Normal	40	8	52	67	4	0	7	0	8	15
ENSMUSG00000043670.4	Diras1	7.01E-05	0.001260243	4	6	52.75	4	48.75	3.183493377	3.652537881	Higher in Normal	134	4	49	24	1	7	1	5	4	6
ENSMUSG00000032511.17	Scn5a	7.03E-05	0.001262251	4	6	229.25	1833.833333	1604.583333	2.960766109	3.33066904	Higher in TRIM24	57	43	489	328	475	4756	1462	787	761	2762
ENSMUSG00000044122.14	Proca1	7.13E-05	0.00127753	4	6	667.75	127.8333333	539.9166667	2.542892197	2.752862067	Higher in Normal	1255	1060	167	189	142	28	105	45	206	241
ENSMUSG00000102548.1	Gm16701	7.19E-05	0.001285522	4	6	0.75	12.16666667	11.41666667	3.387207666	4.066882874	Higher in TRIM24	2	0	0	1	6	17	4	13	15	18
ENSMUSG00000022763.16	Aifm3	7.20E-05	0.001288089	4	6	163	22.16666667	140.8333333	2.693165288	2.949180176	Higher in Normal	220	79	180	173	13	33	2	2	41	42
ENSMUSG00000058743.6	Kcnj14	7.27E-05	0.001297806	4	6	193.25	37.16666667	156.0833333	2.636854935	2.874688343	Higher in Normal	171	486	48	68	25	12	12	24	52	98
ENSMUSG00000111774.1	Gm38398	7.33E-05	0.001306698	4	6	57.75	7.166666667	50.58333333	2.974761644	3.337576937	Higher in Normal	112	97	11	11	2	3	4	6	16	12
ENSMUSG00000037362.8	Nov	7.38E-05	0.001312823	4	6	282.5	1864.166667	1581.666667	2.553117611	2.773470591	Higher in TRIM24	121	150	417	442	459	1320	3539	423	4513	931
ENSMUSG00000003032.8	Klf4	7.44E-05	0.001321192	4	6	15165	2511	12654	2.579557289	2.801112758	Higher in Normal	1722	2254	26577	30107	1435	955	2065	950	1661	8000
ENSMUSG00000027220.2	Syt13	7.56E-05	0.001337554	4	6	133.25	2796.666667	2663.416667	3.304048046	3.874821337	Higher in TRIM24	102	21	204	206	250	286	3738	479	107	11920
ENSMUSG00000068037.10	Mas1	7.56E-05	0.001337554	4	6	36.75	6	30.75	2.744418412	3.017593302	Higher in Normal	82	28	23	14	4	3	3	0	8	18
ENSMUSG00000021087.18	Rtn1	7.68E-05	0.001354672	4	6	1384	209.6666667	1174.333333	2.351042902	2.514891898	Higher in Normal	311	586	2150	2489	248	82	342	288	117	181
ENSMUSG00000033327.16	Tnxb	7.69E-05	0.00135626	4	6	1992.5	392.8333333	1599.666667	2.429828131	2.612347299	Higher in Normal	2413	2781	1591	1185	174	92	507	77	1140	367
ENSMUSG00000046179.17	E2f8	7.70E-05	0.001356828	4	6	206.75	1133.333333	926.5833333	2.446695897	2.638395755	Higher in TRIM24	108	28	343	348	462	1704	931	467	2046	1190
ENSMUSG00000045027.6	Prss22	7.73E-05	0.001361028	4	6	152.5	2259.5	2107	3.335415161	3.930897556	Higher in TRIM24	62	46	206	296	390	2113	845	85	9582	542
ENSMUSG00000033389.16	Arhgap44	7.75E-05	0.001363389	4	6	967.5	156	811.5	2.372529193	2.5414939	Higher in Normal	298	390	1603	1579	318	30	120	89	244	135
ENSMUSG00000010529.6	Gm266	7.82E-05	0.001376263	4	6	1654.5	152.5	1502	3.135688081	3.574219037	Higher in Normal	311	756	2387	3164	102	4	83	25	568	133
ENSMUSG00000053862.6	Slc51b	7.85E-05	0.001379966	4	6	11.75	0.166666667	11.58333333	4.115002927	5.568774583	Higher in Normal	29	7	9	2	0	0	0	1	0	0

ENSMUSG00000005580.11	Adcy9	8.11E-05	0.001420685	4	6	837.5	131.5	706	2.432090971	2.616591052	Higher in Normal	214	111	2056	969	151	90	89	45	215	199
ENSMUSG00000046523.4	Kctd4	8.17E-05	0.001428936	4	6	796.75	148.1666667	648.5833333	2.708467247	2.972771471	Higher in Normal	309	420	1215	1243	16	39	92	10	124	608
ENSMUSG00000112433.1	Gm30122	8.18E-05	0.001429738	4	6	65.5	11.83333333	53.66666667	2.648318811	2.893998365	Higher in Normal	106	99	29	28	7	9	3	1	18	33
ENSMUSG00000107451.1	Gm44421	8.24E-05	0.00143664	4	6	42.5	2.5	40	3.503739877	4.196774176	Higher in Normal	118	45	3	4	4	1	4	1	4	1
ENSMUSG00000048572.4	Tmem252	8.29E-05	0.001444993	4	6	2292.75	360.1666667	1932.583333	2.439700415	2.626659804	Higher in Normal	260	380	3551	4980	387	160	337	243	370	664
ENSMUSG00000039099.7	Wdr93	8.35E-05	0.001455272	4	6	162.25	20	142.25	3.131331017	3.571746109	Higher in Normal	364	198	46	41	12	1	2	6	46	53
ENSMUSG00000021567.15	Nkd2	8.53E-05	0.001482074	4	6	428.75	5166.5	4737.75	3.243023523	3.784203464	Higher in TRIM24	320	144	548	703	20050	1116	1602	421	6849	961
ENSMUSG00000046215.3	Rprml	8.68E-05	0.001504289	4	6	127	7.833333333	119.1666667	3.507889258	4.196305818	Higher in Normal	441	59	4	4	6	3	6	11	6	15
ENSMUSG00000041261.9	Car8	8.69E-05	0.001504289	4	6	983.25	52.66666667	930.5833333	3.629787785	4.411494352	Higher in Normal	243	454	1641	1595	9	6	12	0	256	33
ENSMUSG00000030020.13	Prickle2	8.69E-05	0.001504289	4	6	1662	265.1666667	1396.833333	2.391433905	2.567647476	Higher in Normal	478	210	3368	2592	158	112	214	241	569	297
ENSMUSG00000109282.1	Gm45188	8.78E-05	0.001518273	4	6	20.75	1.5	19.25	3.244961284	3.786775442	Higher in Normal	8	26	40	9	1	4	0	0	2	2
ENSMUSG00000093806.1	Asmt	8.89E-05	0.001532764	4	6	10.25	0.833333333	9.416666667	3.241273421	3.772875259	Higher in Normal	6	7	17	11	0	0	0	1	2	2
ENSMUSG00000030747.5	Dgat2	8.93E-05	0.001537528	4	6	20155	1648.666667	18506.33333	3.342721483	3.910553779	Higher in Normal	52492	21451	2952	3725	893	399	651	236	7217	496
ENSMUSG00000041658.12	Rragb	8.95E-05	0.00153883	4	6	138	20.66666667	117.3333333	2.459792226	2.654644924	Higher in Normal	94	72	180	206	2	10	53	19	25	15
ENSMUSG00000037973.6	Ccdc129	8.98E-05	0.001542853	4	6	3685.25	208.3333333	3476.916667	3.537620398	4.246230715	Higher in Normal	798	2023	5877	6043	244	8	24	5	886	83
ENSMUSG00000058099.15	Nfam1	9.01E-05	0.001546472	4	6	225.25	1350.666667	1125.416667	2.329691992	2.496099598	Higher in TRIM24	285	427	92	97	497	1409	2312	1176	1200	1510
ENSMUSG00000050010.8	Shisa3	9.12E-05	0.001564144	4	6	51.25	7.333333333	43.91666667	2.515645415	2.727361899	Higher in Normal	7	32	69	97	8	4	13	3	10	6
ENSMUSG00000023034.6	Nr4a1	9.17E-05	0.001572071	4	6	27746.25	1387	26359.25	3.48301699	4.151717922	Higher in Normal	522	464	50964	59035	3595	438	1372	300	883	1734
ENSMUSG00000097214.1	Gm19791	9.25E-05	0.001582956	4	6	0.75	16.16666667	15.41666667	3.382274842	4.107739845	Higher in TRIM24	1	2	0	0	5	7	23	12	4	46
ENSMUSG00000042707.6	Dnali1	9.32E-05	0.001593021	4	6	27.5	2.5	25	3.292654891	3.835417262	Higher in Normal	73	5	17	15	2	0	1	0	5	7
ENSMUSG00000096858.4	Olfr805	9.42E-05	0.001608017	4	6	7.75	383	375.25	3.908033907	5.23068569	Higher in TRIM24	22	7	2	0	1	174	423	428	9	1263
ENSMUSG00000111293.1	Gm34006	9.46E-05	0.001613067	4	6	88.75	13	75.75	2.549151029	2.770515629	Higher in Normal	61	78	83	133	26	1	5	9	30	7
ENSMUSG00000027581.12	Stmn3	9.50E-05	0.001618255	4	6	29.75	4	25.75	2.990904257	3.371425381	Higher in Normal	61	24	26	8	0	1	1	2	6	14
ENSMUSG00000039661.14	Dusp26	9.56E-05	0.001626037	4	6	229.25	31.66666667	197.5833333	2.727242506	3.00516688	Higher in Normal	229	372	151	165	87	5	28	3	31	36
ENSMUSG00000096847.1	Tmem151b	9.63E-05	0.001633922	4	6	239.75	22.33333333	217.4166667	2.908036819	3.256234199	Higher in Normal	99	46	422	392	5	34	43	1	27	24
ENSMUSG00000034486.8	Gbx2	9.74E-05	0.001649253	4	6	4.5	29.83333333	25.33333333	2.487285232	2.699238182	Higher in TRIM24	5	3	2	8	14	22	29	13	79	22
ENSMUSG00000033350.7	Chst2	9.76E-05	0.001652105	4	6	646.25	3166.833333	2520.583333	2.35947408	2.535028625	Higher in TRIM24	426	246	796	1117	7395	3412	3303	1182	1204	2505
ENSMUSG00000056078.5	Lipm	9.81E-05	0.001658302	4	6	662.75	67.83333333	594.9166667	3.063001913	3.48096979	Higher in Normal	463	639	773	776	59	13	15	2	282	36
ENSMUSG00000096632.2	Igkv9-124	0.000101246	0.001701187	4	6	64.25	4.166666667	60.08333333	3.27485224	3.822595063	Higher in Normal	11	67	81	98	8	2	13	0	1	1
ENSMUSG00000022496.5	Tnfrsf17	0.000101667	0.001706474	4	6	38.25	4.166666667	34.08333333	3.186369323	3.675429809	Higher in Normal	45	91	13	4	2	0	4	2	8	9
ENSMUSG00000090877.3	Hspa1b	0.000102075	0.001712389	4	6	62492.75	4297	58195.75	3.421675023	4.057657993	Higher in Normal	781	1111	117010	131069	1423	740	1584	3988	4034	14013
ENSMUSG00000042662.16	Dusp15	0.000103814	0.001738942	4	6	59	7.833333333	51.16666667	2.959282355	3.331274865	Higher in Normal	19	81	55	81	1	2	0	3	20	21
ENSMUSG00000071658.4	Gng3	0.000105439	0.001760092	4	6	43.75	9.166666667	34.58333333	2.363487081	2.53807329	Higher in Normal	93	31	39	12	8	5	3	4	12	23
ENSMUSG00000078554.2	Fam229a	0.00010674	0.001774992	4	6	10	0.666666667	9.333333333	3.338090802	3.957783155	Higher in Normal	5	7	12	16	2	0	0	0	1	1
ENSMUSG00000004885.5	Crabp2	0.000107493	0.001783626	4	6	1119.5	10402.33333	9282.833333	2.47180934	2.680333139	Higher in TRIM24	839	356	1340	1943	3562	749	6943	1898	7060	42202
ENSMUSG00000001773.14	Folh1	0.00010768	0.001785746	4	6	268.5	15	253.5	3.832992901	4.868989833	Higher in Normal	406	650	11	7	0	2	5	2	43	38
ENSMUSG00000055737.12	Ghr	0.000109027	0.001807098	4	6	12529	2431	10098	2.678928333	2.945137655	Higher in Normal	22057	23449	2315	2295	1909	635	1384	591	2633	7434
ENSMUSG00000047990.6	C2cd4a	0.000110011	0.001820443	4	6	132.75	8.166666667	124.5833333	3.186016638	3.683767152	Higher in Normal	23	2	196	310	9	9	13	7	8	3
ENSMUSG00000050071.8	Bex1	0.000110182	0.001821571	4	6	173.75	18.16666667	155.5833333	2.896171571	3.246231327	Higher in Normal	72	156	187	280	2	4	71	7	13	12
ENSMUSG00000035459.17	Stab2	0.000110831	0.001827918	4	6	123.75	28.66666667	95.08333333	2.378853086	2.557238492	Higher in Normal	205	59	124	107	2	6	23	9	40	92
ENSMUSG00000110298.1	Gm8189	0.000110883	0.001827918	4	6	106.5	21.33333333	85.16666667	2.678226484	2.943595286	Higher in Normal	128	189	68	41	9	2	8	3	39	67
ENSMUSG00000043673.11	Kcns3	0.000111939	0.001841342	4	6	512.25	56.5	455.75	3.003149043	3.401496334	Higher in Normal	851	944	103	151	13	30	172	8	66	50
ENSMUSG00000097639.2	Platr4	0.000112239	0.001845271	4	6	64.75	7.166666667	57.58333333	3.101330831	3.548220297	Higher in Normal	83	75	63	38	7	0	0	1	22	13
ENSMUSG00000039252.11	Lgi2	0.000113365	0.001859765	4	6	65.75	653.1666667	587.4166667	2.735147596	3.035253079	Higher in TRIM24	79	127	26	31	66	98	1225	746	695	1089
ENSMUSG00000030724.7	Cd19	0.000113544	0.001861712	4	6	289.25	27.33333333	261.9166667	3.053035944	3.477164365	Higher in Normal	44	497	324	292	2	10	101	11	21	19
ENSMUSG00000083355.1	Gm11581	0.000113644	0.001862344	4	6	17	1.166666667	15.83333333	3.361674574	4.007316032	Higher in Normal	40	15	11	2	0	1	1	1	3	1
ENSMUSG00000051041.7	Olfml1	0.000113827	0.001864337	4	6	488	58.66666667	429.3333333	2.975098814	3.361228159	Higher in Normal	947	863	71	71	23	22	85	15	159	48
ENSMUSG00000047502.14	Mroh7	0.000114749	0.00187641	4	6	37.25	5.5	31.75	2.726076198	3.010501769	Higher in Normal	66	11	49	23	0	2	7	1	11	12
ENSMUSG00000024064.13	Galnt14	0.000114859	0.0018772	4	6	173.5	21	152.5	3.045448861	3.464117054	Higher in Normal	146	28	254	266	6	5	1	1	46	67
ENSMUSG00000000305.12	Cdh4	0.000115668	0.001888392	4	6	90.5	17.83333333	72.66666667	2.630661982	2.881431106	Higher in Normal	197	32	92	41	3	3	6	6	23	66
ENSMUSG00000104088.1	Gm38275	0.000116404	0.001899393	4	6	2.25	18	15.75	2.634667453	2.906553547	Higher in TRIM24	4	2	3	0	7	7	17	25	23	29
ENSMUSG00000028610.16	Dmrtb1	0.000117063	0.001909118	4	6	40	1.666666667	38.33333333	3.912545001	5.090101323	Higher in Normal	90	1	59	10	0	0	0	0	4	6
ENSMUSG00000042436.12	Mfap4	0.000117231	0.001909811	4	6	342	2090	1748	2.468664757	2.679019832	Higher in TRIM24	215	59	454	640	331	1065	4745	1119	3265	2015
ENSMUSG00000055194.3	Actbl2	0.000117617	0.001914385	4	6	11.75	0.833333333	10.91666667	3.422560998	4.095996301	Higher in Normal	23	3	11	10	1	0	0	0	1	3
ENSMUSG00000079163.3	Gm15498	0.000117645	0.001914385	4	6	24.5	0.5	24	4.138052552	5.777754226	Higher in Normal	28	54	16	0	0	0	1	0	2	0
ENSMUSG00000040966.9	Slc22a2	0.000118203	0.00192051	4	6	25.75	1.833333333	23.91666667	3.595912248	4.404109319	Higher in Normal	52	41	5	5	1	0	0	0	4	6
ENSMUSG00000085502.1	Gm12320	0.000120773	0.001954743	4	6	33.75	4.333333333	29.41666667	2.767392932	3.071231481	Higher in Normal	44	5	37	49	3	0	1	4	11	7
ENSMUSG00000027297.14	Ltk	0.000120823	0.001954743	4	6	301.25	42.5	258.75	2.660619803	2.924808135	Higher in Normal	195	136	445	429	64	12	4	6	115	54
ENSMUSG00000062151.13	Unc13c	0.000121757	0.001967765	4	6	35	219.5	184.5	2.423001645	2.622216379	Higher in TRIM24	86	29	20	5	187	240	119	249	202	320
ENSMUSG00000025215.10	Tlx1	0.000121869	0.00196852	4	6	11.5	1.166666667	10.33333333	2.975035699	3.386189014	Higher in Normal	10	8	15	13	0	1	1	0	4	1
ENSMUSG00000042641.18	Rgsl1	0.000122225	0.001971174	4	6	70	13.66666667	56.33333333	2.572713986	2.808627007	Higher in Normal	152	70	37	21	5	6	2	7	20	42
ENSMUSG00000084918.1	Gm12708	0.000122227	0.001971174	4	6	2.75	17.83333333	15.08333333	2.422294636	2.627125771	Higher in TRIM24	3	4	4	0	11	20	13	11	25	27
ENSMUSG00000000706.15	Btn1a1	0.000122815	0.001977559	4	6	9584.25	837.5	8746.75	3.022992092	3.436699948	Higher in Normal	4997	1987	14877	16476	1474	362	257	42	2625	265
ENSMUSG00000094006.2	Igkv4-59	0.000123327	0.001981711	4	6	126.25	19.66666667	106.5833333	2.833900509	3.162800185	Higher in Normal	25	268	86	126	13	0	16	6	34	49
ENSMUSG00000049972.4	Skint9	0.000123458	0.00198262	4	6	10.5	0.333333333	10.16666667	3.867406825	4.911591151	Higher in Normal	13	2	12	15	0	0	0	0	0	2
ENSMUSG00000031927.9	1700012B09Rik	0.000125716	0.002013569	4	6	0.75	20.5	19.75	3.710196194	4.86378177	Higher in TRIM24	2	0	1	0	29	2	2	52	9	29
ENSMUSG00000025092.6	Hspa12a	0.000129209	0.002059775	4	6	1049.25	187.6666667	861.5833333	2.550407189	2.781832532	Higher in Normal	1823	1881	286	207	36	119	182	169	260	360
ENSMUSG00000024164.15	C3	0.000131023	0.002086499	4	6	53146.5	9684.166667	43462.33333	2.342766371	2.517505848	Higher in Normal	17338	15632	90717	88899	5813	1650	7361	4568	28073	10640
ENSMUSG00000027574.15	Nkain4	0.000133263	0.002121058	4	6	29.25	5.333333333	23.91666667	2.55274527	2.784808699	Higher in Normal	61	16	28	12	3	0	6	2	7	14
ENSMUSG00000022438.6	Parvb	0.00013569	0.002150701	4	6	651.75	4192.333333	3540.583333	2.424816758	2.627215222	Higher in TRIM24	563	497	752	795	352	5527	5485	2515	1098	10177
ENSMUSG00000040212.12	Emp3	0.000135947	0.002151437	4	6	681.5	5149.5	4468	2.413381522	2.612630019	Higher in TRIM24	641	1379	333	373	882	3652	6321	5031	1645	13366
ENSMUSG00000026826.13	Nr4a2	0.00013754	0.002173226	4	6	2571.25	300.8333333	2270.416667	2.766066315	3.074238128	Higher in Normal	204	144	4903	5034	99	170	325	240	342	629
ENSMUSG00000095210.2	Ighv5-9-1	0.000138255	0.002183396	4	6	36.25	2.5	33.75	3.439651382	4.145328884	Higher in Normal	1	97	21	26	3	0	6	1	1	4
ENSMUSG00000033676.13	Gabrb3	0.00013888	0.002187597	4	6	15.25	161.3333333	146.0833333	3.025847985	3.478667391	Higher in TRIM24	40	7	12	2	27	262	337	36	214	92
ENSMUSG00000004296.14	Il12b	0.000141202	0.002221865	4	6	389.75	47.5	342.25	2.757740378	3.063964791	Higher in Normal	85	21	699	754	19	11	94	18	60	83
ENSMUSG00000048938.12	Nr1h5	0.000142196	0.002234042	4	6	22.25	464.6666667	442.4166667	3.355354808	4.044888259	Higher in TRIM24	73	3	9	4	800	30	17	187	609	1145
ENSMUSG00000112824.1	Gm47917	0.000142704	0.002239718	4	6	74.25	15.16666667	59.08333333	2.700127888	2.983678046	Higher in Normal	84	128	32	53	6	5	1	4	9	66
ENSMUSG00000033831.5	Fgb	0.000143174	0.002245938	4	6	1001.25	67.66666667	933.5833333	3.198265455	3.721065841	Higher in Normal	264	303	1486	1952	51	5	292	16	24	18
ENSMUSG00000100490.1	Gm28455	0.000143823	0.002253791	4	6	32.75	3.666666667	29.08333333	2.916601769	3.293364164	Higher in Normal	48	4	45	34	2	2	0	1	11	6
ENSMUSG00000046070.8	Igfals	0.000145153	0.002272303	4	6	1419.5	134.8333333	1284.666667	3.033191189	3.463999343	Higher in Normal	913	871	1630	2264	58	8	261	14	444	24
ENSMUSG00000048378.2	Olfr1394	0.000145704	0.002279745	4	6	15.5	0.666666667	14.83333333	3.936638641	5.145395379	Higher in Normal	13	31	15	3	0	0	0	0	0	4
ENSMUSG00000076607.1	Igkj4	0.000147155	0.00229891	4	6	9.25	0.333333333	8.916666667	3.780095853	4.855755097	Higher in Normal	3	20	9	5	0	0	1	0	0	1

ENSMUSG00000072624.2	Gm5460	0.000148403	0.002316016	4	6	16.5	0.833333333	15.66666667	3.693881792	4.712603703	Higher in Normal	29	32	2	3	0	1	0	0	2	2
ENSMUSG00000092094.5	Zfp804b	0.000148554	0.002316016	4	6	25.75	2	23.75	3.279194317	3.87272996	Higher in Normal	40	2	52	9	2	2	0	0	2	6
ENSMUSG00000025930.6	Msc	0.000148783	0.002318402	4	6	315.5	43.33333333	272.1666667	2.488719159	2.707083254	Higher in Normal	225	298	316	423	10	14	50	105	64	17
ENSMUSG00000054909.11	Wbscr25	0.000151219	0.002347936	4	6	107.5	15.83333333	91.66666667	2.741584334	3.044519109	Higher in Normal	218	35	106	71	13	4	3	1	47	27
ENSMUSG00000005672.12	Kit	0.000151557	0.002350791	4	6	11731.25	1881.5	9849.75	2.455037982	2.663664096	Higher in Normal	4399	6382	19401	16743	2664	357	1307	375	5409	1177
ENSMUSG00000034472.13	Rasd2	0.000153309	0.002374926	4	6	370.25	31.33333333	338.9166667	2.942358916	3.333132062	Higher in Normal	47	104	626	704	72	21	56	2	18	19
ENSMUSG00000033717.5	Adra2a	0.00015402	0.002382784	4	6	240.75	1822.666667	1581.916667	2.799263721	3.142081705	Higher in TRIM24	101	175	309	378	66	1432	3377	3658	895	1508
ENSMUSG00000051314.11	Ffar2	0.000154167	0.002382784	4	6	1332.5	151	1181.5	3.049576656	3.493678414	Higher in Normal	1273	3835	83	139	67	40	168	138	320	173
ENSMUSG00000011382.8	Dhdh	0.00015462	0.002387352	4	6	2233.75	365	1868.75	2.648172156	2.918475382	Higher in Normal	4810	3429	320	376	271	171	264	154	839	491
ENSMUSG00000030732.13	Chrdl2	0.000155261	0.002395829	4	6	188.5	18.16666667	170.3333333	2.897133515	3.268367466	Higher in Normal	176	5	235	338	6	21	34	2	26	20
ENSMUSG00000021943.6	Gdf10	0.000155326	0.002395829	4	6	249	47.33333333	201.6666667	2.481067857	2.697871359	Higher in Normal	289	368	150	189	26	8	39	8	159	44
ENSMUSG00000015843.10	Rxrg	0.000155883	0.002401861	4	6	310	25.33333333	284.6666667	3.339585347	3.9667709	Higher in Normal	569	606	25	40	27	8	8	1	90	18
ENSMUSG00000053719.10	Klk1b26	0.000155954	0.002401861	4	6	19.25	1.333333333	17.91666667	3.598928577	4.460832689	Higher in Normal	45	21	9	2	1	0	0	0	1	6
ENSMUSG00000052551.16	Adarb2	0.000157524	0.002423599	4	6	24.25	4.166666667	20.08333333	2.738627973	3.038543415	Higher in Normal	28	28	31	10	2	0	3	0	5	15
ENSMUSG00000042734.6	Ttc9	0.000158663	0.002438647	4	6	279.5	1298.5	1019	2.322897583	2.502092949	Higher in TRIM24	65	97	447	509	2252	1730	1338	635	810	1026
ENSMUSG00000098403.1	Gm27415	0.0001599	0.00245395	4	6	28	2.666666667	25.33333333	2.979049494	3.397591882	Higher in Normal	12	4	61	35	1	3	1	0	7	4
ENSMUSG00000031489.15	Adrb3	0.000160697	0.002462451	4	6	5529.25	231.1666667	5298.083333	3.84057613	4.990579752	Higher in Normal	12200	9780	73	64	171	20	53	7	1047	89
ENSMUSG00000044405.4	Adig	0.000162796	0.00248836	4	6	3453.25	202.3333333	3250.916667	3.606305942	4.471308058	Higher in Normal	6701	6568	220	324	192	23	45	5	889	60
ENSMUSG00000027520.15	Zdbf2	0.000163195	0.002492723	4	6	69.5	7.166666667	62.33333333	3.115726278	3.598088146	Higher in Normal	14	7	178	79	2	0	6	1	11	23
ENSMUSG00000031289.10	Il13ra2	0.000164363	0.002504771	4	6	27.25	790.3333333	763.0833333	3.413747521	4.181275202	Higher in TRIM24	48	48	7	6	15	60	125	1006	144	3392
ENSMUSG00000104427.1	Gm38368	0.000165154	0.002515567	4	6	15.25	1.333333333	13.91666667	3.241725209	3.829207571	Higher in Normal	36	13	8	4	2	1	0	0	1	4
ENSMUSG00000031323.8	Dmrtc1a	0.000165724	0.00252298	4	6	17.5	0.666666667	16.83333333	3.705166023	4.730989122	Higher in Normal	11	6	17	36	0	0	0	0	4	0
ENSMUSG00000106957.1	Gm43085	0.000166102	0.00252748	4	6	104	16.33333333	87.66666667	2.951593255	3.348887667	Higher in Normal	82	155	71	108	12	1	2	0	18	65
ENSMUSG00000053963.7	Stum	0.000166852	0.002535092	4	6	127.5	20.5	107	2.512300015	2.740605095	Higher in Normal	57	27	208	218	22	2	13	4	51	31
ENSMUSG00000106004.4	Gm43391	0.000168524	0.002557941	4	6	27.75	2.333333333	25.41666667	3.234441003	3.8097685	Higher in Normal	71	14	20	6	0	2	0	1	7	4
ENSMUSG00000091373.4	Gm8810	0.000169278	0.002566828	4	6	60.25	4.666666667	55.58333333	3.27343719	3.863385215	Higher in Normal	9	34	108	90	3	0	1	0	19	5
ENSMUSG00000074472.10	Zfp872	0.000170766	0.002585525	4	6	58.25	3.833333333	54.41666667	3.424516677	4.136927151	Higher in Normal	106	62	33	32	0	1	2	1	19	0
ENSMUSG00000029195.10	Klb	0.000173888	0.002628863	4	6	452.75	36.5	416.25	3.309787627	3.927930401	Higher in Normal	1206	529	45	31	33	7	11	8	144	16
ENSMUSG00000071177.4	Serpina1d	0.000174669	0.002639276	4	6	30.75	3.166666667	27.58333333	2.847385651	3.214048345	Higher in Normal	63	23	11	26	4	4	2	2	7	0
ENSMUSG00000110622.1	Iqcn	0.000175748	0.002649096	4	6	29.5	2.666666667	26.83333333	3.205482743	3.753064209	Higher in Normal	24	2	73	19	3	0	1	0	3	9
ENSMUSG00000047182.6	Irs3	0.000179137	0.002694847	4	6	699.25	53	646.25	3.378515177	4.052800171	Higher in Normal	1391	1374	14	18	20	23	42	21	172	40
ENSMUSG00000031144.15	Syp	0.000179907	0.002705089	4	6	387.25	60.66666667	326.5833333	2.812080592	3.153350398	Higher in Normal	474	967	59	49	28	11	46	36	126	117
ENSMUSG00000018486.2	Wnt9b	0.000180455	0.002711993	4	6	15.75	2.166666667	13.58333333	2.671866128	2.961967783	Higher in Normal	15	3	27	18	0	1	2	2	3	5
ENSMUSG00000083307.1	AA414768	0.000181631	0.002728314	4	6	38.25	259.1666667	220.9166667	2.479340007	2.708876216	Higher in TRIM24	56	46	23	28	47	125	429	462	65	427
ENSMUSG00000021223.13	Papln	0.000182021	0.002731753	4	6	3549.25	286	3263.25	3.076155635	3.548248266	Higher in Normal	761	1353	7714	4369	761	34	88	21	694	118
ENSMUSG00000102376.1	Gm37975	0.000182039	0.002731753	4	6	140.25	23.83333333	116.4166667	2.815758571	3.157701252	Higher in Normal	281	121	56	103	8	3	5	1	50	76
ENSMUSG00000086455.1	Gm11815	0.000182623	0.002739167	4	6	0.25	7.666666667	7.416666667	3.608419293	4.625402227	Higher in TRIM24	1	0	0	0	5	3	8	8	15	7
ENSMUSG00000074971.4	Fibin	0.000183001	0.002742372	4	6	341.5	2285.833333	1944.333333	2.410498733	2.618518345	Higher in TRIM24	455	429	213	269	294	1212	6693	1152	1503	2861
ENSMUSG00000038663.7	Fsd2	0.000183665	0.002749372	4	6	324.5	36	288.5	3.043015867	3.49807984	Higher in Normal	410	827	26	35	20	16	73	7	64	36
ENSMUSG00000094689.1	Ighv1-81	0.000184006	0.002752592	4	6	11	1.166666667	9.833333333	3.01710373	3.479871811	Higher in Normal	9	19	8	8	1	1	0	0	3	2
ENSMUSG00000047361.16	Gm973	0.000184983	0.002762322	4	6	135.25	30	105.25	2.416643556	2.61981195	Higher in Normal	187	76	120	158	21	3	7	3	57	89
ENSMUSG00000041449.16	Serpina3h	0.000188077	0.002803019	4	6	27.25	286.8333333	259.5833333	2.824949912	3.192767117	Higher in TRIM24	33	18	26	32	218	33	799	10	106	555
ENSMUSG00000113757.1	Gm47507	0.000188267	0.002803102	4	6	1.25	19.16666667	17.91666667	3.393237393	4.203167603	Higher in TRIM24	2	1	1	1	1	55	24	7	17	11
ENSMUSG00000038296.14	Galnt18	0.00018974	0.00282228	4	6	3903.25	542	3361.25	2.499837624	2.729505409	Higher in Normal	837	737	6280	7759	841	155	667	173	1084	332
ENSMUSG00000038227.15	Hoxa9	0.000190266	0.002827342	4	6	272.75	41	231.75	2.845653585	3.20497304	Higher in Normal	395	573	75	48	67	9	14	4	54	98
ENSMUSG00000078949.2	R3hdml	0.000190544	0.002829767	4	6	359.5	47.33333333	312.1666667	2.793906259	3.130747462	Higher in Normal	366	84	373	615	3	2	62	58	21	138
ENSMUSG00000086679.2	Gm15551	0.000190615	0.002829767	4	6	88	2.5	85.5	3.93902816	5.349512175	Higher in Normal	194	157	1	0	3	1	0	2	8	1
ENSMUSG00000001670.13	Tat	0.000190902	0.002831257	4	6	17	2	15	2.851195995	3.217885656	Higher in Normal	19	3	22	24	0	1	3	0	4	4
ENSMUSG00000059027.14	9630013D21Rik	0.000190999	0.002831317	4	6	290	30.33333333	259.6666667	3.227159055	3.797857142	Higher in Normal	681	422	30	27	3	2	8	29	55	85
ENSMUSG00000067594.5	Krt77	0.000192833	0.002852957	4	6	176	21.5	154.5	2.977465579	3.399647652	Higher in Normal	66	18	260	360	6	8	2	1	39	73
ENSMUSG00000048031.15	Fcrl5	0.000193724	0.002863348	4	6	99	12.33333333	86.66666667	2.948300999	3.35689217	Higher in Normal	66	130	92	108	4	0	30	0	13	27
ENSMUSG00000023243.8	Kcnk5	0.000194072	0.002867107	4	6	4477.25	600.6666667	3876.583333	2.756030289	3.078034788	Higher in Normal	730	886	7648	8645	445	24	291	203	1429	1212
ENSMUSG00000045053.1	Kcng3	0.000194199	0.002867596	4	6	22	1.833333333	20.16666667	3.240208372	3.829385357	Higher in Normal	34	1	35	18	1	0	0	1	4	5
ENSMUSG00000048096.7	Lmod1	0.000198645	0.00292615	4	6	2423.75	205	2218.75	2.996599534	3.432342558	Higher in Normal	674	1123	3388	4510	681	16	111	20	310	92
ENSMUSG00000100593.1	1700119H24Rik	0.00019919	0.002929916	4	6	17	2	15	2.989857104	3.424385661	Higher in Normal	37	6	12	13	1	0	0	1	4	6
ENSMUSG00000096499.2	Ighv1-5	0.000199854	0.002938275	4	6	25	2.833333333	22.16666667	2.84402161	3.210742753	Higher in Normal	18	2	34	46	4	1	0	2	3	7
ENSMUSG00000095565.2	Ighv2-9-1	0.000203738	0.002980975	4	6	12.75	0.833333333	11.91666667	3.364807464	4.108486423	Higher in Normal	23	20	3	5	1	0	2	1	0	1
ENSMUSG00000087642.3	Ighe	0.000204739	0.00299275	4	6	173.25	7.333333333	165.9166667	3.607817565	4.527874187	Higher in Normal	17	3	283	390	0	5	21	0	5	13
ENSMUSG00000026380.10	Tfcp2l1	0.000205371	0.003000542	4	6	1301.75	201.1666667	1100.583333	2.346453085	2.534754054	Higher in Normal	601	584	2258	1764	60	316	189	50	457	135
ENSMUSG00000023906.2	Cldn6	0.000205486	0.003000781	4	6	152.75	17.83333333	134.9166667	2.570744543	2.828209452	Higher in Normal	46	57	216	292	4	25	44	12	13	9
ENSMUSG00000083872.1	Gm11928	0.000205911	0.003005557	4	6	13.25	0	13.25	4.213189172	6.273082965	Higher in Normal	41	8	3	1	0	0	0	0	0	0
ENSMUSG00000105095.1	8430422M14Rik	0.000208091	0.003033014	4	6	132.5	20.83333333	111.6666667	2.717645956	3.028499048	Higher in Normal	106	292	45	87	6	4	3	21	59	32
ENSMUSG00000087336.2	Gm15860	0.000211928	0.003078615	4	6	36.5	7.5	29	2.422416255	2.631907775	Higher in Normal	60	36	26	24	1	3	5	1	19	16
ENSMUSG00000112963.1	Gm6093	0.000212383	0.003083755	4	6	18.75	222.1666667	203.4166667	3.018271259	3.503473475	Higher in TRIM24	48	8	13	6	2	269	264	106	425	267
ENSMUSG00000091418.2	Gm3164	0.000214951	0.003119554	4	6	16.5	1.333333333	15.16666667	3.08623852	3.601699924	Higher in Normal	4	7	30	25	0	1	3	0	3	1
ENSMUSG00000030546.14	Plin1	0.000215133	0.003120715	4	6	18254.25	826.3333333	17427.91667	3.747228753	4.847334147	Higher in Normal	36179	36156	331	351	866	57	208	25	3609	193
ENSMUSG00000039676.3	Capsl	0.000216969	0.003143552	4	6	503.75	46.5	457.25	2.944007495	3.359484433	Higher in Normal	106	297	680	932	114	1	29	15	103	17
ENSMUSG00000053219.15	Raet1e	0.000217017	0.003143552	4	6	7.5	59.33333333	51.83333333	2.699653661	3.021947576	Higher in TRIM24	15	5	7	3	8	27	93	124	25	79
ENSMUSG00000030889.14	Vwa3a	0.000218673	0.003164519	4	6	242.25	29.33333333	212.9166667	3.170453342	3.715991198	Higher in Normal	413	209	229	118	4	1	1	2	70	98
ENSMUSG00000028979.17	Masp2	0.000222261	0.003213391	4	6	37.25	5.666666667	31.58333333	2.955218794	3.370332704	Higher in Normal	54	30	36	29	1	0	1	0	8	24
ENSMUSG00000108441.1	Gm32846	0.000224096	0.003236108	4	6	12.5	0.333333333	12.16666667	3.839376456	5.100422528	Higher in Normal	1	18	15	16	0	0	0	0	2	0
ENSMUSG00000027748.11	Trpc4	0.000224151	0.003236108	4	6	69.25	10.66666667	58.58333333	2.888687321	3.274501077	Higher in Normal	107	25	70	75	4	0	1	1	17	41
ENSMUSG00000092474.1	Gm20478	0.000225438	0.003250058	4	6	25	1.333333333	23.66666667	3.276290877	3.953771195	Higher in Normal	3	15	46	36	0	4	0	2	1	1
ENSMUSG00000093973.1	Mrgpra2a	0.000225739	0.003252861	4	6	0	11.33333333	11.33333333	4.043278216	5.737398953	Higher in TRIM24	0	0	0	0	13	0	7	3	32	13
ENSMUSG00000095442.1	Ighv1-4	0.000227012	0.003269657	4	6	41.75	3	38.75	3.181603559	3.754527457	Higher in Normal	2	20	77	68	5	3	4	0	0	6
ENSMUSG00000029368.10	Alb	0.00022715	0.003270104	4	6	249	20.33333333	228.6666667	3.379104109	4.088856108	Higher in Normal	534	450	8	4	2	14	16	4	39	47
ENSMUSG00000070298.6	Trcg1	0.00023036	0.003308491	4	6	35.75	3	32.75	3.163147815	3.725073144	Higher in Normal	99	4	36	4	0	3	2	3	3	7
ENSMUSG00000105260.1	Gm40040	0.00023212	0.003327486	4	6	20.25	2.166666667	18.08333333	3.112440756	3.627620221	Higher in Normal	37	11	25	8	0	0	1	0	6	6
ENSMUSG00000087228.1	Gm12827	0.000233819	0.003350269	4	6	9.75	0.166666667	9.583333333	3.858871548	5.248946013	Higher in Normal	24	2	8	5	0	1	0	0	0	0
ENSMUSG00000027224.14	Duoxa1	0.000234687	0.003359546	4	6	71.25	750.8333333	679.5833333	2.857747762	3.25762419	Higher in TRIM24	98	91	38	58	2133	87	121	85	1416	663
ENSMUSG00000025127.15	Gcgr	0.000236025	0.003375521	4	6	36.5	3.833333333	32.66666667	3.219792968	3.811942415	Higher in Normal	78	50	11	7	5	1	0	0	3	14
ENSMUSG00000085211.2	B430219N15Rik	0.000236224	0.003376784	4	6	90.25	6.666666667	83.58333333	3.296185871	3.949588785	Higher in Normal	253	92	12	4	7	7	3	0	16	7

ENSMUSG00000030786.18	Itgam	0.000241304	0.003433276	4	6	273.5	2029.666667	1756.166667	2.322241599	2.513924999	Higher in TRIM24	256	657	68	113	727	742	2347	2076	1486	4800
ENSMUSG00000054659.13	Pm20d2	0.000242632	0.003447349	4	6	99.75	13.83333333	85.91666667	2.560536289	2.820729416	Higher in Normal	89	22	189	99	29	6	5	1	29	13
ENSMUSG00000113010.1	Gm34084	0.000243921	0.003455979	4	6	30.25	4.666666667	25.58333333	2.724343524	3.045935477	Higher in Normal	50	35	19	17	2	0	1	2	15	8
ENSMUSG00000076587.3	Igkv6-20	0.000245176	0.003467309	4	6	52.5	3.833333333	48.66666667	3.180706068	3.758231369	Higher in Normal	3	83	47	77	8	0	10	2	1	2
ENSMUSG00000060002.14	Chpt1	0.000246274	0.003479608	4	6	16700.5	2383.333333	14317.16667	2.722556321	3.044063972	Higher in Normal	38758	19312	4283	4449	3783	363	1219	638	6472	1825
ENSMUSG00000102187.1	Gm7897	0.000247019	0.003487755	4	6	3	70.5	67.5	3.563135565	4.614322787	Higher in TRIM24	3	0	5	4	176	14	1	4	207	21
ENSMUSG00000106874.1	Gm20186	0.000247149	0.003487755	4	6	8	137.8333333	129.8333333	3.271733982	3.971840523	Higher in TRIM24	9	5	10	8	461	11	6	18	16	315
ENSMUSG00000069867.3	Pabpn1l	0.000247194	0.003487755	4	6	8.5	0.666666667	7.833333333	3.16577231	3.782287502	Higher in Normal	7	10	7	10	0	0	0	2	1	1
ENSMUSG00000061535.11	C1qtnf7	0.000247844	0.003493682	4	6	190.25	37.66666667	152.5833333	2.439906293	2.661043748	Higher in Normal	306	313	77	65	26	13	50	4	82	51
ENSMUSG00000044933.2	Sstr3	0.000248163	0.003495417	4	6	48	2.5	45.5	3.722352819	4.82282939	Higher in Normal	2	3	93	94	0	0	1	0	1	13
ENSMUSG00000047257.13	Prss45	0.000248197	0.003495417	4	6	30.75	1	29.75	3.958705146	5.527433463	Higher in Normal	89	1	32	1	0	0	1	0	0	5
ENSMUSG00000047976.4	Kcna1	0.000253593	0.003559883	4	6	99	13.33333333	85.66666667	3.039983288	3.516648449	Higher in Normal	113	47	155	81	1	1	2	0	24	52
ENSMUSG00000026354.8	Lct	0.000257083	0.003599644	4	6	53.25	9.5	43.75	2.557150055	2.816364743	Higher in Normal	87	9	66	51	6	1	1	4	18	27
ENSMUSG00000025701.12	Alox5	0.000257479	0.003602809	4	6	80.25	701	620.75	2.807958341	3.188955356	Higher in TRIM24	176	93	26	26	102	1259	1675	291	357	522
ENSMUSG00000067144.6	Slc22a7	0.000259313	0.003621819	4	6	15.25	0.5	14.75	3.921746998	5.287892752	Higher in Normal	43	4	11	3	0	0	0	0	0	3
ENSMUSG00000025911.14	Adhfe1	0.00026238	0.003660705	4	6	1508.75	163.6666667	1345.083333	3.035355128	3.51649277	Higher in Normal	3556	2285	107	87	190	49	89	39	456	159
ENSMUSG00000024503.2	Spink1	0.000262749	0.003663091	4	6	10.5	1.166666667	9.333333333	2.955310515	3.413740681	Higher in Normal	11	18	6	7	0	1	2	0	2	2
ENSMUSG00000025279.7	Dnase1l3	0.000265732	0.003701288	4	6	2337.25	346.8333333	1990.416667	2.452617144	2.680259002	Higher in Normal	368	698	3754	4529	98	82	247	486	796	372
ENSMUSG00000068617.4	Efcab1	0.000268073	0.00372877	4	6	186.5	17.83333333	168.6666667	3.150349654	3.704922692	Higher in Normal	44	62	316	324	1	0	9	2	57	38
ENSMUSG00000055254.14	Ntrk2	0.000271934	0.003773861	4	6	3595.75	491	3104.75	2.80660854	3.170922968	Higher in Normal	6122	7292	477	492	759	114	456	105	1035	477
ENSMUSG00000037405.8	Icam1	0.000272478	0.003779686	4	6	11508	1313	10195	2.687547475	3.00037317	Higher in Normal	774	927	20977	23354	765	626	1684	833	2951	1019
ENSMUSG00000106709.1	Gm30270	0.000274296	0.003799705	4	6	39.25	2.833333333	36.41666667	3.365699491	4.106762979	Higher in Normal	96	56	1	4	2	2	2	0	7	4
ENSMUSG00000022061.8	Nkx3-1	0.000275547	0.003810103	4	6	175.5	11.66666667	163.8333333	3.626200715	4.624919238	Higher in Normal	45	30	276	351	1	0	0	0	11	58
ENSMUSG00000000385.7	Tmprss2	0.000276323	0.003819106	4	6	2773.25	376.3333333	2396.916667	2.647684835	2.945417583	Higher in Normal	1623	3544	2923	3003	691	36	329	97	1033	72
ENSMUSG00000035930.9	Chst4	0.000279254	0.00384913	4	6	88.75	7.333333333	81.41666667	3.365669916	4.089704975	Higher in Normal	26	8	142	179	0	1	1	0	13	29
ENSMUSG00000033715.15	Akr1c14	0.000280172	0.003856549	4	6	565.25	87.66666667	477.5833333	2.706462602	3.028549092	Higher in Normal	1482	372	205	202	22	9	63	31	295	106
ENSMUSG00000030664.3	Sox6os	0.000280454	0.003856941	4	6	97.75	4.166666667	93.58333333	3.755780647	4.965613582	Higher in Normal	210	178	3	0	2	1	3	0	15	4
ENSMUSG00000027849.18	Syt6	0.000280609	0.003857288	4	6	16	131	115	2.407726111	2.630709431	Higher in TRIM24	39	4	12	9	56	11	113	50	205	351
ENSMUSG00000039492.7	Ccdc27	0.000281697	0.003868795	4	6	12.75	2	10.75	2.498479713	2.75038843	Higher in Normal	15	8	15	13	1	0	5	2	1	3
ENSMUSG00000072723.10	Gm10044	0.000282828	0.003879947	4	6	50.5	9.166666667	41.33333333	2.407242449	2.623742718	Higher in Normal	30	19	79	74	1	11	2	2	13	26
ENSMUSG00000085272.7	Sbk3	0.000283248	0.003883087	4	6	60.75	10	50.75	2.535904022	2.794068008	Higher in Normal	94	36	65	48	22	1	1	2	15	19
ENSMUSG00000042115.4	Klhdc8a	0.000285442	0.003907882	4	6	77.5	521.1666667	443.6666667	2.705940975	3.044382679	Higher in TRIM24	106	24	78	102	180	1008	108	1004	87	740
ENSMUSG00000076605.2	Igkj2	0.000287579	0.003935367	4	6	8.75	0.5	8.25	3.415599995	4.254606497	Higher in Normal	4	12	16	3	0	0	0	1	1	1
ENSMUSG00000030170.14	Wnt5b	0.000288787	0.003950134	4	6	4883	621	4262	2.685074743	3.000122264	Higher in Normal	1618	1239	7178	9497	779	84	245	95	2127	396
ENSMUSG00000000058.6	Cav2	0.000293345	0.004005274	4	6	8077	96876.16667	88799.16667	2.941618845	3.40786171	Higher in TRIM24	12632	9587	4782	5307	257649	3520	81586	47720	4614	186168
ENSMUSG00000027068.6	Dhrs9	0.000294266	0.004016055	4	6	106.5	1007	900.5	3.008825845	3.518930471	Higher in TRIM24	44	20	199	163	112	2979	469	102	388	1992
ENSMUSG00000050359.7	Sprr1a	0.000295233	0.004027447	4	6	79.5	997	917.5	3.016800332	3.532756524	Higher in TRIM24	93	17	99	109	229	61	532	291	4706	163
ENSMUSG00000025776.13	Crispld1	0.000298644	0.004064867	4	6	319.75	69.16666667	250.5833333	2.339458409	2.537173154	Higher in Normal	196	147	450	486	9	18	18	19	182	169
ENSMUSG00000022235.14	Cmbl	0.000298813	0.004065355	4	6	1613.75	321	1292.75	2.504035517	2.753220045	Higher in Normal	2915	2970	225	345	141	126	397	234	156	872
ENSMUSG00000028838.11	Extl1	0.00030288	0.004115171	4	6	2163.5	217.6666667	1945.833333	2.822249065	3.201347432	Higher in Normal	977	754	3368	3555	542	14	98	72	535	45
ENSMUSG00000073765.6	Gm12863	0.000305939	0.004147747	4	6	10.5	0.666666667	9.833333333	3.393337686	4.160716626	Higher in Normal	12	2	12	16	0	0	0	0	2	2
ENSMUSG00000086446.1	Prkag2os1	0.000306095	0.004147747	4	6	98.5	20.5	78	2.376977148	2.58727524	Higher in Normal	148	181	24	41	22	7	5	14	38	37
ENSMUSG00000074461.2	Gm10699	0.000306807	0.004155544	4	6	28	2.833333333	25.16666667	3.150567226	3.730852024	Higher in Normal	58	45	2	7	1	0	1	3	5	7
ENSMUSG00000022126.6	Acod1	0.000307789	0.004163721	4	6	103.75	989.6666667	885.9166667	2.835409821	3.243975593	Higher in TRIM24	37	24	132	222	957	135	925	63	3475	383
ENSMUSG00000054477.15	Kcnn2	0.000313075	0.004220073	4	6	122.5	13.83333333	108.6666667	3.173230074	3.760881769	Higher in Normal	187	48	148	107	1	4	0	0	23	55
ENSMUSG00000092528.9	Nlrp1c-ps	0.000316483	0.004259029	4	6	12	0.666666667	11.33333333	3.406711221	4.231053449	Higher in Normal	3	8	32	5	0	0	2	0	1	1
ENSMUSG00000043102.2	Qrfp	0.000317392	0.00426665	4	6	38.5	302.5	264	2.716590831	3.067369075	Higher in TRIM24	45	31	30	48	6	618	379	89	327	396
ENSMUSG00000014198.15	Zfp385c	0.000318629	0.004277603	4	6	41	6.833333333	34.16666667	2.625404621	2.922656324	Higher in Normal	107	11	31	15	1	5	7	2	4	22
ENSMUSG00000096862.1	Gm13301	0.000320434	0.004299942	4	6	17.75	0	17.75	4.163023238	6.468182036	Higher in Normal	0	7	36	28	0	0	0	0	0	0
ENSMUSG00000016255.5	Tubb1	0.000322052	0.004319753	4	6	65	9.5	55.5	2.7563322	3.109371246	Higher in Normal	171	22	59	8	4	5	1	4	19	24
ENSMUSG00000065999.13	Zfp985	0.000324045	0.004340748	4	6	70.25	11.83333333	58.41666667	2.809566414	3.185116796	Higher in Normal	165	71	26	19	3	1	3	2	24	38
ENSMUSG00000033219.8	Gm9758	0.000325667	0.004360555	4	6	12.5	0.666666667	11.83333333	3.661887271	4.704442714	Higher in Normal	26	6	11	7	0	0	0	0	0	4
ENSMUSG00000028927.6	Padi2	0.00032615	0.004363048	4	6	442.5	3425.333333	2982.833333	2.792691825	3.182985625	Higher in TRIM24	402	288	537	543	146	6936	8209	1483	733	3045
ENSMUSG00000046367.10	Mgat4e	0.000331723	0.004426078	4	6	24.25	0.833333333	23.41666667	3.850605661	5.317924928	Higher in Normal	73	1	22	1	0	0	0	0	2	3
ENSMUSG00000076526.6	Igkv12-98	0.000332786	0.004438311	4	6	23.25	2.166666667	21.08333333	3.163227428	3.757021896	Higher in Normal	28	1	30	34	2	1	0	0	1	9
ENSMUSG00000086593.2	Gm16548	0.000334677	0.00445183	4	6	68	2.5	65.5	3.686911921	4.849353005	Higher in Normal	1	6	121	144	0	0	2	0	11	2
ENSMUSG00000020713.6	Gh	0.000338943	0.004500708	4	6	20	1.166666667	18.83333333	3.43625555	4.310733049	Higher in Normal	53	3	23	1	0	0	1	2	1	3
ENSMUSG00000039462.4	Col10a1	0.000339165	0.004501699	4	6	9.25	65.5	56.25	2.415610498	2.650196084	Higher in TRIM24	16	8	1	12	92	8	32	74	47	140
ENSMUSG00000051965.8	Nanos2	0.000339705	0.004506907	4	6	22.5	0.666666667	21.83333333	3.881589084	5.41866979	Higher in Normal	59	6	25	0	0	0	0	0	2	2
ENSMUSG00000089948.1	Far2os1	0.000341558	0.004529514	4	6	4.75	71.33333333	66.58333333	3.114668968	3.730898453	Higher in TRIM24	15	2	2	0	3	21	125	96	43	140
ENSMUSG00000076961.1	Gm23251	0.000343379	0.004549699	4	6	13.5	1.5	12	2.83572853	3.251984671	Higher in Normal	31	11	7	5	1	1	3	1	2	1
ENSMUSG00000073409.12	H2-Q6	0.000348248	0.004612197	4	6	27228.5	3398.333333	23830.16667	2.610801315	2.906340428	Higher in Normal	6472	10849	41130	50463	2554	224	1027	5451	8618	2516
ENSMUSG00000026989.5	Dapl1	0.00034852	0.004613794	4	6	59.75	11.83333333	47.91666667	2.462759658	2.702715844	Higher in Normal	80	15	58	86	4	2	1	4	25	35
ENSMUSG00000046240.8	Hepacam	0.000350595	0.004635223	4	6	45.25	9.5	35.75	2.496225814	2.74623752	Higher in Normal	55	24	55	47	2	2	3	0	16	34
ENSMUSG00000065643.1	Gm25897	0.000350878	0.004636953	4	6	12.5	1.166666667	11.33333333	3.000755443	3.506499696	Higher in Normal	4	3	31	12	1	0	1	1	1	3
ENSMUSG00000029005.4	Draxin	0.000351713	0.004643959	4	6	33.75	240.5	206.75	2.523616737	2.797436224	Higher in TRIM24	40	27	39	29	8	268	475	57	346	289
ENSMUSG00000034623.14	Prss55	0.000354035	0.004664504	4	6	32	2.333333333	29.66666667	3.417234761	4.247443073	Higher in Normal	104	4	18	2	0	1	2	0	3	8
ENSMUSG00000020811.16	Wscd1	0.000355864	0.004684542	4	6	78.75	463	384.25	2.440380844	2.684085926	Higher in TRIM24	89	117	45	64	112	1137	506	290	159	574
ENSMUSG00000023267.10	Gabrr2	0.000356133	0.004686059	4	6	64	11.83333333	52.16666667	2.516024326	2.778279546	Higher in Normal	115	98	33	10	2	9	17	3	13	27
ENSMUSG00000095863.2	Ighv1-67	0.000359022	0.004713882	4	6	67.25	15.66666667	51.58333333	2.526566126	2.788959512	Higher in Normal	49	80	80	60	1	2	10	3	6	72
ENSMUSG00000104939.1	Gm33651	0.00035947	0.0047167	4	6	101.75	4.666666667	97.08333333	3.397677175	4.212868254	Higher in Normal	40	25	136	206	22	0	1	0	4	1
ENSMUSG00000035831.2	Krt25	0.000363475	0.004766185	4	6	143	10.33333333	132.6666667	3.303715048	4.021922464	Higher in Normal	60	5	221	286	2	8	0	0	22	30
ENSMUSG00000034209.4	Rasl10a	0.000366396	0.004795163	4	6	75.25	457.8333333	382.5833333	2.586823578	2.887887468	Higher in TRIM24	47	27	119	108	93	1077	765	147	105	560
ENSMUSG00000055782.8	Abcd2	0.00036763	0.004808253	4	6	6259	552	5707	3.092075861	3.644222208	Higher in Normal	13434	10997	292	313	1261	348	460	76	819	348
ENSMUSG00000036925.7	Mucl2	0.000371413	0.004843163	4	6	775.5	25.83333333	749.6666667	3.820287449	5.259529677	Higher in Normal	845	2252	5	0	49	31	3	1	8	63
ENSMUSG00000086861.1	Gm15325	0.000376892	0.004899913	4	6	10.75	1.333333333	9.416666667	2.861076791	3.285256513	Higher in Normal	21	6	9	7	2	0	1	0	1	4
ENSMUSG00000061937.7	Csn1s2a	0.00037923	0.004922769	4	6	42436.75	2833.666667	39603.08333	3.129774462	3.712180478	Higher in Normal	46400	422	50264	72661	1053	4472	2463	135	8056	823
ENSMUSG00000040314.1	Ctsg	0.000380221	0.004930568	4	6	3.75	41	37.25	2.927834243	3.419779584	Higher in TRIM24	4	4	2	5	1	45	57	9	112	22
ENSMUSG00000091897.1	Gm17019	0.000381785	0.004944277	4	6	8.25	0.333333333	7.916666667	3.614574147	4.623057197	Higher in Normal	4	8	16	5	0	0	0	0	0	2
ENSMUSG00000000386.14	Mx1	0.000382806	0.004951429	4	6	119	984	865	2.660863842	2.997584086	Higher in TRIM24	71	218	95	92	3077	241	179	317	1660	430
ENSMUSG00000086825.1	Gm15675	0.000387472	0.005001162	4	6	306.75	46	260.75	2.74315832	3.101915731	Higher in Normal	382	578	132	135	15	16	18	4	189	34
ENSMUSG00000047986.12	Palm3	0.000390465	0.005027004	4	6	58.75	11	47.75	2.40490231	2.634131271	Higher in Normal	89	93	32	21	4	1	8	19	16	18

ENSMUSG00000086706.7	Gm15848	0.000392962	0.005052861	4	6	17.25	2.5	14.75	2.817009429	3.212497587	Higher in Normal	13	30	9	17	1	0	1	0	7	6
ENSMUSG00000100744.1	Gm17764	0.000392971	0.005052861	4	6	8.25	0.833333333	7.416666667	2.904145719	3.382819586	Higher in Normal	11	6	7	9	2	0	0	1	1	1
ENSMUSG00000095285.2	Ighv5-9	0.000395832	0.005081067	4	6	21.75	2.833333333	18.91666667	2.689820596	3.035862454	Higher in Normal	10	45	10	22	1	1	6	3	5	1
ENSMUSG00000107835.1	Gm43999	0.000397216	0.005093305	4	6	21.25	2.666666667	18.58333333	3.00483076	3.51517517	Higher in Normal	13	62	6	4	1	1	1	1	5	7
ENSMUSG00000019796.12	Lrp11	0.000397287	0.005093305	4	6	114.5	694.8333333	580.3333333	2.510244914	2.784932657	Higher in TRIM24	205	135	58	60	184	1739	954	451	307	534
ENSMUSG00000038801.6	Scgb1c1	0.000398396	0.005105341	4	6	7.5	0	7.5	3.838061348	5.323191242	Higher in Normal	13	2	5	10	0	0	0	0	0	0
ENSMUSG00000010122.14	Slc47a1	0.000398561	0.005105341	4	6	88.5	18	70.5	2.356659821	2.569045173	Higher in Normal	126	19	122	87	12	1	18	2	36	39
ENSMUSG00000035186.6	Ubd	0.000399053	0.005109494	4	6	2438.5	213.6666667	2224.833333	3.093542535	3.656316524	Higher in Normal	1030	693	3144	4887	21	4	73	56	1017	111
ENSMUSG00000036095.11	Dgkb	0.00039956	0.005113825	4	6	99.5	11.83333333	87.66666667	3.050457285	3.581512789	Higher in Normal	135	71	94	98	2	0	2	0	37	30
ENSMUSG00000030041.9	M1ap	0.000401405	0.005130978	4	6	45.5	413.3333333	367.8333333	2.743045961	3.124064466	Higher in TRIM24	96	39	17	30	73	959	161	124	81	1082
ENSMUSG00000086096.1	Gm12688	0.00040753	0.005196181	4	6	12.25	0.833333333	11.41666667	3.219817584	3.941730523	Higher in Normal	6	22	6	15	0	0	3	1	1	0
ENSMUSG00000031618.13	Nr3c2	0.000407894	0.005198638	4	6	1177.5	138.1666667	1039.333333	2.694059448	3.033908856	Higher in Normal	568	539	1998	1605	375	14	72	11	280	77
ENSMUSG00000076471.3	Trbv14	0.000409212	0.005211073	4	6	14.75	0.5	14.25	3.501195561	4.64991904	Higher in Normal	1	4	27	27	0	1	1	1	0	0
ENSMUSG00000030228.18	Pik3c2g	0.000410251	0.005219939	4	6	168	29.83333333	138.1666667	2.687603129	3.02321051	Higher in Normal	211	32	236	193	4	3	8	2	57	105
ENSMUSG00000085519.1	Gm13703	0.000413262	0.005249483	4	6	15.25	1	14.25	3.429001412	4.333398696	Higher in Normal	31	23	6	1	1	0	0	0	3	2
ENSMUSG00000028699.9	Tspan1	0.000416239	0.005282892	4	6	851.25	84.83333333	766.4166667	2.81568471	3.215458464	Higher in Normal	213	586	1069	1537	252	1	80	33	98	45
ENSMUSG00000057054.11	Inca1	0.000417011	0.005290481	4	6	293.25	64.33333333	228.9166667	2.360333921	2.576695141	Higher in Normal	604	452	63	54	58	37	38	17	86	150
ENSMUSG00000026961.6	Lrrc26	0.000419424	0.005314449	4	6	142	21.66666667	120.3333333	2.409848049	2.642893381	Higher in Normal	50	172	151	195	37	8	20	16	47	2
ENSMUSG00000091255.1	Speer4e	0.000419888	0.005318113	4	6	21	2	19	3.083565158	3.648272942	Higher in Normal	8	1	32	43	1	1	1	0	1	8
ENSMUSG00000024558.12	Mapk4	0.000420346	0.005321699	4	6	219.5	34.83333333	184.6666667	2.405349224	2.636432846	Higher in Normal	236	21	330	291	4	11	76	33	36	49
ENSMUSG00000056973.6	Ces1d	0.000422435	0.005344426	4	6	11894	560.5	11333.5	3.626593183	4.757529331	Higher in Normal	24256	22910	177	233	802	48	99	6	2259	149
ENSMUSG00000045532.5	C1ql1	0.000422492	0.005344426	4	6	2.25	23.16666667	20.91666667	2.907249101	3.400846233	Higher in TRIM24	0	3	4	2	6	4	61	33	14	21
ENSMUSG00000015599.8	Ttbk1	0.000423091	0.00534921	4	6	55.5	10.16666667	45.33333333	2.49712212	2.760017341	Higher in Normal	143	13	50	16	2	4	4	9	13	29
ENSMUSG00000066583.4	Scgb1b27	0.000423749	0.005351439	4	6	121	2.666666667	118.3333333	3.979423273	6.024010778	Higher in Normal	100	384	0	0	6	1	0	0	2	7
ENSMUSG00000105419.1	Gm43205	0.000424138	0.00535413	4	6	116.25	21.33333333	94.91666667	2.38535547	2.610210115	Higher in Normal	57	118	111	179	0	8	21	14	56	29
ENSMUSG00000026303.6	Mlph	0.000424426	0.005355538	4	6	4844.5	702.6666667	4141.833333	2.492286578	2.753311085	Higher in Normal	1712	3159	7146	7361	1716	108	351	107	1523	411
ENSMUSG00000076512.2	Igkv9-123	0.000425016	0.005360764	4	6	17.5	1.166666667	16.33333333	3.278083955	4.045544877	Higher in Normal	23	34	8	5	0	0	4	2	0	1
ENSMUSG00000091575.2	2010016I18Rik	0.000425341	0.005362646	4	6	149.25	31.16666667	118.0833333	2.355855367	2.571677539	Higher in Normal	234	243	84	36	21	15	7	13	88	43
ENSMUSG00000060924.15	Csmd1	0.000426912	0.005377993	4	6	712.5	86.16666667	626.3333333	2.829394635	3.237892515	Higher in Normal	277	240	1483	850	12	5	16	32	357	95
ENSMUSG00000089829.2	Gm16565	0.000427345	0.005381227	4	6	25.5	4.5	21	2.676959073	3.00931358	Higher in Normal	48	28	15	11	1	1	2	0	9	14
ENSMUSG00000034435.6	Tmem30b	0.000430716	0.005421428	4	6	1899	224.5	1674.5	2.586609048	2.884289758	Higher in Normal	572	1017	2895	3112	701	58	94	107	341	46
ENSMUSG00000102692.1	Dchs2	0.000433742	0.005450511	4	6	440	57.66666667	382.3333333	2.502179799	2.768192459	Higher in Normal	93	29	1144	494	14	53	58	57	93	71
ENSMUSG00000027485.15	Bpifb1	0.000435833	0.005465506	4	6	107.75	8	99.75	3.068429244	3.628345051	Higher in Normal	35	12	174	210	26	2	1	0	11	8
ENSMUSG00000027919.5	Lce1g	0.000438455	0.005491602	4	6	2.5	50	47.5	3.380188914	4.33922564	Higher in TRIM24	4	2	4	0	2	47	180	1	42	28
ENSMUSG00000042501.12	Cpa6	0.000440542	0.00550868	4	6	111.75	17.66666667	94.08333333	2.754352564	3.125733746	Higher in Normal	170	48	109	120	3	2	6	0	48	47
ENSMUSG00000026069.15	Il1rl1	0.000441757	0.005521596	4	6	123.5	2556.333333	2432.833333	3.031663579	3.607153834	Higher in TRIM24	160	121	101	112	75	57	722	1708	198	12578
ENSMUSG00000097527.2	1700112J16Rik	0.000456875	0.005694206	4	6	44	6.833333333	37.16666667	2.628218055	2.948616692	Higher in Normal	92	53	25	6	4	5	3	1	19	9
ENSMUSG00000084935.1	Gm14161	0.000462216	0.00575135	4	6	14.5	0.666666667	13.83333333	3.377269611	4.283790927	Higher in Normal	6	1	22	29	1	0	0	1	2	0
ENSMUSG00000110462.1	Gm35572	0.000464409	0.005773921	4	6	93	7	86	3.604687285	4.733831944	Higher in Normal	55	306	9	2	1	2	0	0	6	33
ENSMUSG00000021208.9	Ifi27l2b	0.000468202	0.005816329	4	6	15.75	2.333333333	13.41666667	2.651144	2.988365839	Higher in Normal	34	16	4	9	1	1	1	2	5	4
ENSMUSG00000091269.2	Gm6682	0.000469333	0.005820879	4	6	32.5	267.6666667	235.1666667	2.795434979	3.218500838	Higher in TRIM24	103	6	12	9	240	362	570	191	195	48
ENSMUSG00000101067.1	Gm29007	0.000474921	0.005873429	4	6	12.5	1.833333333	10.66666667	2.719660637	3.086632883	Higher in Normal	25	11	4	10	1	1	1	0	3	5
ENSMUSG00000064140.2	Trim38	0.000476494	0.005889338	4	6	11.75	1.5	10.25	2.573895227	2.890242532	Higher in Normal	15	3	15	14	1	2	1	2	2	1
ENSMUSG00000104554.1	Gm4610	0.000478984	0.005909286	4	6	8	134.8333333	126.8333333	3.249862328	4.05172304	Higher in TRIM24	12	9	4	7	6	318	2	8	256	219
ENSMUSG00000042784.9	Muc1	0.000479517	0.005913462	4	6	2918	354.1666667	2563.833333	2.724804167	3.08987002	Higher in Normal	699	928	4535	5510	389	46	124	34	1334	198
ENSMUSG00000079497.4	Gm13420	0.000487755	0.005993205	4	6	32.25	3.666666667	28.58333333	3.116237454	3.71864078	Higher in Normal	90	9	24	6	2	0	0	1	4	15
ENSMUSG00000084939.2	Gm830	0.000488127	0.00599536	4	6	14.25	1.333333333	12.91666667	2.992782696	3.531561345	Higher in Normal	9	19	19	10	1	0	2	0	5	0
ENSMUSG00000064193.8	Gm4735	0.000489831	0.006004179	4	6	176	2934.5	2758.5	3.176300676	3.896938956	Higher in TRIM24	623	27	21	33	1320	2172	1757	4368	37	7953
ENSMUSG00000035296.13	Sgcg	0.000490224	0.00600657	4	6	550.5	46.33333333	504.1666667	3.218003132	3.908136289	Higher in Normal	1197	973	19	13	20	12	102	3	97	44
ENSMUSG00000036578.7	Fxyd7	0.000492045	0.006024045	4	6	38.75	7.666666667	31.08333333	2.343318627	2.56247418	Higher in Normal	47	68	14	26	2	1	15	8	9	11
ENSMUSG00000030406.7	Gipr	0.000499558	0.006097236	4	6	116.25	22.83333333	93.41666667	2.429981461	2.677246249	Higher in Normal	189	200	56	20	9	15	4	15	52	42
ENSMUSG00000032191.5	Bcl2l10	0.00050438	0.006142943	4	6	30	1.166666667	28.83333333	3.705839284	5.103430661	Higher in Normal	96	21	3	0	2	0	0	0	2	3
ENSMUSG00000037887.11	Dusp8	0.000507514	0.006173712	4	6	5290	668.3333333	4621.666667	2.555614233	2.850048999	Higher in Normal	374	497	9518	10771	173	607	1003	583	425	1219
ENSMUSG00000030972.6	Acsm5	0.000510681	0.00620479	4	6	141	10.66666667	130.3333333	3.430615253	4.347922694	Higher in Normal	341	205	11	7	1	1	2	0	37	23
ENSMUSG00000114147.1		0.000512098	0.006217049	4	6	13	2.166666667	10.83333333	2.427332688	2.683524509	Higher in Normal	22	12	14	4	3	2	2	1	2	3
ENSMUSG00000005360.14	Slc1a3	0.000520435	0.006300683	4	6	2258.5	332.6666667	1925.833333	2.691603855	3.046810056	Higher in Normal	3429	4127	733	745	678	38	124	39	805	312
ENSMUSG00000028197.4	Col24a1	0.000521496	0.006306	4	6	35.25	279.1666667	243.9166667	2.388689031	2.631890207	Higher in TRIM24	65	49	23	4	43	58	380	225	378	591
ENSMUSG00000095026.2	Gm3336	0.000532183	0.006422472	4	6	38.75	5.5	33.25	2.815269089	3.234703329	Higher in Normal	107	9	34	5	4	0	3	3	5	18
ENSMUSG00000079410.4	Gm2897	0.000534259	0.006434775	4	6	10	1	9	2.928074923	3.435392687	Higher in Normal	2	7	16	15	0	0	1	1	2	2
ENSMUSG00000029727.7	Cyp3a13	0.000534539	0.006435608	4	6	7	74.5	67.5	3.10059281	3.770202162	Higher in TRIM24	17	4	5	2	66	280	34	5	19	43
ENSMUSG00000022582.4	Ly6g	0.00054127	0.006506357	4	6	2.75	28.66666667	25.91666667	2.801019657	3.247366131	Higher in TRIM24	7	0	4	0	33	9	7	15	71	37
ENSMUSG00000058248.12	Kcnh1	0.000544007	0.006531516	4	6	1435.5	256.8333333	1178.666667	2.359456267	2.586164445	Higher in Normal	241	157	3062	2282	269	114	242	34	241	641
ENSMUSG00000110588.1	Gm45774	0.000544495	0.006534795	4	6	59.25	8.333333333	50.91666667	2.535279052	2.82634292	Higher in Normal	7	6	108	116	8	2	12	5	6	17
ENSMUSG00000105687.1	Gm6157	0.000544956	0.006536243	4	6	16.25	1	15.25	3.477231009	4.484163617	Higher in Normal	39	1	18	7	0	0	0	0	2	4
ENSMUSG00000001349.5	Cnn1	0.000545259	0.006536243	4	6	11591	998.1666667	10592.83333	2.957066001	3.463720466	Higher in Normal	1441	2159	17441	25323	82	117	3979	286	541	984
ENSMUSG00000059891.14	Tsks	0.000546619	0.006544807	4	6	34.75	4.333333333	30.41666667	2.934140697	3.427786677	Higher in Normal	95	12	29	3	3	1	0	2	6	14
ENSMUSG00000053522.11	Lgals7	0.000548573	0.006563936	4	6	353.25	4454.666667	4101.416667	3.00710494	3.592607538	Higher in TRIM24	125	475	321	492	6391	21	2435	2908	14437	536
ENSMUSG00000024190.6	Dusp1	0.000548851	0.006563936	4	6	16945.75	2727.833333	14217.91667	2.413681434	2.659061341	Higher in Normal	2409	1654	28759	34961	449	1550	2576	2707	2181	6904
ENSMUSG00000050052.12	Tdrp	0.000552066	0.006597061	4	6	1177.25	164.1666667	1013.083333	2.564526783	2.86764051	Higher in Normal	2231	1736	377	365	47	28	229	392	162	127
ENSMUSG00000076570.6	Igkv12-38	0.000554836	0.006624954	4	6	10	0.666666667	9.333333333	3.238417268	4.053462831	Higher in Normal	2	19	10	9	0	1	2	0	0	1
ENSMUSG00000027596.10	a	0.000559582	0.006668554	4	6	11685	1114	10571	3.082122478	3.680225327	Higher in Normal	3918	20	16851	25951	608	170	410	89	2360	3047
ENSMUSG00000028591.11	Pramef12	0.000561713	0.006689255	4	6	214.5	32.33333333	182.1666667	2.360074214	2.58893716	Higher in Normal	63	176	278	341	19	27	99	8	31	10
ENSMUSG00000004371.14	Il11	0.000561759	0.006689255	4	6	71.75	541.3333333	469.5833333	2.497667445	2.785950549	Higher in TRIM24	27	19	112	129	19	254	315	716	259	1685
ENSMUSG00000027446.2	9230104L09Rik	0.000564218	0.006710672	4	6	12.75	1.333333333	11.41666667	3.206254086	3.901970257	Higher in Normal	20	14	8	9	0	0	0	0	1	7
ENSMUSG00000028865.14	Cd164l2	0.000566813	0.006736273	4	6	569.75	63.33333333	506.4166667	2.768302735	3.168405688	Higher in Normal	368	402	641	868	141	3	26	10	189	11
ENSMUSG00000095620.7	2010005H15Rik	0.000569296	0.006755227	4	6	4	126.8333333	122.8333333	3.234439365	4.061078557	Higher in TRIM24	10	2	3	1	2	3	46	15	40	655
ENSMUSG00000093910.2	Zfp853	0.00057055	0.006767472	4	6	53.25	8.166666667	45.08333333	2.490878801	2.766161728	Higher in Normal	17	22	88	86	2	2	5	3	31	6
ENSMUSG00000034224.14	Slc38a8	0.000570852	0.006768416	4	6	30	1.333333333	28.66666667	3.634282615	4.922609464	Higher in Normal	81	0	36	3	0	0	1	0	3	4
ENSMUSG00000029223.12	Uchl1	0.000574294	0.006803934	4	6	56.5	776.8333333	720.3333333	3.14875323	3.872115423	Higher in TRIM24	86	74	40	26	54	68	2608	1686	43	202
ENSMUSG00000087648.1	E130018N17Rik	0.000575321	0.006810793	4	6	20	1.5	18.5	3.24277216	4.002456601	Higher in Normal	43	17	16	4	1	0	0	0	7	1
ENSMUSG00000108084.1	Gm7932	0.000584774	0.006906597	4	6	13.75	2.5	11.25	2.372105902	2.608045814	Higher in Normal	20	5	15	15	1	1	5	0	3	5
ENSMUSG00000020405.4	Fabp6	0.000589172	0.006942377	4	6	16.25	0.833333333	15.41666667	3.554439297	4.728003806	Higher in Normal	46	1	13	5	0	0	0	1	0	4

ENSMUSG00000022860.14	Chodl	0.000595464	0.007000261	4	6	18.25	2.5	15.75	2.60054502	2.930239087	Higher in Normal	28	10	14	21	0	0	7	2	4	2
ENSMUSG00000090208.1	Gm15851	0.000601753	0.007062815	4	6	37	2.666666667	34.33333333	3.481091807	4.516515179	Higher in Normal	46	96	2	4	1	0	0	0	7	8
ENSMUSG00000094733.1	Gm5416	0.000602267	0.007063876	4	6	2	30.5	28.5	2.908208452	3.449161327	Higher in TRIM24	3	3	2	0	35	1	5	10	41	91
ENSMUSG00000071341.4	Egr4	0.000607061	0.00710096	4	6	99	3.666666667	95.33333333	3.468725683	4.499739133	Higher in Normal	10	0	154	232	12	0	4	1	4	1
ENSMUSG00000015653.13	Steap2	0.000611958	0.007152744	4	6	688.75	4457.333333	3768.583333	2.396063891	2.649007058	Higher in TRIM24	428	408	1010	909	133	6664	4290	1984	2094	11579
ENSMUSG00000080254.4	Fam220-ps	0.000616118	0.007186557	4	6	12.5	0.5	12	3.630998133	4.817214969	Higher in Normal	4	2	22	22	0	0	0	0	0	3
ENSMUSG00000034918.8	Cdhr2	0.000616217	0.007186557	4	6	60.5	7.333333333	53.16666667	2.897340616	3.379066382	Higher in Normal	135	5	75	27	3	3	1	0	20	17
ENSMUSG00000076525.3	Igkv1-99	0.000618045	0.007201376	4	6	13	0.833333333	12.16666667	3.254756756	4.083315792	Higher in Normal	2	27	10	13	0	1	1	0	3	0
ENSMUSG00000000805.18	Car4	0.000621471	0.007233391	4	6	836.5	159	677.5	2.363702447	2.597705481	Higher in Normal	1083	1319	383	561	25	76	341	33	370	109
ENSMUSG00000030544.5	Mesp1	0.000623129	0.007240651	4	6	6.75	0.5	6.25	3.172754251	3.893677083	Higher in Normal	10	7	7	3	1	0	0	0	1	1
ENSMUSG00000073628.5	Gm10552	0.000623284	0.007240651	4	6	10.25	1.166666667	9.083333333	2.868315276	3.347044977	Higher in Normal	17	4	16	4	1	0	2	0	1	3
ENSMUSG00000082658.1	Fau-ps2	0.000630844	0.007311734	4	6	206.75	14.33333333	192.4166667	3.505377303	4.584248268	Higher in Normal	681	4	139	3	1	3	2	0	20	60
ENSMUSG00000039431.16	Mtmr7	0.000634344	0.007346716	4	6	1846	259.6666667	1586.333333	2.370389135	2.607595504	Higher in Normal	371	444	3316	3253	614	52	270	199	299	124
ENSMUSG00000066366.14	Serpina1a	0.000644477	0.007431963	4	6	24.25	2	22.25	3.002683514	3.582785595	Higher in Normal	39	12	19	27	0	4	0	0	7	1
ENSMUSG00000019577.6	Pdk4	0.000650285	0.007488605	4	6	24480	2470.166667	22009.83333	2.619885108	2.957399004	Higher in Normal	3187	2942	44778	47013	2256	870	984	6343	3077	1291
ENSMUSG00000086670.1	Gm13194	0.000656704	0.007548251	4	6	22	4	18	2.418893139	2.678933504	Higher in Normal	32	35	11	10	3	0	2	6	7	6
ENSMUSG00000061947.10	Serpina10	0.000658508	0.007563278	4	6	23.25	1.833333333	21.41666667	3.051985787	3.662028955	Higher in Normal	3	3	42	45	1	0	4	0	4	2
ENSMUSG00000032311.17	Nrg4	0.000661298	0.00758674	4	6	711.75	80.16666667	631.5833333	2.919448757	3.423508382	Higher in Normal	1607	1134	57	49	124	29	19	15	233	61
ENSMUSG00000028977.16	Casz1	0.000662285	0.00759234	4	6	2391.25	391.5	1999.75	2.464388853	2.737710479	Higher in Normal	1071	1249	3809	3436	96	99	207	64	1666	217
ENSMUSG00000004038.9	Gstm3	0.000663059	0.007598349	4	6	18	0.5	17.5	3.824210755	5.506905824	Higher in Normal	46	1	24	1	0	0	0	0	0	3
ENSMUSG00000087361.7	0610043K17Rik	0.000664228	0.007606025	4	6	66.5	12.33333333	54.16666667	2.394820756	2.64304539	Higher in Normal	82	83	54	47	25	1	3	2	28	15
ENSMUSG00000028525.16	Pde4b	0.000668322	0.007644276	4	6	12724.75	2296.333333	10428.41667	2.335537283	2.563309203	Higher in Normal	988	1254	23718	24939	1167	1108	2198	908	2719	5678
ENSMUSG00000085224.2	Gm13425	0.000677001	0.007729027	4	6	17.5	1.5	16	3.157262113	3.858994532	Higher in Normal	30	2	36	2	0	0	1	1	2	5
ENSMUSG00000050556.9	Kcnb1	0.00068361	0.007786967	4	6	797.25	150.8333333	646.4166667	2.453824448	2.72479822	Higher in Normal	1594	741	486	368	111	40	33	15	529	177
ENSMUSG00000087032.1	Gm13874	0.000684031	0.007788843	4	6	24	4.5	19.5	2.552358103	2.858749534	Higher in Normal	37	15	14	30	1	0	3	0	9	14
ENSMUSG00000079017.3	Ifi27l2a	0.000684776	0.007794419	4	6	23516.75	2120.833333	21395.91667	3.065321026	3.679258059	Higher in Normal	28818	64434	322	493	1396	725	2217	3485	2813	2089
ENSMUSG00000057802.8	Gm10030	0.000690138	0.007840788	4	6	95.75	19.16666667	76.58333333	2.441506256	2.707918597	Higher in Normal	189	74	76	44	22	5	6	0	35	47
ENSMUSG00000027500.10	Stmn2	0.000698138	0.007913961	4	6	214.5	3306	3091.5	3.154787558	3.923625407	Higher in TRIM24	217	166	191	284	22	23	3034	8895	873	6989
ENSMUSG00000110195.1	Pde2a	0.00069894	0.007918742	4	6	29	6.666666667	22.33333333	2.386053675	2.631656695	Higher in Normal	44	39	26	7	1	2	2	3	9	23
ENSMUSG00000105802.1	Gm43012	0.00069908	0.007918742	4	6	50.25	9	41.25	2.56526388	2.882146867	Higher in Normal	101	38	56	6	3	1	3	3	24	20
ENSMUSG00000031551.12	Ido1	0.000700933	0.007927939	4	6	407	48.5	358.5	2.725859082	3.121585788	Higher in Normal	74	570	462	522	104	9	31	3	133	11
ENSMUSG00000072944.11	Nup62cl	0.00070144	0.007930156	4	6	24	3.333333333	20.66666667	2.516487836	2.818845736	Higher in Normal	18	14	31	33	0	2	8	1	8	1
ENSMUSG00000053168.5	9030619P08Rik	0.000702619	0.007935214	4	6	25.75	2	23.75	3.341959359	4.243091143	Higher in Normal	69	28	3	3	0	0	0	1	5	6
ENSMUSG00000105954.1	Gm42793	0.000706588	0.007977081	4	6	4.5	60	55.5	3.034848473	3.693121555	Higher in TRIM24	14	1	2	1	37	94	3	6	159	61
ENSMUSG00000032680.11	6820408C15Rik	0.000707018	0.00797898	4	6	38	6.333333333	31.66666667	2.633196635	2.982036903	Higher in Normal	103	7	26	16	2	1	1	4	11	19
ENSMUSG00000029445.13	Hpd	0.000715537	0.008072124	4	6	63.5	3.333333333	60.16666667	3.595038593	4.887488516	Higher in Normal	180	42	32	0	0	0	0	1	8	11
ENSMUSG00000044787.7	Spata32	0.000718722	0.008099058	4	6	17.25	2.166666667	15.08333333	2.920753467	3.435810273	Higher in Normal	44	3	15	7	1	1	1	0	1	9
ENSMUSG00000030739.18	Myh14	0.000724266	0.008146642	4	6	4389.75	475.1666667	3914.583333	2.699923592	3.084542871	Higher in Normal	1547	1642	7194	7176	1252	62	306	55	1099	77
ENSMUSG00000086876.1	Gm12828	0.000724818	0.008146642	4	6	19.75	1	18.75	3.672728256	5.115116184	Higher in Normal	50	20	8	1	0	0	0	0	0	6
ENSMUSG00000028801.14	Stpg1	0.000726053	0.008157514	4	6	34.5	7	27.5	2.44511752	2.713461318	Higher in Normal	62	12	38	26	0	1	3	3	13	22
ENSMUSG00000110823.1	Gm36799	0.000727176	0.008166071	4	6	9	0.666666667	8.333333333	3.134873872	3.867815074	Higher in Normal	4	3	7	22	0	1	0	0	1	2
ENSMUSG00000087042.1	Gm11611	0.000727351	0.008166071	4	6	75.75	9	66.75	2.669578662	3.041233369	Higher in Normal	86	43	103	71	27	1	2	1	20	3
ENSMUSG00000112292.1	Gm8275	0.000729394	0.008176946	4	6	99.75	12.5	87.25	2.749619499	3.161455563	Higher in Normal	115	64	96	124	13	2	2	0	51	7
ENSMUSG00000089760.1	D030046N08Rik	0.000737107	0.008239146	4	6	11.5	0.333333333	11.16666667	3.677908362	5.090537287	Higher in Normal	36	1	8	1	0	0	0	0	1	1
ENSMUSG00000046764.8	A530053G22Rik	0.000738597	0.00824974	4	6	481	23.5	457.5	3.546982643	4.757159654	Higher in Normal	1020	898	4	2	35	2	7	0	74	23
ENSMUSG00000110157.1	Gm32507	0.000742903	0.00829176	4	6	18.5	1.166666667	17.33333333	3.317987699	4.244629385	Higher in Normal	41	30	3	0	2	1	1	0	1	2
ENSMUSG00000074417.9	Gm14548	0.000750823	0.008368136	4	6	23.75	162.6666667	138.9166667	2.438336697	2.719825584	Higher in TRIM24	63	7	22	3	64	199	230	29	244	210
ENSMUSG00000090223.1	Pcp4	0.000752731	0.008383012	4	6	17.25	1.666666667	15.58333333	2.862332031	3.366044137	Higher in Normal	4	15	14	36	0	0	1	5	1	3
ENSMUSG00000074637.7	Sox2	0.000754808	0.00840307	4	6	10.75	322.8333333	312.0833333	3.25506537	4.172328693	Higher in TRIM24	13	6	14	10	128	5	8	159	1	1636
ENSMUSG00000018566.14	Slc2a4	0.000755535	0.008405021	4	6	3572.5	381.6666667	3190.833333	2.949414282	3.489695932	Higher in Normal	7902	4768	738	882	309	141	160	12	1598	70
ENSMUSG00000102301.1	Ighv8-2	0.00076504	0.008485948	4	6	8.75	0.166666667	8.583333333	3.70170388	5.157210654	Higher in Normal	25	3	6	1	0	0	0	0	0	1
ENSMUSG00000083670.1	Gm6829	0.000770433	0.00853334	4	6	27.75	3.333333333	24.41666667	2.690682038	3.079181684	Higher in Normal	3	3	54	51	5	1	2	1	4	7
ENSMUSG00000095197.6	Ighv1-59	0.000772137	0.008545997	4	6	7.5	0.333333333	7.166666667	3.426529288	4.491519371	Higher in Normal	1	13	9	7	1	0	0	0	0	1
ENSMUSG00000043629.12	1700019D03Rik	0.000776258	0.008567638	4	6	285	37.5	247.5	2.545697234	2.862373946	Higher in Normal	214	180	316	430	127	3	15	5	52	23
ENSMUSG00000037541.21	Shank2	0.000786532	0.008651847	4	6	1803.5	225.5	1578	2.577712379	2.909220996	Higher in Normal	661	432	3644	2477	426	43	293	11	483	97
ENSMUSG00000104150.2	Gm44573	0.00079202	0.008693373	4	6	14	1.5	12.5	2.870196313	3.376980014	Higher in Normal	24	6	22	4	1	1	0	0	5	2
ENSMUSG00000083548.1	Gm11514	0.000794457	0.008716977	4	6	18.5	2.333333333	16.16666667	2.824321329	3.294036047	Higher in Normal	26	26	15	7	1	1	0	0	9	3
ENSMUSG00000027859.10	Ngf	0.000802926	0.008784579	4	6	91.75	882	790.25	2.709850648	3.132129757	Higher in TRIM24	79	67	91	130	7	161	2614	559	348	1603
ENSMUSG00000024128.13	Sbp	0.000805734	0.008808978	4	6	11.25	1	10.25	3.218316487	4.020542832	Higher in Normal	11	24	6	4	0	0	0	0	3	3
ENSMUSG00000086147.2	Zfp989	0.000812449	0.008860114	4	6	10	1.166666667	8.833333333	2.808258104	3.286270016	Higher in Normal	24	4	6	6	1	1	0	1	1	3
ENSMUSG00000024388.10	Myo7b	0.000812881	0.008861651	4	6	38.25	4	34.25	3.171172911	3.903745001	Higher in Normal	95	15	37	6	0	0	0	1	7	16
ENSMUSG00000101356.1	Gm28876	0.000814704	0.008878354	4	6	29	3	26	2.980719251	3.566853794	Higher in Normal	66	46	3	1	1	3	2	1	6	5
ENSMUSG00000035228.15	Ccdc106	0.000818944	0.008918167	4	6	28.75	5.166666667	23.58333333	2.377234875	2.629358695	Higher in Normal	10	11	46	48	0	1	6	6	3	15
ENSMUSG00000102331.1	Gm19938	0.000819723	0.008923461	4	6	61.5	10.16666667	51.33333333	2.801990472	3.25418535	Higher in Normal	117	114	10	5	2	2	3	3	18	33
ENSMUSG00000054966.13	Lmntd1	0.000822874	0.008948166	4	6	42.5	8.333333333	34.16666667	2.649006638	3.01379122	Higher in Normal	75	47	29	19	1	0	3	1	14	31
ENSMUSG00000029134.14	Plb1	0.000824379	0.008961333	4	6	1907.75	179.6666667	1728.083333	2.987434229	3.567379829	Higher in Normal	786	673	2643	3529	68	10	40	3	874	83
ENSMUSG00000111854.1	Gm48740	0.000829827	0.009005867	4	6	56.75	9.5	47.25	2.528631311	2.844621108	Higher in Normal	90	110	13	14	3	12	3	3	21	15
ENSMUSG00000037370.13	Enpp1	0.000830076	0.009005867	4	6	1477.25	6831.833333	5354.583333	2.329243267	2.572783147	Higher in TRIM24	446	417	2482	2564	3192	17125	2034	5744	4469	8427
ENSMUSG00000030217.2	Art4	0.000830251	0.009005867	4	6	243.75	25.33333333	218.4166667	2.787226193	3.23339238	Higher in Normal	119	71	333	452	70	3	8	0	46	25
ENSMUSG00000037709.13	Fam13a	0.000832305	0.009018515	4	6	2061.75	358	1703.75	2.493461846	2.791802817	Higher in Normal	3343	2891	1039	974	615	82	90	23	969	369
ENSMUSG00000085345.1	Gm16064	0.000833098	0.009020167	4	6	14.75	0.5	14.25	3.579320169	5.04009236	Higher in Normal	47	4	8	0	0	1	0	0	1	1
ENSMUSG00000092457.3	Gm8835	0.000833939	0.009020167	4	6	17	2.5	14.5	2.616342612	2.976670405	Higher in Normal	37	16	6	9	1	1	5	0	5	3
ENSMUSG00000053025.13	Sv2b	0.000838093	0.009049358	4	6	74.5	10.66666667	63.83333333	2.698406984	3.092618791	Higher in Normal	55	14	122	107	1	0	10	1	29	23
ENSMUSG00000024673.9	Ms4a1	0.000841704	0.009077536	4	6	334.5	51.5	283	2.570747996	2.904013493	Higher in Normal	114	644	267	313	9	6	186	13	41	54
ENSMUSG00000102765.1	Ighv1-62	0.000841926	0.009077536	4	6	43.75	4	39.75	2.880866564	3.393829631	Higher in Normal	26	0	76	73	4	3	5	0	7	5
ENSMUSG00000097993.7	Ptprv	0.000853871	0.009177076	4	6	83	472.8333333	389.8333333	2.412396858	2.690195458	Higher in TRIM24	135	38	98	61	1063	227	45	869	172	461
ENSMUSG00000070641.3	Gm16410	0.000859631	0.009219457	4	6	7.25	0.166666667	7.083333333	3.551072369	4.761208026	Higher in Normal	9	1	14	5	0	0	0	0	0	1
ENSMUSG00000093812.1	Gm5627	0.000863337	0.009246959	4	6	68.75	3.666666667	65.08333333	3.542318328	4.821724532	Higher in Normal	164	107	2	2	0	1	0	0	13	8
ENSMUSG00000026308.8	Klhl30	0.000869474	0.009302089	4	6	26.75	216.6666667	189.9166667	2.676908448	3.088675067	Higher in TRIM24	30	45	17	15	10	91	330	611	63	195
ENSMUSG00000030897.5	Cnga4	0.000874912	0.009356983	4	6	16.75	2.666666667	14.08333333	2.600234893	2.947190331	Higher in Normal	14	11	19	23	0	0	2	0	8	6
ENSMUSG00000100916.3	Lhb	0.000877378	0.009380053	4	6	20.75	2.666666667	18.08333333	2.51050494	2.827632961	Higher in Normal	6	8	28	41	5	2	1	1	6	1
ENSMUSG00000038453.16	Srcin1	0.000880802	0.009413356	4	6	565.5	85.5	480	2.500766141	2.805917345	Higher in Normal	824	295	688	455	250	11	10	11	114	117

ENSMUSG00000019122.8	Ccl9	0.000882159	0.009421253	4	6	951.75	7561.5	6609.75	2.343589191	2.595724969	Higher in TRIM24	951	2263	261	332	1494	4349	14558	3891	2525	18552
ENSMUSG00000076665.4	Ighv7-1	0.00088479	0.009446035	4	6	8.25	0.5	7.75	3.094367135	3.91154	Higher in Normal	4	4	9	16	0	1	1	1	0	0
ENSMUSG00000108255.1	Gm16499	0.00089206	0.009500332	4	6	25.75	2.5	23.25	2.987137346	3.591458882	Higher in Normal	32	53	9	9	6	0	1	0	7	1
ENSMUSG00000047013.15	Fbxo41	0.000901479	0.009555206	4	6	70.75	12.33333333	58.41666667	2.625109087	2.988609661	Higher in Normal	155	19	81	28	2	6	0	4	18	44
ENSMUSG00000103935.1	AY702102	0.000901549	0.009555206	4	6	7.75	0.166666667	7.583333333	3.558765062	4.91896094	Higher in Normal	14	4	12	1	0	0	0	0	1	0
ENSMUSG00000110331.1	Nudc-ps1	0.000901606	0.009555206	4	6	47.25	376	328.75	2.337888319	2.589695299	Higher in TRIM24	69	97	9	14	97	137	345	461	139	1077
ENSMUSG00000087382.7	Ctcflos	0.000902079	0.009556892	4	6	466.25	35.66666667	430.5833333	3.364641857	4.355115436	Higher in Normal	812	1012	26	15	23	4	0	1	105	81
ENSMUSG00000100672.1	Gm28404	0.000903351	0.009567039	4	6	0	13.66666667	13.66666667	3.761082899	5.702159907	Higher in TRIM24	0	0	0	0	17	1	0	2	12	50
ENSMUSG00000084853.1	Gm11791	0.000913375	0.009656548	4	6	10.25	0.5	9.75	3.411611529	4.443939674	Higher in Normal	21	1	15	4	0	0	0	0	2	1
ENSMUSG00000026650.15	Meig1	0.000913389	0.009656548	4	6	12.25	2.166666667	10.08333333	2.460972612	2.752643882	Higher in Normal	17	6	11	15	0	0	1	3	2	7
ENSMUSG00000022894.5	Adamts5	0.00091564	0.009676993	4	6	6836.5	1470	5366.5	2.407168835	2.676344528	Higher in Normal	10830	14629	954	933	906	255	998	982	2576	3103
ENSMUSG00000052188.6	Gm14964	0.000918714	0.009702739	4	6	60.75	12.16666667	48.58333333	2.644683252	3.018104662	Higher in Normal	86	108	21	28	4	0	3	2	22	42
ENSMUSG00000073414.8	Mpig6b	0.000921612	0.009723225	4	6	38.5	6.833333333	31.66666667	2.453816396	2.741779053	Higher in Normal	29	43	45	37	2	3	2	0	26	8
ENSMUSG00000028386.1	Slc46a2	0.00092503	0.009742415	4	6	117.75	21	96.75	2.786963505	3.241323091	Higher in Normal	204	131	76	60	2	3	3	0	35	83
ENSMUSG00000070504.9	Fcrl6	0.000926882	0.009754292	4	6	4.5	58.33333333	53.83333333	2.960674496	3.597114782	Higher in TRIM24	8	4	6	0	1	60	107	6	121	55
ENSMUSG00000091297.1	Gm8439	0.000927933	0.009756114	4	6	36	6.5	29.5	2.437324642	2.719092467	Higher in Normal	73	11	31	29	6	3	1	0	16	13
ENSMUSG00000096638.2	Ighv2-9	0.000930948	0.009781058	4	6	68	8.833333333	59.16666667	2.584365133	2.933011167	Higher in Normal	10	62	96	104	8	1	31	2	3	8
ENSMUSG00000111086.1	Gm48671	0.000938162	0.009846655	4	6	28	4.666666667	23.33333333	2.38174189	2.645365279	Higher in Normal	20	30	28	34	13	0	2	1	8	4
ENSMUSG00000015787.15	Abo	0.000938701	0.009848925	4	6	7.5	0.333333333	7.166666667	3.327315614	4.285824166	Higher in Normal	6	3	11	10	0	0	0	0	2	0
ENSMUSG00000107624.1	Gm44005	0.000939305	0.009850182	4	6	275.5	40.83333333	234.6666667	2.90834323	3.447288375	Higher in Normal	376	681	24	21	14	2	8	13	97	111
ENSMUSG00000085657.7	Gm15984	0.000939468	0.009850182	4	6	23	3.5	19.5	2.824224074	3.302247171	Higher in Normal	53	24	7	8	1	0	2	0	6	12
ENSMUSG00000106073.1	Gm42892	0.000957532	0.010001693	4	6	156.5	26.83333333	129.6666667	2.644093039	3.023517588	Higher in Normal	176	390	36	24	22	11	2	7	67	52
ENSMUSG00000022622.4	Acr	0.000960175	0.01002586	4	6	25.5	4.333333333	21.16666667	2.546874339	2.881858026	Higher in Normal	43	42	11	6	1	1	1	5	10	8
ENSMUSG00000051251.3	Nhlh1	0.000974546	0.010161982	4	6	28.75	2.833333333	25.91666667	3.095741531	3.801316198	Higher in Normal	87	2	24	2	2	1	0	1	2	11
ENSMUSG00000101206.1	Gm5266	0.000977111	0.010181753	4	6	18.75	1.333333333	17.41666667	3.376290403	4.41476681	Higher in Normal	51	2	19	3	0	0	0	0	2	6
ENSMUSG00000045613.9	Chrm2	0.000977675	0.010184139	4	6	16.25	2.166666667	14.08333333	2.919290806	3.460201896	Higher in Normal	12	11	25	17	0	0	0	0	3	10
ENSMUSG00000109097.1	Gm29683	0.000979611	0.010197333	4	6	25.25	3.666666667	21.58333333	2.872820543	3.390322426	Higher in Normal	61	19	15	6	1	2	0	0	3	16
ENSMUSG00000050138.7	Kcnk12	0.000986736	0.010257475	4	6	3.75	63.5	59.75	3.272720216	4.320285407	Higher in TRIM24	5	7	0	3	1	213	95	7	55	10
ENSMUSG00000023132.7	Gzma	0.000996595	0.010331742	4	6	249.5	38.33333333	211.1666667	2.533946107	2.862429094	Higher in Normal	467	267	102	162	6	4	49	32	129	10
ENSMUSG00000034923.9	Ly6g6f	0.001002935	0.010383332	4	6	13	1	12	3.202936348	4.052518093	Higher in Normal	33	3	14	2	1	0	0	1	0	4
ENSMUSG00000036570.14	Fxyd1	0.001003334	0.010383926	4	6	1738	319.3333333	1418.666667	2.418043819	2.696851663	Higher in Normal	2884	3453	246	369	128	145	630	122	638	253
ENSMUSG00000061100.3	Retnla	0.001004773	0.010388784	4	6	3753.5	328.8333333	3424.666667	3.085448997	3.781122912	Higher in Normal	3981	10789	91	153	161	31	873	199	634	75
ENSMUSG00000097326.1	A330048O09Rik	0.001006959	0.010401773	4	6	23	2.333333333	20.66666667	2.830892271	3.334282928	Higher in Normal	13	6	28	45	7	0	0	0	3	4
ENSMUSG00000054083.8	Capn12	0.001007108	0.010401773	4	6	108	22.16666667	85.83333333	2.363904891	2.621258764	Higher in Normal	156	88	101	87	23	2	2	3	62	41
ENSMUSG00000086545.4	Lrrc3c	0.001009283	0.010420699	4	6	17.75	0	17.75	3.894062056	6.603212034	Higher in Normal	48	1	22	0	0	0	0	0	0	0
ENSMUSG00000095338.1	Igkv3-9	0.001017411	0.010490393	4	6	6.75	0.166666667	6.583333333	3.541156262	4.812286985	Higher in Normal	1	13	5	8	0	0	0	0	0	1
ENSMUSG00000070495.11	Ctcfl	0.001021496	0.010523241	4	6	128.25	11.66666667	116.5833333	3.312431697	4.267550584	Higher in Normal	229	250	24	10	1	1	0	0	29	39
ENSMUSG00000025020.11	Slit1	0.001022523	0.010528843	4	6	44.75	294.8333333	250.0833333	2.590924106	2.967682749	Higher in TRIM24	125	9	29	16	74	677	471	169	282	96
ENSMUSG00000054855.13	Rnd1	0.001031532	0.010609284	4	6	2776.25	405	2371.25	2.366723426	2.627052986	Higher in Normal	372	158	4679	5896	98	361	534	331	532	574
ENSMUSG00000112489.1	9230116L04Rik	0.001032157	0.010609284	4	6	48.75	10.16666667	38.58333333	2.385519417	2.651958725	Higher in Normal	97	11	54	33	2	6	1	2	17	33
ENSMUSG00000032515.8	Csrnp1	0.00104775	0.010737758	4	6	11307.5	1359.5	9948	2.608702369	2.97710516	Higher in Normal	356	293	20437	24144	1242	542	1657	911	1551	2254
ENSMUSG00000109056.1	A630009H07Rik	0.001057608	0.010824215	4	6	10	1	9	3.071022332	3.766486112	Higher in Normal	20	5	10	5	0	0	0	0	2	4
ENSMUSG00000104077.1	Gm37027	0.001064736	0.010893509	4	6	11.75	1.833333333	9.916666667	2.582800347	2.945897592	Higher in Normal	23	12	7	5	1	0	1	1	5	3
ENSMUSG00000070933.10	Speer4d	0.001072505	0.010947246	4	6	10.75	1.166666667	9.583333333	3.015292225	3.65821282	Higher in Normal	19	4	8	12	0	0	0	0	2	5
ENSMUSG00000031936.9	Hephl1	0.001074762	0.010960273	4	6	219	31	188	2.804751715	3.288700735	Higher in Normal	549	23	175	129	9	3	5	1	94	74
ENSMUSG00000024990.12	Rbp4	0.001077788	0.010977444	4	6	4092.5	305.6666667	3786.833333	3.226252803	4.08724492	Higher in Normal	9938	6314	49	69	355	8	84	86	1131	170
ENSMUSG00000076562.1	Igkv4-50	0.001093186	0.011113633	4	6	15.25	1.333333333	13.91666667	2.959423239	3.589286622	Higher in Normal	2	21	18	20	0	0	4	2	0	2
ENSMUSG00000102319.1	Gm37626	0.001095865	0.011133437	4	6	62.5	10.83333333	51.66666667	2.748980857	3.200167778	Higher in Normal	65	166	13	6	7	3	2	1	18	34
ENSMUSG00000040016.16	Ptger3	0.001105421	0.011204344	4	6	1101.5	157.6666667	943.8333333	2.699572749	3.122414882	Higher in Normal	3126	1040	118	122	57	32	140	42	557	118
ENSMUSG00000066392.11	Nrxn3	0.001106703	0.011209878	4	6	227	42	185	2.742247049	3.189776364	Higher in Normal	473	339	72	24	15	3	11	2	72	149
ENSMUSG00000015829.13	Tnr	0.001111087	0.011239328	4	6	60	12	48	2.501492105	2.819344996	Higher in Normal	61	23	103	53	1	0	5	2	23	41
ENSMUSG00000037989.15	Wnk2	0.001141598	0.011504911	4	6	2392.25	205.1666667	2187.083333	3.012027204	3.663913684	Higher in Normal	699	671	4306	3893	40	22	44	4	1083	38
ENSMUSG00000096878.2	Gm21083	0.00114189	0.011504911	4	6	27.5	4	23.5	2.846421076	3.356349786	Higher in Normal	10	12	39	49	0	0	2	0	3	19
ENSMUSG00000040490.4	Lrfn2	0.001142184	0.011504911	4	6	9.25	1.166666667	8.083333333	2.849800903	3.383494479	Higher in Normal	13	14	5	5	0	0	1	0	3	3
ENSMUSG00000041737.8	Tmem45b	0.001142254	0.011504911	4	6	3341.5	220	3121.5	3.353265671	4.406454378	Higher in Normal	3886	9367	59	54	135	12	35	8	1077	53
ENSMUSG00000021492.15	F12	0.001156382	0.01163182	4	6	15.75	1.833333333	13.91666667	2.873740744	3.419009466	Higher in Normal	27	7	24	5	1	0	0	0	6	4
ENSMUSG00000046318.15	Ccbe1	0.001178804	0.011841713	4	6	806.75	8047.833333	7241.083333	3.078352919	3.880502372	Higher in TRIM24	290	141	1531	1265	42	28807	9146	5361	689	4242
ENSMUSG00000090118.1	Gm16163	0.001188335	0.011917796	4	6	15	1.666666667	13.33333333	2.94056176	3.548833834	Higher in Normal	35	17	6	2	1	0	1	0	5	3
ENSMUSG00000106820.1	D5Ertd605e	0.001195489	0.011965891	4	6	20.75	169.6666667	148.9166667	2.633368691	3.050764586	Higher in TRIM24	43	17	10	13	25	15	227	471	35	245
ENSMUSG00000083107.1	Gm5303	0.001196107	0.01196815	4	6	6.25	0.333333333	5.916666667	3.23554723	4.160200279	Higher in Normal	12	2	6	5	0	0	0	1	0	1
ENSMUSG00000093601.2	Gm19459	0.001203279	0.012035956	4	6	17	0.5	16.5	3.650453183	5.334993787	Higher in Normal	48	1	19	0	0	0	0	0	1	2
ENSMUSG00000001020.8	S100a4	0.001217511	0.012158343	4	6	27.25	721.8333333	694.5833333	3.05812093	3.846245049	Higher in TRIM24	22	69	3	15	9	29	176	583	55	3479
ENSMUSG00000097173.1	Gm26829	0.001218771	0.012166934	4	6	8	1	7	2.854336692	3.395236714	Higher in Normal	12	7	9	4	0	0	1	0	1	4
ENSMUSG00000006522.15	Itih3	0.001228921	0.012244172	4	6	63.25	11.83333333	51.41666667	2.39216918	2.672739535	Higher in Normal	125	7	69	52	1	8	16	1	14	31
ENSMUSG00000022026.6	Olfm4	0.001240579	0.012344167	4	6	185.25	28.83333333	156.4166667	2.433730841	2.732263613	Higher in Normal	51	10	370	310	16	11	16	5	89	36
ENSMUSG00000097974.1	Gm10605	0.001242678	0.012361008	4	6	127	32	95	2.344916396	2.605895637	Higher in Normal	192	88	99	129	4	1	12	5	55	115
ENSMUSG00000037942.5	Crp	0.001244187	0.012367945	4	6	8.25	0.833333333	7.416666667	2.951253929	3.570208168	Higher in Normal	11	2	14	6	0	0	1	0	1	3
ENSMUSG00000056706.2	Krtap7-1	0.001246145	0.012383358	4	6	62.5	0.833333333	61.66666667	3.742931081	6.266557998	Higher in Normal	0	0	102	148	0	2	0	0	0	3
ENSMUSG00000106826.1	Gm42583	0.001249191	0.012409581	4	6	63.5	10.83333333	52.66666667	2.878312445	3.431604861	Higher in Normal	90	132	19	13	2	2	0	1	11	49
ENSMUSG00000032269.8	Htr3a	0.0012518	0.012431439	4	6	38.75	3.666666667	35.08333333	3.201464656	4.070323615	Higher in Normal	114	2	36	3	1	1	0	0	3	17
ENSMUSG00000034607.6	Pof1b	0.001255357	0.012458644	4	6	747.5	80	667.5	2.813911222	3.324148089	Higher in Normal	329	518	1107	1036	76	1	34	11	349	9
ENSMUSG00000039956.8	Mrap	0.001256049	0.012461448	4	6	1530	121.1666667	1408.833333	3.162710182	3.988067996	Higher in Normal	3679	2385	28	28	136	17	25	14	498	37
ENSMUSG00000097339.5	Gm26671	0.001261731	0.012497457	4	6	37	5.833333333	31.16666667	2.595437364	2.975811414	Higher in Normal	88	46	11	3	3	1	2	6	13	10
ENSMUSG00000050808.13	Muc15	0.001267357	0.01252245	4	6	17041	1333.833333	15707.16667	3.083132335	3.823359054	Higher in Normal	14258	2502	25966	25438	216	82	218	26	7316	145
ENSMUSG00000022738.6	Gsc2	0.001271197	0.012547708	4	6	9.25	0.5	8.75	3.24393649	4.307171621	Higher in Normal	26	2	7	2	1	1	0	0	0	1
ENSMUSG00000022296.8	Baalc	0.001271337	0.012547708	4	6	4	30	26	2.357856161	2.63684593	Higher in TRIM24	0	2	5	9	11	11	23	23	7	105
ENSMUSG00000107318.1	Gm30003	0.001273615	0.012565369	4	6	21	3.833333333	17.16666667	2.560129225	2.916772368	Higher in Normal	31	21	15	17	1	0	0	1	10	11
ENSMUSG00000109750.1	Hmgb1-rs17	0.001273952	0.012565369	4	6	13.25	1.666666667	11.58333333	2.817467624	3.340125452	Higher in Normal	37	5	9	2	1	0	1	1	2	5
ENSMUSG00000002324.7	Rec8	0.001300831	0.012772561	4	6	34.5	7.166666667	27.33333333	2.466713888	2.779653348	Higher in Normal	62	20	39	17	4	0	3	0	12	24
ENSMUSG00000075425.10	Gm13547	0.001309757	0.012847773	4	6	19.5	3.833333333	15.66666667	2.382306372	2.662644805	Higher in Normal	45	7	17	9	3	0	1	3	5	11
ENSMUSG00000065592.2	Mir145a	0.001314299	0.012888175	4	6	22	3	19	2.718790822	3.176650612	Higher in Normal	29	40	6	13	1	1	2	0	12	2
ENSMUSG00000051582.2	Otud6a	0.001315842	0.012894999	4	6	28.5	3.666666667	24.83333333	2.90005718	3.479121976	Higher in Normal	77	8	26	3	3	0	1	0	5	13

ENSMUSG00000025105.9	Bnc1	0.001331213	0.013024672	4	6	92	974.5	882.5	2.792268699	3.333385402	Higher in TRIM24	11	86	134	137	160	2066	353	60	58	3150
ENSMUSG00000027270.14	Lamp5	0.001334942	0.013048574	4	6	22.25	2	20.25	3.117194493	3.899590529	Higher in Normal	7	3	28	51	0	0	0	0	4	8
ENSMUSG00000055125.7	M5C1000I18Rik	0.001337286	0.013056726	4	6	6.75	0.166666667	6.583333333	3.397758734	4.634440954	Higher in Normal	8	1	12	6	0	0	0	0	1	0
ENSMUSG00000057723.3	Krt33b	0.001338053	0.013056726	4	6	11.25	0	11.25	3.726461443	5.833405281	Higher in Normal	13	0	17	15	0	0	0	0	0	0
ENSMUSG00000108308.1	Gm45218	0.001338349	0.013056726	4	6	22.25	3.166666667	19.08333333	2.819979698	3.345917306	Higher in Normal	45	32	11	1	0	1	1	2	4	11
ENSMUSG00000026725.17	Tnn	0.001339797	0.013066665	4	6	76	819.6666667	743.6666667	2.754886324	3.267692397	Higher in TRIM24	152	14	79	59	7	91	2368	215	1140	1097
ENSMUSG00000110816.1	Gm19178	0.001340566	0.013069979	4	6	38	4	34	2.796815584	3.312382495	Higher in Normal	110	1	38	3	6	3	2	2	5	6
ENSMUSG00000039438.7	Ttc36	0.001345747	0.01310379	4	6	20	2.166666667	17.83333333	2.786375006	3.300255968	Higher in Normal	25	30	11	14	5	0	3	0	5	0
ENSMUSG00000112026.1	Gm6653	0.001345755	0.01310379	4	6	1.5	16.83333333	15.33333333	2.743736768	3.259566491	Higher in TRIM24	2	0	3	1	7	1	5	25	12	51
ENSMUSG00000032978.14	Guca2b	0.001348149	0.0131229	4	6	8	0.666666667	7.333333333	2.949942783	3.619926886	Higher in Normal	10	7	8	7	2	0	0	0	2	0
ENSMUSG00000094417.1	Gm13243	0.001348767	0.013124719	4	6	15.75	2.5	13.25	2.666685122	3.091542125	Higher in Normal	40	9	10	4	1	1	0	1	3	9
ENSMUSG00000018927.3	Ccl6	0.001352589	0.013146901	4	6	1098	9761.5	8663.5	2.430340079	2.746869433	Higher in TRIM24	1016	2940	183	253	2731	7507	19240	1976	4959	22156
ENSMUSG00000097293.1	D630002J18Rik	0.001354082	0.013155419	4	6	8.25	1.166666667	7.083333333	2.544338471	2.922419166	Higher in Normal	12	9	4	8	2	1	1	0	2	1
ENSMUSG00000073209.4	Klf14	0.001356176	0.013167365	4	6	82.75	12.83333333	69.91666667	2.865649465	3.423389642	Higher in Normal	128	187	13	3	4	1	6	1	24	41
ENSMUSG00000109052.1	Gm45012	0.001369325	0.013273861	4	6	8	0.333333333	7.666666667	3.322746619	4.472488964	Higher in Normal	18	4	9	1	1	0	0	0	1	0
ENSMUSG00000074003.4	Gucy2d	0.001374238	0.013305892	4	6	17.5	2	15.5	2.955497446	3.594860202	Higher in Normal	46	5	17	2	0	1	2	0	1	8
ENSMUSG00000046450.3	Olfr71	0.001374377	0.013305892	4	6	20.5	1.833333333	18.66666667	3.132215625	3.949783807	Higher in Normal	54	3	23	2	1	0	0	0	4	6
ENSMUSG00000024125.1	Sbpl	0.001378068	0.013328912	4	6	8.5	0.666666667	7.833333333	3.116367344	4.023249254	Higher in Normal	5	22	4	3	0	1	0	1	0	2
ENSMUSG00000086961.1	Gm12946	0.001383177	0.013369834	4	6	40.5	6.666666667	33.83333333	2.584672361	2.962128975	Higher in Normal	65	6	54	37	3	1	1	0	18	17
ENSMUSG00000021499.12	Catsper3	0.001385049	0.01337943	4	6	27.25	4.166666667	23.08333333	2.658935392	3.084237815	Higher in Normal	79	11	15	4	3	2	0	3	3	14
ENSMUSG00000097666.1	A330094K24Rik	0.001391659	0.013421993	4	6	12	0.666666667	11.33333333	3.33944961	4.477293728	Higher in Normal	25	17	6	0	0	0	1	0	2	1
ENSMUSG00000020673.14	Tpo	0.001400067	0.01349027	4	6	26.5	2.666666667	23.83333333	2.923144447	3.544050149	Higher in Normal	68	6	19	13	0	3	0	0	8	5
ENSMUSG00000080078.2	Gm15583	0.001408168	0.013538337	4	6	1.5	21.33333333	19.83333333	2.929919628	3.633542214	Higher in TRIM24	6	0	0	0	29	18	8	2	28	43
ENSMUSG00000065799.1	Gm25517	0.001415979	0.013600544	4	6	9.75	1.666666667	8.083333333	2.442543765	2.757074708	Higher in Normal	4	9	16	10	0	0	2	1	3	4
ENSMUSG00000110646.1	Olfr369-ps1	0.001421708	0.013646961	4	6	3.75	28.5	24.75	2.381103315	2.685768122	Higher in TRIM24	2	8	2	3	2	17	46	27	13	66
ENSMUSG00000084512.1	Gm22482	0.001430851	0.01372175	4	6	12	0.166666667	11.83333333	3.671307626	5.693166878	Higher in Normal	29	17	2	0	0	0	0	0	1	0
ENSMUSG00000107956.1	Speer9-ps1	0.001434018	0.013743464	4	6	5.5	0	5.5	3.507785255	4.961759003	Higher in Normal	10	5	3	4	0	0	0	0	0	0
ENSMUSG00000021478.6	Drd1	0.001436191	0.013759954	4	6	53	11.66666667	41.33333333	2.411014534	2.707197221	Higher in Normal	121	11	46	34	5	1	2	2	19	41
ENSMUSG00000086677.1	Tvp23bos	0.001437357	0.013766515	4	6	39.25	6.333333333	32.91666667	2.632607771	3.0453332	Higher in Normal	86	58	9	4	8	0	3	5	5	17
ENSMUSG00000046095.5	Krt32	0.001459097	0.013920337	4	6	17	1	16	3.394873598	4.621663811	Higher in Normal	31	0	22	15	0	0	0	0	1	5
ENSMUSG00000028214.13	Gem	0.001467903	0.013980204	4	6	8991.75	1234.5	7757.25	2.792218596	3.307815418	Higher in Normal	400	287	16444	18836	146	156	916	530	230	5429
ENSMUSG00000054362.9	Lexm	0.001469615	0.013987917	4	6	18	2.5	15.5	2.837562278	3.382243528	Higher in Normal	43	9	9	11	1	0	0	0	5	9
ENSMUSG00000069306.5	Hist1h4m	0.001482769	0.01408654	4	6	11.75	0.833333333	10.91666667	3.077039133	3.880852912	Higher in Normal	1	3	20	23	2	0	0	0	1	2
ENSMUSG00000037053.6	Azgp1	0.001494209	0.014164278	4	6	10	1.333333333	8.666666667	2.496988608	2.860863485	Higher in Normal	16	7	10	7	3	1	1	1	2	0
ENSMUSG00000032087.10	Dscaml1	0.001504087	0.01425348	4	6	75.5	13.16666667	62.33333333	2.662287411	3.092987177	Higher in Normal	221	9	53	19	3	2	4	1	24	45
ENSMUSG00000078249.5	Hmga1b	0.001508092	0.014286314	4	6	506	3556	3050	2.569778138	2.971065017	Higher in TRIM24	116	78	790	1040	1469	6917	3322	68	2449	7111
ENSMUSG00000021977.11	1700129C05Rik	0.00150849	0.014286314	4	6	16.5	1	15.5	3.410690808	4.689645805	Higher in Normal	53	2	10	1	0	0	0	0	1	5
ENSMUSG00000020890.11	Gucy2e	0.001510165	0.01429329	4	6	55.25	10.83333333	44.41666667	2.565057066	2.940507915	Higher in Normal	157	17	38	9	2	3	2	2	15	41
ENSMUSG00000097702.1	Gm26739	0.001513419	0.014310661	4	6	29.25	2.833333333	26.41666667	3.167805829	4.048849908	Higher in Normal	76	30	10	1	0	0	1	0	6	10
ENSMUSG00000113925.1	Gm7544	0.001514351	0.014310661	4	6	8.25	0.166666667	8.083333333	3.535441858	5.012351211	Higher in Normal	19	1	12	1	0	0	0	0	0	1
ENSMUSG00000056643.5	Chst13	0.00151593	0.014321141	4	6	22	200	178	2.741964583	3.26395942	Higher in TRIM24	50	18	15	5	7	47	149	675	61	261
ENSMUSG00000065564.1	Mirlet7b	0.001517118	0.01432792	4	6	47	8.333333333	38.66666667	2.497841922	2.843368297	Higher in Normal	32	131	10	15	3	3	3	7	25	9
ENSMUSG00000022101.5	Fgf17	0.001519038	0.014341605	4	6	33.25	5.333333333	27.91666667	2.457032542	2.78286304	Higher in Normal	63	9	47	14	2	0	1	8	12	9
ENSMUSG00000023467.18	Tulp2	0.001526693	0.014395537	4	6	73.25	17	56.25	2.339082471	2.609595033	Higher in Normal	140	18	69	66	8	0	5	4	27	58
ENSMUSG00000025038.7	Efhc2	0.001528566	0.014404757	4	6	23.75	5	18.75	2.458410558	2.780156845	Higher in Normal	53	21	9	12	1	0	2	2	8	17
ENSMUSG00000079594.2	BC117090	0.001534374	0.014455017	4	6	1.75	64.16666667	62.41666667	3.33166646	4.73676546	Higher in TRIM24	7	0	0	0	62	2	2	4	179	136
ENSMUSG00000052229.5	Gpr17	0.001541964	0.014501993	4	6	35.75	3.333333333	32.41666667	3.165601736	4.052346552	Higher in Normal	90	42	9	2	0	0	0	1	9	10
ENSMUSG00000050783.4	Htr1f	0.001549947	0.014556691	4	6	16.25	2	14.25	2.865844889	3.439282959	Higher in Normal	16	5	28	16	0	0	0	0	5	7
ENSMUSG00000096936.1	Gm3510	0.001556769	0.014615786	4	6	5.75	0.333333333	5.416666667	3.097463725	3.961528634	Higher in Normal	8	3	7	5	0	1	0	0	1	0
ENSMUSG00000106445.1	Gm21190	0.001558192	0.01462059	4	6	20.75	4.5	16.25	2.332531914	2.599071575	Higher in Normal	10	17	26	30	1	0	1	1	10	14
ENSMUSG00000108256.1	Gm43923	0.001567696	0.01470071	4	6	63.25	13.83333333	49.41666667	2.383131529	2.674988685	Higher in Normal	67	94	65	27	1	4	1	5	37	35
ENSMUSG00000039691.10	Tspan10	0.001569051	0.014708885	4	6	26.5	5.5	21	2.354785086	2.633338677	Higher in Normal	34	8	34	30	0	4	3	0	6	20
ENSMUSG00000027961.7	Lrrc39	0.001580121	0.014780803	4	6	309.25	58.5	250.75	2.473584657	2.808404411	Higher in Normal	754	419	40	24	41	21	25	15	146	103
ENSMUSG00000042306.11	S100a14	0.001604114	0.014986826	4	6	307.5	3766.833333	3459.333333	3.069771302	3.947635096	Higher in TRIM24	80	154	392	604	957	10156	130	108	11141	109
ENSMUSG00000035963.8	Odf3l2	0.00161285	0.015031549	4	6	12.75	0.5	12.25	3.379947919	4.699302199	Higher in Normal	38	8	5	0	1	0	1	0	1	0
ENSMUSG00000109917.1	Gm45671	0.001619808	0.015090333	4	6	17	0.833333333	16.16666667	3.42091418	4.773602362	Higher in Normal	34	4	30	0	0	0	0	0	2	3
ENSMUSG00000026943.12	Lcn12	0.001620149	0.015090333	4	6	8.75	1.333333333	7.416666667	2.51062559	2.874561018	Higher in Normal	12	5	11	7	0	0	1	1	4	2
ENSMUSG00000031170.14	Slc38a5	0.001620896	0.015092675	4	6	8.25	0.833333333	7.416666667	2.936900964	3.597113881	Higher in Normal	7	8	14	4	0	0	0	0	3	2
ENSMUSG00000098424.1	Gm27202	0.001634083	0.015182959	4	6	17	1	16	3.30669553	4.474556578	Higher in Normal	23	37	0	8	1	0	0	0	4	1
ENSMUSG00000040340.13	Tex45	0.001638075	0.015215412	4	6	23.75	2.833333333	20.91666667	2.95082111	3.611494964	Higher in Normal	56	3	33	3	0	1	1	0	3	12
ENSMUSG00000030507.6	Dbx1	0.001647613	0.015285348	4	6	11.5	0.5	11	3.319004726	4.472000153	Higher in Normal	20	2	23	1	0	0	1	0	2	0
ENSMUSG00000022157.7	Mcpt8	0.001680853	0.015521344	4	6	25.5	234.1666667	208.6666667	2.662398924	3.138477096	Higher in TRIM24	90	1	3	8	138	267	405	34	264	297
ENSMUSG00000055679.4	Ctsr	0.001681144	0.015521344	4	6	387	17.83333333	369.1666667	3.312725986	4.45903389	Higher in Normal	18	940	231	359	93	1	1	1	0	11
ENSMUSG00000059040.4	Eno1b	0.001695219	0.015636486	4	6	661.5	7171.5	6510	2.6566562	3.12866501	Higher in TRIM24	1543	915	84	104	2894	4407	3847	10355	196	21330
ENSMUSG00000075324.13	Fign	0.001715804	0.015792865	4	6	230.5	56	174.5	2.330516508	2.606639524	Higher in Normal	491	320	59	52	15	17	19	8	129	148
ENSMUSG00000097776.1	Gm26795	0.001719675	0.015818932	4	6	19.25	0.833333333	18.41666667	3.479127223	5.032871558	Higher in Normal	63	3	11	0	0	0	0	0	2	3
ENSMUSG00000093628.1	Gm20616	0.001728602	0.015886648	4	6	15.25	0.666666667	14.58333333	3.447766178	4.892879771	Higher in Normal	46	5	10	0	0	0	0	0	2	2
ENSMUSG00000089665.2	Fcor	0.001731786	0.015911106	4	6	1027.5	148.5	879	2.65916329	3.106105939	Higher in Normal	2186	1865	34	25	118	67	96	91	314	205
ENSMUSG00000025784.5	Clec3b	0.001739804	0.015957901	4	6	2118.75	334.1666667	1784.583333	2.628397468	3.056577786	Higher in Normal	2297	4970	480	728	118	38	350	30	1334	135
ENSMUSG00000076676.2	Ighv12-3	0.001748486	0.016030688	4	6	6.75	0.333333333	6.416666667	3.207514294	4.319553228	Higher in Normal	2	15	6	4	1	0	1	0	0	0
ENSMUSG00000047161.12	Chst9	0.001749427	0.016034481	4	6	183.75	14.16666667	169.5833333	3.071108378	3.875222898	Higher in Normal	49	107	251	328	6	0	1	0	73	5
ENSMUSG00000024437.6	Gm8615	0.001755315	0.016069099	4	6	12	172	160	2.904998087	3.605205713	Higher in TRIM24	41	1	4	2	242	88	276	4	10	412
ENSMUSG00000106599.1	Ighv1-73	0.001781393	0.016254079	4	6	6.75	0.5	6.25	3.054357645	3.854051816	Higher in Normal	3	3	12	9	1	0	0	0	0	2
ENSMUSG00000092270.1	Gm7031	0.001792018	0.016334444	4	6	22.75	2.333333333	20.41666667	2.982899395	3.701483759	Higher in Normal	59	8	21	3	0	1	0	0	7	6
ENSMUSG00000105387.1	Gm29681	0.00179514	0.016350116	4	6	11.75	1	10.75	3.176637461	4.116444555	Higher in Normal	29	2	12	4	0	0	0	0	1	5
ENSMUSG00000024134.11	Six2	0.00179612	0.016354147	4	6	3.5	50.16666667	46.66666667	2.968931856	3.766951144	Higher in TRIM24	1	12	1	0	191	7	21	39	27	16
ENSMUSG00000048483.6	Zdhhc22	0.001801699	0.016380448	4	6	63.75	9.5	54.25	2.760950718	3.281782992	Higher in Normal	191	22	37	5	3	3	0	2	18	31
ENSMUSG00000033579.16	Fa2h	0.001803898	0.016390652	4	6	63.25	12.16666667	51.08333333	2.340595532	2.624940911	Higher in Normal	80	45	69	59	1	10	0	4	33	25
ENSMUSG00000020656.16	Grhl1	0.001804823	0.016394164	4	6	3858	552	3306	2.443649104	2.774600482	Higher in Normal	2385	1249	5662	6136	993	73	220	73	1853	100
ENSMUSG00000110814.1	Gm38661	0.00181282	0.01645699	4	6	15.5	2.5	13	2.366411866	2.668237676	Higher in Normal	23	2	21	16	0	2	1	3	5	4
ENSMUSG00000107440.1	Gm40377	0.001820468	0.016508252	4	6	25.5	5.166666667	20.33333333	2.375305037	2.672783299	Higher in Normal	52	10	19	21	3	1	1	0	13	13
ENSMUSG00000040152.8	Thbs1	0.001828985	0.016563712	4	6	95293	12334.66667	82958.33333	2.432964178	2.7598159	Higher in Normal	3309	6810	197759	173294	6490	14360	15105	4159	21112	12782

ENSMUSG00000082245.1	Gm6292	0.001836082	0.016598877	4	6	13.5	0.833333333	12.66666667	3.21804676	4.281186007	Higher in Normal	31	5	18	0	0	0	0	1	2	2
ENSMUSG00000063021.3	Hist1h2ak	0.001845353	0.016648091	4	6	2.5	87.16666667	84.66666667	3.196129811	4.369057362	Higher in TRIM24	8	0	0	2	72	11	12	1	4	423
ENSMUSG00000106991.1	Gm43399	0.001849293	0.016670609	4	6	23	4.333333333	18.66666667	2.520112967	2.890577218	Higher in Normal	53	11	26	2	2	1	1	2	3	17
ENSMUSG00000017978.18	Cadps2	0.001849492	0.016670609	4	6	1797.75	227.5	1570.25	2.650423431	3.09934306	Higher in Normal	863	587	2997	2744	168	12	85	6	988	106
ENSMUSG00000058934.8	Igf1os	0.001855434	0.016714275	4	6	53	9	44	2.612735486	3.039570444	Higher in Normal	120	84	3	5	4	2	3	6	16	23
ENSMUSG00000078144.4	Capns2	0.001857926	0.01672277	4	6	4.5	70.83333333	66.33333333	2.671447216	3.16930059	Higher in TRIM24	3	3	6	6	22	5	6	3	28	361
ENSMUSG00000104031.1	Gm17771	0.001877757	0.016850554	4	6	8.5	1.333333333	7.166666667	2.43693091	2.780556272	Higher in Normal	14	3	10	7	2	1	0	1	1	3
ENSMUSG00000093985.7	Gm10406	0.001880098	0.016866599	4	6	27.5	3.5	24	2.512555067	2.890450541	Higher in Normal	22	28	23	37	0	3	11	2	5	0
ENSMUSG00000055413.12	H2-Q5	0.001882025	0.016878908	4	6	2268	316.3333333	1951.666667	2.452592806	2.791438285	Higher in Normal	711	752	4059	3550	33	32	102	475	1069	187
ENSMUSG00000109893.1	Gm9655	0.001908948	0.017065461	4	6	15	277.3333333	262.3333333	3.073977207	4.019461445	Higher in TRIM24	21	6	16	17	1	1	234	564	12	852
ENSMUSG00000041805.3	Pramel1	0.001913918	0.017100841	4	6	9.75	1.166666667	8.583333333	2.850550228	3.461311332	Higher in Normal	20	6	11	2	1	0	0	0	2	4
ENSMUSG00000085407.1	1700095J03Rik	0.001924731	0.017165851	4	6	1	17.33333333	16.33333333	2.9665567	3.798228441	Higher in TRIM24	3	0	0	1	0	3	17	15	24	45
ENSMUSG00000070469.12	Adamtsl3	0.001949096	0.017352654	4	6	118.75	943.5	824.75	2.479688323	2.854050318	Higher in TRIM24	152	66	142	115	24	1693	232	404	157	3151
ENSMUSG00000071531.2	Gprin2	0.001954463	0.017395353	4	6	9.25	1.333333333	7.916666667	2.516481893	2.901406395	Higher in Normal	10	3	14	10	0	1	1	0	4	2
ENSMUSG00000109674.1	Gm45470	0.001959255	0.017427808	4	6	158.5	13.33333333	145.1666667	3.118185438	4.014810322	Higher in Normal	443	181	9	1	4	2	8	0	51	15
ENSMUSG00000110086.1	Gm45623	0.001978571	0.017548364	4	6	9.5	1	8.5	2.861180099	3.49412749	Higher in Normal	16	1	13	8	0	0	2	0	1	3
ENSMUSG00000104417.1	Gm37068	0.001984029	0.017586522	4	6	10.25	1.333333333	8.916666667	2.784714225	3.335297202	Higher in Normal	10	5	18	8	0	0	0	0	3	5
ENSMUSG00000111548.1	Gm8162	0.001985661	0.01759587	4	6	32	1.666666667	30.33333333	3.379580384	4.789789668	Higher in Normal	48	79	1	0	2	0	0	0	5	3
ENSMUSG00000092222.1	Gm20506	0.002005359	0.017739442	4	6	7.75	1	6.75	2.51706937	2.924652212	Higher in Normal	7	5	5	14	0	2	1	1	1	1
ENSMUSG00000084921.1	Gm13838	0.002019063	0.017829593	4	6	6.25	0	6.25	3.478381067	5.097761783	Higher in Normal	1	8	12	4	0	0	0	0	0	0
ENSMUSG00000107634.1	Gm36816	0.00201987	0.017831543	4	6	40	3.666666667	36.33333333	3.028196292	3.827740293	Higher in Normal	80	74	4	2	6	0	1	0	11	4
ENSMUSG00000095351.2	Igkv3-2	0.002032951	0.017902227	4	6	574.5	79.5	495	2.732914117	3.251217412	Higher in Normal	35	1234	449	580	26	1	184	4	122	140
ENSMUSG00000022229.2	Atp12a	0.002041393	0.017946398	4	6	136.5	20.33333333	116.1666667	2.545483493	2.941052259	Higher in Normal	109	15	205	217	21	1	3	1	63	33
ENSMUSG00000092335.1	Zfp977	0.002064868	0.018123697	4	6	29.5	5.166666667	24.33333333	2.422467005	2.755081762	Higher in Normal	47	26	25	20	0	3	4	0	18	6
ENSMUSG00000111899.1	Gm18636	0.002067602	0.018142465	4	6	8	0.333333333	7.666666667	3.350857698	4.555918023	Higher in Normal	13	1	15	3	0	0	0	0	0	2
ENSMUSG00000038233.9	Fam198a	0.002092736	0.018304981	4	6	95.25	18	77.25	2.376386877	2.686685263	Higher in Normal	147	88	84	62	1	3	11	4	75	14
ENSMUSG00000026621.13	1-Mar	0.002099304	0.018341348	4	6	373.75	47.66666667	326.0833333	2.702026145	3.202766974	Higher in Normal	1232	210	46	7	38	21	22	13	156	36
ENSMUSG00000071322.12	Tcp10a	0.002104508	0.018376268	4	6	9.75	1	8.75	2.945336428	3.678598141	Higher in Normal	24	4	9	2	0	1	0	0	1	4
ENSMUSG00000038677.13	Scube3	0.002109391	0.018407375	4	6	1278	224.1666667	1053.833333	2.337283706	2.630799531	Higher in Normal	314	297	2427	2074	28	15	79	375	217	631
ENSMUSG00000054958.6	Nt5c1a	0.002114222	0.018435925	4	6	14.5	1.333333333	13.16666667	2.861244503	3.505999712	Higher in Normal	10	2	34	12	0	0	2	0	5	1
ENSMUSG00000079103.2	Tgm7	0.002122023	0.018476223	4	6	6.75	0.833333333	5.916666667	2.656333696	3.163611589	Higher in Normal	6	7	6	8	0	2	0	0	1	2
ENSMUSG00000020059.9	Sycp3	0.002126953	0.018513852	4	6	135.5	20	115.5	2.595919344	3.027732528	Higher in Normal	311	195	17	19	22	9	3	4	69	13
ENSMUSG00000086881.1	Gm13594	0.002133803	0.018568172	4	6	24	4.666666667	19.33333333	2.460646851	2.814336772	Higher in Normal	16	49	12	19	2	1	0	1	14	10
ENSMUSG00000096672.1	Ighv1-63	0.002150391	0.018673636	4	6	17	2.166666667	14.83333333	2.612939433	3.062943519	Higher in Normal	6	4	27	31	5	1	0	0	1	6
ENSMUSG00000085569.1	Gm12602	0.002172149	0.018799773	4	6	11	217	206	3.055567523	4.022788165	Higher in TRIM24	32	11	0	1	0	219	99	172	328	484
ENSMUSG00000086715.1	B230112J18Rik	0.002172781	0.018799894	4	6	9.5	0.833333333	8.666666667	3.004991108	3.782752939	Higher in Normal	8	1	15	14	0	0	0	0	2	3
ENSMUSG00000053353.5	2310001K24Rik	0.002184148	0.018876795	4	6	8.5	1.166666667	7.333333333	2.749525255	3.298551335	Higher in Normal	15	10	6	3	1	0	0	0	2	4
ENSMUSG00000031957.6	Ctrb1	0.002199489	0.01898782	4	6	26	2.666666667	23.33333333	2.849731718	3.489264073	Higher in Normal	79	1	22	2	0	1	3	3	3	6
ENSMUSG00000100819.1	Gm2693	0.002203089	0.019008117	4	6	10.5	1.833333333	8.666666667	2.383010223	2.712058282	Higher in Normal	10	17	7	8	2	0	2	3	0	4
ENSMUSG00000045004.3	Spata21	0.00220628	0.019023364	4	6	28.25	5.333333333	22.91666667	2.443230383	2.790605485	Higher in Normal	71	4	33	5	1	2	2	3	6	18
ENSMUSG00000091601.3	Olfr93	0.002214293	0.019077761	4	6	6.5	0.5	6	3.081677991	3.921902884	Higher in Normal	12	4	8	2	0	0	0	0	1	2
ENSMUSG00000009114.17	2610028H24Rik	0.00222438	0.019142993	4	6	33.25	5	28.25	2.744161797	3.28047873	Higher in Normal	78	6	33	16	1	1	0	0	10	18
ENSMUSG00000024747.3	Aldh1a7	0.002232307	0.019196803	4	6	1337.5	95.83333333	1241.666667	3.124226012	4.072043556	Higher in Normal	3282	1951	48	69	194	2	14	2	345	18
ENSMUSG00000066072.13	Cyp4a10	0.002244895	0.019280145	4	6	14.25	2.166666667	12.08333333	2.699853976	3.208019228	Higher in Normal	31	14	7	5	2	0	0	0	4	7
ENSMUSG00000103662.1	Gm34294	0.002256092	0.019361183	4	6	44.5	7.833333333	36.66666667	2.646696144	3.11618754	Higher in Normal	81	68	22	7	3	1	1	0	20	22
ENSMUSG00000043089.6	Mmp1a	0.002261581	0.019397355	4	6	0	24.5	24.5	3.562630314	6.727812216	Higher in TRIM24	0	0	0	0	0	30	0	1	42	74
ENSMUSG00000111840.1	Gm48832	0.002268401	0.019438513	4	6	106	11.5	94.5	2.912503	3.607628423	Higher in Normal	237	175	6	6	1	2	10	1	48	7
ENSMUSG00000031786.7	Drc7	0.002268933	0.019438513	4	6	15.75	2.666666667	13.08333333	2.353654232	2.665557667	Higher in Normal	35	1	17	10	1	2	2	2	4	5
ENSMUSG00000109728.1	Gm45359	0.002289247	0.019581965	4	6	0	4.5	4.5	3.329265965	4.676046474	Higher in TRIM24	0	0	0	0	9	1	1	7	5	4
ENSMUSG00000054618.8	Gm9951	0.002289536	0.019581965	4	6	42.75	6.833333333	35.91666667	2.51956943	2.916676939	Higher in Normal	119	45	4	3	3	7	4	3	10	14
ENSMUSG00000061517.8	Sox21	0.002292524	0.019602011	4	6	14.5	122	107.5	2.745662652	3.338233164	Higher in TRIM24	9	2	22	25	245	155	10	16	302	4
ENSMUSG00000027718.8	Il21	0.002307397	0.019723647	4	6	13	2.5	10.5	2.460803303	2.821967279	Higher in Normal	4	25	6	17	1	1	1	0	4	8
ENSMUSG00000016386.15	Mpped2	0.002328268	0.019879727	4	6	312.5	53.83333333	258.6666667	2.32949947	2.626811023	Higher in Normal	184	120	485	461	134	4	19	2	68	96
ENSMUSG00000053441.4	Adamts19	0.002336055	0.019940624	4	6	35.25	6.666666667	28.58333333	2.473197598	2.839852088	Higher in Normal	87	11	28	15	1	0	5	1	16	17
ENSMUSG00000082079.1	Dnmt3c	0.002339667	0.019960265	4	6	27	3	24	2.990113484	3.774554129	Higher in Normal	80	3	20	5	3	0	0	0	2	13
ENSMUSG00000090642.2	Gm17182	0.002349321	0.020016384	4	6	11	1.666666667	9.333333333	2.68650462	3.186363201	Higher in Normal	21	4	12	7	1	0	0	0	2	7
ENSMUSG00000058579.5	Cela2a	0.002358024	0.020060661	4	6	9	1	8	2.859728673	3.508190952	Higher in Normal	6	3	9	18	0	0	0	0	2	4
ENSMUSG00000084757.7	1700057H15Rik	0.002377022	0.020171565	4	6	22.5	1.5	21	3.196450361	4.314051519	Higher in Normal	71	0	18	1	0	1	1	0	2	5
ENSMUSG00000105482.1	Gm42596	0.002378975	0.020182509	4	6	9.25	0.833333333	8.416666667	2.982265342	3.773090639	Higher in Normal	14	3	19	1	1	0	0	0	1	3
ENSMUSG00000109277.1	Gm39121	0.002385904	0.020224386	4	6	24.75	4.166666667	20.58333333	2.658801189	3.146483838	Higher in Normal	54	36	5	4	1	1	0	2	7	14
ENSMUSG00000107036.1	Gm7596	0.002398043	0.020287748	4	6	10.5	1.666666667	8.833333333	2.631015662	3.093773463	Higher in Normal	17	4	13	8	1	0	0	0	2	7
ENSMUSG00000048142.9	Nat8l	0.002404271	0.020329146	4	6	1743	216.6666667	1526.333333	2.699963487	3.218686681	Higher in Normal	3604	3209	68	91	182	75	88	177	732	46
ENSMUSG00000073234.4	Gm8773	0.002430928	0.020503296	4	6	18.25	140	121.75	2.42240086	2.786506282	Higher in TRIM24	38	3	17	15	4	56	191	190	25	374
ENSMUSG00000029843.4	Slc13a4	0.002432922	0.020508747	4	6	97.75	17.66666667	80.08333333	2.6566835	3.144382928	Higher in Normal	273	92	24	2	4	5	3	3	26	65
ENSMUSG00000076709.2	Ighv1-47	0.002460106	0.020674737	4	6	6.75	0.666666667	6.083333333	2.790294489	3.432869168	Higher in Normal	4	3	6	14	1	1	0	0	0	2
ENSMUSG00000086460.1	Gm12236	0.002462433	0.020677377	4	6	11.5	0.166666667	11.33333333	3.516107408	5.666492458	Higher in Normal	23	22	1	0	0	0	1	0	0	0
ENSMUSG00000043122.7	A530016L24Rik	0.00246352	0.0206808	4	6	1722.25	128.3333333	1593.916667	3.13878253	4.146219757	Higher in Normal	4145	2692	22	30	102	7	18	2	599	42
ENSMUSG00000110495.1	Gm45879	0.002467644	0.020704001	4	6	0	3.5	3.5	3.148223123	4.188079001	Higher in TRIM24	0	0	0	0	2	3	4	3	3	6
ENSMUSG00000040919.13	4930505A04Rik	0.002473748	0.020735276	4	6	10.25	1.833333333	8.416666667	2.530457444	2.937758839	Higher in Normal	19	12	6	4	1	0	1	0	3	6
ENSMUSG00000076532.3	Igkv4-91	0.002473765	0.020735276	4	6	123.5	13.83333333	109.6666667	2.604865419	3.062223335	Higher in Normal	15	164	148	167	13	8	55	2	1	4
ENSMUSG00000046750.17	Selenov	0.002491107	0.020856423	4	6	14.75	2.666666667	12.08333333	2.496909713	2.88463019	Higher in Normal	31	10	11	7	1	1	0	0	6	8
ENSMUSG00000101387.1	Pced1c-ps	0.00249129	0.020856423	4	6	13	0.5	12.5	3.369202484	4.827892224	Higher in Normal	32	2	18	0	0	0	0	0	2	1
ENSMUSG00000103748.1	Gm38243	0.002501141	0.020904405	4	6	8.25	0.666666667	7.583333333	3.108068738	4.031424706	Higher in Normal	20	3	8	2	0	0	0	0	1	3
ENSMUSG00000100261.1	Gm6473	0.00250931	0.020945779	4	6	10.5	0.666666667	9.833333333	3.021832635	3.97353287	Higher in Normal	24	1	16	1	0	1	1	1	0	1
ENSMUSG00000044322.6	Dsc1	0.002509509	0.020945779	4	6	26.5	3	23.5	2.908181053	3.623817028	Higher in Normal	74	2	16	14	0	2	0	0	5	11
ENSMUSG00000114051.1	Gm48008	0.002516675	0.020975686	4	6	16.75	2.5	14.25	2.606156295	3.06328904	Higher in Normal	31	4	17	15	2	0	0	0	7	6
ENSMUSG00000095589.2	Ighv1-55	0.002522068	0.021004359	4	6	14	1.166666667	12.83333333	3.087574378	4.047295469	Higher in Normal	0	30	8	18	0	0	1	0	3	3
ENSMUSG00000095771.2	Igkv14-111	0.002540483	0.021105768	4	6	289.25	45.5	243.75	2.496283118	2.887332786	Higher in Normal	71	626	196	264	38	4	156	2	24	49
ENSMUSG00000114178.1	Gm48656	0.00254418	0.021130709	4	6	11	0.5	10.5	3.315110111	4.639794448	Higher in Normal	28	6	10	0	0	0	0	0	2	1
ENSMUSG00000028176.11	Lrrc7	0.002559153	0.021226125	4	6	91	16.83333333	74.16666667	2.58692936	3.031877032	Higher in Normal	163	11	109	81	1	1	1	3	31	64
ENSMUSG00000114149.1	Gm47646	0.002593689	0.021413404	4	6	9.75	0.166666667	9.583333333	3.481792242	5.281607314	Higher in Normal	25	2	12	0	0	0	0	0	0	1
ENSMUSG00000050440.8	Hamp	0.002645386	0.021734135	4	6	12.25	1.666666667	10.58333333	2.489062537	2.891146703	Higher in Normal	14	0	20	15	2	1	1	1	2	3

ENSMUSG00000111400.1	Gm29961	0.002655026	0.021795691	4	6	13.75	1.333333333	12.41666667	3.017588312	3.883565493	Higher in Normal	37	9	7	2	0	0	0	0	4	4
ENSMUSG00000042041.6	2010003K11Rik	0.002678616	0.021942024	4	6	156.25	14.5	141.75	2.874329746	3.569237525	Higher in Normal	487	112	12	14	18	1	1	14	51	2
ENSMUSG00000101693.1	Gm19461	0.002691361	0.02200498	4	6	26	4.5	21.5	2.478240142	2.86315666	Higher in Normal	48	5	39	12	3	0	0	2	10	12
ENSMUSG00000063529.3	Stmnd1	0.002693676	0.022018003	4	6	9.5	1	8.5	2.913214182	3.668307823	Higher in Normal	14	15	3	6	0	0	0	0	4	2
ENSMUSG00000063887.13	Nlgn1	0.0027244	0.022233324	4	6	181.75	35.5	146.25	2.509441529	2.914250952	Higher in Normal	178	50	252	247	8	1	14	0	61	129
ENSMUSG00000027513.11	Pck1	0.002729353	0.022261811	4	6	12395.25	1068.833333	11326.41667	3.005525813	3.855552095	Higher in Normal	19277	29347	424	533	1837	192	249	9	3937	189
ENSMUSG00000047155.13	Cyp4x1	0.002745453	0.022375149	4	6	31.75	3.5	28.25	3.050850882	3.960669127	Higher in Normal	96	24	2	5	1	0	0	0	5	15
ENSMUSG00000080142.2	Gm12838	0.002756494	0.022447109	4	6	27	3.833333333	23.16666667	2.665228463	3.181261856	Higher in Normal	88	3	16	1	3	1	3	1	4	11
ENSMUSG00000020953.17	Coch	0.00275876	0.022459554	4	6	26.25	5	21.25	2.344604746	2.659935823	Higher in Normal	28	7	44	26	0	0	6	1	12	11
ENSMUSG00000057729.12	Prtn3	0.002773133	0.022546423	4	6	91.25	1504.333333	1413.083333	3.026029828	4.037694375	Higher in TRIM24	169	115	39	42	6632	23	23	77	2176	95
ENSMUSG00000106958.1	Gm35960	0.00278997	0.022659113	4	6	0.25	6.666666667	6.416666667	3.158545395	4.442251124	Higher in TRIM24	1	0	0	0	15	1	2	3	16	3
ENSMUSG00000085320.1	Gm11548	0.002793083	0.022678347	4	6	15.75	1.166666667	14.58333333	3.196340581	4.379782614	Higher in Normal	46	2	14	1	1	0	0	0	0	6
ENSMUSG00000107078.1	Gm36840	0.002797546	0.022702474	4	6	12.75	0.666666667	12.08333333	3.149063136	4.254809911	Higher in Normal	7	2	27	15	0	0	0	0	4	0
ENSMUSG00000096257.2	Ccer2	0.002810708	0.022797135	4	6	13	0.833333333	12.16666667	3.196509174	4.403568173	Higher in Normal	32	1	18	1	0	0	0	1	0	4
ENSMUSG00000025936.15	Gm4956	0.002834524	0.022965826	4	6	10.25	1	9.25	2.883354998	3.619031173	Higher in Normal	22	2	15	2	0	0	1	0	3	2
ENSMUSG00000085079.3	Khdc1b	0.002852979	0.023078507	4	6	11.5	0.666666667	10.83333333	3.304774168	4.638808262	Higher in Normal	29	3	13	1	0	0	0	0	0	4
ENSMUSG00000003341.9	Atp8b3	0.002855685	0.023088133	4	6	97	16	81	2.510947148	2.924679028	Higher in Normal	294	6	78	10	5	8	3	18	8	54
ENSMUSG00000057913.2	Gm10032	0.002899197	0.023384043	4	6	436.25	39.83333333	396.4166667	2.858400597	3.552933388	Higher in Normal	199	19	710	817	30	4	3	0	175	27
ENSMUSG00000051431.7	Gpr87	0.00290163	0.023385083	4	6	32.5	442.1666667	409.6666667	3.071169589	4.194359906	Higher in TRIM24	18	10	48	54	30	1510	8	2	1081	22
ENSMUSG00000087162.1	Gm14244	0.002909131	0.023433125	4	6	7.75	1	6.75	2.68862666	3.246018302	Higher in Normal	8	8	13	2	0	1	0	0	2	3
ENSMUSG00000046317.14	BC107364	0.002948324	0.023679915	4	6	56	7.5	48.5	2.921393962	3.688347548	Higher in Normal	69	125	17	13	0	2	0	0	16	27
ENSMUSG00000084713.2	Gm24627	0.002952694	0.023708764	4	6	0	14.33333333	14.33333333	3.471563829	6.289750795	Higher in TRIM24	0	0	0	0	0	0	30	32	2	22
ENSMUSG00000000381.10	Wap	0.002978868	0.023843474	4	6	2287	112.3333333	2174.666667	3.043533192	3.977307783	Higher in Normal	183	2	3412	5551	10	263	81	3	294	23
ENSMUSG00000106478.1	Gm36551	0.002985713	0.023879429	4	6	4	34.83333333	30.83333333	2.597950286	3.114604711	Higher in TRIM24	11	1	3	1	124	5	8	8	32	32
ENSMUSG00000034427.17	Myo15b	0.003010638	0.024034584	4	6	215	34	181	2.744261381	3.33623984	Higher in Normal	629	31	183	17	8	7	1	1	56	131
ENSMUSG00000054200.6	Ffar4	0.00301729	0.024075066	4	6	315	54.33333333	260.6666667	2.358842661	2.692135255	Higher in Normal	558	627	35	40	68	8	64	62	97	27
ENSMUSG00000076672.7	Ighv3-6	0.003021381	0.02410139	4	6	71.75	12.33333333	59.41666667	2.45004875	2.832636203	Higher in Normal	2	89	103	93	8	3	23	0	9	31
ENSMUSG00000032451.6	Trim42	0.003041739	0.024225703	4	6	11.75	1.333333333	10.41666667	2.726321343	3.326066035	Higher in Normal	33	4	8	2	2	0	0	1	3	2
ENSMUSG00000098401.1	Mir6377	0.003061936	0.024322938	4	6	9.25	0.333333333	8.916666667	3.366441117	4.974354455	Higher in Normal	12	24	0	1	0	0	0	0	1	1
ENSMUSG00000086413.1	Gm12415	0.003082494	0.024441611	4	6	0	29.66666667	29.66666667	3.468094583	6.849672759	Higher in TRIM24	0	0	0	0	0	0	11	42	1	124
ENSMUSG00000099320.1	Mir7664	0.003084909	0.024448022	4	6	6.75	0.5	6.25	2.935788991	3.729075908	Higher in Normal	3	1	15	8	0	0	1	0	1	1
ENSMUSG00000027517.13	Ankrd60	0.003087764	0.024464286	4	6	30.75	3.833333333	26.91666667	2.894280654	3.639160101	Higher in Normal	87	6	29	1	1	0	1	0	6	15
ENSMUSG00000104951.1	Gm43413	0.003089776	0.024467493	4	6	97.5	20.66666667	76.83333333	2.391598168	2.743444186	Higher in Normal	237	105	29	19	16	3	1	5	47	52
ENSMUSG00000026387.15	Sctr	0.003094621	0.024480952	4	6	138.5	25.16666667	113.3333333	2.570642648	3.032250498	Higher in Normal	315	216	18	5	12	8	6	2	59	64
ENSMUSG00000037738.11	Nek5	0.003107488	0.024556655	4	6	81.25	18.83333333	62.41666667	2.398464997	2.752818974	Higher in Normal	142	56	61	66	5	0	1	4	33	70
ENSMUSG00000042414.7	Prdm14	0.003111433	0.024575074	4	6	33.75	5	28.75	2.629592555	3.134342517	Higher in Normal	90	2	32	11	3	1	0	1	12	13
ENSMUSG00000066364.2	Serpina3b	0.003115845	0.024603538	4	6	18.75	3.166666667	15.58333333	2.509655025	2.928545384	Higher in Normal	17	2	30	26	1	2	0	0	3	13
ENSMUSG00000097636.7	Mirt1	0.003123761	0.024653257	4	6	130.25	734.6666667	604.4166667	2.373813788	2.735164876	Higher in TRIM24	213	86	135	87	158	2342	148	72	1014	674
ENSMUSG00000090785.1	Gm17116	0.003141503	0.024774015	4	6	9	1	8	2.859568045	3.600828646	Higher in Normal	11	20	3	2	1	0	0	0	3	2
ENSMUSG00000094634.8	Gm3468	0.003148989	0.024807355	4	6	45.25	3.166666667	42.08333333	2.860977048	3.593304671	Higher in Normal	4	26	73	78	0	0	9	7	3	0
ENSMUSG00000021803.9	Cdhr1	0.003152649	0.024817161	4	6	393.75	47.16666667	346.5833333	2.59525361	3.07708973	Higher in Normal	167	69	656	683	97	1	5	3	138	39
ENSMUSG00000049103.14	Ccr2	0.003163807	0.024891884	4	6	723.75	4900.166667	4176.416667	2.333179099	2.672706122	Higher in TRIM24	1011	1685	123	76	891	7191	7785	4964	2609	5961
ENSMUSG00000057050.7	Olfr397	0.003172815	0.02494986	4	6	8	0.5	7.5	3.162626386	4.256873865	Higher in Normal	23	2	6	1	0	0	0	0	1	2
ENSMUSG00000020010.7	Vnn3	0.003176937	0.024962936	4	6	337	24.83333333	312.1666667	3.044810106	4.006762763	Higher in Normal	296	991	28	33	0	0	74	28	39	8
ENSMUSG00000054061.8	Gm9934	0.003189824	0.025038347	4	6	6.75	0.166666667	6.583333333	3.257785801	4.721992451	Higher in Normal	18	1	6	2	0	0	1	0	0	0
ENSMUSG00000082046.1	Gm14464	0.003192434	0.025045927	4	6	34.25	6	28.25	2.645373591	3.164180515	Higher in Normal	81	31	24	1	2	2	1	0	7	24
ENSMUSG00000084390.1	Gm15425	0.003196734	0.02506029	4	6	14.5	1.166666667	13.33333333	3.003492069	3.941206517	Higher in Normal	45	0	7	6	0	1	0	1	1	4
ENSMUSG00000085673.1	Gm16122	0.003204992	0.025105631	4	6	6.25	0.333333333	5.916666667	3.108737167	4.136394706	Higher in Normal	11	1	11	2	0	0	0	0	1	1
ENSMUSG00000094797.1	Igkv6-15	0.003221883	0.025224963	4	6	378	98.33333333	279.6666667	2.509568793	2.93523846	Higher in Normal	114	874	216	308	9	15	38	4	14	510
ENSMUSG00000107622.1	4930512J16Rik	0.003243837	0.025376494	4	6	15.75	2.333333333	13.41666667	2.574667665	3.053631313	Higher in Normal	46	6	9	2	0	2	1	1	4	6
ENSMUSG00000105935.1	Gm43628	0.003271574	0.025548315	4	6	16	3	13	2.362197294	2.706535537	Higher in Normal	31	14	13	6	4	1	0	0	7	6
ENSMUSG00000008028.15	1700008O03Rik	0.003274094	0.025561437	4	6	12.5	2.166666667	10.33333333	2.408038045	2.779157481	Higher in Normal	28	3	15	4	2	1	0	1	3	6
ENSMUSG00000098199.1	Gm9358	0.003292285	0.025668788	4	6	8.75	0.666666667	8.083333333	3.036242823	4.043110667	Higher in Normal	18	9	8	0	0	0	0	1	1	2
ENSMUSG00000085851.1	4921518K17Rik	0.00330961	0.025766043	4	6	8	1	7	2.725192096	3.332690159	Higher in Normal	8	10	9	5	0	0	0	0	4	2
ENSMUSG00000081677.1	Mdk-ps1	0.003323063	0.025849544	4	6	13.5	0.666666667	12.83333333	3.186771607	4.520054485	Higher in Normal	37	0	16	1	1	0	0	1	0	2
ENSMUSG00000094124.5	Ighv1-74	0.003344009	0.025974079	4	6	11.5	2.166666667	9.333333333	2.448113379	2.838466733	Higher in Normal	3	17	16	10	1	0	3	0	1	8
ENSMUSG00000041923.15	Nol4	0.003352563	0.026020608	4	6	5.5	72.33333333	66.83333333	3.032580713	4.159705079	Higher in TRIM24	13	0	5	4	0	267	81	39	5	42
ENSMUSG00000086587.7	Gm11837	0.003359775	0.026056657	4	6	27.25	3.666666667	23.58333333	2.626581735	3.151009511	Higher in Normal	46	51	4	8	0	0	4	8	3	7
ENSMUSG00000104966.1	Gm43273	0.003364992	0.026090468	4	6	7.25	0.5	6.75	3.012674691	3.909426775	Higher in Normal	10	1	14	4	0	0	0	0	2	1
ENSMUSG00000080747.1	Gm14016	0.003384861	0.026230902	4	6	10.25	1.833333333	8.416666667	2.537195466	2.990513643	Higher in Normal	18	16	5	2	1	0	1	1	1	7
ENSMUSG00000023999.14	Kif6	0.003469464	0.026784541	4	6	54.5	8.833333333	45.66666667	2.705237441	3.284373982	Higher in Normal	42	17	102	57	0	0	2	0	12	39
ENSMUSG00000099349.6	1700047G03Rik	0.003477068	0.026836439	4	6	126.25	11.33333333	114.9166667	3.155872832	4.364570855	Higher in Normal	320	164	20	1	2	0	0	0	20	46
ENSMUSG00000062124.7	Defb45	0.003538294	0.02723306	4	6	7.5	0.333333333	7.166666667	3.178597846	4.382412705	Higher in Normal	14	0	8	8	0	0	0	0	1	1
ENSMUSG00000035638.14	Muc20	0.00354688	0.027278453	4	6	2214	197.6666667	2016.333333	2.74770591	3.377058603	Higher in Normal	462	347	3892	4155	489	7	29	11	632	18
ENSMUSG00000051079.8	Rgs13	0.003548216	0.027281464	4	6	23.25	3.333333333	19.91666667	2.85422903	3.577909402	Higher in Normal	20	10	31	32	0	0	0	0	1	19
ENSMUSG00000023978.5	Prph2	0.003553753	0.027293462	4	6	19.5	1.166666667	18.33333333	3.153450838	4.428038765	Higher in Normal	57	1	19	1	0	2	0	0	0	5
ENSMUSG00000092408.1	Vhl-ps1	0.003554103	0.027293462	4	6	20.75	2.833333333	17.91666667	2.64964116	3.193562792	Higher in Normal	39	13	12	19	0	0	0	1	12	4
ENSMUSG00000049548.8	Krt82	0.003577533	0.02744488	4	6	42.5	8.833333333	33.66666667	2.486018981	2.905933245	Higher in Normal	106	5	31	28	2	1	3	0	10	37
ENSMUSG00000031204.3	Asb12	0.003598522	0.027564226	4	6	43	5.166666667	37.83333333	2.715876691	3.32394721	Higher in Normal	116	52	4	0	2	2	9	2	10	6
ENSMUSG00000027556.15	Car1	0.003606573	0.027612003	4	6	22.5	2.166666667	20.33333333	3.008501	3.970968394	Higher in Normal	43	44	2	1	1	0	0	0	7	5
ENSMUSG00000041044.9	Lrit1	0.003622634	0.027688851	4	6	20	2	18	2.992478627	3.919626182	Higher in Normal	52	1	19	8	0	0	0	0	4	8
ENSMUSG00000039518.7	Cdsn	0.003666453	0.027881158	4	6	979	124	855	2.786775444	3.462591182	Higher in Normal	2073	1785	51	7	21	10	179	12	357	165
ENSMUSG00000043441.5	Gpr149	0.003690803	0.028045314	4	6	7.5	402.6666667	395.1666667	3.177135469	4.80304954	Higher in TRIM24	26	0	2	2	6	1	266	20	2	2121
ENSMUSG00000083157.1	Gm13511	0.003724215	0.028242818	4	6	5	0.166666667	4.833333333	3.077354907	4.212945134	Higher in Normal	4	2	10	4	0	0	1	0	0	0
ENSMUSG00000028287.4	1700009N14Rik	0.0037398	0.028297569	4	6	0	5.666666667	5.666666667	3.296856901	4.938542597	Higher in TRIM24	0	0	0	0	0	3	14	3	12	2
ENSMUSG00000022602.14	Arc	0.003749237	0.028335469	4	6	10400.25	1056.333333	9343.916667	2.642352162	3.188542283	Higher in Normal	79	70	19209	22243	1565	343	578	538	2149	1165
ENSMUSG00000024292.14	Cyp4f14	0.003757719	0.028362676	4	6	15.75	3	12.75	2.365888265	2.730889223	Higher in Normal	32	25	3	3	1	3	1	2	3	8
ENSMUSG00000050663.7	Trhde	0.003772676	0.028468507	4	6	97.5	14.16666667	83.33333333	2.840466021	3.580613883	Higher in Normal	256	121	10	3	2	1	3	0	27	52
ENSMUSG00000104429.1	2610034O05Rik	0.003781608	0.028521771	4	6	14.5	0.666666667	13.83333333	3.251316104	4.762225615	Higher in Normal	36	1	21	0	0	0	0	0	2	2
ENSMUSG00000109603.1	Gm32389	0.00379564	0.028599268	4	6	52.25	7.666666667	44.58333333	2.671106831	3.246537148	Higher in Normal	124	77	6	2	6	5	1	0	14	20
ENSMUSG00000091401.1	Gm9252	0.003808608	0.028682782	4	6	24.5	2.666666667	21.83333333	2.831235971	3.571923003	Higher in Normal	73	3	22	0	1	1	2	0	5	7
ENSMUSG00000105712.1	Gm5712	0.003843785	0.028869159	4	6	15.25	1.333333333	13.91666667	3.035119578	4.075999152	Higher in Normal	49	1	10	1	0	0	0	1	1	6

ENSMUSG00000113241.1	Gm40663	0.0038627	0.028961215	4	6	9.75	1.5	8.25	2.499116174	2.949050811	Higher in Normal	21	2	6	10	1	1	0	0	3	4
ENSMUSG00000043168.12	4930426D05Rik	0.003871019	0.029002162	4	6	10.25	1.833333333	8.416666667	2.39391464	2.778903031	Higher in Normal	8	20	9	4	0	1	3	0	4	3
ENSMUSG00000200052.9	Ascl1	0.003878588	0.029051722	4	6	14	2.833333333	11.16666667	2.382052184	2.753209634	Higher in Normal	24	22	5	5	1	1	1	0	7	7
ENSMUSG00000045246.11	Kcng4	0.003905569	0.029178035	4	6	14.5	2.666666667	11.83333333	2.379782985	2.750510961	Higher in Normal	32	9	12	5	0	0	3	1	7	5
ENSMUSG00000106524.1	Gm42706	0.00392018	0.029255307	4	6	36.75	4.666666667	32.08333333	2.469056217	2.898503032	Higher in Normal	13	5	77	52	8	4	0	0	11	5
ENSMUSG00000105362.1	Gm43474	0.003952184	0.029447472	4	6	12	0.833333333	11.16666667	2.907658539	3.808371164	Higher in Normal	32	1	12	3	2	0	0	1	2	0
ENSMUSG00000037129.7	Tmprss13	0.003960861	0.029493837	4	6	1359	169.6666667	1189.333333	2.476783847	2.908478451	Higher in Normal	666	559	1832	2379	511	11	49	7	398	42
ENSMUSG00000105350.1	4930537H20Rik	0.003963395	0.029505105	4	6	11.25	1.166666667	10.08333333	2.694185138	3.328432464	Higher in Normal	26	5	10	4	0	0	0	3	3	1
ENSMUSG00000085177.1	Gm11209	0.003979633	0.029575754	4	6	10.25	1.833333333	8.416666667	2.402266012	2.795278232	Higher in Normal	17	17	5	2	1	0	2	1	4	3
ENSMUSG00000024401.14	Tnf	0.003990719	0.029643676	4	6	1961.25	276.1666667	1685.083333	2.363439705	2.726425683	Higher in Normal	65	174	3428	4178	92	95	510	103	713	144
ENSMUSG00000082199.1	Gm15763	0.004010196	0.02975931	4	6	5.5	0.5	5	2.806279561	3.525017905	Higher in Normal	4	4	12	2	0	0	1	0	1	1
ENSMUSG00000112662.1	Gm47922	0.004026452	0.029858109	4	6	11.75	1.5	10.25	2.786569084	3.488462981	Higher in Normal	27	4	14	2	0	1	0	0	1	7
ENSMUSG00000108175.1	Gm16499	0.004034927	0.029913676	4	6	7.25	0.833333333	6.416666667	2.717846378	3.377392665	Higher in Normal	13	1	8	7	0	1	0	0	1	3
ENSMUSG00000039552.14	Rsph4a	0.004075646	0.030142161	4	6	14	2.166666667	11.83333333	2.634826693	3.185418537	Higher in Normal	35	3	12	6	0	0	2	0	3	8
ENSMUSG00000095298.2	Gm12407	0.004077736	0.030150294	4	6	21.25	2.666666667	18.58333333	2.457579821	2.888942263	Higher in Normal	1	10	27	47	0	1	6	3	4	2
ENSMUSG00000034185.9	6430628N08Rik	0.00408031	0.030161998	4	6	7.75	0.666666667	7.083333333	2.890687703	3.780500318	Higher in Normal	20	1	8	2	0	1	0	0	1	2
ENSMUSG00000032401.15	Lctl	0.004106247	0.030286723	4	6	555.25	60.83333333	494.4166667	2.81256635	3.546091187	Higher in Normal	1698	497	19	7	65	2	8	15	219	56
ENSMUSG00000085336.1	Gm11732	0.004123187	0.030346336	4	6	11.5	1.5	10	2.626732637	3.19576143	Higher in Normal	15	19	4	8	5	0	0	0	1	3
ENSMUSG00000031344.11	Gabrq	0.004126736	0.03036512	4	6	35.75	7	28.75	2.439526231	2.849846231	Higher in Normal	96	14	25	8	1	1	1	1	19	19
ENSMUSG00000027403.12	Tgm6	0.004138889	0.030417786	4	6	16.75	2.166666667	14.58333333	2.847854522	3.619026689	Higher in Normal	32	5	21	9	0	1	0	0	0	12
ENSMUSG00000035592.2	Krt33a	0.004144031	0.030440879	4	6	45	1.833333333	43.16666667	3.231184644	4.814695249	Higher in Normal	2	0	55	123	0	0	0	0	8	3
ENSMUSG00000035948.13	Acss3	0.004153498	0.030488358	4	6	368.5	65.33333333	303.1666667	2.464812569	2.894871508	Higher in Normal	792	486	111	85	49	23	22	0	202	96
ENSMUSG00000081880.1	Gm5385	0.004166338	0.030538437	4	6	41	5.5	35.5	2.794621213	3.508132683	Higher in Normal	123	7	32	2	2	0	1	0	11	19
ENSMUSG00000081490.1	Gm11830	0.004196346	0.030665773	4	6	7.25	0.5	6.75	2.961109946	3.886082652	Higher in Normal	10	5	9	5	0	0	0	0	3	0
ENSMUSG00000109262.1	Gm44744	0.004196804	0.030665773	4	6	58	6.333333333	51.66666667	2.755478028	3.430944112	Higher in Normal	20	33	75	104	2	0	0	0	30	6
ENSMUSG00000094370.7	Gm3373	0.004214031	0.030753164	4	6	7.75	0.5	7.25	2.957262985	4.006652168	Higher in Normal	21	7	1	2	0	1	1	0	1	0
ENSMUSG00000090691.3	Gm3667	0.004216318	0.030753164	4	6	11.25	1.5	9.75	2.517414765	2.998850934	Higher in Normal	1	6	18	20	0	2	2	0	1	4
ENSMUSG00000104430.1	Gm37425	0.004235377	0.030836624	4	6	6.5	0	6.5	3.317195461	5.16036767	Higher in Normal	17	1	7	1	0	0	0	0	0	0
ENSMUSG00000086905.1	Gm13716	0.004247106	0.030885096	4	6	1.25	12	10.75	2.683189901	3.378944997	Higher in TRIM24	3	1	1	0	4	26	12	1	25	4
ENSMUSG00000090925.2	1810064F22Rik	0.004249346	0.030890448	4	6	36	5.666666667	30.33333333	2.497022781	2.951106179	Higher in Normal	58	12	37	37	2	1	0	0	23	8
ENSMUSG00000089871.1	Speer4cos	0.004274064	0.031029259	4	6	15.25	1.833333333	13.41666667	2.869217696	3.685664839	Higher in Normal	40	6	14	1	0	1	0	0	1	9
ENSMUSG00000109589.1	Gm45267	0.004296501	0.031147583	4	6	8.25	1.333333333	6.916666667	2.457255969	2.894560686	Higher in Normal	5	10	12	6	0	1	0	0	4	3
ENSMUSG00000085069.2	Gm13111	0.004297953	0.031150694	4	6	61	10.33333333	50.66666667	2.449893292	2.875276274	Higher in Normal	77	110	20	37	11	2	2	0	41	6
ENSMUSG00000094134.2	Ighv5-15	0.004317487	0.03126994	4	6	21.75	3	18.75	2.754264106	3.425264386	Higher in Normal	24	3	27	33	0	0	0	0	5	13
ENSMUSG00000073774.2	Kncn	0.00432745	0.031290935	4	6	24	1.833333333	22.16666667	3.023145758	4.107860528	Higher in Normal	82	1	13	0	1	0	0	2	2	6
ENSMUSG00000064225.6	Paqr9	0.004328588	0.031290935	4	6	1022.5	113.1666667	909.3333333	2.786473823	3.504539713	Higher in Normal	2693	1364	16	17	132	9	26	21	436	55
ENSMUSG00000076770.3	Trav8d-1	0.004360803	0.031493772	4	6	13	2	11	2.388781612	2.790246253	Higher in Normal	31	2	10	9	1	3	0	2	1	5
ENSMUSG00000090534.1	Gm4675	0.004395914	0.031687216	4	6	1.75	19.33333333	17.58333333	2.701495517	3.420818105	Higher in TRIM24	4	2	1	0	26	19	4	2	63	2
ENSMUSG00000082043.1	Gm12848	0.004418374	0.031834042	4	6	8	0.833333333	7.166666667	2.851886513	3.663119292	Higher in Normal	22	4	5	1	1	0	0	0	1	3
ENSMUSG00000048806.4	Ifnb1	0.004426234	0.03186805	4	6	36	2	34	3.110233995	4.390618252	Higher in Normal	0	0	57	87	1	0	0	1	4	6
ENSMUSG00000050424.9	Pnma5	0.004451445	0.031981644	4	6	12	1.166666667	10.83333333	2.818757197	3.604748422	Higher in Normal	20	2	25	1	0	1	0	0	3	3
ENSMUSG00000096464.2	Ighv2-2	0.004466798	0.03208423	4	6	73.75	10	63.75	2.813101947	3.566274566	Higher in Normal	3	22	123	147	2	2	2	0	0	54
ENSMUSG00000051617.3	Krt9	0.004488479	0.032198369	4	6	9.25	1.666666667	7.583333333	2.446360751	2.868986661	Higher in Normal	11	4	8	14	0	0	0	1	2	7
ENSMUSG00000055730.16	Ces2a	0.004502823	0.032274473	4	6	19.5	2.666666667	16.83333333	2.640705659	3.226406803	Higher in Normal	53	4	19	2	0	2	0	1	5	8
ENSMUSG00000086282.1	Sox5os4	0.004515	0.032346523	4	6	10.75	0.666666667	10.08333333	3.097837075	4.348637401	Higher in Normal	32	9	1	1	0	0	0	0	3	1
ENSMUSG00000029123.8	Stk32b	0.004520664	0.032375037	4	6	34	450.8333333	416.8333333	2.877191905	3.840473039	Higher in TRIM24	82	25	20	9	144	2	1673	850	8	28
ENSMUSG00000074673.15	Ttll9	0.004535261	0.032468754	4	6	22.5	3.833333333	18.66666667	2.629877221	3.193318889	Higher in Normal	51	7	22	10	1	0	0	0	6	16
ENSMUSG00000076543.2	Igkv4-74	0.004571635	0.032675387	4	6	99.25	14.66666667	84.58333333	2.537703178	3.033244824	Higher in Normal	20	71	127	179	4	1	2	0	63	18
ENSMUSG00000041358.1	Nutm1	0.004581082	0.032727543	4	6	17	3	14	2.422627023	2.839933663	Higher in Normal	45	2	19	2	1	1	2	2	2	10
ENSMUSG00000109232.1	Gm44577	0.004620838	0.032988338	4	6	9.5	0.5	9	3.172280469	4.531651167	Higher in Normal	28	3	7	0	0	0	0	0	1	2
ENSMUSG00000108874.1	Gm9711	0.004641216	0.03310216	4	6	11.75	0.666666667	11.08333333	3.085232483	4.348833002	Higher in Normal	39	2	6	0	1	0	0	0	2	1
ENSMUSG00000084983.7	Gm11789	0.004666074	0.033194535	4	6	9.75	0.666666667	9.083333333	2.985050682	4.072847259	Higher in Normal	26	5	8	0	0	0	0	1	2	1
ENSMUSG00000041653.4	Pnpla3	0.004712454	0.033415182	4	6	7417.25	576	6841.25	2.995133031	4.04847006	Higher in Normal	23517	6063	59	30	318	61	123	5	2767	182
ENSMUSG00000059706.4	A830035A12Rik	0.004725107	0.033469108	4	6	10.25	1	9.25	2.670247226	3.328614309	Higher in Normal	25	0	13	3	1	1	1	1	1	1
ENSMUSG00000083090.1	Gm11736	0.004731801	0.033470705	4	6	14	2.166666667	11.83333333	2.536699955	3.042366902	Higher in Normal	40	5	9	2	3	0	2	0	2	6
ENSMUSG00000034177.15	Rnf43	0.004755578	0.033595787	4	6	2334.75	295.1666667	2039.583333	2.431417012	2.857555316	Higher in Normal	806	486	4123	3924	861	26	64	20	718	82
ENSMUSG00000043635.12	Adamts3	0.004821773	0.03396099	4	6	692.25	102.5	589.75	2.418949573	2.838547037	Higher in Normal	202	74	1617	876	55	19	25	4	450	62
ENSMUSG00000102128.1	Gm18349	0.004829346	0.033982884	4	6	10.75	1	9.75	2.88372391	3.783385652	Higher in Normal	24	5	13	1	0	0	0	0	4	2
ENSMUSG00000065530.1	Mir99a	0.004833419	0.034003685	4	6	30.25	4.5	25.75	2.527359338	3.033393413	Higher in Normal	18	98	3	2	1	2	4	7	8	5
ENSMUSG00000081340.1	Gm12275	0.004851602	0.034092219	4	6	13.25	2.666666667	10.58333333	2.356557302	2.737687384	Higher in Normal	29	12	7	5	1	0	0	2	5	8
ENSMUSG00000034467.7	Dynlrb2	0.004929231	0.034502354	4	6	15.5	3.666666667	11.83333333	2.350294836	2.720123077	Higher in Normal	16	14	10	22	0	0	1	1	2	18
ENSMUSG00000112304.1	Gm35206	0.004955061	0.034643329	4	6	16	3.333333333	12.66666667	2.410808804	2.821031681	Higher in Normal	23	11	19	11	1	0	0	0	6	13
ENSMUSG00000107043.1	Gm42849	0.00497499	0.034742776	4	6	13.75	2.833333333	10.91666667	2.353309229	2.735385113	Higher in Normal	30	12	6	7	0	1	0	2	4	10
ENSMUSG00000076655.2	Ighv4-1	0.005018386	0.034973639	4	6	20.5	2.666666667	17.83333333	2.510365282	3.015057465	Higher in Normal	6	48	13	15	2	4	2	0	8	0
ENSMUSG00000050974.4	Gm9847	0.005055314	0.035166602	4	6	27.25	196.8333333	169.5833333	2.340214326	2.737834172	Higher in TRIM24	66	8	21	14	594	16	10	66	164	331
ENSMUSG00000108859.1	Gm44776	0.005058214	0.035178733	4	6	27.75	2	25.75	3.126387889	4.548046855	Higher in Normal	89	17	5	0	0	0	0	0	4	8
ENSMUSG00000073482.2	Gm10517	0.005067084	0.035216725	4	6	33.5	5.166666667	28.33333333	2.424252822	2.855485194	Higher in Normal	60	5	45	24	5	2	0	0	18	6
ENSMUSG00000019909.4	Fam162b	0.005087857	0.035331082	4	6	9.25	0.666666667	8.583333333	3.063079848	4.239181883	Higher in Normal	29	1	6	1	0	0	0	0	1	3
ENSMUSG00000107862.1	Gm44151	0.005137378	0.035566822	4	6	11.25	0.166666667	11.08333333	3.269032905	5.486565088	Higher in Normal	37	1	7	0	0	1	0	0	0	0
ENSMUSG00000052273.4	Dnah3	0.005151988	0.035643653	4	6	87.5	18.66666667	68.83333333	2.476010672	2.9430758	Higher in Normal	221	53	61	15	6	1	3	0	31	71
ENSMUSG00000073764.2	Gm12888	0.005159436	0.035678971	4	6	10.25	0.166666667	10.08333333	3.249994977	5.132694496	Higher in Normal	0	1	15	25	0	0	0	0	1	0
ENSMUSG00000042179.5	Pnliprp1	0.005181773	0.035817166	4	6	45.75	7.333333333	38.41666667	2.683050992	3.331216497	Higher in Normal	145	2	23	13	1	1	1	0	11	30
ENSMUSG00000082229.1	Nap1l2	0.005221019	0.036039353	4	6	5.75	41.83333333	36.08333333	2.325281375	2.718229006	Higher in TRIM24	7	2	9	5	1	4	81	53	12	100
ENSMUSG00000095438.1	Mir133a-1hg	0.005248098	0.036185257	4	6	7.5	0.833333333	6.666666667	2.729750062	3.461488848	Higher in Normal	11	9	10	0	1	0	1	0	1	2
ENSMUSG00000037482.2	Erv3	0.005280853	0.036361695	4	6	8.5	1	7.5	2.529216015	3.069778955	Higher in Normal	5	5	10	14	0	1	1	0	4	0
ENSMUSG00000100253.1	1700020N18Rik	0.005310776	0.03652117	4	6	5.25	0.333333333	4.916666667	2.886270236	3.815454981	Higher in Normal	3	1	10	7	0	0	0	1	0	1
ENSMUSG00000112902.1	Gm48884	0.005327915	0.036602968	4	6	8	1	7	2.603287815	3.198248097	Higher in Normal	14	1	9	8	1	0	0	0	3	2
ENSMUSG00000068231.4	Vmn1r43	0.005335608	0.036631024	4	6	0.25	9.833333333	9.583333333	3.10017144	4.873239919	Higher in TRIM24	1	0	0	0	0	0	8	17	17	17
ENSMUSG00000078691.3	Gm6821	0.005343259	0.036667013	4	6	6.25	0.166666667	6.083333333	3.130959713	4.61530147	Higher in Normal	17	1	6	1	0	0	0	0	1	0
ENSMUSG00000043003.14	Rasef	0.005369246	0.036745965	4	6	1909.25	204.1666667	1705.083333	2.54598798	3.077703607	Higher in Normal	696	541	3516	2884	672	6	59	10	454	24
ENSMUSG00000059654.7	Reg1	0.00538318	0.036783453	4	6	12.5	119.3333333	106.8333333	2.486866471	3.008904879	Higher in TRIM24	42	3	4	1	13	24	144	28	444	63
ENSMUSG00000039639.6	Kcne1	0.00539708	0.036820656	4	6	18.25	0.666666667	17.58333333	3.17030905	4.955164602	Higher in Normal	59	0	14	0	0	1	0	0	2	1

ENSMUSG00000094763.3	Gm1647	0.005478942	0.03730385	4	6	15.5	1.333333333	14.16666667	2.932672916	3.96753891	Higher in Normal	53	1	8	0	1	0	1	0	2	4
ENSMUSG00000042453.14	Reln	0.00551964	0.037530232	4	6	1537	241.3333333	1295.666667	2.422814183	2.864220391	Higher in Normal	384	289	3329	2146	17	15	676	8	166	566
ENSMUSG00000019102.10	Aldh3a1	0.005540847	0.037647731	4	6	38	815.6666667	777.6666667	2.730805129	3.537273346	Higher in TRIM24	18	12	60	62	11	3	31	37	346	4466
ENSMUSG00000081070.1	Gm13181	0.005547937	0.037672522	4	6	34	6.5	27.5	2.398947026	2.825894886	Higher in Normal	109	7	18	2	1	4	2	4	4	24
ENSMUSG00000041771.13	Slc24a4	0.005604075	0.03797749	4	6	49.75	11.5	38.25	2.36695326	2.768986663	Higher in Normal	110	6	60	23	2	2	3	0	12	50
ENSMUSG00000039323.18	Igfbp2	0.00567942	0.038385564	4	6	533.75	4367.333333	3833.583333	2.654901799	3.365619169	Higher in TRIM24	190	33	739	1173	20184	51	862	2820	580	1707
ENSMUSG00000052974.8	Cyp2f2	0.005694834	0.038464131	4	6	533.25	83.83333333	449.4166667	2.362429834	2.767680495	Higher in Normal	794	613	307	419	287	4	12	4	128	68
ENSMUSG00000024738.5	Pga5	0.005734427	0.038675314	4	6	6	0.333333333	5.666666667	3.005014589	4.133134662	Higher in Normal	16	1	6	1	0	0	0	0	1	1
ENSMUSG00000110757.1	Gm18228	0.005740668	0.038696448	4	6	5.25	0.333333333	4.916666667	2.934591516	3.93178097	Higher in Normal	11	2	7	1	0	0	0	0	1	1
ENSMUSG00000079277.9	Hoxd3	0.005744052	0.038710687	4	6	17	3.166666667	13.83333333	2.361963971	2.769668535	Higher in Normal	37	15	9	7	0	2	1	0	10	6
ENSMUSG00000076518.6	Igkv2-112	0.005751839	0.038746014	4	6	15.5	1.833333333	13.66666667	2.449134432	2.936647632	Higher in Normal	9	21	10	22	7	1	2	1	0	0
ENSMUSG00000021539.8	Lect2	0.005766642	0.038811386	4	6	0.25	5.5	5.25	2.961992814	4.245343924	Higher in TRIM24	1	0	0	0	0	8	12	3	4	6
ENSMUSG00000100798.1	Gm19589	0.005791922	0.038938497	4	6	12.5	83.83333333	71.33333333	2.414819519	2.888977016	Higher in TRIM24	34	1	9	6	2	174	156	25	59	87
ENSMUSG00000096316.7	Faiml	0.005793338	0.038939413	4	6	33.75	4.166666667	29.58333333	2.82916114	3.694212407	Higher in Normal	90	8	36	1	0	0	0	1	7	17
ENSMUSG00000033576.11	Apol6	0.005798984	0.038960164	4	6	4465.5	586.5	3879	2.678516556	3.355132893	Higher in Normal	7489	9842	252	279	529	24	129	25	2580	232
ENSMUSG00000047324.5	4931429P17Rik	0.00580862	0.03901609	4	6	28	4.833333333	23.16666667	2.604207189	3.199553816	Higher in Normal	69	15	13	15	0	1	0	0	9	19
ENSMUSG00000094094.2	Igkv5-45	0.005831348	0.039117184	4	6	7	1	6	2.639272005	3.267592784	Higher in Normal	5	6	11	6	0	0	1	0	0	5
ENSMUSG00000095609.1	Gm21188	0.005833774	0.039123214	4	6	0.25	18	17.75	3.096640483	5.150598741	Higher in TRIM24	0	1	0	0	3	0	9	0	19	77
ENSMUSG00000025468.15	Caly	0.005868739	0.039315509	4	6	25.5	4.666666667	20.83333333	2.398947986	2.834532592	Higher in Normal	74	9	17	2	3	3	0	1	7	14
ENSMUSG00000005373.13	Mlxipl	0.005869952	0.039315509	4	6	2454	317.6666667	2136.333333	2.579265516	3.158451906	Higher in Normal	7063	2429	181	143	187	251	60	19	1319	70
ENSMUSG00000097364.1	Gm26719	0.005871535	0.039317464	4	6	71.25	14.16666667	57.08333333	2.490877825	2.993494093	Higher in Normal	83	192	8	2	4	4	3	2	35	37
ENSMUSG00000036357.5	Gpr101	0.005886902	0.039368416	4	6	8	1.166666667	6.833333333	2.448775804	2.926252835	Higher in Normal	5	3	14	10	1	0	0	0	4	2
ENSMUSG00000085333.1	1700030A11Rik	0.005903284	0.039451974	4	6	7.75	0.166666667	7.583333333	3.153462202	4.806016633	Higher in Normal	9	0	18	4	0	0	0	0	1	0
ENSMUSG00000001182.9	Fgf23	0.005908987	0.039481424	4	6	303.25	36.5	266.75	2.563001715	3.128669145	Higher in Normal	27	12	580	594	5	17	29	0	121	47
ENSMUSG00000038044.8	Cct8l1	0.005911263	0.039487965	4	6	9.25	0.5	8.75	3.005616955	4.317984959	Higher in Normal	25	1	11	0	0	1	0	0	1	1
ENSMUSG00000108950.1	9130015G15Rik	0.00593726	0.039635538	4	6	11.5	1.833333333	9.666666667	2.351660705	2.761411616	Higher in Normal	18	3	19	6	2	0	0	1	6	2
ENSMUSG00000110929.1	Gm47757	0.005973655	0.039843489	4	6	52.75	8.166666667	44.58333333	2.531742618	3.072995761	Higher in Normal	72	78	28	33	17	0	0	0	15	17
ENSMUSG00000083534.1	H2-M6-ps	0.005988291	0.039906211	4	6	12	2	10	2.494494697	3.004660755	Higher in Normal	28	3	14	3	0	0	1	2	1	8
ENSMUSG00000040935.12	Padi6	0.005998905	0.039959453	4	6	53	4.666666667	48.33333333	2.94654089	4.04243794	Higher in Normal	163	0	48	1	2	2	0	0	7	17
ENSMUSG00000114132.1	Gm4808	0.006024748	0.04009433	4	6	12.75	0.333333333	12.41666667	3.203430026	5.105563908	Higher in Normal	38	1	12	0	0	0	0	0	2	0
ENSMUSG00000056605.6	Krt72	0.006073194	0.040308778	4	6	13	1	12	2.954091429	4.068308166	Higher in Normal	6	0	32	14	0	0	0	0	2	4
ENSMUSG00000099958.1	1700010B13Rik	0.006075177	0.040308778	4	6	25.75	3.666666667	22.08333333	2.627601578	3.263866028	Higher in Normal	75	1	26	1	1	2	2	0	4	13
ENSMUSG00000085653.7	Gm15179	0.006091713	0.040400886	4	6	21.25	2.5	18.75	2.698996157	3.424976696	Higher in Normal	50	17	18	0	0	2	5	0	1	7
ENSMUSG00000091754.8	Gm3636	0.006140711	0.040663845	4	6	93.25	7.666666667	85.58333333	2.643463113	3.30297619	Higher in Normal	2	33	138	200	0	6	12	13	15	0
ENSMUSG00000033156.9	Cst10	0.006149566	0.040695935	4	6	7.25	0.166666667	7.083333333	3.157445003	5.005579661	Higher in Normal	7	18	0	4	1	0	0	0	0	0
ENSMUSG00000004894.10	Hapln2	0.006172228	0.040828155	4	6	17.25	1.666666667	15.58333333	2.750321911	3.549843359	Higher in Normal	41	1	25	2	4	0	0	0	3	3
ENSMUSG00000106609.1	Gm43181	0.006178522	0.04085204	4	6	1	13	12	2.64290769	3.400911301	Higher in TRIM24	4	0	0	0	2	9	15	1	23	28
ENSMUSG00000028766.10	Alpl	0.006183059	0.040864291	4	6	5542.75	597.3333333	4945.416667	2.554258977	3.121502815	Higher in Normal	826	1395	8977	10973	1358	19	120	58	1994	35
ENSMUSG00000035653.16	Lrfn5	0.006214776	0.041020486	4	6	87.75	19.5	68.25	2.330443523	2.724433911	Higher in Normal	122	30	99	100	9	1	1	0	38	68
ENSMUSG00000041857.10	Oosp1	0.006216887	0.041025529	4	6	28	5.666666667	22.33333333	2.532492491	3.076399753	Higher in Normal	59	40	9	4	0	0	2	1	8	23
ENSMUSG00000086181.1	C230034O21Rik	0.006229633	0.041075322	4	6	16	1.5	14.5	2.667550937	3.370226303	Higher in Normal	18	1	35	10	0	0	3	1	5	0
ENSMUSG00000085180.1	AI838599	0.006238607	0.041108251	4	6	237	43.83333333	193.1666667	2.37223774	2.796405772	Higher in Normal	379	231	212	126	10	7	0	11	184	51
ENSMUSG00000032987.9	Olfr281	0.00625332	0.04116788	4	6	31	4	27	2.819667089	3.698742144	Higher in Normal	88	6	29	1	1	0	0	0	5	18
ENSMUSG00000026166.14	Ccl20	0.006258218	0.041182308	4	6	94.25	12.83333333	81.41666667	2.340456997	2.744552455	Higher in Normal	15	5	143	214	16	1	20	4	33	3
ENSMUSG00000110390.1	Gm45869	0.006261645	0.041186986	4	6	7.75	1.333333333	6.416666667	2.416423549	2.881624954	Higher in Normal	10	12	6	3	2	0	0	0	3	3
ENSMUSG00000092494.1	Gm20420	0.006274359	0.04124389	4	6	8.75	0.833333333	7.916666667	2.811629602	3.736569598	Higher in Normal	24	1	9	1	0	1	0	0	1	3
ENSMUSG00000095774.2	Olfr970	0.006293541	0.041325395	4	6	10.25	0.666666667	9.583333333	2.974896817	4.173035133	Higher in Normal	33	2	6	0	0	0	1	0	2	1
ENSMUSG00000078575.2	Gm12887	0.00639319	0.041808385	4	6	33	5.5	27.5	2.645513425	3.305322332	Higher in Normal	51	28	30	23	0	0	0	0	11	22
ENSMUSG00000026969.3	Fam166a	0.006490376	0.04232576	4	6	7.5	1	6.5	2.583309154	3.191787855	Higher in Normal	7	2	10	11	0	0	0	0	3	3
ENSMUSG00000102364.1	Ighv8-5	0.006495691	0.042351351	4	6	15.25	2	13.25	2.387797433	2.843121132	Higher in Normal	19	1	16	25	5	2	2	0	0	3
ENSMUSG00000075465.2	Gm10837	0.006506855	0.042415055	4	6	20.75	2	18.75	2.934664843	4.052511636	Higher in Normal	71	6	6	0	0	0	1	0	3	8
ENSMUSG00000099418.2	Gm6657	0.00653628	0.042579515	4	6	8.5	0.833333333	7.666666667	2.778840796	3.674211439	Higher in Normal	26	5	2	1	0	1	0	0	2	2
ENSMUSG00000051452.8	Gm11437	0.006575341	0.042733398	4	6	35	5.333333333	29.66666667	2.679951587	3.390483723	Higher in Normal	111	12	15	2	1	0	0	1	10	20
ENSMUSG00000084816.1	Platr29	0.006595932	0.042851518	4	6	19.25	3.166666667	16.08333333	2.574638185	3.173196002	Higher in Normal	63	4	9	1	1	0	1	1	4	12
ENSMUSG00000110399.1	Gm45238	0.00659845	0.042856158	4	6	17	1.666666667	15.33333333	2.740224977	3.550704052	Higher in Normal	47	5	16	0	2	0	0	1	5	2
ENSMUSG00000057216.6	Gm10807	0.006612106	0.042922482	4	6	9.25	0	9.25	3.238586744	5.656433767	Higher in Normal	23	1	13	0	0	0	0	0	0	0
ENSMUSG00000093920.2	Olfr463	0.006635	0.043043836	4	6	10	0.5	9.5	3.102356792	4.605750861	Higher in Normal	33	1	6	0	0	0	0	0	1	2
ENSMUSG00000112723.1	Gm9110	0.00663583	0.043043836	4	6	26.25	3.166666667	23.08333333	2.804469579	3.688318986	Higher in Normal	83	2	18	2	1	0	0	0	6	12
ENSMUSG00000111003.1	Gm47122	0.006650064	0.043099436	4	6	6.5	1.166666667	5.333333333	2.391427661	2.839003915	Higher in Normal	11	4	5	6	0	0	1	0	2	4
ENSMUSG00000021097.15	Clmn	0.006653045	0.04310958	4	6	6405.75	876.1666667	5529.583333	2.358688652	2.783154837	Higher in Normal	2606	3138	11736	8143	2587	87	106	57	2263	157
ENSMUSG00000075068.3	Olfr140	0.006659855	0.043126176	4	6	10.25	0.666666667	9.583333333	3.02921681	4.327153099	Higher in Normal	18	2	21	0	0	0	0	0	1	3
ENSMUSG00000098083.1	Gm8957	0.006665075	0.043142093	4	6	22.75	1.333333333	21.41666667	3.08241883	4.671511387	Higher in Normal	74	0	17	0	0	0	1	0	2	5
ENSMUSG00000060890.11	Arr3	0.006692199	0.043273772	4	6	27.25	4.333333333	22.91666667	2.474485477	2.989033688	Higher in Normal	66	5	37	1	4	1	0	1	9	11
ENSMUSG00000064224.10	Gsdma3	0.006696373	0.043284313	4	6	31	5.666666667	25.33333333	2.430681102	2.905144557	Higher in Normal	40	2	42	40	1	2	0	0	10	21
ENSMUSG00000113246.1	Gm36752	0.006707352	0.04333237	4	6	27.25	4.333333333	22.91666667	2.618808707	3.266897932	Higher in Normal	79	9	20	1	2	1	0	0	6	17
ENSMUSG00000052281.14	Tspan32os	0.006717288	0.043361454	4	6	5.25	0.833333333	4.416666667	2.439860411	2.946743564	Higher in Normal	4	6	6	5	0	1	0	0	1	3
ENSMUSG00000030866.8	Ern2	0.0067344	0.043443027	4	6	32.5	3.333333333	29.16666667	2.880068837	3.906982954	Higher in Normal	107	2	21	0	0	1	1	0	5	13
ENSMUSG00000076534.6	Igkv12-89	0.006739816	0.043449907	4	6	36	3.833333333	32.16666667	2.530448122	3.109661605	Higher in Normal	8	82	27	27	0	13	5	0	2	3
ENSMUSG00000110400.1	Gm45416	0.006821663	0.043875861	4	6	6	0.666666667	5.333333333	2.651200991	3.386462905	Higher in Normal	9	3	11	1	0	1	0	0	1	2
ENSMUSG00000112666.1	Gm48485	0.006863796	0.044053302	4	6	8.5	1.5	7	2.322357708	2.73552845	Higher in Normal	16	8	6	4	0	1	1	0	5	2
ENSMUSG00000100247.1	A530053M12Rik	0.006875161	0.04409836	4	6	1.25	9.666666667	8.416666667	2.528790383	3.170929363	Higher in TRIM24	4	0	0	1	3	26	8	6	5	10
ENSMUSG00000083732.1	Gm14197	0.006887385	0.044167461	4	6	5.25	0.333333333	4.916666667	2.861203678	3.81707926	Higher in Normal	1	2	12	6	0	0	0	0	1	1
ENSMUSG00000086268.1	Gm11670	0.006900175	0.04423865	4	6	13	1.833333333	11.16666667	2.54834293	3.142711758	Higher in Normal	38	7	5	2	1	0	0	1	6	3
ENSMUSG00000081388.1	Gm11801	0.006940013	0.044430091	4	6	15.5	0.666666667	14.83333333	3.164134673	5.115473634	Higher in Normal	46	1	15	0	0	0	0	0	0	4
ENSMUSG00000036892.12	Prodh2	0.006991651	0.044685516	4	6	26.25	3.166666667	23.08333333	2.684195701	3.426780455	Higher in Normal	83	3	19	0	2	0	1	1	8	7
ENSMUSG00000090105.2	Gm15890	0.007016661	0.044795258	4	6	5.25	0.5	4.75	2.581400588	3.31124099	Higher in Normal	5	2	8	6	0	1	0	1	1	0
ENSMUSG00000056370.15	Sftpb	0.007084119	0.045087217	4	6	40.25	6.666666667	33.58333333	2.462138196	2.977076975	Higher in Normal	116	1	42	2	1	4	4	1	10	20
ENSMUSG00000029372.7	Ppbp	0.007094517	0.045143962	4	6	57.25	532.3333333	475.0833333	2.326871215	2.764772193	Higher in TRIM24	103	109	10	7	98	54	399	69	2124	450
ENSMUSG00000021553.13	Slc28a3	0.007109275	0.045181214	4	6	1611.75	247.5	1364.25	2.37530561	2.821783295	Higher in Normal	1257	286	2618	2286	145	33	51	3	1156	97
ENSMUSG00000078234.6	Klhdc7a	0.007122714	0.045228864	4	6	8151	1175.333333	6975.666667	2.418605567	2.898270551	Higher in Normal	9537	6295	8763	8009	2059	90	246	19	4531	107
ENSMUSG00000099216.1	Obox8	0.007134075	0.045262247	4	6	22.75	2.833333333	19.91666667	2.496824883	3.049506454	Higher in Normal	27	1	49	14	0	3	1	0	10	3
ENSMUSG00000029154.13	Cwh43	0.007158915	0.045383021	4	6	68.25	585.5	517.25	2.513571846	3.123614531	Higher in TRIM24	60	55	76	82	1442	230	36	3	1759	43

ENSMUSG00000103965.1	Gm30173	0.007189634	0.04550197	4	6	22.75	3.333333333	19.41666667	2.558197418	3.167260043	Higher in Normal	57	28	2	4	4	0	1	0	10	5
ENSMUSG00000099552.1	9830004L10Rik	0.007215491	0.045618207	4	6	10.75	1.666666667	9.083333333	2.567186875	3.185330027	Higher in Normal	25	9	8	1	2	0	0	0	2	6
ENSMUSG00000097740.1	E030044B06Rik	0.007251688	0.045791667	4	6	8	1	7	2.591707862	3.250256496	Higher in Normal	14	2	7	9	0	0	3	0	0	3
ENSMUSG00000096410.2	Ighv1-19	0.007259543	0.045811103	4	6	11	1.666666667	9.333333333	2.508180388	3.074561534	Higher in Normal	1	20	11	12	0	0	4	0	2	4
ENSMUSG00000109328.2	Olfr1438-ps1	0.007280976	0.045889292	4	6	21.5	3	18.5	2.640870708	3.3450247	Higher in Normal	73	8	4	1	2	2	0	0	3	11
ENSMUSG00000025955.13	Akr1cl	0.007302261	0.046004397	4	6	131.75	21	110.75	2.654907978	3.370851251	Higher in Normal	138	365	12	12	7	1	2	0	68	48
ENSMUSG00000067704.1	Wfdc13	0.007310479	0.04601702	4	6	7.5	1.333333333	6.166666667	2.396831746	2.865770194	Higher in Normal	15	5	6	4	1	0	0	0	3	4
ENSMUSG00000078143.3	Gm17344	0.007335988	0.046150034	4	6	10.5	2.166666667	8.333333333	2.342893246	2.76942262	Higher in Normal	27	6	6	3	1	1	1	0	1	9
ENSMUSG00000063157.9	Csn2	0.007339987	0.046165653	4	6	87078.25	10751.83333	76326.41667	2.482183398	3.020663061	Higher in Normal	87493	845	108687	151288	5479	5082	5520	235	46670	1525
ENSMUSG00000082771.1	Gm12803	0.007378116	0.046338463	4	6	8.25	0.333333333	7.916666667	3.064980402	4.686184303	Higher in Normal	27	5	0	1	1	0	0	0	0	1
ENSMUSG00000089301.1	Gm24841	0.007392603	0.04637256	4	6	7.75	0.166666667	7.583333333	3.143226333	4.965315539	Higher in Normal	23	1	7	0	0	0	0	0	0	1
ENSMUSG00000073063.3	Hbq1b	0.007413713	0.046466187	4	6	21.5	2.333333333	19.16666667	2.655072151	3.397677792	Higher in Normal	70	10	6	0	0	4	2	1	1	6
ENSMUSG00000060560.8	Ces4a	0.007435182	0.046542429	4	6	61	6.666666667	54.33333333	2.883089856	3.969078346	Higher in Normal	184	2	56	2	0	1	0	0	9	30
ENSMUSG00000084863.1	Gm12523	0.007438106	0.046542429	4	6	5.75	0.5	5.25	2.865013539	3.845070835	Higher in Normal	14	6	1	2	0	0	0	0	1	2
ENSMUSG00000098284.7	A330093E20Rik	0.007445107	0.04656945	4	6	9.25	1.333333333	7.916666667	2.453612019	2.985731925	Higher in Normal	19	1	13	4	0	2	1	0	1	4
ENSMUSG00000109847.1	Gm45278	0.007445484	0.04656945	4	6	8.75	1.333333333	7.416666667	2.462697345	2.999119811	Higher in Normal	21	5	8	1	0	1	1	0	3	3
ENSMUSG00000103658.1	Gm37201	0.007489963	0.046751621	4	6	0.5	6.5	6	2.617085318	3.472987499	Higher in TRIM24	1	1	0	0	11	1	9	1	3	14
ENSMUSG00000035551.6	Igfbpl1	0.007491977	0.046754603	4	6	21.5	3.666666667	17.83333333	2.465674032	2.991527297	Higher in Normal	49	4	18	15	0	0	0	2	9	11
ENSMUSG00000064263.6	Platr26	0.007550008	0.047039624	4	6	9.25	0.5	8.75	3.091158306	4.685534368	Higher in Normal	19	12	6	0	0	0	0	0	0	3
ENSMUSG00000101695.1	1700094J05Rik	0.007557537	0.047067272	4	6	6	0.5	5.5	2.797316362	3.688028898	Higher in Normal	13	1	7	3	0	0	0	0	2	1
ENSMUSG00000042631.7	Xkr7	0.00762672	0.047391505	4	6	14.75	0.666666667	14.08333333	3.125138075	5.027921241	Higher in Normal	40	1	18	0	0	0	0	0	0	4
ENSMUSG00000107350.1	Gm19610	0.007636982	0.047435909	4	6	4.25	48.16666667	43.91666667	2.475391128	3.064721272	Higher in TRIM24	10	0	2	5	0	6	22	45	42	174
ENSMUSG00000026514.14	Cnih3	0.007723862	0.047731345	4	6	34	7.166666667	26.83333333	2.378653576	2.837373526	Higher in Normal	81	6	32	17	4	0	0	1	10	28
ENSMUSG00000105713.1	Gm43827	0.007767973	0.047899002	4	6	8.75	0.333333333	8.416666667	3.010970469	4.528172248	Higher in Normal	19	1	15	0	1	0	0	0	1	0
ENSMUSG00000108141.1	Gm44079	0.007817377	0.048124441	4	6	22.5	2.666666667	19.83333333	2.771833426	3.685844172	Higher in Normal	46	41	1	2	0	1	0	0	8	7
ENSMUSG00000097671.2	1700015O11Rik	0.007823024	0.048130014	4	6	9.5	0.666666667	8.833333333	2.95715818	4.231914169	Higher in Normal	28	5	5	0	0	0	0	0	2	2
ENSMUSG00000087410.7	2310065F04Rik	0.007864494	0.048325018	4	6	25	3.5	21.5	2.729537284	3.569581351	Higher in Normal	78	15	7	0	1	0	2	0	3	15
ENSMUSG00000106514.1	Mir6976	0.007916309	0.048492606	4	6	5	0.333333333	4.666666667	2.811191335	3.749925687	Higher in Normal	1	2	11	6	0	0	0	0	1	1
ENSMUSG00000036816.4	Atoh7	0.007932726	0.048569405	4	6	12.25	2.166666667	10.08333333	2.552300746	3.171399203	Higher in Normal	18	19	11	1	1	0	0	0	3	9
ENSMUSG00000073094.9	Smim9	0.007959246	0.048653508	4	6	6	0.833333333	5.166666667	2.492350537	3.082543809	Higher in Normal	15	4	2	3	1	0	0	1	1	2
ENSMUSG00000111495.1	Gm5366	0.00806678	0.049133289	4	6	15.5	0.833333333	14.66666667	3.085280722	4.90747317	Higher in Normal	46	0	14	2	0	0	0	0	0	5
ENSMUSG00000100212.1	Gm5522	0.008090575	0.049236981	4	6	6.5	0.166666667	6.333333333	3.029886345	4.689786405	Higher in Normal	7	7	12	0	0	1	0	0	0	0
ENSMUSG00000030834.7	Abcc6	0.008102359	0.049251473	4	6	37	7	30	2.492250109	3.059099132	Higher in Normal	99	5	31	13	0	4	1	0	4	33
ENSMUSG00000112047.1	Gm47852	0.008168901	0.049547149	4	6	7.25	0.333333333	6.916666667	2.978726399	4.436727701	Higher in Normal	21	2	6	0	0	0	0	1	0	1
ENSMUSG00000054013.6	Tmem179	0.008175303	0.049576106	4	6	258.75	49	209.75	2.331851879	2.769559861	Higher in Normal	408	599	15	13	12	11	14	48	159	50
ENSMUSG00000065515.1	Mir152	0.008179016	0.049578875	4	6	7.75	0	7.75	3.147202519	5.410761425	Higher in Normal	18	2	11	0	0	0	0	0	0	0
ENSMUSG00000113702.1	Gm35558	0.008213009	0.049690291	4	6	11.25	1.833333333	9.416666667	2.384465203	2.872963327	Higher in Normal	32	8	1	4	2	1	1	0	4	3
ENSMUSG00000056600.4	Olfr90	0.008225334	0.049750691	4	6	26.5	5	21.5	2.535360808	3.14467468	Higher in Normal	80	6	15	5	1	0	0	1	5	23
ENSMUSG00000034533.10	Scn10a	0.00823921	0.049795052	4	6	40.25	8	32.25	2.32324719	2.757119444	Higher in Normal	129	11	20	1	3	6	1	4	5	29
ENSMUSG00000050087.3	Cby3	0.008277922	0.049949694	4	6	7.25	1	6.25	2.583600907	3.264102838	Higher in Normal	19	2	5	3	0	0	0	1	1	4
ENSMUSG00000086645.2	Gm15743	0.008285731	0.049986909	4	6	19.25	2.5	16.75	2.603605706	3.305000528	Higher in Normal	56	5	14	2	0	2	0	0	7	6
