# Supplementary material for: Mammary-specific expression of Trim24 establishes a mouse model of human metaplastic breast cancer
Source: Nat Commun. 2021 Sep 10;12:5389. doi: 10.1038/s41467-021-25650-z (PMC8433435; doi:10.1038/s41467-021-25650-z)
Supplement: Supplementary file 10 — Dataset 7 [file 41467_2021_25650_MOESM10_ESM.pdf]

**Supplementary Table 7: List of antibodies used in RPPA and normalized linear scores associated with Cre control and TRIM24-driven tumors.**

| Antibodies | Cre-1 | Cre-2 | Trim24COE-897.1 | Trim24COE-897.2 | Trim24COE-64.1 | Trim24COE-64.2 |
|---|---|---|---|---|---|---|
| N-Cadherin | 0.95770565 | 0.96599156 | 1.013776543 | 1.000375075 | 1.009022537 | 1.039834126 |
| B-Raf | 0.93865789 | 0.95576823 | 1.004370039 | 1.005287521 | 1.018259421 | 1.022198541 |
| NF-kB-p65_pS536 | 0.95314089 | 0.92805396 | 1.001754007 | 1.004232252 | 1.137117894 | 1.154919515 |
| b-Catenin_pT41_S45 | 0.87332292 | 0.84090276 | 1.002564931 | 1.003448897 | 1.167114294 | 1.128178054 |
| Elk1_pS383 | 0.95821458 | 0.93415014 | 1.059989743 | 1.091141023 | 0.996342409 | 0.965474792 |
| MIF | 0.94842029 | 0.9369278 | 1.054374118 | 1.05714489 | 0.993507045 | 0.968190064 |
| Bak | 0.94585477 | 0.96625489 | 1.048399927 | 1.112049507 | 0.951182948 | 0.972248109 |
| Sox2 | 1.02089994 | 0.98114902 | 1.095423358 | 1.150353426 | 1 | 0.895960296 |
| Merlin | 0.92583504 | 0.91450189 | 1.173486123 | 1.191485695 | 0.970345282 | 0.990370813 |
| 43377 | 0.73937169 | 0.76362871 | 1.015100212 | 1.01611125 | 1.007722736 | 1 |
| MDM2_pS166 | 0.74069458 | 0.72945429 | 1.000222744 | 1.005711457 | 1.060342506 | 1.003464052 |
| Jagged1 | 0.78419102 | 0.80685208 | 1.004836182 | 1.00125486 | 1.049099815 | 1.056763221 |
| YB1_pS102 | 0.80492401 | 0.8103776 | 1.005341674 | 1.000766553 | 1.103368337 | 1.083126009 |
| PAICS | 0.77533462 | 0.80223282 | 1.09604395 | 1.074497406 | 0.971477958 | 0.989286111 |
| MMP2 | 0.70854228 | 0.69974975 | 1.049075424 | 1.052845499 | 0.998964175 | 0.962964071 |
| EGFR_pY1173 | 0.72817686 | 0.73298452 | 1.00369186 | 1.002360279 | 1.131120488 | 1.102966984 |
| 4E-BP1_pS65 | 0.74190259 | 0.76565059 | 1.00539377 | 1.000716228 | 1.159419462 | 1.146934794 |
| GCN5L2 | 0.60572109 | 0.59799206 | 1.128832609 | 1.111152632 | 1.005645494 | 0.956565738 |
| IR-b | 0.61533501 | 0.6179095 | 1.080004618 | 1.067711457 | 1.021049954 | 0.941813733 |
| PDGFR-b | 0.62428942 | 0.61669428 | 1.071547421 | 1.163867705 | 0.961924744 | 0.998434699 |
| Raptor | 0.63577728 | 0.61992393 | 1.018305676 | 1.010383143 | 1.004575074 | 1.017248347 |
| eIF4G | 0.61667181 | 0.62843111 | 1.024446798 | 1.0365794 | 0.998544691 | 0.994387118 |
| AMPKa | 0.5230509 | 0.49322317 | 1.019786421 | 1.071598536 | 1.009133727 | 0.960633925 |
| eEF2 | 0.44266536 | 0.4406616 | 1.050583591 | 1.037856409 | 0.993179174 | 0.968504049 |
| PKM2 | 0.43553005 | 0.4287731 | 1.003444839 | 1.002598902 | 1.080579535 | 0.997941694 |
| PAK1 | 0.47139788 | 0.47791086 | 1.000170356 | 1.005762064 | 1.180133951 | 1.097836812 |
| b-Actin | 0.69406779 | 0.69469409 | 1.003592632 | 1.002456134 | 1.223329232 | 1.195718159 |
| RSK | 0.64772628 | 0.63442144 | 1.004425854 | 1.001651238 | 1.315556286 | 1.228071322 |
| Mcl-1 | 0.78616194 | 0.80480197 | 1.002966441 | 1.003061036 | 1.236430512 | 1.184764446 |
| A-Raf | 0.75248921 | 0.75179688 | 1.004822744 | 1.001267841 | 1.284141458 | 1.21815923 |
| Histone-H3 | 0.77820968 | 0.765203 | 1.002197122 | 1.003804202 | 1.627911794 | 1.615273309 |
| p38_pT180_Y182 | 1.00895339 | 1.1069253 | 1.07129813 | 1.044458375 | 0.759116035 | 0.828368356 |
| PAK4 | 1.02278883 | 1.04992974 | 1.057996429 | 1.051361107 | 0.760871935 | 0.809729321 |
| IGFRb | 1.02244761 | 1.01506334 | 1.058709344 | 1.075740122 | 0.832603272 | 0.868937342 |
| PEA-15_pS116 | 1.07283672 | 1.11588341 | 1.009879291 | 1.020347367 | 0.869910747 | 0.877175558 |
| Glutaminase | 1.016186 | 1.02083732 | 1.17965329 | 1.173482699 | 0.826838791 | 0.849115622 |
| Gys_pS641 | 0.98081555 | 1.05345319 | 1.191643591 | 1.245571264 | 0.675831924 | 0.633371381 |
| Caveolin-1 | 1.04543624 | 0.99386503 | 1.338080589 | 1.248747682 | 0.578615008 | 0.553732926 |
| c-Myc | 0.92739218 | 0.98529259 | 1.369447929 | 1.429798911 | 0.99236179 | 0.953582509 |
| DJ1 | 0.93786434 | 0.88057058 | 1.392180806 | 1.305276179 | 1.037174172 | 0.926372455 |
| HSP70 | 0.73440859 | 0.73078934 | 1.523382853 | 1.529354536 | 0.987792555 | 0.973662518 |
| Cox2 | 0.6274945 | 0.65146765 | 1.593415081 | 1.513981154 | 0.978844178 | 0.982231887 |
| Gys | 0.90865274 | 0.92948856 | 1.636258644 | 1.6137447 | 0.993871481 | 0.967841064 |
| PAR | 1.00381898 | 1.03224122 | 1.706421078 | 1.679261818 | 0.786306659 | 0.802611745 |
| p16INK4a | 0.65017043 | 0.6951588 | 1.789476326 | 1.873252634 | 0.966336294 | 0.99421 |
| MCT4 | 0.49305181 | 0.47591022 | 1.864229649 | 1.877121069 | 0.950604808 | 1.009275181 |
| Connexin-43 | 0.38754564 | 0.39145443 | 2.256522525 | 2.304875909 | 0.97829135 | 0.9827613 |
| Ets-1 | 1.06001323 | 1.02765388 | 1.013962239 | 1.014873886 | 1.003775365 | 0.936077714 |
| Cyclin-D1 | 1.00742668 | 1.0181704 | 1.032596757 | 1.037494557 | 0.964708668 | 0.948587385 |
| ARID1A | 1.00069189 | 0.98020102 | 1.019351782 | 1.018705226 | 0.965248697 | 0.973071597 |
| Beclin | 1.07356965 | 1.05105947 | 1.009174632 | 1.02892593 | 0.966350279 | 0.948819656 |
| TTF1 | 1.07907206 | 1.0817636 | 1.003884499 | 1.019499773 | 0.946344312 | 0.943975806 |
| XRCC1 | 1.46898485 | 1.46704647 | 1.000480532 | 1.003395618 | 0.924688655 | 0.942840458 |
| GATA6 | 1.30911959 | 1.34667823 | 1.005685757 | 1.000434167 | 0.939036855 | 0.932042162 |
| Rab25 | 1.73131391 | 1.81516832 | 1.003371215 | 1.002670023 | 0.622065086 | 0.573538217 |
| S6_pS240_S244 | 0.60277173 | 0.6194196 | 1.005211327 | 1.000892468 | 1.786186132 | 1.710506087 |
| PMS2 | 2.03949966 | 2.07899107 | 1.001336015 | 1.004636034 | 0.763075146 | 0.811253703 |
| Progesterone-Receptor | 2.09149289 | 2.32927119 | 1.005670622 | 1.000448788 | 0.864227164 | 0.897852552 |
| YAP_pS127 | 0.69084171 | 0.67689099 | 5.575401155 | 5.468664352 | 1.026926175 | 0.936186399 |
| PDK1 | 1.00919165 | 1.02728696 | 4.621940608 | 4.76723522 | 0.583590944 | 0.542776479 |
| PD-L1 | 1.01998813 | 1.01733129 | 3.640996363 | 3.54180767 | 0.44119037 | 0.449639369 |

| | | | | | | |
|---|---|---|---|---|---|---|
| **FoxO3a-(p_S318-S321)** | 0.91478852 | 0.89605532 | 0.996012062 | 1.009778991 | 1.099797219 | 1.044327508 |
| **Rictor_pT1135** | 0.87250192 | 0.86802619 | 0.990627141 | 1.014980844 | 1.05536394 | 1.050868467 |
| **Creb** | 0.87601138 | 0.85330058 | 0.998349243 | 1.007521266 | 1.072356788 | 1.082102767 |
| **INPP4b** | 0.98801732 | 0.98079465 | 0.985246513 | 1.016599763 | 1.000362815 | 1.034422785 |
| **p27_pT198** | 0.8423934 | 0.98254956 | 0.982984675 | 1.042845812 | 0.998566104 | 1.005085513 |
| **c-IAP2** | 0.85227779 | 0.84478427 | 0.988407739 | 1.017124793 | 1.180105952 | 1.118059596 |
| **Notch3** | 0.83934347 | 0.88074735 | 0.996067705 | 1.009725239 | 1.202167067 | 1.191879707 |
| **Stat3_pY705** | 0.7611933 | 0.75973186 | 0.990165764 | 1.015426535 | 1.032566642 | 1.039285727 |
| **WIPI1** | 0.69840255 | 0.73851097 | 0.968788679 | 1.05432796 | 1.053199169 | 1.050481179 |
| **Bcl-xL** | 0.78541886 | 0.80370202 | 0.984318452 | 1.027766055 | 1.037949432 | 1.027317403 |
| **JNK_pT183_Y185** | 0.68259562 | 0.67447767 | 0.991847978 | 1.01380151 | 1.141672115 | 1.087434338 |
| **Bad_pS112** | 0.75170368 | 0.81027075 | 0.993058762 | 1.012631889 | 1.117049534 | 1.124961173 |
| **TSC1** | 0.62967664 | 0.62293449 | 0.99513263 | 1.010628525 | 1.150485832 | 1.039479565 |
| **mTOR** | 0.62906152 | 0.62550366 | 0.999667191 | 1.006248124 | 1.116781313 | 1.037175603 |
| **p44-42-MAPK** | 0.58848151 | 0.60367781 | 0.998981717 | 1.006910294 | 1.190240952 | 1.131067172 |
| **Chk1_pS296** | 0.55245693 | 0.55850188 | 0.989838063 | 1.015743096 | 1.078185952 | 1.085044015 |
| **4E-BP1** | 0.50527012 | 0.48886308 | 0.978794025 | 1.026411675 | 1.094387802 | 1.049345415 |
| **Atg7** | 0.6621546 | 0.6555573 | 0.996379501 | 1.009424043 | 1.244692341 | 1.194660116 |
| **Rictor** | 0.76579999 | 0.77938872 | 0.999022681 | 1.006870723 | 1.22899673 | 1.176347567 |
| **Caspase-3** | 0.83510657 | 0.8408703 | 0.943604696 | 1.06040469 | 1.396419268 | 1.322360208 |
| **RPA32_pS4_S8** | 0.81102035 | 0.79844949 | 0.98955253 | 1.016018921 | 1.290037092 | 1.260828435 |
| **Bim** | 0.65088587 | 0.61081407 | 0.98783159 | 1.017681356 | 1.446783804 | 1.403170232 |
| **PKCa** | 0.61846729 | 0.6204974 | 0.986877818 | 1.018602703 | 1.434816877 | 1.353448059 |
| **Smad3** | 0.78080847 | 0.75145026 | 0.988851127 | 1.01669648 | 1.536316932 | 1.427104832 |
| **NAPSIN-A** | 0.77835101 | 0.74756803 | 0.9910535 | 1.014568979 | 1.482065764 | 1.394009714 |
| **c-Kit** | 0.79662859 | 0.77416759 | 0.974104017 | 1.030942239 | 1.654111329 | 1.520108515 |
| **TIGAR** | 0.51647754 | 0.5194255 | 0.972439259 | 1.032550401 | 1.408777107 | 1.350346391 |
| **p38-MAPK** | 0.53914503 | 0.55317422 | 0.973110309 | 1.031902164 | 1.366224166 | 1.252218744 |
| **Fibronectin** | 0.33641277 | 0.30531015 | 0.87807914 | 1.325712692 | 1.142273011 | 1.612263353 |
| **Akt** | 0.48303607 | 0.50465493 | 0.999139259 | 1.006758108 | 1.59743719 | 1.516142293 |
| **Stat3** | 0.32952512 | 0.33577567 | 0.978254349 | 1.026933004 | 1.470586091 | 1.414420881 |
| **ERCC5** | 1.1051744 | 1.09613648 | 0.997520801 | 1.008321543 | 0.768017171 | 0.872931087 |
| **Slfn11** | 1.05894589 | 1.06583021 | 0.866868135 | 1.033982449 | 0.980780917 | 0.962225527 |
| **PDHK1** | 1.04013935 | 1.02721158 | 0.994473762 | 1.003644182 | 0.984287918 | 0.981322642 |
| **XPF** | 1.01751115 | 1.02843576 | 0.996329525 | 1.008463722 | 0.997688748 | 0.964185477 |
| **SDHA** | 1.2493162 | 1.29207533 | 0.980329469 | 1.024928431 | 0.877168056 | 0.870078072 |
| **B7-H4** | 1.44559706 | 1.51408947 | 0.995581999 | 1.010194433 | 0.913418095 | 0.918655646 |
| **ER** | 1.52021354 | 1.5356687 | 0.999978339 | 1.005947553 | 0.921971017 | 0.911230859 |
| **eEF2K** | 1.61075322 | 1.57563835 | 0.97847859 | 1.007500729 | 0.895982897 | 0.93884425 |
| **CD4** | 1.68622748 | 1.51462776 | 0.889807824 | 1.011170652 | 0.976637065 | 0.873625883 |
| **HER2_pY1248** | 1.43232438 | 1.43233738 | 0.971146283 | 1.03055569 | 0.898440444 | 0.916400909 |
| **CD20** | 1.40601202 | 1.3578484 | 0.995153306 | 1.010608553 | 0.909150023 | 0.925246367 |
| **CD134** | 1.70792592 | 1.72257286 | 0.970795339 | 1.0063258 | 0.891799848 | 0.93998801 |
| **p53** | 1.6560036 | 1.7179942 | 0.991324424 | 1.014307266 | 0.938788543 | 0.882855009 |
| **WIPI2** | 1.75703578 | 1.83282433 | 0.987730476 | 1.017127997 | 0.864481267 | 0.929472382 |
| **IGFBP2** | 1.80281664 | 1.87271596 | 0.576046608 | 1.017572018 | 0.934195161 | 0.92904014 |
| **HSP27_pS82** | 1.72735068 | 1.73147702 | 0.994732856 | 1.011014709 | 0.754341591 | 0.776645504 |
| **MSI2** | 1.74282752 | 1.63334885 | 0.96890813 | 1.035961484 | 0.78419669 | 0.754949871 |
| **XBP-1** | 1.78842145 | 1.60328167 | 0.992393714 | 1.013274328 | 0.608953563 | 0.616414305 |
| **IRF-1** | 1.9373607 | 1.84295318 | 0.973742519 | 1.031291447 | 0.609821939 | 0.606045845 |
| **Jak2** | 0.69623381 | 0.68189884 | 0.969136405 | 1.035740969 | 1.850043496 | 1.520802849 |
| **S6_pS235_S236** | 0.43150118 | 0.45657145 | 0.987265034 | 1.018228651 | 2.214268734 | 2.087396262 |
| **MEK1** | 2.1722262 | 2.31307969 | 0.952778346 | 1.023048181 | 0.881653336 | 0.923709254 |
| **Wee1** | 2.39507779 | 2.45482866 | 0.998035322 | 1.007824515 | 0.808276256 | 0.85851111 |
| **cdc25C** | 2.3083811 | 2.40512976 | 0.997820096 | 1.008032424 | 0.861114843 | 0.908834425 |
| **LDHA** | 2.39032055 | 2.31766609 | 0.982534903 | 1.010369771 | 0.839468691 | 0.93605132 |
| **Cyclin-B1** | 3.03042353 | 3.05846575 | 0.909923328 | 1.066376539 | 0.904442679 | 0.881530344 |
| **ACC_pS79** | 2.87526877 | 3.09970187 | 0.974692996 | 1.030373283 | 0.902950159 | 0.907646344 |
| **PLK1** | 3.22267075 | 3.23460335 | 0.94992282 | 1.05430136 | 0.768919456 | 0.872641601 |
| **TRIM25** | 2.47867311 | 3.23886427 | 0.975716804 | 1.004656506 | 0.863081462 | 0.941613019 |
| **AR** | 3.22636431 | 3.44988141 | 0.978135676 | 1.027047642 | 0.710262755 | 0.184126953 |
| **ACC1** | 2.78707071 | 3.9232403 | 0.99268569 | 1.011328954 | 0.924269643 | 0.935117583 |
| **PARP** | 0.91295788 | 0.94417033 | 1.00938308 | 0.996862541 | 1.102181842 | 1.068976847 |

| | | | | | | |
|---|---|---|---|---|---|---|
| **Smad1** | 0.88383979 | 0.92490696 | 1.023059319 | 0.983651246 | 1.070330482 | 1.077408908 |
| **IGF1R_pY1135_Y1136** | 0.87181429 | 0.87908299 | 1.013522189 | 0.992864147 | 1.042733976 | 1.064873886 |
| **Heregulin** | 0.8152416 | 0.83834345 | 1.016445824 | 0.990039906 | 1.032827856 | 0.996696536 |
| **Stathmin-1** | 0.81236588 | 0.82729125 | 1.009117295 | 0.99711929 | 1.034629652 | 1.005454378 |
| **HER3** | 0.85047242 | 0.84900981 | 1.012040387 | 0.994295572 | 1.056445599 | 1.032556184 |
| **Bax** | 0.83196745 | 0.84097896 | 1.056352372 | 0.967364161 | 1.021167506 | 1.007599506 |
| **PKC-delta_pS664** | 0.8576418 | 0.88654681 | 1.016072004 | 0.992994983 | 1.006768468 | 1.000088161 |
| **GSK-3a-b_pS21_S9** | 0.93452198 | 0.94466179 | 1.045347525 | 0.982223769 | 1.006062327 | 1.016710646 |
| **p27-Kip-1** | 0.95719588 | 0.94664962 | 1.00946849 | 0.996780035 | 1.185374391 | 1.151806234 |
| **Notch1** | 0.93806647 | 0.93075214 | 1.006709058 | 0.999445655 | 1.07989667 | 1.147507273 |
| **PRAS40_pT246** | 0.95548834 | 0.90359065 | 1.017713339 | 0.988815481 | 1.127249245 | 1.089536854 |
| **LC3A-B** | 0.86218011 | 0.89329774 | 1.030566669 | 0.976399119 | 1.236047628 | 1.119786657 |
| **D-a-Tubulin** | 0.73832928 | 0.74374322 | 1.00998343 | 0.996282601 | 1.032285862 | 1.029152648 |
| **Paxillin** | 0.75868597 | 0.78356299 | 1.017670832 | 0.988856544 | 1.067515793 | 1.017100855 |
| **C-Raf** | 0.80554494 | 0.80746598 | 1.006623691 | 0.99952812 | 1.052653887 | 1.019291358 |
| **HER3_pY1289** | 0.72321021 | 0.72756075 | 1.009840471 | 0.996420699 | 1.1470054 | 1.077255872 |
| **PI3K-p110-a** | 0.70049792 | 0.71051547 | 1.01283046 | 0.993532359 | 1.128303371 | 1.085744646 |
| **EGFR** | 0.6281988 | 0.75124003 | 1.044143528 | 0.999684365 | 0.988313176 | 1.168826949 |
| **VASP** | 0.58705097 | 0.5725404 | 1.015191268 | 0.991251811 | 1.010931431 | 0.986113952 |
| **14-3-3-zeta** | 0.64104989 | 0.64931598 | 1.006774319 | 0.999382613 | 1.087846632 | 1.032783008 |
| **B-Raf_pS445** | 0.58298146 | 0.58125215 | 1.017244845 | 0.989268048 | 1.173747582 | 1.099597722 |
| **VEGFR-2** | 0.38303018 | 0.411461 | 1.007791646 | 0.998399871 | 1.089040985 | 1.020855637 |
| **P-Met_pY1234_Y1235** | 0.66840454 | 0.66599255 | 1.006135204 | 1 | 1.257933351 | 1.170684416 |
| **p70-S6K1** | 0.64266595 | 0.67370687 | 1.007947069 | 0.998249733 | 1.321219391 | 1.137181945 |
| **MEK1_pS217_S221** | 0.73936578 | 0.74160832 | 1.025659968 | 0.98113901 | 1.370625972 | 1.289030515 |
| **PEA-15** | 0.63107855 | 0.67331155 | 1.055522417 | 0.95229178 | 1.302403469 | 1.293579084 |
| **Lck** | 0.76591797 | 0.76171113 | 1.021069239 | 0.985573671 | 1.230574385 | 1.164130281 |
| **mTOR_pS2448** | 0.88758077 | 0.86165335 | 1.030857353 | 0.976118318 | 1.408052696 | 1.304668666 |
| **Src_pY527** | 0.83063801 | 0.79768743 | 1.019546507 | 0.987044635 | 1.45075901 | 1.314146669 |
| **HES1** | 0.82170207 | 0.84740104 | 1.037587546 | 0.969616928 | 1.303043835 | 1.356930765 |
| **PKC-b-II_pS660** | 0.75774193 | 0.75972566 | 1.009968927 | 0.996296611 | 1.587102521 | 1.459610881 |
| **Akt_pT308** | 0.97985065 | 0.97378977 | 1.002836779 | 0.991154629 | 1.48776601 | 1.525666823 |
| **NDRG1_pT346** | 0.44375355 | 0.45372687 | 1.010400248 | 0.995879953 | 1.361829125 | 1.32308577 |
| **53BP1** | 0.50820329 | 0.54359348 | 1.00670421 | 0.999450339 | 1.279580172 | 1.199904339 |
| **AMPKa_pT172** | 0.46809512 | 0.42102011 | 1.056181289 | 0.951655307 | 1.231349355 | 1.206760597 |
| **eIF4E** | 0.22779392 | 0.21689893 | 1.010743185 | 0.995548675 | 1.176238745 | 1.149649689 |
| **CDK1** | 0.43290513 | 0.47023242 | 1.021332115 | 0.985319731 | 1.528717572 | 1.527679642 |
| **PKA-a** | 0.43940353 | 0.46132892 | 1.013349829 | 0.993030648 | 1.700699273 | 1.570745086 |
| **YAP** | 1.15647243 | 1.17140605 | 1.01185006 | 0.99447943 | 0.690007364 | 0.702134728 |
| **MSH6** | 1.12205509 | 1.11771275 | 1.010053293 | 0.996215113 | 0.652079348 | 0.666789391 |
| **PLC-gamma2_pY759** | 1.06584587 | 1.0651898 | 1.000512936 | 0.968056824 | 0.998268854 | 1.023048952 |
| **Tuberin_pT1462** | 1.08322259 | 1.08530311 | 1.021236395 | 0.985412197 | 0.95482691 | 0.939779936 |
| **Glutamate-D1-2** | 1.08083284 | 1.0843439 | 1.013429101 | 0.99295407 | 0.912080895 | 0.949331799 |
| **Aurora-B** | 1.21754628 | 1.2138738 | 1.028993273 | 0.977919026 | 0.827016391 | 0.822801035 |
| **Bid** | 1.44297588 | 1.52104345 | 1.009240354 | 0.997000415 | 0.960585644 | 0.933489166 |
| **SOD2** | 1.35568744 | 1.34334497 | 1.002407618 | 0.992953837 | 0.961651354 | 0.95300523 |
| **Atg3** | 1.46782406 | 1.38233375 | 1.259718136 | 0.713554939 | 0.730822286 | 0.735007757 |
| **G6PD** | 1.68461864 | 1.68312461 | 1.023825537 | 0.982911077 | 0.868530428 | 0.272988334 |
| **PTEN** | 0.69246563 | 0.68669996 | 1.008776518 | 0.997448482 | 1.933228981 | 1.887037303 |
| **PDK1_pS241** | 0.65096201 | 0.62488709 | 1.010385413 | 0.995894284 | 1.749732821 | 1.451804865 |
| **Caspase-7-cleaved** | 0.78213752 | 0.75645176 | 1.007914053 | 0.998281626 | 2.992213468 | 2.948743874 |
| **SLC1A5** | 2.08267382 | 2.15468981 | 1.012438421 | 0.993911071 | 0.826674123 | 0.869362929 |
| **DUSP4** | 2.07813915 | 2.1901166 | 1.014485049 | 0.991934021 | 0.772335203 | 0.818569158 |
| **FAK_pY397** | 1.97356026 | 1.97105416 | 1.015614741 | 0.990842735 | 0.894339356 | 0.895497381 |
| **RIP** | 2.47208517 | 2.55730423 | 1.022530654 | 0.984161939 | 0.868499999 | 0.905884974 |
| **Myosin-IIa_pS1943** | 2.35315314 | 2.29383514 | 1.03591129 | 0.971236197 | 0.939285966 | 0.945412377 |
| **TFAM** | 2.28365585 | 2.28492934 | 1.012937337 | 0.977721101 | 0.962318241 | 0.967074643 |
| **Myosin-Heavy-Chain-11** | 2.49446961 | 2.42168402 | 1.00633978 | 0.999802379 | 0.14547361 | 0.130406315 |
| **TFRC** | 2.87521756 | 2.91972729 | 1.01415684 | 0.992251072 | 0.843663481 | 0.853234588 |
| **FASN** | 3.7487033 | 3.5803249 | 1.008802581 | 0.997423305 | 0.727719767 | 0.740005739 |
| **Axl** | 5.85303227 | 6.56876227 | 1.035414296 | 0.968184937 | 0.974049945 | 0.945937837 |
| **Rab11** | 0.95616478 | 0.97359004 | 0.990614611 | 0.993613346 | 1.007739026 | 1.021239506 |
| **COG3** | 0.98699194 | 0.98134479 | 0.997257479 | 0.983545408 | 1.018971577 | 1.042228133 |

| | | | | | | |
|---|---|---|---|---|---|---|
| **TAZ** | 1.0146941 | 1.02221303 | 0.802434841 | 0.783993784 | 1.453749147 | 1.379186863 |
| **Akt_pS473** | 1 | 1.03576278 | 0.715535417 | 0.716052287 | 1.410149815 | 1.426473848 |
| **Granzyme-B** | 1.02178699 | 1.01567252 | 0.851115549 | 0.819017858 | 1.352741278 | 1.267926546 |
| **TUFM** | 1.0066442 | 1.02963602 | 0.74006413 | 0.717169716 | 1.300140216 | 1.2590373 |
| **ER-a_pS118** | 1.0178195 | 1.01933103 | 0.835375613 | 0.930202334 | 1.4257499 | 1.365625596 |
| **U-Histone-H2B** | 1.05196614 | 0.98784366 | 0.797113927 | 0.898686068 | 1.385722131 | 1.394328432 |
| **c-Abl** | 1.04326834 | 1.08023288 | 0.962836349 | 0.96281468 | 1.019583975 | 1.013283829 |
| **Rad50** | 1.06648839 | 1.06375539 | 0.94582001 | 0.924384698 | 0.997662257 | 1.020446122 |
| **Claudin-7** | 1.07301632 | 1.10046716 | 0.962944257 | 0.975380119 | 0.92250077 | 1.027182092 |
| **JNK2** | 1.09923924 | 1.10987698 | 0.96794635 | 0.921180212 | 0.981037712 | 0.98013126 |
| **SHP-2_pY542** | 1.09447287 | 1.12661828 | 0.89323635 | 0.900769877 | 0.971242525 | 1.004146864 |
| **Src-(phospho-Y416)** | 0.98401374 | 1.06367317 | 0.969659796 | 0.975886008 | 1.012504827 | 1.019946486 |
| **14-3-3-beta** | 0.97442371 | 1.0244141 | 0.924668734 | 0.940167139 | 1.050308777 | 1.00644215 |
| **c-Jun_pS73** | 0.97179907 | 1.09505358 | 0.961279114 | 0.941101232 | 1.047864458 | 1.089759026 |
| **GCLM** | 1.01833059 | 1.04629899 | 0.869703473 | 0.873363873 | 1.043127339 | 1.064634129 |
| **Tyro3** | 1.02049929 | 1.0260504 | 0.889861765 | 0.882504 | 1.041079899 | 1.038708278 |
| **PI3K-p85** | 1.01304438 | 1.02373427 | 0.923358237 | 0.933342392 | 1.121253269 | 1.056914619 |
| **Mnk1** | 0.99721043 | 1.01383574 | 0.969551826 | 0.950821381 | 1.224256833 | 1.207992681 |
| **AMPK-a2_pS345** | 1.02282209 | 1.01348357 | 0.93342689 | 0.951176076 | 1.218417039 | 1.21290479 |
| **DM-K9-Histone-H3** | 0.94812436 | 0.99943171 | 0.999301505 | 0.963267423 | 1.239230856 | 1.216169322 |
| **RBM15** | 1.0094201 | 1.0270763 | 0.848069913 | 0.83059644 | 1.166649557 | 1.260223694 |
| **ATM** | 1.02327645 | 0.99567235 | 0.7651204 | 0.969115074 | 1.181806766 | 1.169028815 |
| **b-Catenin** | 1.00768728 | 1.02867417 | 0.715585636 | 0.754566961 | 1.137175395 | 1.083885124 |
| **ULK1_pS757** | 1.07168377 | 1.09251484 | 0.766320368 | 0.760577691 | 0.992757383 | 1.030753818 |
| **Collagen-VI** | 1.26495527 | 1.2556548 | 0.998267253 | 0.98072355 | 0.947394598 | 0.964911063 |
| **LRP6_pS1490** | 1.2476854 | 1.26909208 | 0.979744173 | 0.980415414 | 0.962848903 | 0.965211024 |
| **FoxM1** | 1.24129734 | 1.24959562 | 0.99206759 | 0.994611553 | 0.936760002 | 0.951391492 |
| **eIF4E_pS209** | 1.25538307 | 1.28665702 | 0.951305435 | 0.95639551 | 0.974866028 | 0.986041543 |
| **UBAC1** | 1.12347852 | 1.15070104 | 1 | 0.993209211 | 0.993245075 | 0.952756632 |
| **IRS1** | 1.12929758 | 1.15265787 | 0.968997964 | 0.959254541 | 0.966515402 | 0.985810525 |
| **ATM_pS1981** | 1.15980325 | 1.18171595 | 0.995732634 | 0.983105855 | 0.91628955 | 0.962591957 |
| **MAPK_pT202_Y204** | 1.16994866 | 1.20125015 | 0.930493244 | 0.937910969 | 0.974032228 | 0.986840027 |
| **ZAP-70** | 1.17233396 | 1.17444384 | 0.918193205 | 0.931840668 | 0.998032953 | 0.963855851 |
| **ATR_pS428** | 1.21239917 | 1.24141851 | 0.899000764 | 0.885565724 | 0.995351325 | 0.966423898 |
| **Myt1** | 1.15929089 | 1.21788061 | 0.8643983 | 0.865939282 | 0.973147709 | 0.987687082 |
| **FAK** | 1.2352298 | 1.11826196 | 0.879991236 | 0.960254794 | 0.971782567 | 0.984836808 |
| **Chk2_pT68** | 1.1358238 | 1.10849629 | 0.837819021 | 0.919138909 | 1.002139852 | 0.959922899 |
| **p70-S6K_pT389** | 1.23696259 | 1.28400106 | 0.835313257 | 0.848737763 | 0.986705796 | 0.974703247 |
| **C-Raf_pS338** | 1.26112337 | 1.28760164 | 0.789070019 | 0.826842989 | 0.976914386 | 0.984079942 |
| **Cdc2_pY15** | 1.271923 | 1.28851853 | 0.890532941 | 0.870978196 | 0.992063222 | 0.969572735 |
| **FoxO3a** | 1.51934487 | 1.49581407 | 0.979795378 | 0.986833221 | 0.981895068 | 0.958963474 |
| **p21** | 1.53591374 | 1.58250624 | 0.996124099 | 0.97419196 | 1.002796297 | 0.959294258 |
| **PREX1** | 1.54521088 | 1.6151031 | 0.98278879 | 0.98716597 | 0.932360122 | 0.958639553 |
| **DM-Histone-H3** | 1.53645157 | 1.57426037 | 0.87995061 | 0.849946021 | 0.954781386 | 1.005275501 |
| **Tuberin** | 1.47940305 | 1.4972051 | 0.892988818 | 0.87386753 | 0.956843884 | 1.00330036 |
| **Shc_pY317** | 1.33645901 | 1.39632395 | 0.996374375 | 0.975604842 | 0.916864647 | 0.969893978 |
| **Wee1_pS642** | 1.32693066 | 1.37369501 | 0.954115325 | 0.952997683 | 0.969361285 | 0.991313133 |
| **Stat5a** | 1.37398749 | 1.3676816 | 0.902350378 | 0.895854891 | 0.995494091 | 0.96628718 |
| **STING** | 1.32793823 | 1.44233911 | 0.903387593 | 0.893838122 | 0.951136032 | 1.008766457 |
| **Rb_pS807_S811** | 1.46358008 | 1.53505457 | 0.661937832 | 0.673930401 | 0.982978035 | 0.978273119 |
| **Pdcd4** | 1.44835008 | 1.48096733 | 0.63813397 | 0.654814582 | 0.950313462 | 1.009554187 |
| **p90RSK_pT573** | 1.38105957 | 1.41370876 | 0.746715923 | 0.719633541 | 0.96808216 | 0.99253808 |
| **E-Cadherin** | 1.34270239 | 1.38357239 | 0.549320653 | 0.567493241 | 0.942302296 | 1.01722604 |
| **Rad51** | 1.71138261 | 1.74605694 | 0.96588917 | 0.949404736 | 0.931297854 | 0.995399027 |
| **BRD4** | 1.72023411 | 1.76501665 | 0.944952936 | 0.901068866 | 0.984839312 | 0.976490676 |
| **P-Cadherin** | 1.66347625 | 1.77241588 | 0.989815616 | 0.983258715 | 0.990768229 | 0.962443153 |
| **Hexokinase-II** | 1.67699128 | 1.75294174 | 0.883349804 | 0.882129187 | 1.006660252 | 0.955593961 |
| **Gab2** | 1.0379509 | 1 | 0.985523569 | 0.964756375 | 1.967419315 | 1.913087567 |
| **MERIT40_pS29** | 2.03358923 | 2.17180395 | 0.995793402 | 0.984097513 | 0.879089365 | 0.961626607 |
| **Cox-IV** | 2.34988899 | 2.85726102 | 0.980097619 | 0.976174993 | 0.972476833 | 0.969338952 |
