# Supplementary material for: Mammary-specific expression of Trim24 establishes a mouse model of human metaplastic breast cancer
Source: Nat Commun. 2021 Sep 10;12:5389. doi: 10.1038/s41467-021-25650-z (PMC8433435; doi:10.1038/s41467-021-25650-z)
Supplement: Supplementary file 11 — Dataset 8 [file 41467_2021_25650_MOESM11_ESM.pdf]

Supplementary Table 8: A murine TRIM24 signature and its correlation to human TNBC MpBC patient signature.

Gene ID	Gene Name	p.value	FDR	Num Samples NONMETA	Num Samples META	Mean NONMETA	Mean META	Delta	Log_2 Fold Change	Log_2 Fold Change (MLE)	Direction	Carcinoma	Carcinoma.1	Carcinoma.2	Carcinosarcoma	Carcinosarcoma.1	Carcinosarcoma.2
ENSMUSG00000048616.4	Nog	7.41E-17	3.71E-14	3	3	8	343.3333333	335.3333333	4.497324476	5.004602774	Higher in META	4	10	10	366	268	396
ENSMUSG00000028480.14	Glipr2	2.84E-14	8.09E-12	3	3	863	6167.666667	5304.666667	2.298060284	2.364787371	Higher in META	739	561	1289	6116	3229	9158
ENSMUSG00000038402.2	Foxf2	1.37E-13	3.49E-11	3	3	31.33333333	837.3333333	806	4.021524664	4.464837712	Higher in META	15	13	66	511	754	1247
ENSMUSG00000044303.6	Cdkn2a	2.90E-13	7.15E-11	3	3	2823	34839	32016	2.865736855	3.012449748	Higher in META	3687	1385	3397	22212	16559	65746
ENSMUSG00000035062.13	Zc4h2	5.52E-13	1.28E-10	3	3	76	546.6666667	470.6666667	2.255348808	2.325989156	Higher in META	75	57	96	442	338	860
ENSMUSG00000022324.14	Matn2	5.57E-13	1.28E-10	3	3	924.3333333	6516.333333	5592	2.131384064	2.190469885	Higher in META	976	710	1087	4918	3106	11525
ENSMUSG00000001555.10	Fkbp10	8.91E-13	1.91E-10	3	3	2017	13716.66667	11699.66667	2.261907671	2.334413057	Higher in META	1821	1623	2607	11645	10827	18678
ENSMUSG00000044499.11	Hs3st5	7.79E-12	1.53E-09	3	3	4.666666667	169	164.3333333	3.997320994	4.555844088	Higher in META	2	5	7	141	69	297
ENSMUSG00000026873.9	Phf19	4.88E-11	8.65E-09	3	3	120.3333333	773.6666667	653.3333333	2.132068194	2.204086608	Higher in META	132	75	154	748	443	1130
ENSMUSG00000026207.16	Speg	6.08E-11	1.05E-08	3	3	246	2487	2241	2.751006654	2.915249799	Higher in META	129	164	445	2422	1373	3666
ENSMUSG00000037032.16	Apbb1	2.39E-10	3.82E-08	3	3	211	1547.666667	1336.666667	2.354598715	2.461511751	Higher in META	267	125	241	1461	1155	2027
ENSMUSG00000026069.15	Il1rl1	3.49E-10	5.39E-08	3	3	110	5002.666667	4892.666667	4.031003147	4.728306646	Higher in META	75	57	198	722	1708	12578
ENSMUSG00000026764.15	Kif5c	3.56E-10	5.45E-08	3	3	83.66666667	796.6666667	713	2.766776842	2.951075307	Higher in META	64	50	137	986	553	851
ENSMUSG00000054196.6	Cthrc1	4.54E-10	6.80E-08	3	3	1019.666667	8950	7930.333333	2.530478123	2.669942869	Higher in META	626	586	1847	5603	5589	15658
ENSMUSG00000038319.14	Kcnh2	1.70E-09	2.33E-07	3	3	18.33333333	645	626.6666667	4.132308539	5.013435946	Higher in META	19	5	31	1312	349	274
ENSMUSG00000026637.13	Traf5	2.51E-09	3.35E-07	3	3	235.3333333	1966.333333	1731	2.31422741	2.429764622	Higher in META	168	169	369	1184	864	3851
ENSMUSG00000042604.5	Kcna4	2.67E-09	3.50E-07	3	3	6.333333333	610	603.6666667	4.479883373	5.841013573	Higher in META	1	2	16	172	104	1554
ENSMUSG00000043441.5	Gpr149	3.87E-09	4.94E-07	3	3	3	802.3333333	799.3333333	4.785817981	7.006887188	Higher in META	6	1	2	266	20	2121
ENSMUSG00000073293.4	Nudt10	4.97E-09	6.17E-07	3	3	4.666666667	218.6666667	214	4.256189497	5.409144162	Higher in META	4	3	7	420	169	67
ENSMUSG00000007039.12	Ddah2	1.01E-08	1.17E-06	3	3	1277	6261.666667	4984.666667	1.729752527	1.779896208	Higher in META	1316	708	1807	4530	3615	10640
ENSMUSG00000038523.10	1700003F12Rik	2.18E-08	2.37E-06	3	3	31.33333333	298.6666667	267.3333333	2.702550566	2.92561019	Higher in META	20	24	50	348	224	324
ENSMUSG00000033906.5	Zdhhc15	2.73E-08	2.92E-06	3	3	73.33333333	761	687.6666667	2.845545357	3.115191618	Higher in META	95	39	86	538	898	847
ENSMUSG00000001020.8	S100a4	4.96E-08	5.00E-06	3	3	31	1412.666667	1381.666667	3.824803793	4.696611295	Higher in META	9	29	55	576	183	3479
ENSMUSG00000022261.5	Sdc2	5.88E-08	5.78E-06	3	3	2642.333333	27257	24614.66667	2.610193295	2.823000317	Higher in META	1923	3084	2920	13529	25015	43227
ENSMUSG00000059625.6	Sohlh1	6.16E-08	5.97E-06	3	3	0.333333333	61.33333333	61	4.435137031	7.083032923	Higher in META	1	0	0	34	75	75
ENSMUSG00000028186.14	Uox	1.02E-07	9.51E-06	3	3	144	1394	1250	2.484569689	2.673392711	Higher in META	75	179	178	1077	889	2216
ENSMUSG00000019775.17	Rgs17	1.36E-07	1.24E-05	3	3	25.33333333	700.6666667	675.3333333	3.646503966	4.439073678	Higher in META	3	20	53	1098	243	761
ENSMUSG00000054932.6	Afp	1.41E-07	1.28E-05	3	3	12.66666667	436.6666667	424	3.988673344	5.206943264	Higher in META	7	10	21	158	976	176
ENSMUSG00000031520.6	Vegfc	2.20E-07	1.88E-05	3	3	540.6666667	4600	4059.333333	2.608283106	2.844895099	Higher in META	348	487	787	5351	4699	3750
ENSMUSG00000022996.14	Wnt10b	2.73E-07	2.30E-05	3	3	23.33333333	490	466.6666667	3.244487506	3.774636939	Higher in META	9	10	51	333	105	1032
ENSMUSG00000068923.14	Syt11	3.46E-07	2.83E-05	3	3	357	2192.666667	1835.666667	2.197641047	2.336450369	Higher in META	219	211	641	2055	1830	2693
ENSMUSG00000047181.12	Samd14	6.66E-07	5.14E-05	3	3	607.3333333	2170.666667	1563.333333	1.273456985	1.299602324	Higher in META	607	457	758	1821	1249	3442
ENSMUSG00000070858.12	Gm1673	6.86E-07	5.25E-05	3	3	54	630.6666667	576.6666667	2.861417629	3.226960845	Higher in META	16	54	92	714	522	656
ENSMUSG00000028541.14	B4galt2	6.96E-07	5.30E-05	3	3	552	2156.666667	1604.666667	1.511960534	1.556640212	Higher in META	515	337	804	2009	1465	2996
ENSMUSG00000026674.9	Ddr2	8.13E-07	6.07E-05	3	3	865.3333333	9896	9030.666667	2.641420469	2.920215576	Higher in META	411	702	1483	3154	6429	20105
ENSMUSG00000032561.15	Acpp	8.15E-07	6.07E-05	3	3	316.3333333	5063	4746.666667	2.860879109	3.231436407	Higher in META	115	304	530	3492	1056	10641
ENSMUSG00000047878.12	A4galt	9.92E-07	7.21E-05	3	3	310.6666667	1466.333333	1155.666667	1.806406709	1.887122207	Higher in META	233	204	495	1274	1188	1937
ENSMUSG00000027254.13	Map1a	1.14E-06	8.16E-05	3	3	275	2519	2244	2.594192426	2.865069941	Higher in META	99	263	463	2924	1961	2672
ENSMUSG00000046814.3	Gchfr	1.33E-06	9.23E-05	3	3	210.6666667	1236	1025.333333	1.854762549	1.944974421	Higher in META	133	199	300	918	629	2161
ENSMUSG00000038916.7	Soga3	1.33E-06	9.23E-05	3	3	0	43.33333333	43.33333333	4.196644242	7.594470996	Higher in META	0	0	0	28	56	46
ENSMUSG00000029223.12	Uchl1	1.42E-06	9.67E-05	3	3	55	1498.666667	1443.666667	3.573588835	4.553500233	Higher in META	54	68	43	2608	1686	202
ENSMUSG00000042842.11	Serpinb6b	1.44E-06	9.76E-05	3	3	171.3333333	1961.666667	1790.333333	2.548276078	2.808887141	Higher in META	72	144	298	1238	571	4076
ENSMUSG00000029718.14	Pcolce	1.52E-06	0.000102083	3	3	3672	25501.66667	21829.66667	2.239678643	2.408634712	Higher in META	2651	2524	5841	11417	24725	40363
ENSMUSG00000034118.15	Tpst1	1.52E-06	0.000102083	3	3	882	3222.666667	2340.666667	1.407024901	1.445206664	Higher in META	852	601	1193	3068	2225	4375
ENSMUSG00000042817.15	Flt3	1.55E-06	0.00010355	3	3	84.33333333	957.3333333	873	2.860651591	3.257163092	Higher in META	48	20	185	1276	428	1168
ENSMUSG00000029185.14	Fam114a1	1.60E-06	0.000106397	3	3	1946.666667	6554.666667	4608	1.198277438	1.221605918	Higher in META	1749	1456	2635	5116	3895	10653
ENSMUSG00000033065.13	Pfkm	1.76E-06	0.000114948	3	3	1238	6671.666667	5433.666667	1.82457668	1.912349154	Higher in META	1759	664	1291	5325	4144	10546
ENSMUSG00000066456.14	Hmgn3	1.79E-06	0.000116069	3	3	279.3333333	1647	1367.666667	2.148310761	2.297717419	Higher in META	218	122	498	1266	1507	2168
ENSMUSG00000028167.15	Bdh2	2.24E-06	0.000140055	3	3	53.66666667	555.3333333	501.6666667	2.819680369	3.212332273	Higher in META	30	27	104	260	717	689
ENSMUSG00000004837.2	Grap	2.26E-06	0.000141003	3	3	215.3333333	998.3333333	783	1.737088664	1.813941437	Higher in META	168	173	305	1066	704	1225
ENSMUSG00000032625.14	Thsd7a	2.27E-06	0.000141003	3	3	82.33333333	842.3333333	760	2.816741612	3.208085224	Higher in META	36	34	177	468	977	1082
ENSMUSG00000023827.8	Agpat4	2.40E-06	0.00014741	3	3	395.6666667	2424.666667	2029	1.97395898	2.090598681	Higher in META	269	458	460	2309	1676	3289
ENSMUSG00000028214.13	Gem	2.51E-06	0.000152432	3	3	177.3333333	2291.666667	2114.333333	2.531653368	2.801763618	Higher in META	146	156	230	916	530	5429
ENSMUSG00000021318.15	Gli3	2.72E-06	0.000165052	3	3	318	1800.333333	1482.333333	1.783212325	1.868208202	Higher in META	423	204	327	1145	949	3307
ENSMUSG00000063605.5	Ccdc102a	3.20E-06	0.000189145	3	3	594.6666667	2704.333333	2109.666667	1.717778766	1.794400371	Higher in META	578	480	726	2276	2434	3403
ENSMUSG00000027994.14	Mcub	4.21E-06	0.00024002	3	3	173.3333333	2054.666667	1881.333333	2.508043589	2.785151596	Higher in META	74	250	196	1570	804	3790
ENSMUSG00000035385.5	Ccl2	4.23E-06	0.00024002	3	3	728.6666667	5848	5119.333333	2.175188715	2.345154693	Higher in META	391	583	1212	2599	2803	12142
ENSMUSG00000032101.6	Ddx25	4.43E-06	0.000250601	3	3	14.66666667	171.3333333	156.6666667	2.962110484	3.478832239	Higher in META	13	4	27	179	203	132
ENSMUSG00000025037.6	Maoa	5.18E-06	0.000288311	3	3	552	3803.333333	3251.333333	2.06009115	2.204993072	Higher in META	766	204	686	2915	1596	6899
ENSMUSG00000038119.15	Cdon	5.47E-06	0.000303713	3	3	662	2884.666667	2222.666667	1.689900495	1.766583982	Higher in META	544	514	928	2549	2560	3545
ENSMUSG00000001642.18	Akr1b3	5.59E-06	0.0003076	3	3	2043	40926.33333	38883.33333	2.922279436	3.424961237	Higher in META	1788	1010	3331	10838	5272	106669
ENSMUSG00000017969.13	Ptgis	5.59E-06	0.0003076	3	3	82	437.6666667	355.6666667	1.822678947	1.920768635	Higher in META	52	69	125	310	300	703
ENSMUSG00000024041.8	Cryaa	5.82E-06	0.000315426	3	3	7.333333333	224	216.6666667	3.494073106	4.626550416	Higher in META	2	5	15	24	273	375
ENSMUSG00000032532.7	Cck	6.37E-06	0.000336561	3	3	38.66666667	1105	1066.333333	3.547220482	4.776112185	Higher in META	4	16	96	2520	347	448
ENSMUSG00000059003.12	Grin2a	6.55E-06	0.000344994	3	3	14.33333333	453.6666667	439.3333333	3.704013004	5.323851837	Higher in META	4	5	34	204	1084	73
ENSMUSG00000035934.16	Pknox2	6.69E-06	0.000348379	3	3	78.66666667	388.6666667	310	1.805146003	1.901996994	Higher in META	91	46	99	301	323	542
ENSMUSG00000002104.11	Rapsn	6.72E-06	0.000348916	3	3	120.3333333	629.6666667	509.3333333	1.909609786	2.025657967	Higher in META	124	48	189	657	389	843
ENSMUSG00000027797.15	Dclk1	6.93E-06	0.000357191	3	3	1100	3764.333333	2664.333333	1.196275006	1.222857767	Higher in META	981	903	1416	3201	2086	6006
ENSMUSG00000048988.8	Elfn1	7.35E-06	0.00037602	3	3	30.33333333	347.6666667	317.3333333	2.723533357	3.124153264	Higher in META	43	16	32	110	392	541
ENSMUSG00000068522.4	Aard	7.53E-06	0.000384099	3	3	1	154	153	3.957197543	7.60478651	Higher in META	0	0	3	110	344	8
ENSMUSG00000040591.18	1110051M20Rik	8.57E-06	0.000434173	3	3	293	1206.666667	913.6666667	1.507933062	1.563844671	Higher in META	221	192	466	838	784	1998
ENSMUSG00000073650.8	Catip	8.60E-06	0.000434173	3	3	16.66666667	232	215.3333333	2.825019203	3.295532104	Higher in META	3	13	34	216	95	385
ENSMUSG00000024238.13	Zeb1	8.90E-06	0.000445904	3	3	729.3333333	4184	3454.666667	1.802574862	1.901853228	Higher in META	429	770	989	3461	2078	7013
ENSMUSG00000020814.13	Mxra7	9.66E-06	0.000473024	3	3	580.6666667	3244	2663.333333	1.916785241	2.039161855	Higher in META	325	587	830	3505	2185	4042

ENSMUSG00000019929.16	Dcn	9.70E-06	0.000473024	3	3	6283.333333	56166.66667	49883.33333	2.487603812	2.784380753	Higher in META	1941	2828	14081	33898	35763	98839
ENSMUSG00000020805.14	Slc13a5	9.73E-06	0.000473024	3	3	2.666666667	74.66666667	72	3.44399768	4.644284437	Higher in META	5	0	3	42	105	77
ENSMUSG00000022679.13	Mpv17l	1.04E-05	0.0005014	3	3	77.33333333	348.6666667	271.3333333	1.551357475	1.613897492	Higher in META	73	63	96	327	173	546
ENSMUSG00000027220.2	Syt13	1.18E-05	0.000553514	3	3	214.3333333	5379	5164.666667	2.987108733	3.598982679	Higher in META	250	286	107	3738	479	11920
ENSMUSG00000063632.6	Sox11	1.18E-05	0.000553514	3	3	89	1600.333333	1511.333333	3.182101458	3.984914468	Higher in META	21	11	235	627	1416	2758
ENSMUSG00000027811.9	4930579G24Rik	1.23E-05	0.000573617	3	3	192.6666667	914	721.3333333	1.794236279	1.895637081	Higher in META	132	150	296	1017	678	1047
ENSMUSG00000017314.12	Mpp2	1.41E-05	0.000653	3	3	161	732.6666667	571.6666667	1.664233567	1.745159725	Higher in META	139	152	192	677	587	934
ENSMUSG00000021384.14	Susd3	1.44E-05	0.000658799	3	3	163	635.6666667	472.6666667	1.480696131	1.536564678	Higher in META	136	126	227	541	484	882
ENSMUSG00000017466.9	Timp2	1.69E-05	0.000754061	3	3	10162.66667	63468.33333	53305.66667	1.892136292	2.017069955	Higher in META	6989	11855	11644	69744	26882	93779
ENSMUSG00000054423.13	Cadps	1.73E-05	0.000765268	3	3	5.666666667	154	148.3333333	3.454427916	4.817127514	Higher in META	4	6	7	57	362	43
ENSMUSG00000035376.9	Hacd2	1.74E-05	0.000767917	3	3	1269.666667	7077	5807.333333	2.070010965	2.239050618	Higher in META	1232	992	1585	8036	7540	5655
ENSMUSG00000033436.13	Armcx2	1.76E-05	0.000777203	3	3	1254.666667	5769.333333	4514.666667	1.649990194	1.730525051	Higher in META	1618	540	1606	4943	3365	9000
ENSMUSG00000050288.6	Fzd2	1.94E-05	0.000845654	3	3	1191	4186	2995	1.223485253	1.255211861	Higher in META	1194	775	1604	3615	1956	6987
ENSMUSG00000039103.12	Nexn	2.28E-05	0.000963822	3	3	331.6666667	1517.333333	1185.666667	1.65657884	1.740629602	Higher in META	187	256	552	1380	911	2261
ENSMUSG00000009731.4	Kcnd1	2.35E-05	0.000987253	3	3	129.6666667	664.6666667	535	1.905591207	2.038728572	Higher in META	79	85	225	487	607	900
ENSMUSG00000027834.15	Serpini1	2.60E-05	0.001074948	3	3	27	151.3333333	124.3333333	1.979886889	2.133108527	Higher in META	22	15	44	144	113	197
ENSMUSG00000007950.9	Abhd8	2.65E-05	0.001094484	3	3	1029.333333	4339.666667	3310.333333	1.410088893	1.461330807	Higher in META	917	967	1204	2786	2662	7571
ENSMUSG00000007908.14	Hmgcll1	2.74E-05	0.00112574	3	3	14.33333333	224.3333333	210	3.072948579	3.876384163	Higher in META	11	5	27	483	117	73
ENSMUSG00000028031.6	Dkk2	3.13E-05	0.001274971	3	3	303.6666667	6356.333333	6052.666667	3.039435309	3.818405335	Higher in META	149	393	369	441	7032	11596
ENSMUSG00000028989.3	Angptl7	3.21E-05	0.001297885	3	3	12.33333333	241	228.6666667	2.842200565	3.432654337	Higher in META	11	7	19	48	65	610
ENSMUSG00000034520.14	Gjc1	3.40E-05	0.001358876	3	3	394.6666667	1718.333333	1323.666667	1.691390904	1.785738557	Higher in META	426	256	502	2111	1270	1774
ENSMUSG00000028173.10	Wls	3.56E-05	0.001406077	3	3	5648.666667	29692	24043.33333	1.600304789	1.679643416	Higher in META	4335	5274	7337	17032	13039	59005
ENSMUSG00000082229.1	Nap1l2	3.59E-05	0.001413173	3	3	5.666666667	78	72.33333333	2.840489584	3.447722289	Higher in META	1	4	12	81	53	100
ENSMUSG00000027457.15	Snph	3.83E-05	0.001483807	3	3	7.333333333	268	260.6666667	3.394045899	4.900815825	Higher in META	3	9	10	709	65	30
ENSMUSG00000019539.11	Rcn3	3.92E-05	0.001510477	3	3	1769	12174	10405	2.287598842	2.553033212	Higher in META	593	1429	3285	10148	12984	13390
ENSMUSG00000020186.7	Csrp2	3.95E-05	0.001513651	3	3	1250	8054.333333	6804.333333	1.94123093	2.092404541	Higher in META	806	962	1982	2950	5178	16035
ENSMUSG00000011589.8	Fsd1	4.02E-05	0.001529668	3	3	9	101	92	2.801157931	3.384228591	Higher in META	8	6	13	114	133	56
ENSMUSG00000042182.16	Bend6	4.04E-05	0.001531005	3	3	102.3333333	506.3333333	404	1.831507079	1.956685641	Higher in META	136	56	115	458	453	608
ENSMUSG00000030268.17	Bcat1	4.04E-05	0.001531005	3	3	459.6666667	3640.333333	3180.666667	2.14569928	2.358871599	Higher in META	163	512	704	2116	2142	6663
ENSMUSG00000033565.16	Rbfox2	4.33E-05	0.001592698	3	3	3664.333333	11011	7346.666667	1.054035393	1.075949497	Higher in META	3789	2604	4600	10140	6259	16634
ENSMUSG00000045664.4	Cdc42ep2	4.33E-05	0.001592698	3	3	362	1760	1398	1.667212491	1.760041402	Higher in META	200	351	535	1356	1151	2773
ENSMUSG00000031740.8	Mmp2	4.45E-05	0.001609731	3	3	9951.333333	37381.66667	27430.33333	1.297487608	1.339431615	Higher in META	11511	7277	11066	36172	17734	58239
ENSMUSG00000031149.7	Praf2	4.53E-05	0.001628868	3	3	392.3333333	2045.666667	1653.333333	1.777059409	1.892029333	Higher in META	257	428	492	1418	1751	2968
ENSMUSG00000049436.4	Upk1b	4.59E-05	0.001643975	3	3	6	126	120	3.212474989	4.348543566	Higher in META	3	3	12	19	222	137
ENSMUSG00000024424.14	Ttc39c	5.12E-05	0.001805591	3	3	582	3637	3055	1.922367334	2.073895364	Higher in META	245	624	877	2592	2246	6073
ENSMUSG00000039007.9	Cpq	5.30E-05	0.001861917	3	3	914	3010.666667	2096.666667	1.288141819	1.330036728	Higher in META	907	588	1247	2728	2317	3987
ENSMUSG00000005973.6	Rcn1	5.45E-05	0.001905093	3	3	3232.333333	16030.33333	12798	1.774873592	1.892113729	Higher in META	4736	1665	3296	11263	13963	22865
ENSMUSG00000025151.16	Maged1	5.56E-05	0.001933004	3	3	7804	35085	27281	1.700523762	1.802705101	Higher in META	8948	5273	9191	22504	35995	46756
ENSMUSG00000020695.14	Mrc2	5.64E-05	0.00195115	3	3	2765.333333	15277	12511.66667	1.81658788	1.943730532	Higher in META	1442	2862	3992	9359	11742	24730
ENSMUSG00000031760.9	Mt3	5.66E-05	0.001954463	3	3	21.66666667	278	256.3333333	2.58647927	3.03400742	Higher in META	10	35	20	112	251	471
ENSMUSG00000015053.14	Gata2	5.73E-05	0.0019656	3	3	223	1156	933	1.789321876	1.910552176	Higher in META	263	111	295	563	935	1970
ENSMUSG00000036030.9	Prtg	6.75E-05	0.002264783	3	3	50.66666667	465.3333333	414.6666667	2.377648028	2.708509605	Higher in META	13	28	111	323	206	867
ENSMUSG00000031289.10	Il13ra2	7.38E-05	0.002427796	3	3	73	1507.666667	1434.666667	2.943253375	3.744408562	Higher in META	15	60	144	125	1006	3392
ENSMUSG00000054091.9	1810037I17Rik	7.56E-05	0.002479322	3	3	2155.333333	6944.333333	4789	1.129121851	1.15815427	Higher in META	2222	1479	2765	4690	4510	11633
ENSMUSG00000027500.10	Stmn2	7.96E-05	0.002599908	3	3	306	6306	6000	3.210309095	4.499838656	Higher in META	22	23	873	3034	8895	6989
ENSMUSG00000001281.9	Itgb7	8.08E-05	0.00262621	3	3	276	2342.333333	2066.333333	2.283056936	2.57659201	Higher in META	178	62	588	1273	915	4839
ENSMUSG00000035373.2	Ccl7	8.15E-05	0.002637908	3	3	741.3333333	4237.666667	3496.333333	1.838818299	1.978096207	Higher in META	358	534	1332	2168	2551	7994
ENSMUSG00000002732.14	Fkbp7	8.26E-05	0.002662479	3	3	459	1966.666667	1507.666667	1.594004982	1.681361681	Higher in META	250	367	760	1719	1379	2802
ENSMUSG00000005667.8	Mthfd2	8.30E-05	0.002670237	3	3	797.3333333	3422.333333	2625	1.462631657	1.528945394	Higher in META	1089	507	796	2341	2120	5806
ENSMUSG00000029659.16	Medag	8.83E-05	0.002808002	3	3	550	4500	3950	2.371566174	2.714100265	Higher in META	169	442	1039	1785	4931	6784
ENSMUSG00000026875.14	Traf1	9.04E-05	0.002870896	3	3	614.3333333	2587.666667	1973.333333	1.560728946	1.643305917	Higher in META	379	333	1131	2207	1414	4142
ENSMUSG00000049288.4	Lix1l	9.25E-05	0.002916412	3	3	2165	7886.333333	5721.333333	1.304616276	1.351442556	Higher in META	2535	1791	2169	7108	5355	11196
ENSMUSG00000048489.12	Depp1	9.43E-05	0.00296734	3	3	206.6666667	1157.333333	950.6666667	1.759892144	1.882832975	Higher in META	216	130	274	1240	316	1916
ENSMUSG00000028864.7	Hgf	9.60E-05	0.002994178	3	3	227.3333333	2107.333333	1880	2.262330756	2.5545342	Higher in META	198	123	361	402	1181	4739
ENSMUSG00000073295.4	Nudt11	9.97E-05	0.003070943	3	3	6	130	124	3.209140744	4.615119698	Higher in META	2	2	14	182	193	15
ENSMUSG00000031962.6	Cdh15	0.000100825	0.003092172	3	3	76.66666667	1231.666667	1155	3.113245924	4.267697186	Higher in META	37	45	148	474	3053	168
ENSMUSG00000022887.8	Masp1	0.000107218	0.003240911	3	3	72	522.3333333	450.3333333	1.991801841	2.182276305	Higher in META	74	69	73	608	132	827
ENSMUSG00000003411.10	Rab3b	0.000107918	0.003248052	3	3	12	230	218	2.911193473	3.739783489	Higher in META	3	2	31	64	100	526
ENSMUSG00000032387.15	Rbpms2	0.000113678	0.003414345	3	3	324.6666667	2316.333333	1991.666667	2.334029305	2.670994712	Higher in META	401	178	395	3933	2000	1016
ENSMUSG00000022620.14	Arsa	0.000115084	0.003428225	3	3	1308.666667	5284.666667	3976	1.388780429	1.44756793	Higher in META	1444	1249	1233	4101	3681	8072
ENSMUSG00000055435.6	Maf	0.000115853	0.003444079	3	3	2664.666667	12030	9365.333333	1.73710658	1.858423598	Higher in META	1452	2034	4508	9148	11422	15520
ENSMUSG00000053398.11	Phgdh	0.000118028	0.003494461	3	3	2202	15512.33333	13310.33333	2.000559859	2.196304171	Higher in META	4030	883	1693	11508	7528	27501
ENSMUSG00000024208.15	Uqcc2	0.000122356	0.003600578	3	3	2154.333333	8443.333333	6289	1.479006088	1.551473357	Higher in META	2143	1333	2987	4828	7366	13136
ENSMUSG00000007655.16	Cav1	0.000126054	0.003687457	3	3	18530.33333	326352.6667	307822.3333	2.69852828	3.307095736	Higher in META	43288	3496	8807	170742	81914	726402
ENSMUSG00000025348.9	Itga7	0.00013775	0.00395745	3	3	554.3333333	3921.666667	3367.333333	2.116063118	2.360172125	Higher in META	157	631	875	4037	2600	5128
ENSMUSG00000030638.13	Sh3gl3	0.000138815	0.003980186	3	3	41	454.6666667	413.6666667	2.61670522	3.164465345	Higher in META	8	42	73	169	568	627
ENSMUSG00000025130.11	P4hb	0.000141306	0.004043647	3	3	29378.33333	98522	69143.66667	1.15854411	1.19271482	Higher in META	32427	24770	30938	78647	63853	153066
ENSMUSG00000026728.9	Vim	0.000159866	0.004521359	3	3	25159.66667	169230.3333	144070.6667	1.82233263	1.971723642	Higher in META	14642	33396	27441	118098	70704	318889
ENSMUSG00000034205.16	Loxl2	0.000160744	0.004533954	3	3	3613.333333	18501.66667	14888.33333	1.6984853	1.8167585	Higher in META	1866	4250	4724	16483	12320	26702
ENSMUSG00000028381.8	Ugcg	0.000164614	0.004613755	3	3	2708.333333	9382.666667	6674.333333	1.353932171	1.410957188	Higher in META	2158	2218	3749	9708	7256	11184
ENSMUSG00000028484.16	Psip1	0.000167547	0.004669786	3	3	2626.666667	8629	6002.333333	1.184388627	1.221878642	Higher in META	2573	1483	3824	5724	5514	14649
ENSMUSG00000033491.13	Prss35	0.000167684	0.004669786	3	3	80.66666667	1299.333333	1218.666667	2.937611729	3.907063567	Higher in META	16	50	176	3202	394	302
ENSMUSG00000014633.15	Cmc2	0.000173169	0.00479496	3	3	504	2508.666667	2004.666667	1.728100935	1.854783826	Higher in META	656	250	606	1194	2133	4199
ENSMUSG00000027805.16	Pfn2	0.000173816	0.004803745	3	3	755	4447.333333	3692.333333	2.16854212	2.445554983	Higher in META	1040	314	911	4593	5748	3001
ENSMUSG00000034783.7	Cd207	0.000187011	0.005119724	3	3	2.333333333	45.33333333	43	2.905651111	3.924702397	Higher in META	1	4	2	57	45	34
ENSMUSG00000039168.15	Dap	0.000197436	0.005384858	3	3	6867.333333	47600.66667	40733.33333	1.797166982	1.944568618	Higher in META	8043	6001	6558	18830	17591	106381

ENSMUSG00000028789.16	Azin2	0.000200287	0.005434126	3	3	546	2527	1981	1.531715473	1.61893358	Higher in META	313	498	827	2295	1062	4224
ENSMUSG00000031960.14	Aars	0.000204199	0.005502591	3	3	4488.666667	14277.33333	9788.666667	1.099055886	1.12959268	Higher in META	5218	3299	4949	10955	9182	22695
ENSMUSG00000029767.16	Calu	0.000204781	0.005502591	3	3	19177.66667	66333.66667	47156	1.211485206	1.252912647	Higher in META	17930	18300	21303	64849	40025	94127
ENSMUSG00000076433.4	Cep295nl	0.000207804	0.005563241	3	3	12.33333333	95.66666667	83.33333333	2.08039728	2.330126398	Higher in META	11	12	14	49	62	176
ENSMUSG00000052584.9	Serp2	0.000215432	0.005714833	3	3	35.33333333	280	244.6666667	2.146925447	2.425428704	Higher in META	10	35	61	166	172	502
ENSMUSG00000038463.8	Olfml2b	0.000218407	0.005772697	3	3	1314.333333	8101.333333	6787	2.185181721	2.481769169	Higher in META	725	1264	1954	6517	11965	5822
ENSMUSG00000039601.15	Rcan2	0.000222547	0.005860803	3	3	68.66666667	455	386.3333333	2.202589056	2.509690856	Higher in META	31	61	114	258	607	500
ENSMUSG00000003420.8	Fcgrt	0.000233629	0.006064756	3	3	2409	8397.666667	5988.666667	1.274610323	1.324185296	Higher in META	1751	1676	3800	8695	4052	12446
ENSMUSG00000001870.15	Ltbp1	0.000234611	0.006079386	3	3	4110.666667	14140	10029.33333	1.362348642	1.42363099	Higher in META	3313	1955	7064	12743	9501	20176
ENSMUSG00000025169.6	Ogfod3	0.000235564	0.006082404	3	3	247.3333333	783.6666667	536.3333333	1.137930663	1.172665454	Higher in META	203	162	377	602	473	1276
ENSMUSG00000034220.7	Gpc1	0.000253427	0.006486043	3	3	4612.333333	24489	19876.66667	1.686467609	1.810559897	Higher in META	2278	5606	5953	19280	15253	38934
ENSMUSG00000074793.10	Hspa12b	0.000257704	0.006583901	3	3	298.3333333	1168	869.6666667	1.574680279	1.673956426	Higher in META	227	201	467	1451	870	1183
ENSMUSG00000014813.8	Stc1	0.000266169	0.006764507	3	3	505.3333333	2912	2406.666667	2.01327178	2.242387237	Higher in META	253	447	816	1440	3586	3710
ENSMUSG00000031990.15	Jam3	0.000269894	0.006847209	3	3	133.6666667	929.3333333	795.6666667	2.283088086	2.646889563	Higher in META	60	135	206	1173	1126	489
ENSMUSG00000050447.15	Lypd6	0.000271404	0.006873515	3	3	11	280.6666667	269.6666667	3.061277717	4.539632494	Higher in META	2	9	22	736	84	22
ENSMUSG00000028811.11	Yars	0.000281174	0.007059451	3	3	1664	5855.666667	4191.666667	1.274582378	1.325575428	Higher in META	1086	1440	2466	4564	3968	9035
ENSMUSG00000056665.2	Them6	0.000293356	0.007339957	3	3	373	1463	1090	1.477471863	1.559788194	Higher in META	442	144	533	1373	879	2137
ENSMUSG00000054408.9	Spcs3	0.000300407	0.007477777	3	3	5055.333333	17717.66667	12662.33333	1.26035593	1.310072865	Higher in META	4363	4795	6008	13104	13913	26136
ENSMUSG00000006958.13	Chrd	0.00031685	0.00774126	3	3	70	480	410	1.888372473	2.077596773	Higher in META	31	69	110	249	211	980
ENSMUSG00000027669.14	Gnb4	0.00031865	0.007772163	3	3	1153.333333	6049.333333	4896	1.684841107	1.813346941	Higher in META	682	1528	1250	5654	4046	8448
ENSMUSG00000038074.16	Fkbp14	0.000321918	0.007838733	3	3	833	2425	1592	1.075939489	1.106520649	Higher in META	713	604	1182	2393	1567	3315
ENSMUSG00000030786.18	Itgam	0.000326132	0.007927612	3	3	985	3074.333333	2089.333333	1.133216956	1.169247252	Higher in META	727	742	1486	2347	2076	4800
ENSMUSG00000054409.4	Tmem74	0.000327352	0.007931197	3	3	5	150.3333333	145.3333333	2.904800828	4.05795856	Higher in META	0	7	8	121	16	314
ENSMUSG00000047344.9	Lancl3	0.000330313	0.007976347	3	3	5.666666667	79.66666667	74	2.780922219	3.674586285	Higher in META	2	1	14	29	96	114
ENSMUSG00000027447.6	Cst3	0.000331914	0.00798931	3	3	13066.66667	42281	29214.33333	1.205164963	1.248999073	Higher in META	10803	11515	16882	35368	33563	57912
ENSMUSG00000045555.3	Mettl24	0.000332105	0.00798931	3	3	28	182.3333333	154.3333333	2.177179497	2.49517531	Higher in META	12	21	51	124	220	203
ENSMUSG00000031780.2	Ccl17	0.000332499	0.00798931	3	3	47.66666667	637.3333333	589.6666667	2.686759443	3.438690217	Higher in META	34	57	52	1511	290	111
ENSMUSG00000046756.10	Mrps7	0.000338295	0.008075188	3	3	1551.666667	4739.333333	3187.666667	1.120422681	1.155390997	Higher in META	1219	1099	2337	3464	3376	7378
ENSMUSG00000028161.17	Ppp3ca	0.000342792	0.008169121	3	3	6385.333333	25111.66667	18726.33333	1.354170601	1.417951581	Higher in META	4983	3915	10258	13187	14356	47792
ENSMUSG00000019831.14	Wasf1	0.000363763	0.008601133	3	3	299.6666667	1110.666667	811	1.387399259	1.457060986	Higher in META	200	279	420	1053	828	1451
ENSMUSG00000032036.15	Kirrel3	0.00038118	0.008894412	3	3	139	909	770	1.965651868	2.190795922	Higher in META	182	143	92	1385	441	901
ENSMUSG00000042812.5	Foxf1	0.000384535	0.008943972	3	3	44.66666667	468.6666667	424	2.415401471	2.90538745	Higher in META	39	38	57	55	510	841
ENSMUSG00000047842.7	Diras2	0.000390517	0.009054133	3	3	17	146.3333333	129.3333333	2.330156155	2.754135393	Higher in META	4	7	40	131	78	230
ENSMUSG00000022048.8	Dpysl2	0.000392946	0.009081486	3	3	1063.333333	4432	3368.666667	1.401182214	1.473973297	Higher in META	1117	1109	964	3104	3259	6933
ENSMUSG00000001864.13	Aif1l	0.000405929	0.00931971	3	3	527.3333333	5228	4700.666667	2.242204962	2.610623262	Higher in META	904	94	584	3616	1209	10859
ENSMUSG00000053647.4	Gper1	0.000406145	0.00931971	3	3	63	424	361	2.256478849	2.634505029	Higher in META	59	27	103	148	645	479
ENSMUSG00000039943.16	Plcb4	0.000406651	0.00931971	3	3	449.3333333	1862	1412.666667	1.542879564	1.643012992	Higher in META	253	440	655	1642	1554	2390
ENSMUSG00000032192.9	Gnb5	0.000406962	0.00931971	3	3	440	1409	969	1.15252336	1.191876305	Higher in META	326	379	615	1284	891	2052
ENSMUSG00000040963.15	Asgr2	0.0004071	0.00931971	3	3	55.66666667	1352.666667	1297	2.934328271	4.230296945	Higher in META	18	81	68	3738	185	135
ENSMUSG00000049036.7	Tmem121	0.000420161	0.009543559	3	3	153.6666667	813	659.3333333	1.776492663	1.937918256	Higher in META	209	134	118	488	825	1126
ENSMUSG00000032456.13	Nmnat3	0.000421685	0.009559887	3	3	151.3333333	574.6666667	423.3333333	1.417131509	1.493629657	Higher in META	108	142	204	485	472	767
ENSMUSG00000053063.11	Clec12a	0.000424447	0.009595929	3	3	300	1850	1550	2.039008705	2.300582819	Higher in META	135	275	490	750	2216	2584
ENSMUSG00000078949.2	R3hdml	0.000439934	0.009823843	3	3	8.666666667	86	77.33333333	2.458171074	3.000382646	Higher in META	3	2	21	62	58	138
ENSMUSG00000035498.10	Cdcp1	6.21E-40	9.02E-36	3	3	4855.666667	125.3333333	4730.333333	5.31878957	5.608241507	Higher in NONMETA	4825	3158	6584	157	91	128
ENSMUSG00000019734.16	Tmc4	2.21E-29	1.60E-25	3	3	3667	132	3535	5.061118771	5.4151667	Higher in NONMETA	3288	3967	3746	161	49	186
ENSMUSG00000054889.9	Dsp	3.93E-28	1.90E-24	3	3	7635	187	7448	5.364261131	5.819011341	Higher in NONMETA	3048	5535	14322	188	83	290
ENSMUSG00000025383.2	Il23a	3.32E-27	1.08E-23	3	3	1259.333333	42.66666667	1216.666667	5.22743077	5.662561791	Higher in NONMETA	1629	1156	993	21	19	88
ENSMUSG00000029149.14	Krtcap3	3.70E-27	1.08E-23	3	3	1511.333333	125.6666667	1385.666667	3.871261764	4.034516038	Higher in NONMETA	1888	950	1696	113	92	172
ENSMUSG00000096215.8	Smim22	2.15E-25	5.22E-22	3	3	404.6666667	21.66666667	383	4.350104639	4.612716571	Higher in NONMETA	433	256	525	23	17	25
ENSMUSG00000061859.16	Patj	1.74E-24	3.61E-21	3	3	2161.333333	212.6666667	1948.666667	3.848903936	4.028981747	Higher in NONMETA	1851	2176	2457	175	87	376
ENSMUSG00000024774.15	Ankrd22	7.22E-23	1.31E-19	3	3	679.3333333	21	658.3333333	5.205255863	5.747017042	Higher in NONMETA	797	551	690	5	12	46
ENSMUSG00000037600.16	Kdf1	1.68E-22	2.71E-19	3	3	1071	36	1035	4.885697096	5.329531704	Higher in NONMETA	997	776	1440	58	12	38
ENSMUSG00000025504.13	Eps8l2	7.81E-22	1.14E-18	3	3	1082.666667	91	991.6666667	3.949265079	4.173527277	Higher in NONMETA	903	747	1598	87	27	159
ENSMUSG00000054065.12	Pkp3	1.15E-21	1.52E-18	3	3	3769.666667	392	3377.666667	3.518214754	3.674517223	Higher in NONMETA	2758	2916	5635	449	240	487
ENSMUSG00000040525.13	Cblc	2.06E-20	2.49E-17	3	3	934.3333333	40.66666667	893.6666667	4.621577706	5.036343533	Higher in NONMETA	760	710	1333	58	10	54
ENSMUSG00000019312.10	Grb7	6.44E-20	7.21E-17	3	3	1746.666667	41.66666667	1705	5.090743313	5.696621505	Higher in NONMETA	1133	1465	2642	74	22	29
ENSMUSG00000057454.5	Lypd3	1.36E-19	1.42E-16	3	3	2370	20.66666667	2349.333333	5.904803942	7.020297221	Higher in NONMETA	420	1215	5475	22	17	23
ENSMUSG00000031995.9	St14	2.54E-19	2.46E-16	3	3	8492.333333	414.6666667	8077.666667	4.383222297	4.753465254	Higher in NONMETA	7763	7166	10548	668	200	376
ENSMUSG00000018166.8	Erbb3	3.38E-19	3.07E-16	3	3	4238.333333	230.3333333	4008	4.402658337	4.781805264	Higher in NONMETA	3357	4896	4462	319	87	285
ENSMUSG00000040728.15	Esrp1	3.89E-19	3.33E-16	3	3	4114.333333	108	4006.333333	4.983649181	5.5732902	Higher in NONMETA	2615	3375	6353	194	49	81
ENSMUSG00000030713.6	Klk7	5.19E-19	4.19E-16	3	3	3166	8.666666667	3157.333333	6.877178948	9.587406635	Higher in NONMETA	792	5667	3039	0	3	23
ENSMUSG00000044393.15	Dsg2	6.50E-19	4.97E-16	3	3	2703.666667	114	2589.666667	4.379924371	4.761452563	Higher in NONMETA	2977	1514	3620	113	132	97
ENSMUSG00000067006.12	Serpinb5	7.27E-19	5.28E-16	3	3	3854	80.33333333	3773.666667	5.216093515	5.928667144	Higher in NONMETA	1040	2479	8043	105	35	101
ENSMUSG00000010760.5	Phlda2	1.11E-18	7.70E-16	3	3	609	39.66666667	569.3333333	4.0104047	4.300213976	Higher in NONMETA	561	285	981	23	36	60
ENSMUSG00000027315.13	Spint1	1.77E-18	1.17E-15	3	3	2169.666667	62.33333333	2107.333333	4.80303058	5.347915732	Higher in NONMETA	2322	1630	2557	93	59	35
ENSMUSG00000022865.13	Cxadr	3.08E-18	1.94E-15	3	3	5553.333333	659.6666667	4893.666667	3.495088618	3.682998795	Higher in NONMETA	6239	2837	7584	555	221	1203
ENSMUSG00000022286.15	Grhl2	3.81E-18	2.31E-15	3	3	2536.333333	250	2286.333333	3.765428706	4.006532155	Higher in NONMETA	3133	1334	3142	117	118	515
ENSMUSG00000029832.16	Nfe2l3	4.35E-18	2.53E-15	3	3	1253.666667	105	1148.666667	3.955796136	4.240824777	Higher in NONMETA	1216	1068	1477	113	26	176
ENSMUSG00000030800.10	Prss8	1.24E-17	6.94E-15	3	3	1345	69.66666667	1275.333333	4.314710068	4.712169975	Higher in NONMETA	1967	866	1202	102	35	72
ENSMUSG00000019996.17	Map7	1.66E-17	8.96E-15	3	3	1149.666667	47.33333333	1102.333333	4.545834471	5.027502451	Higher in NONMETA	1153	718	1578	79	13	50
ENSMUSG00000043088.16	Il17re	4.30E-17	2.23E-14	3	3	832.6666667	71.66666667	761	3.816489941	4.089167117	Higher in NONMETA	733	765	1000	95	25	95
ENSMUSG00000038879.7	Nipal2	8.41E-17	4.07E-14	3	3	1294	136.3333333	1157.666667	3.486446805	3.693209885	Higher in NONMETA	1781	583	1518	136	81	192
ENSMUSG00000032092.5	Mpzl2	2.04E-16	9.59E-14	3	3	584.6666667	20	564.6666667	4.738045345	5.355020132	Higher in NONMETA	942	159	653	22	8	30
ENSMUSG00000050063.18	Klk6	2.73E-16	1.24E-13	3	3	12176	34.33333333	12141.66667	6.510029063	9.486384212	Higher in NONMETA	546	22050	13932	7	13	83
ENSMUSG00000028392.15	Bspry	4.10E-16	1.81E-13	3	3	1500	98	1402	4.015384666	4.364717226	Higher in NONMETA	2175	508	1817	112	49	133
ENSMUSG00000033634.7	Nat8f2	5.08E-16	2.17E-13	3	3	160.6666667	8.333333333	152.3333333	4.244069657	4.684261257	Higher in NONMETA	160	72	250	8	5	12

ENSMUSG00000037279.13	Ovol2	8.57E-16	3.56E-13	3	3	132.6666667	9.333333333	123.3333333	4.038747943	4.409092479	Higher in NONMETA	125	90	183	8	3	17
ENSMUSG00000028572.13	Hook1	1.51E-15	6.11E-13	3	3	1259	144.6666667	1114.333333	3.40506303	3.615519524	Higher in NONMETA	1005	680	2092	156	50	228
ENSMUSG00000003309.14	Ap1m2	3.44E-15	1.35E-12	3	3	2689.333333	81.33333333	2608	4.692540291	5.355038858	Higher in NONMETA	4045	1093	2930	148	37	59
ENSMUSG00000017607.9	Tns4	4.01E-15	1.53E-12	3	3	7485.333333	364.6666667	7120.666667	4.328327231	4.822430358	Higher in NONMETA	3395	8354	10707	558	156	380
ENSMUSG00000045314.5	Sowahb	4.81E-15	1.79E-12	3	3	404.6666667	13	391.6666667	4.73870637	5.454193469	Higher in NONMETA	196	577	441	18	9	12
ENSMUSG00000033590.8	Myo5c	5.51E-15	2.00E-12	3	3	331.6666667	41.33333333	290.3333333	3.366247392	3.578242165	Higher in NONMETA	309	195	491	38	13	73
ENSMUSG00000022900.14	Ildr1	5.66E-15	2.01E-12	3	3	888.6666667	57	831.6666667	3.977204844	4.351442698	Higher in NONMETA	1125	550	991	91	27	53
ENSMUSG00000037995.15	Igsf9	5.87E-15	2.03E-12	3	3	2076	153.6666667	1922.333333	3.848667883	4.182632206	Higher in NONMETA	1641	2518	2069	177	134	150
ENSMUSG00000041782.14	Lad1	7.20E-15	2.43E-12	3	3	9217.333333	222	8995.333333	4.96193005	5.811086821	Higher in NONMETA	3615	15765	8272	245	238	183
ENSMUSG00000021118.7	Plek2	1.08E-14	3.58E-12	3	3	915	152.6666667	762.3333333	3.09587347	3.261416167	Higher in NONMETA	667	797	1281	88	68	302
ENSMUSG00000001672.14	Marveld3	1.21E-14	3.90E-12	3	3	896	28.33333333	867.6666667	4.683611149	5.382130333	Higher in NONMETA	999	359	1330	49	5	31
ENSMUSG00000026638.15	Irf6	1.35E-14	4.27E-12	3	3	2864	111.3333333	2752.666667	4.370644996	4.910373458	Higher in NONMETA	1968	1285	5339	194	50	90
ENSMUSG00000042306.11	S100a14	2.13E-14	6.57E-12	3	3	7418	115.6666667	7302.333333	5.237916516	6.36776392	Higher in NONMETA	957	10156	11141	130	108	109
ENSMUSG00000014791.10	Elmo3	2.40E-14	7.28E-12	3	3	789.6666667	166	623.6666667	2.666588613	2.772580964	Higher in NONMETA	644	586	1139	100	113	285
ENSMUSG00000084128.10	Esrp2	2.79E-14	8.09E-12	3	3	1776.333333	91.66666667	1684.666667	4.268254428	4.778404958	Higher in NONMETA	1858	472	2999	97	22	156
ENSMUSG00000029370.10	Rassf6	2.80E-14	8.09E-12	3	3	384.3333333	62.33333333	322	2.952428457	3.100736691	Higher in NONMETA	449	297	407	64	42	81
ENSMUSG00000036687.13	Tmem184a	3.28E-14	9.18E-12	3	3	529.6666667	22.66666667	507	4.292081701	4.824215862	Higher in NONMETA	638	196	755	37	12	19
ENSMUSG00000023232.17	Serinc2	4.09E-14	1.12E-11	3	3	5813.333333	601.3333333	5212	3.401211063	3.638547432	Higher in NONMETA	4646	5328	7466	826	417	561
ENSMUSG00000035852.11	Misp	7.68E-14	2.07E-11	3	3	619.6666667	53.66666667	566	3.731516721	4.066828826	Higher in NONMETA	698	698	463	37	50	74
ENSMUSG00000074227.12	Spint2	1.17E-13	3.09E-11	3	3	13717.33333	760	12957.33333	4.028542736	4.470636762	Higher in NONMETA	14511	9289	17352	1363	408	509
ENSMUSG00000029154.13	Cwh43	1.36E-13	3.49E-11	3	3	1143.666667	27.33333333	1116.333333	4.923098848	5.885090555	Higher in NONMETA	1442	230	1759	36	3	43
ENSMUSG00000006179.8	Prss16	2.89E-13	7.15E-11	3	3	354.6666667	43.66666667	311	3.253414547	3.477288514	Higher in NONMETA	367	315	382	56	27	48
ENSMUSG00000021751.13	Acox2	4.12E-13	9.99E-11	3	3	268	9.666666667	258.3333333	4.65079575	5.467974478	Higher in NONMETA	486	92	226	8	2	19
ENSMUSG00000010080.15	Epn3	4.83E-13	1.15E-10	3	3	1096.333333	45	1051.333333	4.213569424	4.774593781	Higher in NONMETA	1285	468	1536	74	36	25
ENSMUSG00000023039.16	Krt7	6.31E-13	1.43E-10	3	3	31425.33333	621	30804.33333	4.883440631	5.895004984	Higher in NONMETA	24981	43597	25698	823	843	197
ENSMUSG00000021636.14	Marveld2	6.46E-13	1.44E-10	3	3	852	80	772	3.595235031	3.917593062	Higher in NONMETA	756	779	1021	122	27	91
ENSMUSG00000026994.9	Galnt3	7.06E-13	1.55E-10	3	3	3433.333333	492.3333333	2941	2.93712901	3.102175267	Higher in NONMETA	3843	1700	4757	525	402	550
ENSMUSG00000030431.8	Tmem238	8.75E-13	1.90E-10	3	3	1099.333333	327	772.3333333	2.169986664	2.233648513	Higher in NONMETA	1219	645	1434	268	204	509
ENSMUSG00000031710.4	Ucp1	1.33E-12	2.81E-10	3	3	3563	10.66666667	3552.333333	5.811483301	8.90374133	Higher in NONMETA	3639	26	7024	6	3	23
ENSMUSG00000046826.7	Fam187b	1.48E-12	3.08E-10	3	3	153.3333333	23	130.3333333	3.060122992	3.256624042	Higher in NONMETA	126	127	207	23	12	34
ENSMUSG00000034112.9	Atp2c2	1.67E-12	3.41E-10	3	3	673.3333333	34.33333333	639	4.246246488	4.859016462	Higher in NONMETA	173	574	1273	32	11	60
ENSMUSG00000008601.12	Rab25	4.47E-12	8.91E-10	3	3	1912.666667	70.33333333	1842.333333	4.319523494	5.011034737	Higher in NONMETA	1204	1434	3100	144	31	36
ENSMUSG00000038007.14	Acer2	4.48E-12	8.91E-10	3	3	747	121	626	3.006350413	3.199250694	Higher in NONMETA	673	402	1166	113	36	214
ENSMUSG00000001247.16	Lsr	1.12E-11	2.15E-09	3	3	1777	103.6666667	1673.333333	4.016883324	4.569070164	Higher in NONMETA	1462	1833	2036	194	29	88
ENSMUSG00000032358.12	Fam83b	1.12E-11	2.15E-09	3	3	348.6666667	8	340.6666667	4.857690163	6.070748232	Higher in NONMETA	171	110	765	5	0	19
ENSMUSG00000044641.7	Pard6b	1.63E-11	3.09E-09	3	3	1150.666667	63.33333333	1087.333333	4.108103367	4.724235894	Higher in NONMETA	361	1784	1307	75	43	72
ENSMUSG00000029032.12	Arhgef16	2.36E-11	4.41E-09	3	3	1283	40.66666667	1242.333333	4.330818893	5.108181307	Higher in NONMETA	587	849	2413	77	28	17
ENSMUSG00000015647.9	Lama5	2.85E-11	5.20E-09	3	3	7233	1896	5337	2.488581601	2.60298067	Higher in NONMETA	6671	4737	10291	1064	776	3848
ENSMUSG00000031075.18	Ano1	2.86E-11	5.20E-09	3	3	11377.66667	1675.333333	9702.333333	3.206149794	3.468395103	Higher in NONMETA	7137	13657	13339	1718	534	2774
ENSMUSG00000003051.13	Elf3	3.26E-11	5.85E-09	3	3	1996	81	1915	4.174191661	4.856948789	Higher in NONMETA	3145	385	2458	125	52	66
ENSMUSG00000042724.8	Map3k9	5.36E-11	9.39E-09	3	3	425.3333333	53.66666667	371.6666667	3.160543923	3.420924988	Higher in NONMETA	319	463	494	67	39	55
ENSMUSG00000005251.14	Ripk4	6.46E-11	1.11E-08	3	3	1457	69	1388	3.927315488	4.493288387	Higher in NONMETA	1270	907	2194	100	78	29
ENSMUSG00000044279.15	Crb3	7.38E-11	1.25E-08	3	3	802.3333333	27.66666667	774.6666667	4.294887054	5.107452287	Higher in NONMETA	269	506	1632	53	9	21
ENSMUSG00000027562.12	Car2	9.59E-11	1.60E-08	3	3	5697	572.3333333	5124.666667	3.392138367	3.732058641	Higher in NONMETA	3798	1497	11796	404	279	1034
ENSMUSG00000029381.15	Shroom3	1.44E-10	2.39E-08	3	3	1956.666667	339.6666667	1617	2.711024628	2.875044382	Higher in NONMETA	2057	1098	2715	457	199	363
ENSMUSG00000051431.7	Gpr87	1.77E-10	2.89E-08	3	3	873.6666667	10.66666667	863	5.113265276	7.262859751	Higher in NONMETA	30	1510	1081	8	2	22
ENSMUSG00000024421.16	Lama3	1.79E-10	2.89E-08	3	3	14833.33333	192.3333333	14641	5.008722084	6.856561838	Higher in NONMETA	1426	35668	7406	128	246	203
ENSMUSG00000045394.8	Epcam	3.10E-10	4.91E-08	3	3	41294	1247.666667	40046.33333	4.259109342	5.123735524	Higher in NONMETA	18896	16962	88024	2593	618	532
ENSMUSG00000043430.5	Psapl1	3.21E-10	5.01E-08	3	3	362.3333333	5	357.3333333	4.846508326	6.522429612	Higher in NONMETA	63	137	887	8	0	7
ENSMUSG00000037681.13	Esyt3	4.40E-10	6.66E-08	3	3	639.3333333	41.33333333	598	3.642666718	4.127656735	Higher in NONMETA	304	273	1341	61	26	37
ENSMUSG00000053646.13	Plxnb1	5.27E-10	7.82E-08	3	3	3082.666667	623	2459.666667	2.529002401	2.669443933	Higher in NONMETA	2088	1680	5480	698	346	825
ENSMUSG00000033998.9	Kcnk1	6.00E-10	8.81E-08	3	3	1639	65.66666667	1573.333333	4.138622333	4.949492824	Higher in NONMETA	3062	734	1121	129	34	34
ENSMUSG00000018569.12	Cldn7	6.13E-10	8.90E-08	3	3	4069.666667	185	3884.666667	4.052347116	4.795265297	Higher in NONMETA	4014	3868	4327	407	73	75
ENSMUSG00000020411.2	Nipal4	6.18E-10	8.90E-08	3	3	362.3333333	25.66666667	336.6666667	3.733546245	4.276475456	Higher in NONMETA	238	237	612	41	4	32
ENSMUSG00000061517.8	Sox21	1.19E-09	1.70E-07	3	3	234	10	224	3.933201721	4.655303564	Higher in NONMETA	245	155	302	10	16	4
ENSMUSG00000052566.8	Hook2	1.37E-09	1.93E-07	3	3	2274	711	1563	2.249927175	2.351716504	Higher in NONMETA	2540	1555	2727	383	338	1412
ENSMUSG00000018554.13	Ybx2	1.41E-09	1.97E-07	3	3	80	3.333333333	76.66666667	4.163183597	5.092492813	Higher in NONMETA	68	27	145	2	1	7
ENSMUSG00000040434.16	Large2	1.68E-09	2.32E-07	3	3	2403.666667	33.33333333	2370.333333	4.720847431	6.423810458	Higher in NONMETA	4742	67	2402	54	19	27
ENSMUSG00000038295.14	Atg9b	2.34E-09	3.17E-07	3	3	4738	256.6666667	4481.333333	4.188333675	5.142324679	Higher in NONMETA	742	9188	4284	171	91	508
ENSMUSG00000064023.4	Klk8	2.49E-09	3.35E-07	3	3	8869.333333	176.6666667	8692.666667	4.714383581	6.485010582	Higher in NONMETA	387	20192	6029	100	128	302
ENSMUSG00000041842.15	Fhdc1	2.62E-09	3.46E-07	3	3	1768.333333	419	1349.333333	2.413940894	2.546652877	Higher in NONMETA	2049	755	2501	430	191	636
ENSMUSG00000021338.17	Carmil1	2.70E-09	3.50E-07	3	3	1700.333333	773.3333333	927	1.638855385	1.67799978	Higher in NONMETA	1559	1114	2428	594	409	1317
ENSMUSG00000020782.18	Llgl2	2.96E-09	3.81E-07	3	3	1205	153.6666667	1051.333333	3.158394005	3.487750116	Higher in NONMETA	632	1551	1432	210	81	170
ENSMUSG00000060716.7	Plekhh1	3.97E-09	5.02E-07	3	3	648	111.6666667	536.3333333	2.839864379	3.072902574	Higher in NONMETA	514	544	886	151	33	151
ENSMUSG00000028841.14	Cnksr1	4.47E-09	5.60E-07	3	3	1008.333333	76.66666667	931.6666667	3.494580166	3.983417285	Higher in NONMETA	773	713	1539	150	35	45
ENSMUSG00000018849.6	Wwc1	6.01E-09	7.40E-07	3	3	3861	851	3010	2.772998043	2.993937598	Higher in NONMETA	4800	2867	3916	339	321	1893
ENSMUSG00000001583.13	Tnk1	6.22E-09	7.60E-07	3	3	581.3333333	42.66666667	538.6666667	3.85543033	4.58809857	Higher in NONMETA	526	625	593	47	2	79
ENSMUSG00000020455.16	Trim11	6.72E-09	8.14E-07	3	3	2686.333333	945	1741.333333	1.98992766	2.065799672	Higher in NONMETA	3271	1436	3352	669	508	1658
ENSMUSG00000015970.18	Chdh	7.06E-09	8.48E-07	3	3	618.3333333	54.33333333	564	3.530008441	4.053975059	Higher in NONMETA	641	127	1087	47	12	104
ENSMUSG00000020847.15	Rph3al	7.15E-09	8.52E-07	3	3	272.3333333	52.33333333	220	2.552747417	2.723519949	Higher in NONMETA	240	148	429	63	33	61
ENSMUSG00000024526.8	Cidea	8.22E-09	9.72E-07	3	3	1854	15.66666667	1838.333333	4.78467584	7.228281043	Higher in NONMETA	1368	73	4121	25	0	22
ENSMUSG00000022512.2	Cldn1	9.70E-09	1.14E-06	3	3	3754.666667	263.6666667	3491	3.545228027	4.092120024	Higher in NONMETA	2046	1170	8048	430	76	285
ENSMUSG00000028860.13	Sytl1	9.97E-09	1.16E-06	3	3	937.6666667	85.66666667	852	3.394118473	3.861004781	Higher in NONMETA	303	1103	1407	122	52	83
ENSMUSG00000045659.18	Plekha7	1.10E-08	1.26E-06	3	3	1555.666667	176.3333333	1379.333333	3.222208172	3.610496021	Higher in NONMETA	398	1229	3040	166	84	279
ENSMUSG00000047501.3	Cldn4	1.27E-08	1.44E-06	3	3	10348.33333	520	9828.333333	3.876040363	4.675503975	Higher in NONMETA	8495	15016	7534	981	408	171
ENSMUSG00000041439.15	Mfsd6	1.40E-08	1.58E-06	3	3	2139	555	1584	2.39077286	2.534149558	Higher in NONMETA	1649	2147	2621	647	216	802

ENSMUSG00000070473.4	Cldn3	1.62E-08	1.81E-06	3	3	5365.333333	294.3333333	5071	3.667752331	4.320501374	Higher in NONMETA	5020	4922	6154	426	371	86
ENSMUSG00000027376.15	Prom2	1.71E-08	1.90E-06	3	3	827	77	750	3.270169008	3.69559348	Higher in NONMETA	1071	423	987	139	43	49
ENSMUSG00000018907.10	Alox12e	2.16E-08	2.37E-06	3	3	857.6666667	15	842.6666667	4.393630524	5.979038942	Higher in NONMETA	1174	25	1374	27	8	10
ENSMUSG00000045319.13	Proser2	2.18E-08	2.37E-06	3	3	1073.666667	214.6666667	859	2.824801748	3.082372477	Higher in NONMETA	610	912	1699	119	59	466
ENSMUSG00000005373.13	Mlxipl	2.45E-08	2.63E-06	3	3	585.6666667	49.66666667	536	3.390725414	3.891225529	Higher in NONMETA	187	251	1319	60	19	70
ENSMUSG00000057092.12	Fxyd3	2.95E-08	3.13E-06	3	3	1789.666667	63	1726.666667	4.013060408	5.032275843	Higher in NONMETA	2146	211	3012	129	16	44
ENSMUSG00000029999.14	Tgfa	3.27E-08	3.44E-06	3	3	4876.666667	482.6666667	4394	3.515271449	4.102919139	Higher in NONMETA	1530	7199	5901	557	100	791
ENSMUSG00000024186.15	Rgs11	3.36E-08	3.52E-06	3	3	374	68	306	2.785239028	3.038887524	Higher in NONMETA	182	243	697	42	28	134
ENSMUSG00000001622.15	Csn3	3.45E-08	3.58E-06	3	3	59790.66667	1081	58709.66667	4.402535634	6.103453709	Higher in NONMETA	113475	1550	64347	2053	274	916
ENSMUSG00000027111.15	Itga6	3.67E-08	3.79E-06	3	3	20765.33333	4580.666667	16184.66667	2.707122856	2.9392105	Higher in NONMETA	29506	20262	12528	3881	1755	8106
ENSMUSG00000026147.16	Col9a1	4.29E-08	4.39E-06	3	3	6602	62.66666667	6539.333333	4.655666121	7.482844951	Higher in NONMETA	16330	48	3428	52	8	128
ENSMUSG00000040714.14	Klc3	4.59E-08	4.66E-06	3	3	958.6666667	201.3333333	757.3333333	2.517896139	2.703187387	Higher in NONMETA	429	846	1601	194	123	287
ENSMUSG00000079330.8	Lemd1	5.08E-08	5.10E-06	3	3	187.6666667	16	171.6666667	3.723405653	4.494645449	Higher in NONMETA	311	118	134	4	3	41
ENSMUSG00000067889.4	Sptbn2	5.38E-08	5.36E-06	3	3	1470.333333	108	1362.333333	3.469653662	4.055915325	Higher in NONMETA	2595	291	1525	166	63	95
ENSMUSG00000044433.16	Camsap3	5.47E-08	5.41E-06	3	3	507.6666667	36.33333333	471.3333333	3.383836563	3.919984092	Higher in NONMETA	381	182	960	64	24	21
ENSMUSG00000006154.13	Eps8l1	5.98E-08	5.84E-06	3	3	428.6666667	32.66666667	396	3.315615303	3.817177672	Higher in NONMETA	368	192	726	53	29	16
ENSMUSG00000009633.3	G0s2	7.72E-08	7.43E-06	3	3	2039.666667	469.3333333	1570.333333	2.571636048	2.778369283	Higher in NONMETA	1242	2149	2728	499	140	769
ENSMUSG00000024903.10	Lao1	7.92E-08	7.58E-06	3	3	135.6666667	9	126.6666667	3.713327619	4.518839749	Higher in NONMETA	100	59	248	9	0	18
ENSMUSG00000038167.6	Plekhg6	8.04E-08	7.64E-06	3	3	536	66.33333333	469.6666667	3.049128256	3.423798642	Higher in NONMETA	205	240	1163	66	25	108
ENSMUSG00000035818.13	Plekhs1	8.18E-08	7.72E-06	3	3	2437	37.33333333	2399.666667	4.430265667	6.531069377	Higher in NONMETA	4676	20	2615	47	8	57
ENSMUSG00000020159.8	Gabrp	9.38E-08	8.80E-06	3	3	1780.333333	28	1752.333333	4.34411589	6.214966458	Higher in NONMETA	1691	19	3631	45	8	31
ENSMUSG00000030494.13	Rhpn2	1.16E-07	1.08E-05	3	3	656.6666667	115.3333333	541.3333333	2.82979585	3.12597279	Higher in NONMETA	732	253	985	104	24	218
ENSMUSG00000026822.14	Lcn2	1.19E-07	1.10E-05	3	3	127293.6667	1718.666667	125575	4.394956009	6.53155308	Higher in NONMETA	289220	1275	91386	3023	715	1418
ENSMUSG00000022297.14	Fzd6	1.20E-07	1.10E-05	3	3	2039	902.6666667	1136.333333	1.701811147	1.758197335	Higher in NONMETA	2202	1242	2673	573	487	1648
ENSMUSG00000038980.1	Rbbp8nl	1.59E-07	1.43E-05	3	3	97.66666667	2.666666667	95	4.053098686	5.480656079	Higher in NONMETA	115	38	140	7	0	1
ENSMUSG00000019039.13	Dalrd3	1.62E-07	1.45E-05	3	3	1534.333333	526.3333333	1008	1.868426984	1.945918466	Higher in NONMETA	1072	1113	2418	538	343	698
ENSMUSG00000021638.12	Ocln	1.69E-07	1.50E-05	3	3	887.3333333	77	810.3333333	3.514268224	4.206093386	Higher in NONMETA	432	1074	1156	113	7	111
ENSMUSG00000040350.16	Trim7	1.75E-07	1.55E-05	3	3	342	100.6666667	241.3333333	2.351671169	2.515441333	Higher in NONMETA	308	376	342	75	38	189
ENSMUSG00000045180.13	Shroom2	1.89E-07	1.66E-05	3	3	790.6666667	339	451.6666667	1.684326561	1.740828703	Higher in NONMETA	868	570	934	348	176	493
ENSMUSG00000089876.3	Tmem102	1.96E-07	1.70E-05	3	3	641	95.66666667	545.3333333	2.898346918	3.237261677	Higher in NONMETA	802	677	444	149	51	87
ENSMUSG00000020758.15	Itgb4	2.05E-07	1.77E-05	3	3	8197.666667	1449.666667	6748	3.01002125	3.398687816	Higher in NONMETA	5143	11220	8230	1176	260	2913
ENSMUSG00000024922.1	Ovol1	2.08E-07	1.79E-05	3	3	279	7.666666667	271.3333333	3.947762677	5.189370526	Higher in NONMETA	153	20	664	10	7	6
ENSMUSG00000047281.3	Sfn	2.26E-07	1.93E-05	3	3	8727.666667	1219.333333	7508.333333	2.935062073	3.29332611	Higher in NONMETA	2086	5959	18138	1016	603	2039
ENSMUSG00000019590.16	Cyb561	2.77E-07	2.33E-05	3	3	2086	807.6666667	1278.333333	1.938839844	2.029735198	Higher in NONMETA	2024	2233	2001	686	408	1329
ENSMUSG00000054662.8	Ano9	3.13E-07	2.62E-05	3	3	421	10.33333333	410.6666667	3.988960596	5.372444615	Higher in NONMETA	57	205	1001	22	6	3
ENSMUSG00000068299.11	Nat8f4	3.17E-07	2.64E-05	3	3	254.3333333	85.66666667	168.6666667	2.08214199	2.19787122	Higher in NONMETA	210	268	285	71	45	141
ENSMUSG00000023019.12	Gpd1	3.25E-07	2.68E-05	3	3	6452	174.6666667	6277.333333	4.012910801	5.423413119	Higher in NONMETA	4628	134	14594	266	49	209
ENSMUSG00000037145.14	2210407C18Rik	3.35E-07	2.75E-05	3	3	179.3333333	3.666666667	175.6666667	4.187780488	6.128910982	Higher in NONMETA	366	5	167	4	1	6
ENSMUSG00000060962.12	Dmkn	4.25E-07	3.45E-05	3	3	4446.333333	36	4410.333333	4.360694433	7.289604374	Higher in NONMETA	1124	9	12206	23	13	72
ENSMUSG00000048458.8	Inka2	4.70E-07	3.79E-05	3	3	456.3333333	61.33333333	395	2.945406559	3.337048077	Higher in NONMETA	803	331	235	48	62	74
ENSMUSG00000062115.15	Rai1	4.75E-07	3.81E-05	3	3	2657	1463	1194	1.412278913	1.447305773	Higher in NONMETA	2543	2199	3229	1260	736	2393
ENSMUSG00000020634.12	Ubxn2a	4.83E-07	3.86E-05	3	3	1626.333333	758	868.3333333	1.621898055	1.675919947	Higher in NONMETA	1811	1336	1732	723	388	1163
ENSMUSG00000028544.14	Slc5a9	5.14E-07	4.08E-05	3	3	979.3333333	17.33333333	962	4.215267481	6.408953688	Higher in NONMETA	1364	6	1568	15	2	35
ENSMUSG00000001420.13	Tmem79	5.41E-07	4.27E-05	3	3	474.6666667	127.3333333	347.3333333	2.451956118	2.659147275	Higher in NONMETA	392	408	624	96	28	258
ENSMUSG00000046314.16	Stxbp6	5.94E-07	4.67E-05	3	3	1186.666667	152.6666667	1034	2.997814048	3.423759877	Higher in NONMETA	2269	391	900	211	64	183
ENSMUSG00000036292.14	Gramd1c	6.28E-07	4.91E-05	3	3	272.6666667	113.6666667	159	1.679747469	1.741569793	Higher in NONMETA	252	216	350	96	80	165
ENSMUSG00000026304.13	Rab17	6.38E-07	4.96E-05	3	3	410.6666667	30.33333333	380.3333333	3.607217678	4.517017202	Higher in NONMETA	882	54	296	18	7	66
ENSMUSG00000021097.15	Clmn	6.68E-07	5.14E-05	3	3	1645.666667	106.6666667	1539	3.511813585	4.323895758	Higher in NONMETA	2587	87	2263	106	57	157
ENSMUSG00000029102.9	Hgfac	7.94E-07	6.01E-05	3	3	231	3.666666667	227.3333333	4.215679975	6.78348551	Higher in NONMETA	4	290	399	3	0	8
ENSMUSG00000069892.9	9930111J21Rik2	8.03E-07	6.04E-05	3	3	415.6666667	169	246.6666667	1.710612481	1.777332383	Higher in NONMETA	490	242	515	134	113	260
ENSMUSG00000041372.10	B4galnt3	8.53E-07	6.33E-05	3	3	343.6666667	9.666666667	334	4.146661192	6.350797294	Higher in NONMETA	19	870	142	6	1	22
ENSMUSG00000000706.15	Btn1a1	8.98E-07	6.63E-05	3	3	1487	188	1299	2.966079305	3.39347684	Higher in NONMETA	1474	362	2625	257	42	265
ENSMUSG00000072825.11	Cep170b	9.31E-07	6.84E-05	3	3	5833.333333	1893.666667	3939.666667	2.192311062	2.342559742	Higher in NONMETA	4770	7505	5225	1666	867	3148
ENSMUSG00000029859.6	Epha1	9.70E-07	7.09E-05	3	3	725.3333333	182.6666667	542.6666667	2.587375177	2.850915797	Higher in NONMETA	521	724	931	68	57	423
ENSMUSG00000001552.14	Jup	1.00E-06	7.23E-05	3	3	10911.33333	4025	6886.333333	1.842903897	1.928997204	Higher in NONMETA	8886	9499	14349	4887	1972	5216
ENSMUSG00000027356.8	Fermt1	1.14E-06	8.16E-05	3	3	2837	139.6666667	2697.333333	3.584584781	4.544965271	Higher in NONMETA	1815	4731	1965	110	265	44
ENSMUSG00000030688.15	Stard10	1.20E-06	8.52E-05	3	3	3791	735	3056	2.45723368	2.683039193	Higher in NONMETA	3563	3972	3838	741	831	633
ENSMUSG00000035873.8	Pawr	1.20E-06	8.52E-05	3	3	927.3333333	232.6666667	694.6666667	2.318599813	2.504048166	Higher in NONMETA	1005	664	1113	331	74	293
ENSMUSG00000028262.13	Clca3a2	1.25E-06	8.85E-05	3	3	5416.666667	108.6666667	5308	3.979593043	5.731635906	Higher in NONMETA	4661	64	11525	218	35	73
ENSMUSG00000074121.3	Ntf5	1.28E-06	8.96E-05	3	3	361	44.66666667	316.3333333	3.054060441	3.550337397	Higher in NONMETA	440	68	575	43	10	81
ENSMUSG00000062157.6	Ifnlr1	1.33E-06	9.23E-05	3	3	645.6666667	54.33333333	591.3333333	3.161938586	3.736019287	Higher in NONMETA	778	78	1081	69	42	52
ENSMUSG00000020173.17	Cobl	1.34E-06	9.23E-05	3	3	899.6666667	75	824.6666667	3.357322784	4.089919953	Higher in NONMETA	1702	390	607	138	11	76
ENSMUSG00000048924.13	Ccdc125	1.38E-06	9.47E-05	3	3	471.3333333	175.6666667	295.6666667	1.907658485	2.006703761	Higher in NONMETA	625	345	444	168	87	272
ENSMUSG00000046761.12	Fam83h	1.49E-06	0.000100846	3	3	2992.666667	957	2035.666667	2.265742665	2.440891034	Higher in NONMETA	2740	2134	4104	453	266	2152
ENSMUSG00000060512.7	0610040J01Rik	1.63E-06	0.000107613	3	3	2104.333333	131.6666667	1972.666667	3.465502951	4.330472561	Higher in NONMETA	4502	172	1639	185	78	132
ENSMUSG00000056632.7	Dsg3	1.65E-06	0.000108835	3	3	1665	16.66666667	1648.333333	4.174640141	7.232067539	Higher in NONMETA	193	15	4787	4	2	44
ENSMUSG00000056025.12	Clca3a1	1.71E-06	0.000111907	3	3	896.6666667	35.66666667	861	3.72135394	4.962238207	Higher in NONMETA	641	24	2025	44	9	54
ENSMUSG00000020205.8	Phlda1	1.80E-06	0.000116279	3	3	5628	2447.333333	3180.666667	1.661326293	1.726577455	Higher in NONMETA	7427	3266	6191	1843	1542	3957
ENSMUSG00000039452.6	Snx22	1.86E-06	0.000119929	3	3	274.6666667	21	253.6666667	3.469003055	4.360425913	Higher in NONMETA	656	50	118	12	11	40
ENSMUSG00000042268.10	Slc26a9	1.90E-06	0.000121831	3	3	401.6666667	21.66666667	380	3.75069718	5.072371742	Higher in NONMETA	916	58	231	2	8	55
ENSMUSG00000035545.13	Leng8	1.92E-06	0.000122185	3	3	4259	1799.333333	2459.666667	1.757395021	1.835847412	Higher in NONMETA	2660	3909	6208	1366	925	3107
ENSMUSG00000019737.14	Syne4	1.94E-06	0.000123427	3	3	980	36.66666667	943.3333333	3.754060778	5.090714096	Higher in NONMETA	1637	14	1289	34	23	53
ENSMUSG00000025582.4	Nptx1	1.96E-06	0.000123898	3	3	1481.666667	71	1410.666667	3.838538913	5.363936826	Higher in NONMETA	82	3780	583	64	28	121
ENSMUSG00000050520.4	Cldn8	2.03E-06	0.000127696	3	3	835	34.33333333	800.6666667	3.683996868	4.898043276	Higher in NONMETA	1554	33	918	60	13	30
ENSMUSG00000020940.13	1700023F06Rik	2.33E-06	0.000144388	3	3	59	7.333333333	51.66666667	2.960847098	3.451147636	Higher in NONMETA	44	62	71	10	5	7
ENSMUSG00000035686.8	Thrsp	2.39E-06	0.000147198	3	3	7448.666667	156.3333333	7292.333333	3.922250187	5.77686478	Higher in NONMETA	8757	86	13503	323	25	121

ENSMUSG00000034949.18	Zfr2	2.50E-06	0.000152432	3	3	112	39	73	1.978226689	2.096148971	Higher in NONMETA	96	94	146	32	20	65
ENSMUSG00000038605.11	Samd10	2.76E-06	0.000166185	3	3	608.6666667	185.3333333	423.3333333	2.166449114	2.326940701	Higher in NONMETA	489	682	655	223	73	260
ENSMUSG00000021792.15	Fam213a	2.79E-06	0.000167768	3	3	2595.666667	893.6666667	1702	1.918276704	2.026019024	Higher in NONMETA	2413	1089	4285	525	528	1628
ENSMUSG00000039087.16	Rreb1	2.82E-06	0.000168587	3	3	2312.333333	1349.666667	962.6666667	1.305885175	1.337881548	Higher in NONMETA	2112	1781	3044	1214	657	2178
ENSMUSG00000004864.12	Mapk13	2.88E-06	0.000171856	3	3	2614	468	2146	2.930859205	3.397917968	Higher in NONMETA	1269	3743	2830	385	67	952
ENSMUSG00000026915.16	Strbp	3.09E-06	0.000183559	3	3	2405	1298	1107	1.327280862	1.361240845	Higher in NONMETA	2396	1810	3009	1055	937	1902
ENSMUSG00000026383.14	Epb41l5	3.35E-06	0.000197211	3	3	941	405.6666667	535.3333333	1.553529738	1.609528254	Higher in NONMETA	777	665	1381	407	285	525
ENSMUSG00000031372.7	Trex2	3.50E-06	0.000205423	3	3	73.33333333	0.666666667	72.66666667	4.008426627	6.986549078	Higher in NONMETA	9	13	198	1	0	1
ENSMUSG00000057729.12	Prtn3	3.57E-06	0.000208361	3	3	2943.666667	65	2878.666667	3.883944918	5.814180122	Higher in NONMETA	6632	23	2176	23	77	95
ENSMUSG00000026971.15	Itgb6	3.72E-06	0.000216354	3	3	277	63	214	2.379668087	2.607362719	Higher in NONMETA	350	161	320	91	22	76
ENSMUSG00000037685.15	Atp8a1	3.98E-06	0.000230234	3	3	1907.333333	768.3333333	1139	1.821235055	1.915619693	Higher in NONMETA	2492	1629	1601	544	503	1258
ENSMUSG00000024769.7	Cdc42bpg	4.02E-06	0.000231959	3	3	3508.666667	1205	2303.666667	2.107514203	2.259706629	Higher in NONMETA	3150	4542	2834	1086	568	1961
ENSMUSG00000028555.15	Ttc39a	4.04E-06	0.000231959	3	3	951.6666667	71.33333333	880.3333333	3.329235254	4.156780742	Higher in NONMETA	1888	71	896	96	28	90
ENSMUSG00000027570.15	Col9a3	4.16E-06	0.000237813	3	3	3276.333333	117	3159.333333	3.742906673	5.282007347	Higher in NONMETA	7948	48	1833	137	54	160
ENSMUSG00000039865.8	Slc44a3	4.57E-06	0.000257599	3	3	650.6666667	78	572.6666667	2.960699269	3.476746364	Higher in NONMETA	996	72	884	73	40	121
ENSMUSG00000035936.6	Aldh5a1	4.64E-06	0.000260369	3	3	291.3333333	128.3333333	163	1.646605686	1.715904626	Higher in NONMETA	283	162	429	98	64	223
ENSMUSG00000000303.12	Cdh1	4.76E-06	0.00026624	3	3	13725.66667	1299	12426.66667	3.0672164	3.663600807	Higher in NONMETA	12575	9683	18919	2986	415	496
ENSMUSG00000024940.10	Ltbp3	5.62E-06	0.000308242	3	3	12802	7781	5021	1.190620989	1.216282816	Higher in NONMETA	13173	9562	15671	6412	5190	11741
ENSMUSG00000033256.14	Shf	5.68E-06	0.0003103	3	3	3987	1617.333333	2369.666667	1.793969648	1.887154614	Higher in NONMETA	5333	1878	4750	1106	802	2944
ENSMUSG00000026437.11	Cdk18	5.81E-06	0.000315426	3	3	1272	502	770	1.841906429	1.943701009	Higher in NONMETA	1287	1384	1145	357	358	791
ENSMUSG00000040564.14	Apoc1	5.87E-06	0.000317003	3	3	1026	60	966	3.463219991	4.527708017	Higher in NONMETA	1805	42	1231	86	14	80
ENSMUSG00000024331.11	Dsc2	5.92E-06	0.000318751	3	3	2163.666667	252	1911.666667	3.084388563	3.713369279	Higher in NONMETA	502	1015	4974	190	34	532
ENSMUSG00000054342.8	Kcnn4	5.97E-06	0.000320378	3	3	7091.666667	2941.333333	4150.333333	1.742000176	1.827248545	Higher in NONMETA	8272	6897	6106	2542	2041	4241
ENSMUSG00000029438.9	Bcl7a	6.30E-06	0.000336281	3	3	1041	276.3333333	764.6666667	2.139272624	2.307089221	Higher in NONMETA	864	321	1938	208	161	460
ENSMUSG00000053414.7	Hunk	6.32E-06	0.000336281	3	3	2139.666667	92.66666667	2047	3.651191887	5.103489776	Higher in NONMETA	5059	34	1326	91	33	154
ENSMUSG00000001249.14	Hpn	6.34E-06	0.000336361	3	3	1390	133	1257	3.060151408	3.676173989	Higher in NONMETA	2300	167	1703	212	61	126
ENSMUSG00000004791.7	Pgf	6.62E-06	0.000347417	3	3	3665.333333	1686.666667	1978.666667	1.671138864	1.746556921	Higher in NONMETA	2603	2913	5480	970	850	3240
ENSMUSG00000074272.10	Ceacam1	6.65E-06	0.000347571	3	3	4613.666667	246.6666667	4367	3.535443406	4.750329724	Higher in NONMETA	10419	167	3255	339	55	346
ENSMUSG00000032062.2	2310030G06Rik	6.92E-06	0.000357191	3	3	729.3333333	215	514.3333333	2.093895778	2.251879065	Higher in NONMETA	867	586	735	305	95	245
ENSMUSG00000028115.16	Bnipl	7.32E-06	0.00037602	3	3	661	38.66666667	622.3333333	3.417862831	4.457909737	Higher in NONMETA	1097	20	866	48	17	51
ENSMUSG00000068744.12	Psrc1	8.03E-06	0.000407951	3	3	2451.666667	118.6666667	2333	3.484460117	4.653146344	Higher in NONMETA	6304	183	868	72	149	135
ENSMUSG00000074796.10	Slc4a11	8.74E-06	0.000439579	3	3	3837	249	3588	3.489210633	4.685973563	Higher in NONMETA	756	9072	1683	488	61	198
ENSMUSG00000074277.4	Phldb3	8.93E-06	0.000446202	3	3	634	252.6666667	381.3333333	1.620679034	1.691356138	Higher in NONMETA	497	392	1013	219	196	343
ENSMUSG00000025507.13	Pidd1	8.98E-06	0.000447048	3	3	401.6666667	163.3333333	238.3333333	1.655159065	1.73091612	Higher in NONMETA	425	317	463	137	137	216
ENSMUSG00000034382.14	AI661453	9.27E-06	0.000459933	3	3	1050	194.6666667	855.3333333	2.602188926	2.9496434	Higher in NONMETA	414	1405	1331	288	89	207
ENSMUSG00000020541.12	Tom1l1	9.31E-06	0.000460339	3	3	1023.666667	537	486.6666667	1.496315455	1.551310273	Higher in NONMETA	934	689	1448	397	218	996
ENSMUSG00000025500.12	Lmntd2	9.41E-06	0.000463312	3	3	175.3333333	34	141.3333333	2.739596411	3.157649365	Higher in NONMETA	132	104	290	10	8	84
ENSMUSG00000020021.4	Fgd6	9.43E-06	0.000463312	3	3	2131.666667	876	1255.666667	1.955596356	2.085848578	Higher in NONMETA	1758	2322	2315	519	320	1789
ENSMUSG00000039063.5	Echdc3	1.03E-05	0.000500504	3	3	450.3333333	224.3333333	226	1.513510444	1.571115368	Higher in NONMETA	423	266	662	162	106	405
ENSMUSG00000049971.17	Glt1d1	1.07E-05	0.000514268	3	3	372.3333333	26	346.3333333	3.474385549	4.691712232	Higher in NONMETA	575	37	505	10	1	67
ENSMUSG00000039853.18	Trim14	1.10E-05	0.000525661	3	3	362	120	242	1.855315672	1.967084537	Higher in NONMETA	316	254	516	155	73	132
ENSMUSG00000006411.12	Nectin4	1.12E-05	0.000535018	3	3	1723	383	1340	2.350381605	2.597869203	Higher in NONMETA	682	1943	2544	494	238	417
ENSMUSG00000061397.7	Krt79	1.13E-05	0.000540029	3	3	2000	22	1978	3.823779807	6.442410816	Higher in NONMETA	1017	2	4981	33	18	15
ENSMUSG00000068876.14	Cgn	1.14E-05	0.0005407	3	3	1159.666667	376.3333333	783.3333333	2.071707455	2.232905133	Higher in NONMETA	715	1356	1408	435	153	541
ENSMUSG00000079620.13	Muc4	1.16E-05	0.00054731	3	3	511	17.33333333	493.6666667	3.644336553	5.335331222	Higher in NONMETA	303	4	1226	10	5	37
ENSMUSG00000040447.15	Spns2	1.18E-05	0.000553514	3	3	4457	733.6666667	3723.333333	2.815446347	3.297651285	Higher in NONMETA	1809	8554	3008	958	374	869
ENSMUSG00000040413.15	Timd2	1.18E-05	0.000553514	3	3	331.3333333	13.33333333	318	3.601825089	5.176339358	Higher in NONMETA	654	4	336	13	4	23
ENSMUSG00000047230.6	Cldn2	1.32E-05	0.000613457	3	3	160.3333333	6.333333333	154	3.567780843	5.104950007	Higher in NONMETA	228	4	249	8	1	10
ENSMUSG00000034285.15	Nipsnap1	1.37E-05	0.000636029	3	3	987.6666667	250.6666667	737	2.156286167	2.345019019	Higher in NONMETA	1490	326	1147	248	181	323
ENSMUSG00000031958.16	Ldhd	1.43E-05	0.000656188	3	3	890.3333333	190	700.3333333	2.290275021	2.523430867	Higher in NONMETA	507	327	1837	109	159	302
ENSMUSG00000034362.7	Csta1	1.44E-05	0.000658799	3	3	83.33333333	3	80.33333333	3.559703307	5.154098762	Higher in NONMETA	66	4	180	1	2	6
ENSMUSG00000037188.7	Grhl3	1.46E-05	0.000665493	3	3	286.3333333	5.333333333	281	3.708099171	5.87312104	Higher in NONMETA	66	3	790	2	5	9
ENSMUSG00000042010.16	Acacb	1.47E-05	0.000668845	3	3	1555	110.6666667	1444.333333	3.161735504	3.973225735	Higher in NONMETA	838	98	3729	162	44	126
ENSMUSG00000076434.9	Wfdc3	1.50E-05	0.000680288	3	3	401.6666667	41	360.6666667	3.108633966	3.863580453	Higher in NONMETA	595	28	582	17	19	87
ENSMUSG00000073968.4	Trim68	1.53E-05	0.000690482	3	3	632	344.6666667	287.3333333	1.360070708	1.40305034	Higher in NONMETA	554	407	935	235	201	598
ENSMUSG00000025329.3	Padi1	1.56E-05	0.000702842	3	3	630.6666667	64.33333333	566.3333333	3.226049797	4.128255169	Higher in NONMETA	56	1285	551	49	33	111
ENSMUSG00000027692.16	Tnik	1.69E-05	0.000754061	3	3	1877.333333	464.6666667	1412.666667	2.152336185	2.344577205	Higher in NONMETA	2666	1032	1934	666	316	412
ENSMUSG00000070780.11	Rbm47	1.71E-05	0.00076079	3	3	2137	696.3333333	1440.666667	1.946529715	2.083940933	Higher in NONMETA	3344	978	2089	520	517	1052
ENSMUSG00000015134.15	Aldh1a3	1.72E-05	0.000765268	3	3	5267.666667	382.3333333	4885.333333	3.295305711	4.314023347	Higher in NONMETA	4413	192	11198	319	51	777
ENSMUSG00000055976.6	Cldn23	1.77E-05	0.000777263	3	3	268.6666667	27.33333333	241.3333333	2.871520368	3.429988332	Higher in NONMETA	80	142	584	45	18	19
ENSMUSG00000053797.10	Krt16	1.87E-05	0.000816799	3	3	4005.333333	131	3874.333333	3.599319181	5.383970431	Higher in NONMETA	418	67	11531	60	33	300
ENSMUSG00000032091.15	Tmprss4	1.88E-05	0.000821162	3	3	640	8	632	3.772288465	6.771716815	Higher in NONMETA	1719	3	198	16	1	7
ENSMUSG00000074923.10	Pak6	1.98E-05	0.000855654	3	3	826.3333333	160.3333333	666	2.554213659	2.91292508	Higher in NONMETA	838	170	1471	58	80	343
ENSMUSG00000016763.14	Scube1	1.98E-05	0.000855654	3	3	1817.666667	96	1721.666667	3.413439351	4.688360078	Higher in NONMETA	4708	71	674	138	42	108
ENSMUSG00000078922.9	Tgtp1	1.98E-05	0.000855654	3	3	2280	87.33333333	2192.666667	3.569148843	5.277460728	Higher in NONMETA	2165	9	4666	40	24	198
ENSMUSG00000073988.13	Ttpa	2.06E-05	0.000887336	3	3	543	30	513	3.371252542	4.571706032	Higher in NONMETA	418	21	1190	38	3	49
ENSMUSG00000062345.10	Serpinb2	2.16E-05	0.000925502	3	3	1471	94.66666667	1376.333333	3.373938844	4.591904292	Higher in NONMETA	58	3056	1299	137	53	94
ENSMUSG00000024511.15	Rab27b	2.21E-05	0.000944788	3	3	919.6666667	346.6666667	573	1.932299233	2.070588876	Higher in NONMETA	1438	599	722	277	154	609
ENSMUSG00000029074.14	Ttll10	2.23E-05	0.000950646	3	3	118	14.66666667	103.3333333	3.093295663	3.880604036	Higher in NONMETA	41	115	198	7	1	36
ENSMUSG00000006143.12	Upk3bl	2.24E-05	0.000950646	3	3	124.6666667	8.666666667	116	3.378209408	4.609454219	Higher in NONMETA	35	54	285	1	1	24
ENSMUSG00000034616.16	Ssh3	2.27E-05	0.000963716	3	3	2521.666667	1128.333333	1393.333333	1.67333154	1.760114054	Higher in NONMETA	1864	2665	3036	1172	515	1698
ENSMUSG00000050747.14	Trim15	2.31E-05	0.000975077	3	3	84.66666667	8	76.66666667	3.107711267	3.941927285	Higher in NONMETA	185	31	38	4	7	13
ENSMUSG00000026435.15	Slc45a3	2.43E-05	0.00101609	3	3	1210.666667	265.3333333	945.3333333	2.274986986	2.518772662	Higher in NONMETA	1395	581	1656	450	110	236
ENSMUSG00000064246.10	Chil1	2.44E-05	0.001017606	3	3	20283.66667	625	19658.66667	3.613608647	5.654964634	Higher in NONMETA	53313	105	7433	718	137	1020
ENSMUSG00000037469.9	Acp7	2.44E-05	0.00101824	3	3	366.3333333	2.666666667	363.6666667	3.763335427	7.495439568	Higher in NONMETA	147	0	952	1	1	6
ENSMUSG00000039813.14	Tbc1d2	2.51E-05	0.001042752	3	3	723.3333333	185	538.3333333	2.489991479	2.827981473	Higher in NONMETA	306	1176	688	128	70	357

ENSMUSG00000027901.12	Dennd2d	2.71E-05	0.001117659	3	3	625.6666667	103	522.6666667	2.527540669	2.889011009	Higher in NONMETA	519	102	1256	114	52	143
ENSMUSG00000015533.8	Itga2	2.86E-05	0.001170174	3	3	778	132.3333333	645.6666667	2.889032322	3.502936681	Higher in NONMETA	141	1262	931	60	41	296
ENSMUSG00000078954.9	Arhgap8	3.00E-05	0.00122443	3	3	992	111.6666667	880.3333333	3.147144847	4.055212273	Higher in NONMETA	2040	109	827	36	16	283
ENSMUSG00000057286.6	St6galnac2	3.17E-05	0.001288769	3	3	1928.333333	185.3333333	1743	2.960646409	3.655878105	Higher in NONMETA	4416	266	1103	233	163	160
ENSMUSG00000039835.16	Nhsl1	3.19E-05	0.001292434	3	3	5557.666667	1238	4319.666667	2.389846886	2.691140498	Higher in NONMETA	9609	1938	5126	1642	365	1707
ENSMUSG00000028080.16	Lrba	3.31E-05	0.001331916	3	3	1242.333333	673	569.3333333	1.36720586	1.414913041	Higher in NONMETA	1281	958	1488	721	326	972
ENSMUSG00000029086.15	Prom1	3.32E-05	0.001334154	3	3	2913	113.6666667	2799.333333	3.507246925	5.292960318	Higher in NONMETA	7174	24	1541	141	19	181
ENSMUSG00000025330.6	Padi4	3.34E-05	0.001339495	3	3	3752	959.3333333	2792.666667	2.409202068	2.721494331	Higher in NONMETA	1235	5491	4530	875	342	1661
ENSMUSG00000049265.7	Kcnk3	3.44E-05	0.001370007	3	3	334.3333333	32.66666667	301.6666667	2.890309453	3.529840481	Higher in NONMETA	250	30	723	21	30	47
ENSMUSG00000027318.17	Adam33	3.45E-05	0.001370007	3	3	260.6666667	95.66666667	165	1.744187976	1.848660688	Higher in NONMETA	205	126	451	73	64	150
ENSMUSG00000021795.9	Sftpd	3.55E-05	0.001404904	3	3	174.3333333	19	155.3333333	3.091049148	3.952215487	Higher in NONMETA	287	23	213	11	2	44
ENSMUSG00000025505.16	Tmem80	3.60E-05	0.001414534	3	3	658	346.6666667	311.3333333	1.373890758	1.422837607	Higher in NONMETA	802	400	772	316	204	520
ENSMUSG00000033006.9	Sox10	3.66E-05	0.00143333	3	3	6217	222.3333333	5994.666667	3.467473652	5.1584142	Higher in NONMETA	12227	41	6383	400	52	215
ENSMUSG00000002459.17	Rgs20	3.71E-05	0.001449333	3	3	388.3333333	134.6666667	253.6666667	1.950198817	2.102272527	Higher in NONMETA	521	149	495	109	53	242
ENSMUSG00000021567.15	Nkd2	3.77E-05	0.001469687	3	3	9338.333333	994.6666667	8343.666667	2.923762544	3.602460942	Higher in NONMETA	20050	1116	6849	1602	421	961
ENSMUSG00000026768.10	Itga8	3.83E-05	0.001483807	3	3	1822.333333	136.3333333	1686	3.216855139	4.289696222	Higher in NONMETA	3322	44	2101	146	30	233
ENSMUSG00000038859.7	Baiap2l1	3.85E-05	0.001489161	3	3	699	240.3333333	458.6666667	1.99770586	2.163633633	Higher in NONMETA	1177	378	542	168	130	423
ENSMUSG00000053693.16	Mast1	3.93E-05	0.001510477	3	3	158.6666667	46.66666667	112	2.205563798	2.439002415	Higher in NONMETA	175	112	189	14	28	98
ENSMUSG00000028786.15	Tmem54	3.99E-05	0.001526628	3	3	465.3333333	90	375.3333333	2.394838831	2.709320384	Higher in NONMETA	711	116	569	51	87	132
ENSMUSG00000026870.19	Cutal	4.00E-05	0.001527932	3	3	155	32.66666667	122.3333333	2.586850339	3.006921923	Higher in NONMETA	86	270	109	30	17	51
ENSMUSG00000033268.8	Duox1	4.07E-05	0.001538438	3	3	708.3333333	61.33333333	647	3.171019561	4.18081073	Higher in NONMETA	1197	20	908	35	17	132
ENSMUSG00000058897.17	Col25a1	4.13E-05	0.001554391	3	3	1280.666667	89	1191.666667	3.343305578	4.709401837	Higher in NONMETA	3036	38	768	47	11	209
ENSMUSG00000035638.14	Muc20	4.15E-05	0.001560544	3	3	376	19.33333333	356.6666667	3.26483482	4.462718084	Higher in NONMETA	489	7	632	29	11	18
ENSMUSG00000049128.7	Ivl	4.17E-05	0.001563391	3	3	405.6666667	8.666666667	397	3.604215546	6.256587221	Higher in NONMETA	165	8	1044	0	1	25
ENSMUSG00000031150.12	Ccdc120	4.20E-05	0.001569265	3	3	979.6666667	531.6666667	448	1.38399401	1.435023312	Higher in NONMETA	821	919	1199	526	282	787
ENSMUSG00000016526.8	Dyrk3	4.24E-05	0.001580374	3	3	1691.666667	388.6666667	1303	2.392140426	2.7073874	Higher in NONMETA	2918	1500	657	466	214	486
ENSMUSG00000027375.14	Mal	4.26E-05	0.001583699	3	3	401.6666667	38	363.6666667	3.146135802	4.128824982	Higher in NONMETA	76	793	336	68	6	40
ENSMUSG00000078234.6	Klhdc7a	4.27E-05	0.001583699	3	3	2226.666667	124	2102.666667	3.232458037	4.364067976	Higher in NONMETA	2059	90	4531	246	19	107
ENSMUSG00000006471.18	Ndor1	4.30E-05	0.001591264	3	3	1625	1006	619	1.257715619	1.29558798	Higher in NONMETA	1434	1151	2290	742	454	1822
ENSMUSG00000036617.16	Etl4	4.34E-05	0.001592698	3	3	1901.333333	734.6666667	1166.666667	1.708836905	1.809406368	Higher in NONMETA	1698	1634	2372	999	393	812
ENSMUSG00000078606.8	Gm4070	4.38E-05	0.001596818	3	3	455	49.66666667	405.3333333	2.775344886	3.337289148	Higher in NONMETA	184	63	1118	48	36	65
ENSMUSG00000029188.14	Slc34a2	4.38E-05	0.001596818	3	3	234	26	208	3.014768882	3.817082969	Higher in NONMETA	433	22	247	22	5	51
ENSMUSG00000035967.15	Ints6l	4.39E-05	0.001596818	3	3	758	399.6666667	358.3333333	1.437564613	1.495346869	Higher in NONMETA	611	478	1185	259	197	743
ENSMUSG00000028836.14	Slc30a2	4.39E-05	0.001596818	3	3	1028.666667	33	995.6666667	3.51294765	5.553640778	Higher in NONMETA	2876	8	202	16	23	60
ENSMUSG00000020264.5	Slc36a2	4.45E-05	0.001609731	3	3	596.3333333	68	528.3333333	2.865944911	3.508228692	Higher in NONMETA	466	65	1258	86	13	105
ENSMUSG00000024913.16	Lrp5	4.46E-05	0.001609731	3	3	7203.333333	3439.333333	3764	1.480699638	1.544358937	Higher in NONMETA	7430	6223	7957	4026	2039	4253
ENSMUSG00000041936.18	Agrn	4.54E-05	0.001628868	3	3	13498	5568.333333	7929.666667	1.736252058	1.842753578	Higher in NONMETA	8708	14994	16792	6207	2731	7767
ENSMUSG00000028640.11	Tfap2c	4.72E-05	0.00168734	3	3	3154	116.3333333	3037.666667	3.417281793	5.077294115	Higher in NONMETA	5341	35	4086	233	14	102
ENSMUSG00000019851.8	Perp	4.84E-05	0.001723072	3	3	11573.66667	1798.666667	9775	2.983278902	3.762055253	Higher in NONMETA	3672	13176	17873	269	206	4921
ENSMUSG00000043782.8	Bicdl2	5.05E-05	0.001793633	3	3	189	36.33333333	152.6666667	2.367622842	2.681230228	Higher in NONMETA	80	152	335	52	23	34
ENSMUSG00000021696.8	Elovl7	5.06E-05	0.001793633	3	3	990.3333333	208.6666667	781.6666667	2.500967605	2.882621309	Higher in NONMETA	590	1227	1154	323	37	266
ENSMUSG00000024479.2	Mal2	5.08E-05	0.001798197	3	3	1962.666667	90	1872.666667	3.380415908	4.953941093	Higher in NONMETA	4837	16	1035	106	36	128
ENSMUSG00000019647.16	Sema6a	5.18E-05	0.001823833	3	3	1654.333333	672.3333333	982	1.871474102	2.010185717	Higher in NONMETA	1855	1607	1501	699	197	1121
ENSMUSG00000030935.15	Acsm3	5.40E-05	0.001892763	3	3	120.3333333	23.66666667	96.66666667	2.516519663	2.909467384	Higher in NONMETA	77	43	241	16	6	49
ENSMUSG00000035640.18	Cbarp	5.52E-05	0.00192442	3	3	690.6666667	313.6666667	377	1.49288151	1.560046891	Higher in NONMETA	514	468	1090	214	245	482
ENSMUSG00000027513.11	Pck1	5.58E-05	0.00193496	3	3	1988.666667	149	1839.666667	3.107931267	4.087454888	Higher in NONMETA	1837	192	3937	249	9	189
ENSMUSG00000072949.6	Acot1	5.68E-05	0.001957635	3	3	1533.666667	155.3333333	1378.333333	2.993357248	3.812143313	Higher in NONMETA	3301	76	1224	160	65	241
ENSMUSG00000040502.5	9-Mar	5.72E-05	0.00196482	3	3	556	169.3333333	386.6666667	2.203799509	2.449254788	Higher in NONMETA	257	596	815	146	41	321
ENSMUSG00000027186.14	Elf5	6.07E-05	0.002075248	3	3	3501.666667	183.3333333	3318.333333	3.242524115	4.507855457	Higher in NONMETA	4945	79	5481	367	32	151
ENSMUSG00000054702.14	Ap1s3	6.12E-05	0.002089921	3	3	335.6666667	109	226.6666667	1.883371093	2.028671973	Higher in NONMETA	422	227	358	150	64	113
ENSMUSG00000105096.1	Gbp10	6.16E-05	0.00209764	3	3	547	42.33333333	504.6666667	3.145849985	4.216255696	Higher in NONMETA	582	11	1048	29	11	87
ENSMUSG00000066894.14	Vsig10	6.27E-05	0.002128425	3	3	1061.666667	646	415.6666667	1.206521366	1.241394724	Higher in NONMETA	949	942	1294	576	404	958
ENSMUSG00000070305.10	Mpzl3	6.38E-05	0.002162472	3	3	1239	453.3333333	785.6666667	1.791033663	1.914544211	Higher in NONMETA	1051	1427	1239	415	402	543
ENSMUSG00000021188.14	Trip11	6.48E-05	0.002189657	3	3	2449	1623.666667	825.3333333	1.178130438	1.210620293	Higher in NONMETA	2170	2025	3152	1238	779	2854
ENSMUSG00000034177.15	Rnf43	6.71E-05	0.00226166	3	3	535	55.33333333	479.6666667	2.928965017	3.696692515	Higher in NONMETA	861	26	718	64	20	82
ENSMUSG00000039238.6	Zfp750	6.72E-05	0.00226166	3	3	579.6666667	15.66666667	564	3.392393642	5.202903465	Higher in NONMETA	171	19	1549	38	2	7
ENSMUSG00000039529.8	Atp8b1	6.84E-05	0.002292615	3	3	1299.666667	527	772.6666667	1.659365511	1.756385599	Higher in NONMETA	833	1084	1982	640	260	681
ENSMUSG00000041287.5	Sox15	6.87E-05	0.002294942	3	3	114.3333333	3.666666667	110.6666667	3.345426385	5.032643936	Higher in NONMETA	18	7	318	5	2	4
ENSMUSG00000026831.16	1700007K13Rik	7.05E-05	0.002349447	3	3	38.33333333	1.333333333	37	3.402836215	5.443142321	Higher in NONMETA	90	8	17	2	0	2
ENSMUSG00000003134.10	Tbc1d8	7.25E-05	0.002410398	3	3	1224	533	691	1.594368895	1.680294919	Higher in NONMETA	1191	1160	1321	655	315	629
ENSMUSG00000028434.12	Epb41l4b	7.33E-05	0.002427034	3	3	1784	176.6666667	1607.333333	2.917129626	3.683899318	Higher in NONMETA	2769	79	2504	245	65	220
ENSMUSG00000026158.11	Ogfrl1	7.33E-05	0.002427034	3	3	3643	1590.333333	2052.666667	1.58246147	1.666425217	Higher in NONMETA	5185	1876	3868	1592	943	2236
ENSMUSG00000026235.14	Epha4	7.35E-05	0.002427034	3	3	947.3333333	253.3333333	694	2.132760276	2.360006222	Higher in NONMETA	542	501	1799	318	65	377
ENSMUSG00000022037.14	Clu	7.38E-05	0.002427796	3	3	21643.33333	5353	16290.33333	2.43259135	2.79809415	Higher in NONMETA	12579	39553	12798	6316	2120	7623
ENSMUSG00000032232.14	Cgnl1	7.81E-05	0.002558072	3	3	5798.666667	2054	3744.666667	1.917102191	2.076338899	Higher in NONMETA	5561	7578	4257	1587	1718	2857
ENSMUSG00000023067.13	Cdkn1a	8.00E-05	0.002607434	3	3	8642	3376	5266	1.813625413	1.946322204	Higher in NONMETA	13017	7018	5891	3550	1767	4811
ENSMUSG00000045103.17	Dmd	8.13E-05	0.002637908	3	3	932	298	634	2.02351971	2.215873958	Higher in NONMETA	1234	525	1037	382	74	438
ENSMUSG00000069893.10	9930111J21Rik1	8.21E-05	0.002652179	3	3	83	28	55	2.024960999	2.217172868	Higher in NONMETA	94	49	106	17	11	56
ENSMUSG00000020836.15	Coro6	8.43E-05	0.002704126	3	3	426	108.3333333	317.6666667	2.187907264	2.441802802	Higher in NONMETA	192	293	793	139	31	155
ENSMUSG00000003271.17	Sult2b1	8.53E-05	0.002732846	3	3	803.3333333	101.6666667	701.6666667	2.79725941	3.453178763	Higher in NONMETA	128	428	1854	122	15	168
ENSMUSG00000038342.15	Mlxip	8.56E-05	0.002733961	3	3	3685.666667	1753.333333	1932.333333	1.436690246	1.499577538	Higher in NONMETA	4495	1857	4705	1836	1037	2387
ENSMUSG00000038764.14	Ptpn3	9.12E-05	0.002888723	3	3	1625.333333	761.6666667	863.6666667	1.441900587	1.50609225	Higher in NONMETA	2067	948	1861	689	583	1013
ENSMUSG00000035206.10	Sppl2b	9.19E-05	0.002904786	3	3	1605.333333	1021	584.3333333	1.134855748	1.165112209	Higher in NONMETA	1763	1228	1825	922	639	1502
ENSMUSG00000046694.6	Tent5b	9.50E-05	0.002984302	3	3	356	132.6666667	223.3333333	1.849178187	1.994472758	Higher in NONMETA	186	413	469	122	77	199
ENSMUSG00000041849.7	Card6	9.54E-05	0.002986471	3	3	744	437.6666667	306.3333333	1.391364015	1.448941993	Higher in NONMETA	693	639	900	321	175	817
ENSMUSG00000025017.9	Pik3ap1	9.55E-05	0.002986471	3	3	1113.666667	549.6666667	564	1.360431851	1.414118654	Higher in NONMETA	1120	604	1617	586	337	726

ENSMUSG00000053062.15	Jam2	9.73E-05	0.003029391	3	3	1063	465.6666667	597.3333333	1.761803097	1.885771955	Higher in NONMETA	1032	1291	866	450	211	736
ENSMUSG00000051397.5	Tacstd2	9.77E-05	0.003033591	3	3	3038	135	2903	3.214804783	4.605838711	Higher in NONMETA	47	1547	7520	265	53	87
ENSMUSG00000030994.15	D7Ertd443e	9.88E-05	0.003063669	3	3	137	39	98	2.182420133	2.440834602	Higher in NONMETA	219	70	122	18	24	75
ENSMUSG00000045349.15	Sh2d5	9.93E-05	0.003070943	3	3	3307.333333	978	2329.333333	2.183239573	2.441806784	Higher in NONMETA	1116	2913	5893	395	444	2095
ENSMUSG00000028454.16	Pigo	9.95E-05	0.003070943	3	3	985	625.6666667	359.3333333	1.181035587	1.215631573	Higher in NONMETA	800	765	1390	539	303	1035
ENSMUSG00000020183.11	Cpm	0.000100784	0.003092172	3	3	1175.666667	72.66666667	1103	3.210302391	4.603932339	Higher in NONMETA	3144	32	351	94	18	106
ENSMUSG00000003227.4	Edar	0.000101458	0.00310505	3	3	501.6666667	21.66666667	480	3.378161723	5.493612654	Higher in NONMETA	1378	6	121	5	4	56
ENSMUSG00000031200.16	Mtcp1	0.000102645	0.00313476	3	3	229.3333333	104.6666667	124.6666667	1.52739361	1.605863139	Higher in NONMETA	159	175	354	104	57	153
ENSMUSG00000026185.8	Igfbp5	0.00010334	0.00314937	3	3	43084	3281.333333	39802.66667	2.981191492	3.901207588	Higher in NONMETA	63997	1663	63592	6318	1285	2241
ENSMUSG00000021506.7	Pitx1	0.000105211	0.003199699	3	3	600	17.33333333	582.6666667	3.427038702	6.013802668	Higher in NONMETA	1144	1	655	5	1	46
ENSMUSG00000063130.4	Calml3	0.000106851	0.003240911	3	3	415.3333333	18.66666667	396.6666667	3.296196343	5.028943124	Higher in NONMETA	502	2	742	17	2	37
ENSMUSG00000032554.15	Trf	0.000107235	0.003240911	3	3	2865.333333	438	2427.333333	2.613412538	3.129407853	Higher in NONMETA	5941	380	2275	570	209	535
ENSMUSG00000024978.10	Gpam	0.000107847	0.003248052	3	3	657.3333333	321	336.3333333	1.492940718	1.566250084	Higher in NONMETA	599	394	979	330	119	514
ENSMUSG00000025202.7	Scd3	0.000113959	0.003415705	3	3	110.3333333	11	99.33333333	3.010005843	4.006559627	Higher in NONMETA	229	9	93	7	3	23
ENSMUSG00000012350.15	Ehf	0.000114759	0.003427998	3	3	4711.333333	228.6666667	4482.666667	3.172209834	4.517700898	Higher in NONMETA	2615	62	11457	411	46	229
ENSMUSG00000030337.16	Vamp1	0.00011484	0.003427998	3	3	610.3333333	228	382.3333333	1.844135869	1.991769858	Higher in NONMETA	382	302	1147	126	96	462
ENSMUSG00000006218.4	Fam131c	0.000117871	0.003494461	3	3	103.6666667	7	96.66666667	3.167306674	4.524873357	Higher in NONMETA	197	4	110	6	1	14
ENSMUSG00000030720.16	Cln3	0.000118564	0.003503169	3	3	1937.333333	1301.666667	635.6666667	1.08411491	1.111253201	Higher in NONMETA	2171	1305	2336	983	791	2131
ENSMUSG00000046727.12	Cystm1	0.000121276	0.003576037	3	3	2941	593	2348	2.45946777	2.874853707	Higher in NONMETA	2850	4902	1071	746	483	550
ENSMUSG00000037110.19	Ralgapa2	0.000123571	0.003628999	3	3	940.6666667	616.3333333	324.3333333	1.144535723	1.176817854	Higher in NONMETA	980	604	1238	511	291	1047
ENSMUSG00000008167.14	Fbxw9	0.000126069	0.003687457	3	3	1746.666667	730.3333333	1016.333333	1.786857211	1.921788303	Higher in NONMETA	2505	1615	1120	507	450	1234
ENSMUSG00000020532.18	Acaca	0.000127706	0.003727838	3	3	5166	1482	3684	2.065969027	2.287531108	Higher in NONMETA	5541	1058	8899	950	703	2793
ENSMUSG00000044037.15	Als2cl	0.00012872	0.003749901	3	3	1098.666667	191.3333333	907.3333333	2.491099714	2.932597019	Higher in NONMETA	166	1092	2038	242	99	233
ENSMUSG00000028536.12	2610528J11Rik	0.000129775	0.003773069	3	3	206.3333333	12.33333333	194	3.084022251	4.273904345	Higher in NONMETA	296	10	313	26	3	8
ENSMUSG00000056973.6	Ces1d	0.000130999	0.003799594	3	3	1036.333333	84.66666667	951.6666667	3.018211069	4.062936235	Higher in NONMETA	802	48	2259	99	6	149
ENSMUSG00000028150.14	Rorc	0.000131458	0.003799594	3	3	614	114.3333333	499.6666667	2.270481329	2.583019878	Higher in NONMETA	662	153	1027	171	82	90
ENSMUSG00000074115.5	Saa1	0.000131471	0.003799594	3	3	1022	53	969	3.186521477	4.643735509	Higher in NONMETA	1574	23	1469	97	3	59
ENSMUSG00000033508.7	Asprv1	0.000135774	0.003916153	3	3	1865.333333	167.3333333	1698	2.921114477	3.811031567	Higher in NONMETA	441	140	5015	180	32	290
ENSMUSG00000041488.16	Stx3	0.000136994	0.003943533	3	3	2889.333333	1604.666667	1284.666667	1.426015059	1.49153963	Higher in NONMETA	3426	2733	2509	1300	875	2639
ENSMUSG00000024812.9	Tjp2	0.000143463	0.00409729	3	3	6245	2150	4095	1.916703962	2.090986536	Higher in NONMETA	4329	8287	6119	2855	1156	2439
ENSMUSG00000063428.8	Ddo	0.000148527	0.004233606	3	3	260.6666667	31.33333333	229.3333333	2.829319103	3.608685871	Higher in NONMETA	283	18	481	26	5	63
ENSMUSG00000029868.13	Trpv6	0.000151955	0.004322842	3	3	585.6666667	28	557.6666667	3.246009143	5.034753486	Higher in NONMETA	1314	29	414	38	0	46
ENSMUSG00000040606.13	Kazn	0.000152602	0.004332767	3	3	1369.666667	486.6666667	883	1.652851827	1.760209363	Higher in NONMETA	1422	695	1992	585	389	486
ENSMUSG00000024276.6	Zfp397	0.000156956	0.004447685	3	3	2171.666667	1257.666667	914	1.413494675	1.478430463	Higher in NONMETA	1688	1933	2894	751	561	2461
ENSMUSG00000028760.16	Eif4g3	0.000160936	0.004533954	3	3	6008.666667	4157.666667	1851	1.091681409	1.120592739	Higher in NONMETA	5803	5099	7124	3716	2058	6699
ENSMUSG00000022687.12	Boc	0.000162037	0.004556156	3	3	1039.333333	506.6666667	532.6666667	1.345945705	1.401799192	Higher in NONMETA	832	579	1707	479	350	691
ENSMUSG00000034171.13	Faah	0.00016472	0.004613755	3	3	296.3333333	72.66666667	223.6666667	2.33804008	2.701055671	Higher in NONMETA	173	519	197	89	40	89
ENSMUSG00000057465.5	Saa2	0.000165607	0.004629678	3	3	598	31.33333333	566.6666667	3.172526138	4.719743992	Higher in NONMETA	1014	14	766	51	1	42
ENSMUSG00000080316.10	Spaca6	0.000168182	0.0046747	3	3	1242.666667	519	723.6666667	1.862981919	2.02515662	Higher in NONMETA	890	1211	1627	178	250	1129
ENSMUSG00000021950.15	Anxa8	0.000169587	0.00470474	3	3	4186	2254	1932	1.320539035	1.373444845	Higher in NONMETA	2833	3256	6469	2299	1174	3289
ENSMUSG00000027580.17	Helz2	0.000179214	0.004936622	3	3	2600.333333	1207	1393.333333	1.443332867	1.514149868	Higher in NONMETA	1528	1907	4366	1007	863	1751
ENSMUSG00000058886.9	Deaf1	0.000179304	0.004936622	3	3	1290.333333	801	489.3333333	1.093418068	1.122922648	Higher in NONMETA	1223	777	1871	705	524	1174
ENSMUSG00000018570.17	2810408A11Rik	0.000182373	0.005011638	3	3	192	71.66666667	120.3333333	2.020844737	2.237741168	Higher in NONMETA	125	225	226	48	20	147
ENSMUSG00000034227.7	Foxj1	0.000182911	0.005016935	3	3	133	24.33333333	108.6666667	2.402636192	2.815426025	Higher in NONMETA	237	47	115	36	14	23
ENSMUSG00000039956.8	Mrap	0.00019716	0.005384858	3	3	217	25.33333333	191.6666667	2.679297746	3.332823018	Higher in NONMETA	136	17	498	25	14	37
ENSMUSG00000040987.14	Mill2	0.000198286	0.005397923	3	3	137.3333333	45.66666667	91.66666667	1.862358056	2.02978234	Higher in NONMETA	88	142	182	52	32	53
ENSMUSG00000000983.13	Wfdc18	0.000200364	0.005434126	3	3	8562	632.3333333	7929.666667	2.92851843	3.930453031	Higher in NONMETA	10900	310	14476	1337	159	401
ENSMUSG00000037686.6	Aspg	0.000202953	0.005494088	3	3	353.3333333	27.66666667	325.6666667	2.85295757	3.738355255	Higher in NONMETA	465	13	582	23	34	26
ENSMUSG00000034981.9	Parm1	0.000203854	0.005502591	3	3	2823.666667	260.6666667	2563	2.899020638	3.853660647	Higher in NONMETA	6040	75	2356	355	105	322
ENSMUSG00000034282.3	Evpl	0.000204643	0.005502591	3	3	471.6666667	75.33333333	396.3333333	2.410058815	2.835357992	Higher in NONMETA	100	407	908	66	87	73
ENSMUSG00000038754.4	Elovl3	0.000206977	0.005551343	3	3	213	6.666666667	206.3333333	3.252731204	5.463831711	Higher in NONMETA	352	0	287	7	2	11
ENSMUSG00000049739.10	Zfp646	0.000209079	0.005587098	3	3	1711.333333	1104	607.3333333	1.212139	1.25375173	Higher in NONMETA	1355	1440	2339	866	492	1954
ENSMUSG00000021375.9	Kif13a	0.000210244	0.005607932	3	3	1607.333333	1156.666667	450.6666667	1.014163004	1.038047382	Higher in NONMETA	1646	1114	2062	982	571	1917
ENSMUSG00000055639.16	Dach1	0.000212436	0.005656019	3	3	282.3333333	128.3333333	154	1.515452279	1.600728924	Higher in NONMETA	311	251	285	129	97	159
ENSMUSG00000034255.17	Arhgap27	0.000214847	0.005709754	3	3	1721.333333	805.3333333	916	1.413319886	1.48135387	Higher in NONMETA	1351	1296	2517	980	502	934
ENSMUSG00000022479.15	Vdr	0.000217176	0.005750603	3	3	3399.333333	1726.666667	1672.666667	1.595516179	1.696345504	Higher in NONMETA	4406	1967	3825	892	690	3598
ENSMUSG00000014498.9	Ankrd52	0.0002189	0.00577522	3	3	2337	1498	839	1.231829426	1.275913604	Higher in NONMETA	2055	2253	2703	1276	711	2507
ENSMUSG00000061589.14	Dot1l	0.000225172	0.005919218	3	3	2536	1597.333333	938.6666667	1.105905059	1.137486295	Higher in NONMETA	2573	1554	3481	1135	1115	2542
ENSMUSG00000045761.15	Togaram2	0.000226378	0.005920837	3	3	755	60.33333333	694.6666667	3.01338098	4.241504706	Higher in NONMETA	1278	7	980	49	14	118
ENSMUSG00000019960.8	Dusp6	0.000226416	0.005920837	3	3	14958.33333	8835.666667	6122.666667	1.176062895	1.214369336	Higher in NONMETA	16757	11106	17012	7401	6791	12315
ENSMUSG00000022991.4	Lalba	0.000226792	0.005920837	3	3	2078.333333	252.6666667	1825.666667	2.843250225	3.732919644	Higher in NONMETA	4465	183	1587	273	21	464
ENSMUSG00000043924.16	Ncmap	0.000226863	0.005920837	3	3	359.3333333	37	322.3333333	2.934942393	3.989377588	Higher in NONMETA	824	14	240	12	17	82
ENSMUSG00000026922.13	Agpat2	0.0002276	0.00592942	3	3	3222.666667	1347.333333	1875.333333	1.575001416	1.672377476	Higher in NONMETA	2492	1219	5957	1101	723	2218
ENSMUSG00000043592.15	Unc5cl	0.000229499	0.005968209	3	3	379.6666667	26	353.6666667	3.065017759	4.445429295	Higher in NONMETA	702	4	433	28	4	46
ENSMUSG00000017639.13	Rab11fip4	0.000235252	0.006082404	3	3	530.6666667	141.6666667	389	2.04797313	2.286114969	Higher in NONMETA	675	106	811	137	78	210
ENSMUSG00000039110.14	Mycbpap	0.000238215	0.006130317	3	3	95.33333333	28.66666667	66.66666667	1.961707315	2.167845883	Higher in NONMETA	55	64	167	17	22	47
ENSMUSG00000060275.11	Nrg2	0.000238263	0.006130317	3	3	189.3333333	47	142.3333333	2.137848932	2.416651655	Higher in NONMETA	281	60	227	64	20	57
ENSMUSG00000036528.15	Ppfibp2	0.000245917	0.006316078	3	3	1707.666667	246.6666667	1461	2.658107329	3.319429518	Higher in NONMETA	3955	158	1010	284	95	361
ENSMUSG00000036492.12	Rnf39	0.000249224	0.006389714	3	3	160.3333333	19.66666667	140.6666667	2.617217706	3.240508256	Higher in NONMETA	48	60	373	34	6	19
ENSMUSG00000027333.18	Smox	0.000259929	0.006629114	3	3	8734.666667	4248	4486.666667	1.45029175	1.526381327	Higher in NONMETA	12139	4266	9799	2972	2965	6807
ENSMUSG00000060188.6	Cxcl17	0.000263735	0.006714388	3	3	78.66666667	8.666666667	70	2.755022795	3.561201642	Higher in NONMETA	134	8	94	11	4	11
ENSMUSG00000020696.18	Rffl	0.000278011	0.007020707	3	3	794.6666667	487.3333333	307.3333333	1.255149281	1.303607619	Higher in NONMETA	976	480	928	356	240	866
ENSMUSG00000022223.8	Sdr39u1	0.000278192	0.007020707	3	3	1421	1002.333333	418.6666667	1.077328255	1.107349687	Higher in NONMETA	1225	1166	1872	787	484	1736
ENSMUSG00000006587.6	Snai3	0.000278665	0.007020707	3	3	176	10	166	2.95866181	4.158088029	Higher in NONMETA	31	10	487	9	9	12
ENSMUSG00000052151.12	Plpp2	0.000279444	0.007028156	3	3	6395.666667	3039.333333	3356.333333	1.730993691	1.868542062	Higher in NONMETA	5823	8249	5115	2514	1127	5477
ENSMUSG00000030278.11	Cidec	0.000291431	0.007304361	3	3	1029.666667	165.3333333	864.3333333	2.471557683	2.975762596	Higher in NONMETA	947	132	2010	243	35	218

ENSMUSG00000026450.14	Chit1	0.000295481	0.007380434	3	3	102.3333333	2	100.3333333	3.226013628	5.993744571	Higher in NONMETA	50	0	257	1	1	4
ENSMUSG00000001665.11	Gstt3	0.000298034	0.007431423	3	3	965.6666667	459	506.6666667	1.399586004	1.468975214	Higher in NONMETA	1182	535	1180	349	389	639
ENSMUSG00000039196.2	Orm1	0.000303028	0.007530126	3	3	448.6666667	33.66666667	415	2.944060014	4.126783945	Higher in NONMETA	791	8	547	54	7	40
ENSMUSG00000040154.11	Wfdc5	0.000305142	0.007563995	3	3	48	1	47	3.210630995	5.93851962	Higher in NONMETA	23	1	120	1	0	2
ENSMUSG00000029307.7	Dmp1	0.000305432	0.007563995	3	3	341.6666667	45.33333333	296.3333333	2.67088014	3.384902679	Higher in NONMETA	611	18	396	48	17	71
ENSMUSG00000044405.4	Adig	0.00030825	0.00761125	3	3	368	36.66666667	331.3333333	2.793917959	3.682653561	Higher in NONMETA	192	23	889	45	5	60
ENSMUSG00000073433.10	Arhgdig	0.000308387	0.00761125	3	3	222.6666667	56	166.6666667	2.081159711	2.34754735	Higher in NONMETA	327	186	155	68	54	46
ENSMUSG00000032496.7	Ltf	0.000309547	0.007626914	3	3	9397	888.6666667	8508.333333	2.705932941	3.465934709	Higher in NONMETA	8464	624	19103	1814	377	475
ENSMUSG00000038068.14	Rnf144b	0.00031096	0.007648776	3	3	785	421.3333333	363.6666667	1.446254451	1.523729061	Higher in NONMETA	752	425	1178	299	148	817
ENSMUSG00000016024.9	Lbp	0.000312911	0.007681215	3	3	9883.333333	934.6666667	8948.666667	2.805737642	3.717014874	Higher in NONMETA	22266	567	6817	1803	341	660
ENSMUSG00000027536.6	Chmp4c	0.000313336	0.007681215	3	3	2036.666667	689.6666667	1347	1.755184562	1.902209794	Higher in NONMETA	1685	2132	2293	831	645	593
ENSMUSG00000040471.17	Ggt6	0.000315108	0.007711665	3	3	86.66666667	4.333333333	82.33333333	3.034309212	4.546093896	Higher in NONMETA	174	4	82	9	2	2
ENSMUSG00000041737.8	Tmem45b	0.000326659	0.007927612	3	3	408	32	376	2.885029907	3.961487625	Higher in NONMETA	135	12	1077	35	8	53
ENSMUSG00000027399.1	Il1a	0.000328718	0.007951044	3	3	983.6666667	136	847.6666667	2.526398012	3.094550631	Higher in NONMETA	179	569	2203	250	40	118
ENSMUSG00000028766.10	Alpl	0.000335806	0.008041824	3	3	1123.666667	71	1052.666667	2.892015901	3.993989537	Higher in NONMETA	1358	19	1994	120	58	35
ENSMUSG00000024747.3	Aldh1a7	0.000336135	0.008041824	3	3	180.3333333	11.33333333	169	2.997533532	4.386970032	Higher in NONMETA	194	2	345	14	2	18
ENSMUSG00000032311.17	Nrg4	0.000336344	0.008041824	3	3	128.6666667	31.66666667	97	2.184522813	2.505590621	Higher in NONMETA	124	29	233	19	15	61
ENSMUSG00000017897.18	Eya2	0.000352957	0.008397595	3	3	1161	202	959	2.23766467	2.593470009	Higher in NONMETA	1686	487	1310	204	293	109
ENSMUSG00000021223.13	Papln	0.00035547	0.008443576	3	3	496.3333333	75.66666667	420.6666667	2.5610426	3.174737853	Higher in NONMETA	761	34	694	88	21	118
ENSMUSG00000030546.14	Plin1	0.000359662	0.008529207	3	3	1510.666667	142	1368.666667	2.777759541	3.680053689	Higher in NONMETA	866	57	3609	208	25	193
ENSMUSG00000042510.7	AA986860	0.000363954	0.008601133	3	3	317	105.3333333	211.6666667	2.122360568	2.415917005	Higher in NONMETA	156	515	280	92	37	187
ENSMUSG00000046159.16	Chrm3	0.00036447	0.008601133	3	3	235	20.66666667	214.3333333	2.928714501	4.150788483	Higher in NONMETA	292	5	408	14	2	46
ENSMUSG00000019866.14	Crybg1	0.000366993	0.00864665	3	3	3504.333333	1414.333333	2090	2.013658115	2.256677249	Higher in NONMETA	2350	4253	3910	742	276	3225
ENSMUSG00000028246.13	Faxc	0.000367838	0.008652524	3	3	451.6666667	220	231.6666667	1.666083711	1.792809186	Higher in NONMETA	449	478	428	197	66	397
ENSMUSG00000010064.15	Slc38a3	0.000371656	0.0087282	3	3	284.6666667	33.66666667	251	2.711801409	3.521735064	Higher in NONMETA	568	14	272	42	13	46
ENSMUSG00000020566.19	Atp6v1c2	0.000372544	0.008734959	3	3	4505.666667	181.3333333	4324.333333	3.079084735	4.876263588	Higher in NONMETA	10619	33	2865	431	52	61
ENSMUSG00000026255.15	Efhd1	0.000373578	0.008745085	3	3	107.6666667	32.66666667	75	1.898252846	2.096887184	Higher in NONMETA	110	43	170	40	16	42
ENSMUSG00000029605.16	Oas1b	0.000381164	0.008894412	3	3	571	124	447	2.162898649	2.480855754	Higher in NONMETA	549	89	1075	75	103	194
ENSMUSG00000031134.16	Rbmx	0.000382231	0.008904642	3	3	1610	900.3333333	709.6666667	1.195530828	1.239239796	Higher in NONMETA	1647	814	2369	792	615	1294
ENSMUSG00000047417.17	Rexo1	0.000388881	0.009030607	3	3	2216.333333	1370	846.3333333	1.305844411	1.363804934	Higher in NONMETA	1873	2240	2536	1177	578	2355
ENSMUSG00000039481.5	Nrtn	0.000392797	0.009081486	3	3	1275.666667	98.66666667	1177	2.843023194	3.896003945	Higher in NONMETA	2404	37	1386	200	42	54
ENSMUSG00000003526.11	Prodh	0.000405731	0.00931971	3	3	776	230.3333333	545.6666667	2.052661578	2.318222702	Higher in NONMETA	1565	278	485	190	130	371
ENSMUSG00000075590.2	Nrbp2	0.000410971	0.009382787	3	3	1884.333333	412	1472.333333	2.375663846	2.838882898	Higher in NONMETA	2694	191	2768	248	105	883
ENSMUSG00000028289.12	Epha7	0.000411146	0.009382787	3	3	809.3333333	55.33333333	754	3.014297744	4.593945538	Higher in NONMETA	1899	3	526	33	16	117
ENSMUSG00000029165.16	Agbl5	0.000417343	0.009509281	3	3	707.3333333	368.3333333	339	1.388243895	1.45956827	Higher in NONMETA	937	383	802	376	176	553
ENSMUSG00000034917.8	Tjp3	0.000418032	0.009510069	3	3	741	124.6666667	616.3333333	2.367183026	2.826033895	Higher in NONMETA	570	63	1590	124	69	181
ENSMUSG00000028456.18	Unc13b	0.000422195	0.009559887	3	3	656.6666667	216.3333333	440.3333333	2.060103616	2.33109959	Higher in NONMETA	218	826	926	198	63	388
ENSMUSG00000026821.15	Ralgds	0.000433375	0.009782573	3	3	4504.666667	2918.333333	1586.333333	1.198917276	1.243904675	Higher in NONMETA	5266	2753	5495	2324	1207	5224
ENSMUSG00000030966.13	Trim21	0.000435471	0.009814628	3	3	1420	721.3333333	698.6666667	1.310990449	1.37079406	Higher in NONMETA	1415	640	2205	553	516	1095
ENSMUSG00000079559.4	Colca2	0.000437976	0.009819334	3	3	109.6666667	35.66666667	74	1.784325025	1.95005412	Higher in NONMETA	83	67	179	45	23	39
ENSMUSG00000036304.13	Zdhhc23	0.000438901	0.009819334	3	3	65.66666667	12.33333333	53.33333333	2.532129385	3.146572777	Higher in NONMETA	123	23	51	10	2	25
ENSMUSG00000033208.7	S100b	0.000439038	0.009819334	3	3	480.3333333	71.66666667	408.6666667	2.588886142	3.269102143	Higher in NONMETA	1025	30	386	46	43	126
ENSMUSG00000022899.8	Slc15a2	0.000439052	0.009819334	3	3	2134	514.3333333	1619.666667	2.328155539	2.761882063	Higher in NONMETA	4226	319	1857	307	159	1077
ENSMUSG00000001827.12	Folr1	0.000439057	0.009819334	3	3	319.3333333	43	276.3333333	2.638955933	3.381924696	Higher in NONMETA	425	12	521	34	15	80
ENSMUSG00000024843.15	Chka	0.000447816	0.009984513	3	3	3516	2441.333333	1074.666667	1.10455879	1.139518626	Higher in NONMETA	3789	2887	3872	1607	1437	4280
