# Supplementary material for: Mammary-specific expression of Trim24 establishes a mouse model of human metaplastic breast cancer
Source: Nat Commun. 2021 Sep 10;12:5389. doi: 10.1038/s41467-021-25650-z (PMC8433435; doi:10.1038/s41467-021-25650-z)
Supplement: Supplementary file 12 — Dataset 9 [file 41467_2021_25650_MOESM12_ESM.pdf]

Supplementary Table 9: List of human TNBC and MpBC patient tumor details including response to chemotherapy, percentage of vimentin staining and tumor classification.

Patient ID	Metaplastic	Vimentin (%)	Pathologic Response	Subtype
P01	N	1	RCB - III	UNS
P02	N	0	RCB - II	MSL
P03	N	<1	RCB - II	IM
P04	Y	5	RCB - III	IM
P05	N	1	RCB - I	LAR
P06	N	5	RCB - I	UNS
P07	N	30	pCR	IM
P08	Y	15	RCB - II	IM
P09	Y	15	RCB - II	UNS
P10	N	0	RCB - II	LAR
P11	Y	90	RCB - II	M
P12	N	10	RCB - III	MSL
P13	N	10	RCB - II	BL1
P14	N	1	RCB - III	BL2
P15	N	0		IM
P16	N	0	RCB - II	UNS
P17	N	75	RCB - II	BL1
P18	Y	<1	RCB - II	M
P19	N		RCB - II	BL1
P20	N	2	RCB - III	MSL
P21	N	0	RCB - III	IM
P22	Y	60	RCB - II	M
P23	N	50	RCB - III	BL1
P24	N		RCB - I	BL1
P25	Y	95	RCB - II	MSL
P26	N	0		LAR
P27	Y	90	RCB - II	IM
P28	N	15	RCB - III	BL2

**M***	**Mesenchymal**
**MSL**	**Mesenchymal Stem-Like**
**BL1**	**Basal-Like 1**
**BL2**	**Basal-Like 2**
**LAR**	**Luminal Androgen Receptor**
**IM**	**Immunomodulatory**
**UNS**	**Unspecific**

**pCR**	**pathological Complete Response**
**RCB I-III**	**Residual Cancer Burden (I, II and III)**
