# Supplementary material for: Mammary-specific expression of Trim24 establishes a mouse model of human metaplastic breast cancer
Source: Nat Commun. 2021 Sep 10;12:5389. doi: 10.1038/s41467-021-25650-z (PMC8433435; doi:10.1038/s41467-021-25650-z)
Supplement: Supplementary file 13 — Reporting Summary [file 41467_2021_25650_MOESM13_ESM.pdf]

nature research

Corresponding author(s): Michelle Craig Barton (Primary)
Stacy L. Moulder
Jeffrey T. Chang

Last updated by author(s): 07/01/2021

Reporting Summary

Nature Research wishes to improve the reproducibility of the work that we publish. This form provides structure for consistency and transparency in reporting. For further information on Nature Research policies, see our Editorial Policies and the Editorial Policy Checklist.

Statistics

For all statistical analyses, confirm that the following items are present in the figure legend, table legend, main text, or Methods section.

n/a	Confirmed	
☐	☒	The exact sample size (n) for each experimental group/condition, given as a discrete number and unit of measurement
☐	☒	A statement on whether measurements were taken from distinct samples or whether the same sample was measured repeatedly
☐	☒	The statistical test(s) used AND whether they are one- or two-sided<br>*Only common tests should be described solely by name; describe more complex techniques in the Methods section.*
☒	☐	A description of all covariates tested
☒	☐	A description of any assumptions or corrections, such as tests of normality and adjustment for multiple comparisons
☐	☒	A full description of the statistical parameters including central tendency (e.g. means) or other basic estimates (e.g. regression coefficient) AND variation (e.g. standard deviation) or associated estimates of uncertainty (e.g. confidence intervals)
☐	☒	For null hypothesis testing, the test statistic (e.g. F, t, r) with confidence intervals, effect sizes, degrees of freedom and P value noted<br>*Give P values as exact values whenever suitable.*
☒	☐	For Bayesian analysis, information on the choice of priors and Markov chain Monte Carlo settings
☐	☒	For hierarchical and complex designs, identification of the appropriate level for tests and full reporting of outcomes
☒	☐	Estimates of effect sizes (e.g. Cohen's d, Pearson's r), indicating how they were calculated

Our web collection on statistics for biologists contains articles on many of the points above.

Software and code

Policy information about availability of computer code

Data collection	RNA-Seq, ATAC-Seq and WGS were collected using Illumina Sequencing platforms. CyTOF data were collected using Fluidigm Helios CyTOF Mass Cytometer. CTG analysis was done using FLUOstar Omega Multidetection Microplate Reader (BMG Labtech). IHC stained slides were scanned using Digital Slide Reader (Leica).
Data analysis	GraphPad Prism (version 8 and 9) , FastQC (0.11.8), Trimmomatic (0.39), STAR aligner (2.7.2b) , HTSeq-Count (0.11), Picard (2.9) ,DESeq2 (3.13), ssGSEA (2.0), GSEA (4.1.0) , R (version 3.6), Microsoft Excel (365), Bowtie (version 1.1.2), Integrative Genomics Viewer (IGV) (2.8), , FlowJo (v10.3), CytoBank(v6.7), BWA (v 0.7.15), RSEM (v 1.3.1).

For manuscripts utilizing custom algorithms or software that are central to the research but not yet described in published literature, software must be made available to editors and reviewers. We strongly encourage code deposition in a community repository (e.g. GitHub). See the Nature Research guidelines for submitting code & software for further information.

Data

Policy information about availability of data

All manuscripts must include a data availability statement. This statement should provide the following information, where applicable:

- Accession codes, unique identifiers, or web links for publicly available datasets
- A list of figures that have associated raw data
- A description of any restrictions on data availability

Data Availability
The RNA-Seq data generated for TRIM24-driven mouse model (Figure3) are deposited in the GEO database under accession code GSE179036 . The RNA-Seq data associated with human metaplastic breast cancer patients (Figure 6) are deposited in the GEO database under accession code GSE165407. The ATAC-Seq data of TRIM24-driven metaplastic carcinosarcoma cell line and control cell line (Figure 5) are available at GEO database under accession code GSE149685. Source data are

available as a Source data file. WGS data associated with identification of Flag-tagged mTrim24 transposon in transgenic mice are available at figshare repository (Sample 1 R1 file at doi.org/10.6084/m9.figshare.14818542.v1(2021) , Sample 1 R2 file at doi.org/10.6084/m9.figshare.14818560.v1(2021) , Sample 2 R1 file at doi.org/10.6084/m9.figshare.14818536.v1(2021) and Sample 2 R2 files at doi.org/10.6084/m9.figshare.14818575.v1(2021)). We have used LIEN_BREAST_CARCINOMA_METAPLASTIC_VS_DUCTAL_UP gene set from MSigDB to compare gene expressions of TRIM24-driven metaplastic tumors. The remaining data and information associated with this manuscript are available within the article as a supplementary information.

Field-specific reporting

Please select the one below that is the best fit for your research. If you are not sure, read the appropriate sections before making your selection.

- [x] Life sciences
- [ ] Behavioural & social sciences
- [ ] Ecological, evolutionary & environmental sciences

For a reference copy of the document with all sections, see nature.com/documents/nr-reporting-summary-flat.pdf

Life sciences study design

All studies must disclose on these points even when the disclosure is negative.

Sample size	Sample size for transgenic mice were selected based on the power analysis. Sample size for other experiments were designed based on ref (5-8, 20-21, 58), not predetermined by a statistical method.
Data exclusions	No data related to manuscript were excluded from reporting.
Replication	Statistical analysis was carried out using Prism 8/9 (GraphPad Software). Statistical analysis was conducted on data from three or more biologically independent experimental replicates. Data distribution was assumed to be normal, but this was not formally tested. Comparisons between groups were planned before statistical testing and target effect sizes were not predetermined. Error bars displayed on graphs represent the mean ± SEM of at least three independent experiments and median or mean with SD for 2 biological replicates. We have repeated each experiments with at least 3 biological replicates for all in vitro experiments, IHC and RNA-Seq. For ATAC-Seq and RPPA, we have 2 biological replicates with 2 technical replicates. We have used two-tailed paired t-test, two way ANOVA for multiple comparison using Tukey method or Holm-Sidak method. All tests were two sided.*p<0.05, **p<0.01, and ***p<0.001 were considered significant.
Randomization	Only female mice were used for experiments. Experimental mice and control mice were age matched, hence, no randomization was used. For in vitro studies, samples were randomly allocated into treatment groups.
Blinding	for in vitro and in vivo studies, investigators were blinded for data acquisition and data analysis.

Reporting for specific materials, systems and methods

We require information from authors about some types of materials, experimental systems and methods used in many studies. Here, indicate whether each material, system or method listed is relevant to your study. If you are not sure if a list item applies to your research, read the appropriate section before selecting a response.

Materials & experimental systems

n/a	Involved in the study	
[ ]	[x]	Antibodies
[ ]	[x]	Eukaryotic cell lines
[x]	[ ]	Palaeontology and archaeology
[ ]	[x]	Animals and other organisms
[ ]	[x]	Human research participants
[ ]	[x]	Clinical data
[x]	[ ]	Dual use research of concern

Methods

n/a	Involved in the study	
[x]	[ ]	ChIP-seq
[ ]	[x]	Flow cytometry
[x]	[ ]	MRI-based neuroimaging

Antibodies

Antibodies used	Flag (Sigma), ER (Santa Cruz), PR (Abcam), E-cadherin (Proteintech), Vimentin (Abcam), human mitochondria (Chemicon # MAB1273), and TRIM24 (Proteintech), Keratin 8 (Sigma), Keratin 14 (Covance)
Validation	All antibodies were validated with negative and positive controls, as appropriate for the application based on manufacturer's recommendation on website. CyTOF antibodies were developed as derivatized reagents with in-house negative and positive controls (ref 66) . Validation statement for each primary antibody is provided on the manufacturer's website.

Eukaryotic cell lines

Policy information about cell lines

Cell line source(s)	The cell lines 64, 567, and 897 were generated from TRIM24-driven carcinosarcoma tumors. Primary cell line 823 was generated from a spontaneous mammary tumor generated in MMTV-Cre mouse and used as a control. 4T1, derived from mouse mammary breast tumor, cell line was purchased from ATCC (CRL-2539). 293FT cells were purchased from Thermo Fisher Scientific (R70007)
Authentication	4T1 cell line was grown as per manufacturer's recommendation and morphology was confirmed as described. The cell lines prepared in the lab were grown into tumors by injecting in nude mammary fat pad and tumors generated from them were validated as carcinosarcoma, hence, cell lines are validated. 293FT cells were used for transfection/transduction as per manufacturer's recommendation (https://www.thermofisher.com/us/en/home/life-science/cell-culture/cell-culture-learning-center/cell-culture-resource-library/cell-culture-transfection-application-notes/improve-lentiviral-production-using-lipofectamine-3000-reagent.html).
Mycoplasma contamination	All the cell lines used in the experiments were tested for Mycoplasma and found negative. Murine cell lines derived from TRIM24-driven tumors were also tested using an external service - IMPACT mouse (Idexx Bioanalytics).
Commonly misidentified lines (See ICLAC register)	No commonly misidentified lines were used.

Animals and other organisms

Policy information about studies involving animals; ARRIVE guidelines recommended for reporting animal research

Laboratory animals	Mus musculus. To generate transgenic mouse line, we have used FVB/NCr (Charles River Laboratories) mice, females 2-3 months old . To activate the transgene, we bred our transgenic line to the Tg(MMTV-cre)4Mam (The Jackson laboratory, stock 003553). for crosses, males 2-4 months old, females 3-6 months old. Aging cohort consist of virgin females was kept for 600 days or till tumor burden was detected. For allograft tumors, we used Foxn1nu or athymic female mice (6-8 weeks old). Mice were maintained in air-filtered cages with controlled temperature (20 °C) and humidity (50%), in a 12 h light/dark cycle, and fed standard mouse chow (Research Diets; New Brunswick, NJ).
Wild animals	No wild animals were used in this study.
Field-collected samples	No field-collected samples were used in this study.
Ethics oversight	Animal handling and experiments were performed as approved by the Institutional Animal Care and Use Committee at the MD Anderson Cancer Center.

Note that full information on the approval of the study protocol must also be provided in the manuscript.

Human research participants

Policy information about studies involving human research participants

Population characteristics	Data from 28 patients enrolled on the ARTEMIS study were included in this manuscript. All 28 patients were diagnosed with stage I-III triple-negative breast cancer and enrolled on this study from 1/29/2016 through 11/2/2017.
Recruitment	Patients with stage I-III triple-negative breast cancer were enrolled on this study and underwent a pre-treatment core-needle biopsy prior to initiation of neoadjuvant systemic therapy.
Ethics oversight	The ARTEMIS study protocol was reviewed by The University of Texas MD Anderson Cancer Center Institutional Review Board and all patients provided informed consent. All study procedures performed were in accordance with ethical standard of the Institutional Review Board and with the 1964 Helsinki declaration and its later amendments or comparable ethical standards.

Note that full information on the approval of the study protocol must also be provided in the manuscript.

Clinical data

Policy information about clinical studies

All manuscripts should comply with the ICMJEguidelines for publication of clinical research and a completedCONSORT checklist must be included with all submissions.

Clinical trial registration	NCT02276443
Study protocol	The study is ongoing and the protocol can be made available by contacting the study PI (cyam@mdanderson.org)
Data collection	All patients received medical care at The University of Texas MD Anderson Cancer Center. Data was collected during follow up visits at The University of Texas MD Anderson Cancer Center. Where applicable, medical records were obtained from other medical centers when patients received part of their care at another location.
Outcomes	The primary endpoint of this study is pathologic response at the time of surgery using the residual cancer burden (RCB) index as part of routine clinical care (PMID: 17785706).

Flow Cytometry

Plots

Confirm that:

- [ ] The axis labels state the marker and fluorochrome used (e.g. CD4-FITC).
- [ ] The axis scales are clearly visible. Include numbers along axes only for bottom left plot of group (a 'group' is an analysis of identical markers).
- [x] All plots are contour plots with outliers or pseudocolor plots.
- [x] A numerical value for number of cells or percentage (with statistics) is provided.

Methodology

Sample preparation	Mammary allogrft tumors were digested into single cells in DMEM/F12 media supplemented with hyaluronidase (0.5mg/mL) and Collagenase Type IV (3mg/mL) for 30 minutes. For more details, see Methods.
Instrument	CyTOF Helios with software version 6.7
Software	For data analysis, FlowJo_v10.3 was used to remove beads, debris and to obtain single cells. This was followed by CytoBank to generate viSNE plots.
Cell population abundance	Not applicable for CyTOF analysis.
Gating strategy	Ce140 (beads)/Iridium191 (DNA) was gated to remove beads. Event length/ Iridium193(DNA) was gated to remove debris and cell multiplexes. Samples were then separated based on the metal tagging (barcode) (194Pt, 195Pt and 196Pt).<br>ViSNE clusters were based on EMT markers.

- [ ] Tick this box to confirm that a figure exemplifying the gating strategy is provided in the Supplementary Information.
