# Supplementary figures and images for: Mammary-specific expression of Trim24 establishes a mouse model of human metaplastic breast cancer
Source: Nat Commun. 2021 Sep 10;12:5389. doi: 10.1038/s41467-021-25650-z (PMC8433435; doi:10.1038/s41467-021-25650-z)

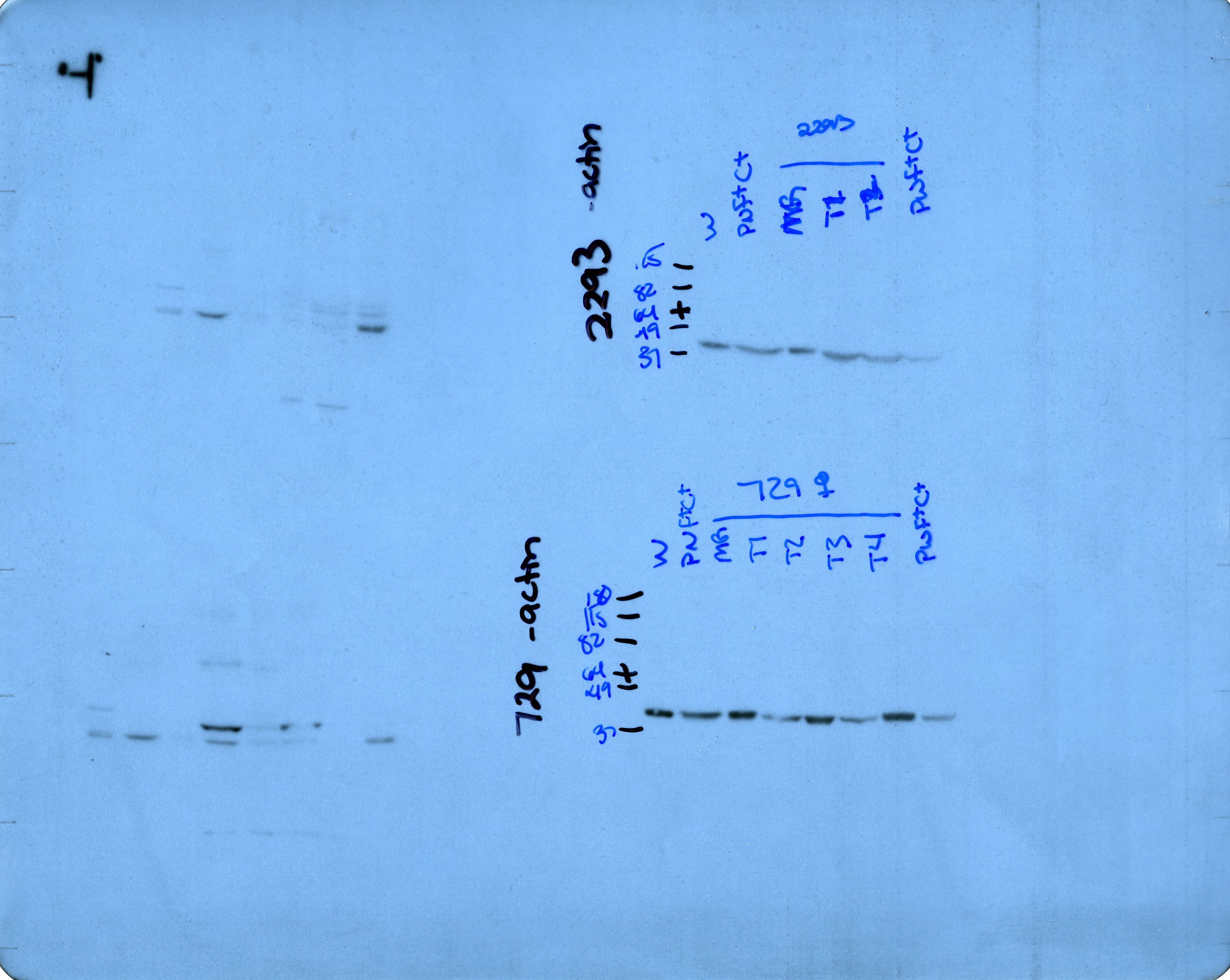

Supplement: Supplementary file 14 — Source Data [file 41467_2021_25650_MOESM14_ESM.zip › Source Data/WB_figS1d_bactin.jpg]

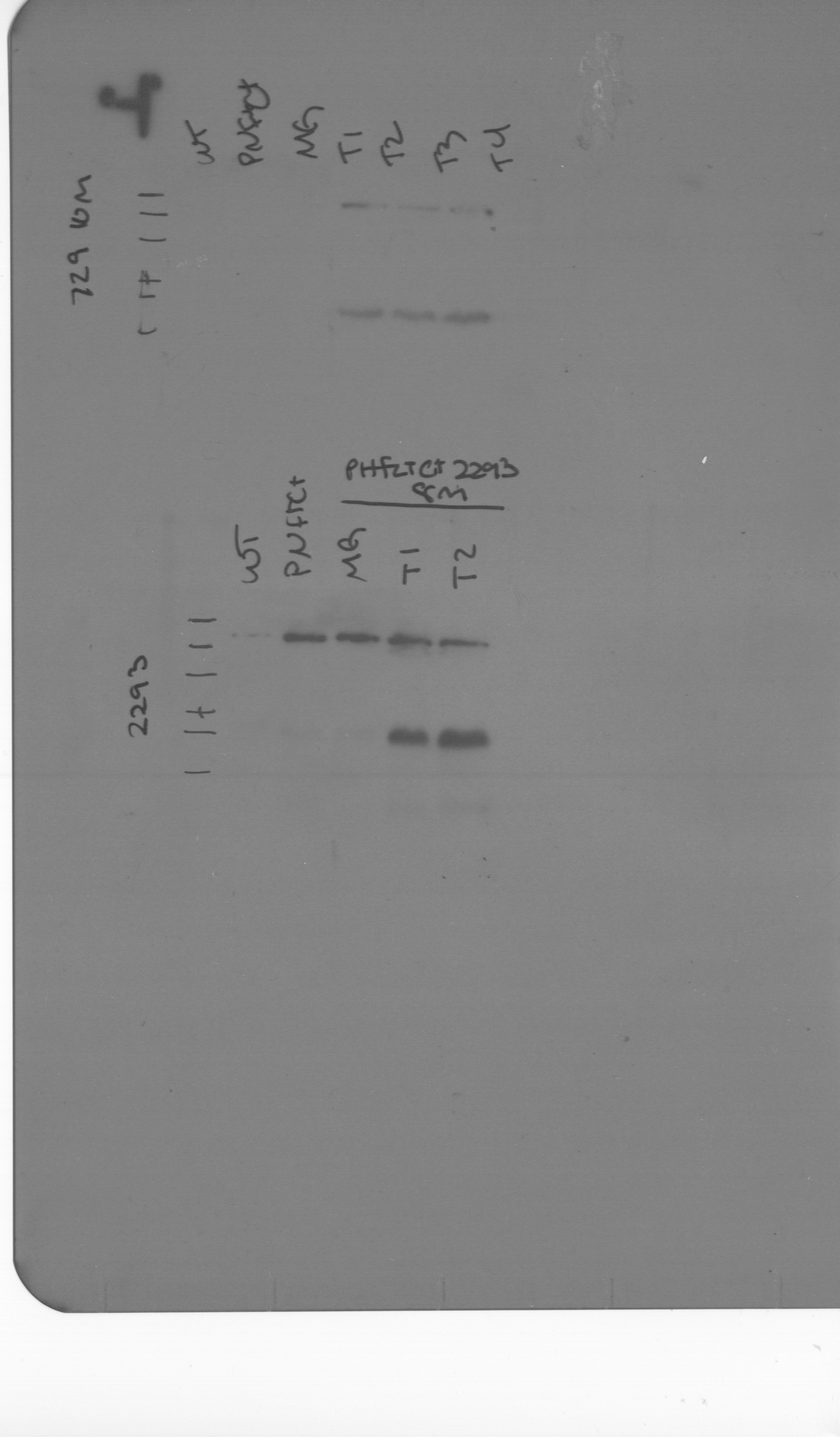

Supplement: Supplementary file 14 — Source Data [file 41467_2021_25650_MOESM14_ESM.zip › Source Data/WB_figS1d_FLAG.tiff]

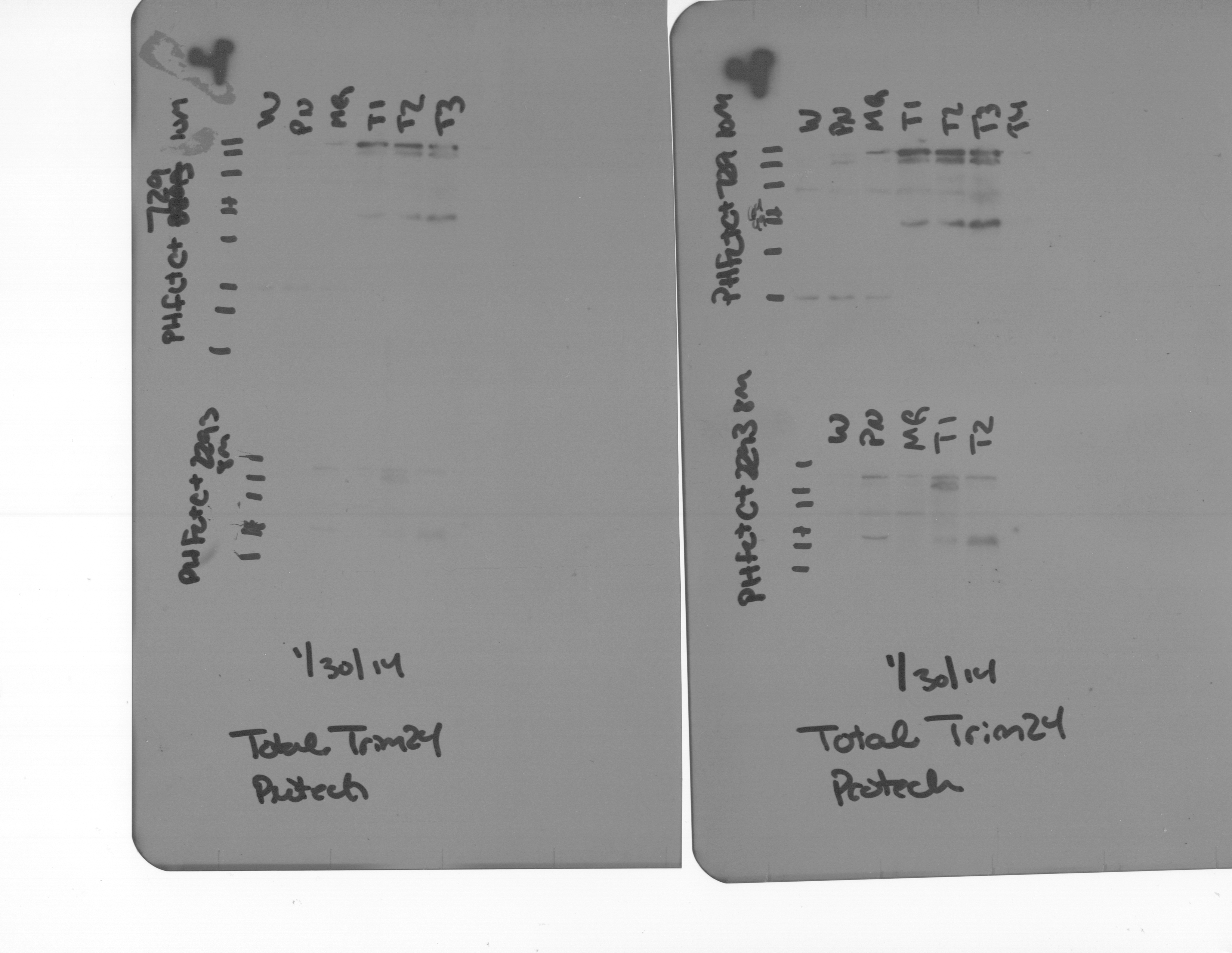

Supplement: Supplementary file 14 — Source Data [file 41467_2021_25650_MOESM14_ESM.zip › Source Data/WB_figS1d_TRIM24.tiff]
